# Supplementary material for: COVID-19 in patients with hepatobiliary and pancreatic diseases: a single-centre cross-sectional study in East London
Source: BMJ Open. 2021 Apr 19;11(4):e045077. doi: 10.1136/bmjopen-2020-045077 (PMC8057071; doi:10.1136/bmjopen-2020-045077)
Supplement: Supplementary data [file bmjopen-2020-045077supp001.pdf]

Supplemental Table 1 Codelist for hepato-pancreato-biliary diagnosis groups

Group	Subgroup	Terminology system	Code	Code description	Exclusion
HPB Cancer		ICD-10	C22	Malignant neoplasm of liver and intrahepatic bile ducts	
HPB Cancer		ICD-10	C23	Malignant neoplasm of gallbladder	
HPB Cancer		ICD-10	C24	Malignant neoplasm of other and unspecified parts of biliary tract	
HPB Cancer		ICD-10	C25	Malignant neoplasm of pancreas	
HPB Cancer		ICD-10	D015	Carcinoma in situ of Liver, gallbladder and bile ducts	
HPB Cancer		ICD-10	D017	Carcinoma in situ of Other specified digestive organs incl. Pancreas	
HPB Cancer		ICD-10	D376	Neoplasm of uncertain or unknown behaviour of Liver, gallbladder and bile ducts	
HPB Cancer		READ	B15	Malignant neoplasm of liver and intrahepatic bile ducts	
HPB Cancer		READ	B16	Malignant neoplasm gallbladder and extrahepatic bile ducts	
HPB Cancer		READ	B17	Malignant neoplasm of pancreas	
HPB Cancer		READ	B808.	Carcinoma in situ of liver and biliary system	
HPB Cancer		READ	B8080	Carcinoma in situ of liver	
HPB Cancer		READ	B8081	Carcinoma in situ of intrahepatic bile ducts	
HPB Cancer		READ	B8082	Carcinoma in situ of hepatic duct	
HPB Cancer		READ	B8083	Carcinoma in situ of gall bladder	
HPB Cancer		READ	B8085	Carcinoma in situ of common bile duct	
HPB Cancer		READ	B8086	Carcinoma in situ of ampulla of Vater	
HPB Cancer		READ	B8087	Carcinoma in situ of sphincter of Oddi	
HPB Cancer		READ	B80z0	Carcinoma in situ of pancreas	
HPB Cancer		READ	B903.	Neoplasm of uncertain behaviour of liver and biliary passage	
HPB Cancer		READ	B9030	Neoplasm of uncertain behaviour of liver	
HPB Cancer		READ	B9031	Neoplasm of uncertain behaviour of intra-hepatic bile ducts	

HPB Cancer	READ	B9032	Neoplasm of uncertain behaviour of hepatic duct	
HPB Cancer	READ	B9033	Neoplasm of uncertain behaviour of gall bladder	
HPB Cancer	READ	B9034	Neoplasm of uncertain behaviour of cystic duct	
HPB Cancer	READ	B9035	Neoplasm of uncertain behaviour of common bile duct	
HPB Cancer	READ	B9036	Neoplasm of uncertain behaviour of ampulla of Vater	
HPB Cancer	READ	B9037	Neoplasm of uncertain behaviour of sphincter of Oddi	
HPB Cancer	READ	B9051	Neoplasm of uncertain behaviour of pancreas	
HPB Cancer	READ	Byu10	[X]Other sarcomas of the liver	
HPB Cancer	READ	Byu11	[X]Other specified carcinomas of liver	
HPB Cancer	READ	Byu12	[X]Malignant neoplasm of intestinal tract, part unspecified	
HPB Cancer	SNOMED CT	92545000	Carcinoma in situ of biliary tract (disorder)	
HPB Cancer	SNOMED CT	92644006	Carcinoma in situ of liver (disorder)	
HPB Cancer	SNOMED CT	92672004	Carcinoma in situ of pancreas (disorder)	
HPB Cancer	SNOMED CT	93870000	Malignant neoplasm of liver (disorder)	94381002 (metastasis to liver)
HPB Cancer	SNOMED CT	94910002	Neoplasm of uncertain behavior of liver (disorder)	
HPB Cancer	SNOMED CT	94978003	Neoplasm of uncertain behavior of pancreas (disorder)	
HPB Cancer	SNOMED CT	255064003	Neoplasm of uncertain behavior of biliary system (disorder)	
HPB Cancer	SNOMED CT	363415003	Malignant tumor of biliary tract (disorder)	94185003 (metastasis to biliary tract)
HPB Cancer	SNOMED CT	363418001	Malignant tumor of pancreas (disorder)	94459006 (metastasis to pancreas)
HPB Cancer	CTV3	B15..	Malignant neoplasm of liver and intrahepatic bile ducts	
HPB Cancer	CTV3	B16..	Malignant tumour of biliary tract	
HPB Cancer	CTV3	B162.	Malignant tumour of ampulla of Vater	
HPB Cancer	CTV3	B17..	Malignant tumour of pancreas	B162. (ampullary tumor) X78kd (metastasis to pancreas)

HPB Cancer		CTV3	B80z0	Carcinoma in situ of pancreas	B8086 (ampullary carcinoma in situ)
HPB Cancer		CTV3	B9030	Neoplasm of uncertain behaviour of liver	
HPB Cancer		CTV3	B9031	Neoplasm of uncertain behaviour of intrahepatic bile ducts	
HPB Cancer		CTV3	B9032	Neoplasm of uncertain behaviour of hepatic duct	
HPB Cancer		CTV3	B9033	Neoplasm of uncertain behaviour of gallbladder	
HPB Cancer		CTV3	B9034	Neoplasm of uncertain behaviour of cystic duct	
HPB Cancer		CTV3	B9035	Neoplasm of uncertain behaviour of common bile duct	
HPB Cancer		CTV3	B9036	Neoplasm of uncertain behaviour of ampulla of Vater	
HPB Cancer		CTV3	B9037	Neoplasm of uncertain behaviour of sphincter of Oddi	
HPB Cancer		CTV3	B903z	Neop of uncertain behaviour of liver or biliary passages NOS	
HPB Cancer		CTV3	B9051	Neoplasm of uncertain behaviour of pancreas	
HPB Cancer		CTV3	X78ed	Neoplasm of uncertain behaviour of biliary system	
HPB Cancer		CTV3	X78mC	Carcinoma in situ of biliary tract	
HPB Cancer		CTV3	Xa97q	Malignant tumour of liver	
HPB Cancer		CTV3	XE2ve	Neoplasm of uncertain behaviour of liver and biliary passage	
Pancreatic disease	Acute	ICD-10	K85	Acute pancreatitis	
Pancreatic disease	Acute	ICD-10	K871	Disorders of pancreas in diseases classified elsewhere	
Pancreatic disease	Acute	READ	J670	Acute pancreatitis	
Pancreatic disease	Acute	SNOMED CT	39205007	Infectious pancreatitis (disorder)	838375006 (Chronic)
Pancreatic disease	Acute	SNOMED CT	197456007	Acute pancreatitis (disorder)	
Pancreatic disease	Acute	CTV3	J670.	Acute pancreatitis	
Pancreatic disease	Acute	CTV3	J6704	Subacute pancreatitis	
Pancreatic disease	Acute	CTV3	J670z	Pancreatitis (& [acute NOS])	
Pancreatic disease	Acute	CTV3	Jyu87	[X]Disorders of pancreas in diseases classified elsewhere	
Pancreatic disease	Acute	CTV3	X3092	Pancreatic abscess	
Pancreatic disease	Chronic	ICD-10	D136	Benign neoplasm of Pancreas excl. Endocrine pancreas	

Pancreatic disease	Chronic	ICD-10	D137	Benign neoplasm of Endocrine pancreas
Pancreatic disease	Chronic	ICD-10	K86	Other diseases of pancreas
Pancreatic disease	Chronic	ICD-10	Q450	Agenesis, aplasia and hypoplasia of pancreas
Pancreatic disease	Chronic	ICD-10	Q451	Annular pancreas
Pancreatic disease	Chronic	ICD-10	Q452	Congenital pancreatic cyst
Pancreatic disease	Chronic	ICD-10	Q453	Other congenital malformations of pancreas and pancreatic duct
Pancreatic disease	Chronic	READ	B716	Benign neoplasm of pancreas, excluding islets of Langerhans
Pancreatic disease	Chronic	READ	B717	Benign neoplasm of islets of Langerhans
Pancreatic disease	Chronic	READ	J671	Chronic pancreatitis
Pancreatic disease	Chronic	READ	J672	Cyst and pseudocyst of pancreas
Pancreatic disease	Chronic	READ	J67y	Other diseases of pancreas
Pancreatic disease	Chronic	READ	J67z.	Diseases of pancreas NOS
Pancreatic disease	Chronic	READ	PB7	Anomalies of pancreas
Pancreatic disease	Chronic	SNOMED CT	1835003	Necrosis of pancreas (disorder)
Pancreatic disease	Chronic	SNOMED CT	15402006	Calculus of pancreas (disorder)
Pancreatic disease	Chronic	SNOMED CT	25942009	Fibrosis of pancreas (disorder)
Pancreatic disease	Chronic	SNOMED CT	31258000	Cyst of pancreas (disorder)
Pancreatic disease	Chronic	SNOMED CT	37992001	Pancreatic insufficiency (disorder)
Pancreatic disease	Chronic	SNOMED CT	88281007	Atrophy of pancreas (disorder)
Pancreatic disease	Chronic	SNOMED CT	92264007	Benign neoplasm of pancreas (disorder)
Pancreatic disease	Chronic	SNOMED CT	235494005	Chronic pancreatitis (disorder)
Pancreatic disease	Chronic	SNOMED CT	235977001	Congenital malformation of pancreas (disorder)
Pancreatic disease	Chronic	SNOMED CT	838375006	Chronic infectious pancreatitis (disorder)
Pancreatic disease	Chronic	CTV3	J671.	Chronic pancreatitis
Pancreatic disease	Chronic	CTV3	J672.	Cyst and pseudocyst of pancreas
Pancreatic disease	Chronic	CTV3	J6720	Pancreatic cyst
Pancreatic disease	Chronic	CTV3	J6721	Pseudocyst of pancreas

Pancreatic disease	Chronic	CTV3	J67y0	Atrophy of pancreas
Pancreatic disease	Chronic	CTV3	J67y1	Calculus of pancreas
Pancreatic disease	Chronic	CTV3	J67y2	Fibrosis of pancreas
Pancreatic disease	Chronic	CTV3	J67z.	Diseases of pancreas NOS
Pancreatic disease	Chronic	CTV3	X3093	Pancreatic and peripancreatic necrosis
Pancreatic disease	Chronic	CTV3	X309O	Pancreatic insufficiency
Pancreatic disease	Chronic	CTV3	X309P	Congenital abnormality of pancreas
Pancreatic disease	Chronic	CTV3	X78oE	Benign tumour of pancreas
Pancreatic disease	Chronic	CTV3	XE0dV	(Disease pancreas NOS) or (cyst pancr) or (pseudocyst pancr)
Biliary disease	Acute	ICD-10	K800	Calculus of gallbladder with acute cholecystitis
Biliary disease	Acute	ICD-10	K803	Calculus of bile duct with cholangitis
Biliary disease	Acute	ICD-10	K804	Calculus of bile duct with cholecystitis
Biliary disease	Acute	ICD-10	K810	Acute cholecystitis
Biliary disease	Acute	ICD-10	K820	Obstruction of gallbladder
Biliary disease	Acute	ICD-10	K821	Hydrops of gallbladder
Biliary disease	Acute	ICD-10	K822	Perforation of gallbladder
Biliary disease	Acute	ICD-10	K823	Fistula of gallbladder
Biliary disease	Acute	ICD-10	K830	Cholangitis
Biliary disease	Acute	ICD-10	K831	Obstruction of bile duct
Biliary disease	Acute	ICD-10	K832	Perforation of bile duct
Biliary disease	Acute	ICD-10	K833	Fistula of bile duct
Biliary disease	Acute	READ	J640	Gallbladder calculus with acute cholecystitis
Biliary disease	Acute	READ	J643	Bile duct calculus with acute cholecystitis
Biliary disease	Acute	READ	J644	Bile duct calculus with other cholecystitis
Biliary disease	Acute	READ	J646	Calculus of bile duct with cholangitis
Biliary disease	Acute	READ	J650	Acute cholecystitis
Biliary disease	Acute	READ	J652	Obstruction of gallbladder

Biliary disease	Acute	READ	J653	Mucocele of gallbladder	
Biliary disease	Acute	READ	J654	Perforation of gallbladder	
Biliary disease	Acute	READ	J655	Fistula of gallbladder	
Biliary disease	Acute	READ	J661	Cholangitis	
Biliary disease	Acute	READ	J662	Obstruction of bile duct	
Biliary disease	Acute	READ	J663	Perforation of bile duct	
Biliary disease	Acute	READ	J664	Fistula of bile duct	
Biliary disease	Acute	READ	J666.	Biliary sepsis	
Biliary disease	Acute	SNOMED CT	6215006	Acute cholangitis (disorder)	
Biliary disease	Acute	SNOMED CT	16957005	Fistula of gallbladder (disorder)	
Biliary disease	Acute	SNOMED CT	25345001	Perforation of gallbladder	
Biliary disease	Acute	SNOMED CT	30093007	Calculus of bile duct (disorder)	91316003 (with chronic cholecystitis)
Biliary disease	Acute	SNOMED CT	30144000	Obstruction of bile duct (disorder)	68368005 (with chronic cholecystitis) 4661003 (calculus of bile duct)
Biliary disease	Acute	SNOMED CT	37439003	Perforation of bile duct (disorder)	
Biliary disease	Acute	SNOMED CT	47312008	Hydrops of gallbladder (disorder)	
Biliary disease	Acute	SNOMED CT	53206008	Fistula of bile duct (disorder)	
Biliary disease	Acute	SNOMED CT	59771005	Calculus of gallbladder with acute cholecystitis (disorder)	
Biliary disease	Acute	SNOMED CT	68368005	Calculus of common bile duct with chronic cholecystitis (disorder)	
Biliary disease	Acute	SNOMED CT	75726005	Obstruction of gallbladder (disorder)	
Biliary disease	Acute	SNOMED CT	750511000000101	Biliary sepsis (disorder)	
Biliary disease	Acute	CTV3	J640.	Gallbladder calculus with acute cholecystitis	
Biliary disease	Acute	CTV3	J643.	Bile duct calculus with acute cholecystitis	
Biliary disease	Acute	CTV3	J650.	(Ac cholecystitis) or (empyema gallblad) or (absc gallblad)	
Biliary disease	Acute	CTV3	J653.	(Mucocele of gallbladder) or (hydrops of gallbladder)	

Biliary disease	Acute	CTV3	J655.	Fistula of gallbladder
Biliary disease	Acute	CTV3	J6550	Biliary tract fistula
Biliary disease	Acute	CTV3	J65y4	Cyst of gallbladder
Biliary disease	Acute	CTV3	J661.	Cholangitis
Biliary disease	Acute	CTV3	X3087	Obstruction of biliary tree
Biliary disease	Acute	CTV3	X308B	Empyema of gallbladder
Biliary disease	Acute	CTV3	X308C	Hydrops of gallbladder
Biliary disease	Acute	CTV3	X308E	Biliary stricture
Biliary disease	Acute	CTV3	X308P	Perforation of biliary tree
Biliary disease	Acute	CTV3	X308V	Obstructive jaundice
Biliary disease	Acute	CTV3	XaAzZ	Dilation of biliary tract
Biliary disease	Acute	CTV3	XaWzz	Biliary sepsis
Biliary disease	Acute	CTV3	XE0bF	Acute cholecystitis
Biliary disease	Acute	CTV3	XE0bG	Mucocele of gallbladder
Biliary disease	Chronic	ICD-10	D135	Benign neoplasm of Extrahepatic bile ducts
Biliary disease	Chronic	ICD-10	K801	Calculus of gallbladder with other cholecystitis
Biliary disease	Chronic	ICD-10	K802	Calculus of gallbladder without cholecystitis
Biliary disease	Chronic	ICD-10	K805	Calculus of bile duct without cholangitis or cholecystitis
Biliary disease	Chronic	ICD-10	K808	Other cholelithiasis
Biliary disease	Chronic	ICD-10	K811	Chronic cholecystitis
Biliary disease	Chronic	ICD-10	K818	Other cholecystitis
Biliary disease	Chronic	ICD-10	K819	Cholecystitis, unspecified
Biliary disease	Chronic	ICD-10	K824	Cholesterolosis of gallbladder
Biliary disease	Chronic	ICD-10	K828	Other specified diseases of gallbladder
Biliary disease	Chronic	ICD-10	K829	Disease of gallbladder, unspecified
Biliary disease	Chronic	ICD-10	K834	Spasm of sphincter of Oddi
Biliary disease	Chronic	ICD-10	K835	Biliary cyst
Biliary disease	Chronic	ICD-10	K838	Other specified diseases of biliary tract

Biliary disease	Chronic	ICD-10	K839	Disease of biliary tract, unspecified
Biliary disease	Chronic	ICD-10	Q440	Agenesis, aplasia and hypoplasia of gallbladder
Biliary disease	Chronic	ICD-10	Q441	Other congenital malformations of gallbladder
Biliary disease	Chronic	ICD-10	Q442	Atresia of bile ducts
Biliary disease	Chronic	ICD-10	Q443	Congenital stenosis and stricture of bile ducts
Biliary disease	Chronic	ICD-10	Q444	Choledochal cyst
Biliary disease	Chronic	ICD-10	Q445	Other congenital malformations of bile ducts
Biliary disease	Chronic	READ	B715.	Benign neoplasm of liver and biliary ducts
Biliary disease	Chronic	READ	B7152	Benign neoplasm of gallbladder
Biliary disease	Chronic	READ	B7155	Benign neoplasm of bile duct
Biliary disease	Chronic	READ	B7156	Benign neoplasm of sphincter of Oddi
Biliary disease	Chronic	READ	B7157	Benign neoplasm of ampulla of Vater
Biliary disease	Chronic	READ	J64..	Cholelithiasis
Biliary disease	Chronic	READ	J641	Gallbladder calculus with other cholecystitis
Biliary disease	Chronic	READ	J642	Gallbladder calculus without mention of cholecystitis
Biliary disease	Chronic	READ	J645	Bile duct calculus without mention of cholecystitis
Biliary disease	Chronic	READ	J64z	Cholelithiasis NOS
Biliary disease	Chronic	READ	J65..	Other gallbladder disorders
Biliary disease	Chronic	READ	J651	Other cholecystitis
Biliary disease	Chronic	READ	J656	Cholesterolosis of gallbladder
Biliary disease	Chronic	READ	J65y	Other specified gallbladder disorders
Biliary disease	Chronic	READ	J65z.	Other gallbladder disorders NOS
Biliary disease	Chronic	READ	J665	Spasm of sphincter of Oddi
Biliary disease	Chronic	READ	J66y	Other bile duct disorders
Biliary disease	Chronic	READ	J66z.	Bile duct disorder NOS
Biliary disease	Chronic	READ	PB601	Gallbladder anomaly, unspecified
Biliary disease	Chronic	READ	PB602	Bile duct anomaly, unspecified
Biliary disease	Chronic	READ	PB61	Biliary atresia

Biliary disease	Chronic	READ	PB640	Duplication of biliary duct
Biliary disease	Chronic	READ	PB641	Duplication of cystic duct
Biliary disease	Chronic	READ	PB642	Duplication of gallbladder
Biliary disease	Chronic	READ	PB6y0	Congenital choledochal cyst
Biliary disease	Chronic	READ	PB6y1	Congenital hepatomegaly
Biliary disease	Chronic	READ	PB6y2	Congenital floating gallbladder
Biliary disease	Chronic	READ	PB6y4	Intrahepatic gallbladder
Biliary disease	Chronic	READ	PB6y5	Hypoplasia of gallbladder
Biliary disease	Chronic	READ	PB6y7	Congenital dilation of bile duct
Biliary disease	Chronic	READ	PB6y8	Congenital diverticulum of bile duct
Biliary disease	Chronic	READ	PB6yx	Other congenital anomaly of gallbladder
Biliary disease	Chronic	SNOMED CT	1698001	Ulcer of bile duct (disorder)
Biliary disease	Chronic	SNOMED CT	4711003	Congenital anomaly of bile ducts (disorder)
Biliary disease	Chronic	SNOMED CT	13516000	Adhesion of gallbladder (disorder)
Biliary disease	Chronic	SNOMED CT	26874005	Hypertrophy of bile duct (disorder)
Biliary disease	Chronic	SNOMED CT	28132005	Spasm of sphincter of Oddi (disorder)
Biliary disease	Chronic	SNOMED CT	49714001	Congenital anomaly of gallbladder (disorder)
Biliary disease	Chronic	SNOMED CT	51854002	Atrophy of bile duct (disorder)
Biliary disease	Chronic	SNOMED CT	59612001	Ulcer of gallbladder (disorder)
Biliary disease	Chronic	SNOMED CT	61565001	Cholesterolosis of gallbladder (disorder)
Biliary disease	Chronic	SNOMED CT	64664008	Atrophy of gallbladder (disorder)
Biliary disease	Chronic	SNOMED CT	71912000	Chronic cholangitis (disorder)
Biliary disease	Chronic	SNOMED CT	76875008	Hypertrophy of gallbladder (disorder)
Biliary disease	Chronic	SNOMED CT	77972001	Adhesion of bile duct (disorder)
Biliary disease	Chronic	SNOMED CT	78900008	Nonfunctioning cystic duct (disorder)
Biliary disease	Chronic	SNOMED CT	80527006	Nonfunctioning gallbladder (disorder)
Biliary disease	Chronic	SNOMED CT	91316003	Calculus of bile duct with chronic cholecystitis (disorder)
Biliary disease	Chronic	SNOMED CT	91991003	Benign neoplasm of biliary tract (disorder)

Biliary disease	Chronic	SNOMED CT	95559000	Chronic cholecystitis with calculus (disorder)
Biliary disease	Chronic	SNOMED CT	204787003	Congenital absence of liver and/or gallbladder (disorder)
Biliary disease	Chronic	SNOMED CT	235924006	Cyst of biliary tract (disorder)
Biliary disease	Chronic	SNOMED CT	253804002	Biliary anomalies (disorder)
Biliary disease	Chronic	SNOMED CT	721721001	Dyskinesia of gallbladder (disorder)
Biliary disease	Chronic	SNOMED CT	722869007	Calculus of gallbladder without cholecystitis or cholangitis (disorder)
Biliary disease	Chronic	CTV3	B715.	Benign neoplasm: [liver & biliary ducts] or [biliary system]
Biliary disease	Chronic	CTV3	J641.	Gallbladder calculus with other cholecystitis
Biliary disease	Chronic	CTV3	J642.	Gallbladder calculus without mention of cholecystitis
Biliary disease	Chronic	CTV3	J644.	Bile duct calculus with other cholecystitis
Biliary disease	Chronic	CTV3	J645.	Bile duct calculus (& [without mention of cholecystitis])
Biliary disease	Chronic	CTV3	J64z.	Cholelithiasis NOS
Biliary disease	Chronic	CTV3	J651.	Other cholecystitis
Biliary disease	Chronic	CTV3	J6510	Chronic cholecystitis
Biliary disease	Chronic	CTV3	J651y	Other cholecystitis OS
Biliary disease	Chronic	CTV3	J651z	Cholecystitis NOS
Biliary disease	Chronic	CTV3	J656.	Cholesterolosis of gallbladder
Biliary disease	Chronic	CTV3	J65y8	Ulcer of gallbladder
Biliary disease	Chronic	CTV3	J65y9	Ulcer of cystic duct
Biliary disease	Chronic	CTV3	J65yA	Non-functioning gallbladder
Biliary disease	Chronic	CTV3	J65yz	Other specified gallbladder disorder NOS
Biliary disease	Chronic	CTV3	J65z.	Other gallbladder disorders NOS
Biliary disease	Chronic	CTV3	J665.	Spasm of sphincter of Oddi
Biliary disease	Chronic	CTV3	Jyu80	[X]Other cholelithiasis
Biliary disease	Chronic	CTV3	Jyu81	[X]Other cholecystitis
Biliary disease	Chronic	CTV3	Jyu82	[X]Other specified diseases of gallbladder
Biliary disease	Chronic	CTV3	X3082	Congenital disorder of gallbladder and biliary tract

Biliary disease	Chronic	CTV3	X308F	Biliary cyst
Biliary disease	Chronic	CTV3	X308L	Cyst of biliary tract
Biliary disease	Chronic	CTV3	X308Q	Adhesions of biliary tree
Biliary disease	Chronic	CTV3	X308R	Atrophy of biliary tree
Biliary disease	Chronic	CTV3	X308S	Hypertrophy of biliary tract
Biliary disease	Chronic	CTV3	X308U	Ulceration of biliary tree
Biliary disease	Chronic	CTV3	X78oB	Benign tumour of biliary tract
Biliary disease	Chronic	CTV3	Xa4g2	Poorly functioning gallbladder
Biliary disease	Chronic	CTV3	XE0bE	Bile duct calculus without mention of cholecystitis
Biliary disease	Chronic	CTV3	XE2xC	Benign neoplasm of liver and biliary ducts
Liver disease	Mild	ICD-10	B18	Chronic viral hepatitis
Liver disease	Mild	ICD-10	B18	Chronic viral hepatitis
Liver disease	Mild	ICD-10	D134	Benign neoplasm of Liver
Liver disease	Mild	ICD-10	K700	Alcoholic fatty liver
Liver disease	Mild	ICD-10	K701	Alcoholic hepatitis
Liver disease	Mild	ICD-10	K702	Alcoholic fibrosis and sclerosis of liver
Liver disease	Mild	ICD-10	K703	Alcoholic cirrhosis of liver
Liver disease	Mild	ICD-10	K709	Alcoholic liver disease, unspecified
Liver disease	Mild	ICD-10	K713	Toxic liver disease with chronic persistent hepatitis
Liver disease	Mild	ICD-10	K714	Toxic liver disease with chronic lobular hepatitis
Liver disease	Mild	ICD-10	K715	Toxic liver disease with chronic active hepatitis
Liver disease	Mild	ICD-10	K716	Toxic liver disease with hepatitis, not elsewhere classified
Liver disease	Mild	ICD-10	K717	Toxic liver disease with fibrosis and cirrhosis of liver
Liver disease	Mild	ICD-10	K718	Toxic liver disease with other disorders of liver
Liver disease	Mild	ICD-10	K719	Toxic liver disease, unspecified
Liver disease	Mild	ICD-10	K73	Chronic hepatitis, not elsewhere classified
Liver disease	Mild	ICD-10	K74	Fibrosis and cirrhosis of liver
Liver disease	Mild	ICD-10	K750	Abscess of liver

Liver disease	Mild	ICD-10	K753	Granulomatous hepatitis, not elsewhere classified
Liver disease	Mild	ICD-10	K758	Other specified inflammatory liver diseases
Liver disease	Mild	ICD-10	K759	Inflammatory liver disease, unspecified
Liver disease	Mild	ICD-10	K760	Fatty (change of) liver, not elsewhere classified
Liver disease	Mild	ICD-10	K762	Central haemorrhagic necrosis of liver
Liver disease	Mild	ICD-10	K763	Infarction of liver
Liver disease	Mild	ICD-10	K764	Peliosis hepatis
Liver disease	Mild	ICD-10	K768	Other specified diseases of liver
Liver disease	Mild	ICD-10	K769	Liver disease, unspecified
Liver disease	Mild	ICD-10	K770	Liver disorders in infectious and parasitic diseases classified elsewhere
Liver disease	Mild	ICD-10	K778	Liver disorders in other diseases classified elsewhere
Liver disease	Mild	ICD-10	Q446	Cystic disease of liver
Liver disease	Mild	ICD-10	Z944	Liver transplant status
Liver disease	Mild	READ	A707	Chronic viral hepatitis
Liver disease	Mild	READ	B715.	Benign neoplasm of liver and biliary ducts
Liver disease	Mild	READ	B7150	Benign neoplasm of liver
Liver disease	Mild	READ	B7151	Benign neoplasm of intrahepatic bile ducts
Liver disease	Mild	READ	B7154	Benign neoplasm of hepatic duct
Liver disease	Mild	READ	B7158	Focal nodular hyperplasia of liver
Liver disease	Mild	READ	J6001	Acute hepatitis - noninfective
Liver disease	Mild	READ	J6011	Subacute hepatitis - noninfective
Liver disease	Mild	READ	J610.	Alcoholic fatty liver
Liver disease	Mild	READ	J611.	Acute alcoholic hepatitis
Liver disease	Mild	READ	J612	Alcoholic cirrhosis of liver
Liver disease	Mild	READ	J614	Chronic hepatitis
Liver disease	Mild	READ	J615	Cirrhosis - non alcoholic
Liver disease	Mild	READ	J616	Biliary cirrhosis

Liver disease	Mild	READ	J617	Alcoholic hepatitis	
Liver disease	Mild	READ	J61y	Other non-alcoholic chronic liver disease	
Liver disease	Mild	READ	J61z.	Chronic liver disease NOS	
Liver disease	Mild	READ	J620	Liver abscess - excluding amoebic liver abscess	
Liver disease	Mild	READ	J62y.	Other sequelae of chronic liver disease	
Liver disease	Mild	READ	J62z.	Liver abscess and chronic liver disease causing sequelae NOS	
Liver disease	Mild	READ	J631	Hepatitis in viral diseases EC	
Liver disease	Mild	READ	J632	Hepatitis in other infectious diseases EC	
Liver disease	Mild	READ	J633	Hepatitis unspecified	
Liver disease	Mild	READ	J634.	Hepatic infarction	
Liver disease	Mild	READ	J6353	Toxic liver disease with chronic persistent hepatitis	
Liver disease	Mild	READ	J6354	Toxic liver disease with chronic lobular hepatitis	
Liver disease	Mild	READ	J6355	Toxic liver disease with chronic active hepatitis	
Liver disease	Mild	READ	J6356	Toxic liver disease with fibrosis and cirrhosis of liver	
Liver disease	Mild	READ	J635X	Toxic liver disease, unspecified	
Liver disease	Mild	READ	J636.	Central haemorrhagic necrosis of liver	
Liver disease	Mild	READ	J638.	Peliosis hepatis	
Liver disease	Mild	READ	J639.	Hepatic granulomas in berylliosis	
Liver disease	Mild	READ	J63A.	Hepatic granulomas in sarcoidosis	
Liver disease	Mild	READ	J63X.	Granulomatous hepatitis, not elsewhere classified	
Liver disease	Mild	READ	J63y0	Hepatoptosis	
Liver disease	Mild	READ	J63y2	Liver cyst	
Liver disease	Mild	SNOMED CT	18027006	Transplantation of liver (procedure)	
Liver disease	Mild	SNOMED CT	27916005	Abscess of liver (disorder)	
Liver disease	Mild	SNOMED CT	41309000	Alcoholic liver damage (disorder)	235881000 (Alcoholic hepatic failure)
Liver disease	Mild	SNOMED CT	50325005	Alcoholic fatty liver (disorder)	

Liver disease	Mild	SNOMED CT	50701000	Hepatoptosis (disorder)	
Liver disease	Mild	SNOMED CT	58008004	Peliosis hepatis (disorder)	
Liver disease	Mild	SNOMED CT	62484002	Hepatic fibrosis (disorder)	
Liver disease	Mild	SNOMED CT	72925005	Congenital cystic disease of liver (disorder)	
Liver disease	Mild	SNOMED CT	76783007	Chronic hepatitis (disorder)	
Liver disease	Mild	SNOMED CT	85057007	Cyst of liver (disorder)	
Liver disease	Mild	SNOMED CT	86514004	Granulomatous hepatitis (disorder)	
Liver disease	Mild	SNOMED CT	87248009	Hepatic necrosis (disorder)	
Liver disease	Mild	SNOMED CT	92186001	Benign neoplasm of liver (disorder)	
Liver disease	Mild	SNOMED CT	128241005	Inflammatory disease of liver (disorder)	69800000 (Neonatal hepatitis) 276553003 (Idiopathic hepatitis in infancy) 276551001 (Perinatal hepatitis)
Liver disease	Mild	SNOMED CT	197321007	Steatosis of liver (disorder)	
Liver disease	Mild	SNOMED CT	197354009	Toxic liver disease (disorder)	197355005 (with cholestasis) 197356006 (with hepatic necrosis) 197358007 (with acute hepatitis)
Liver disease	Mild	SNOMED CT	235875008	Alcoholic hepatitis (disorder)	
Liver disease	Mild	SNOMED CT	240789006	Hepatosplenic schistosomiasis (disorder)	
Liver disease	Mild	SNOMED CT	278527001	Focal nodular hyperplasia of liver (disorder)	
Liver disease	Mild	SNOMED CT	442685003	Nonalcoholic steatohepatitis (disorder)	
Liver disease	Mild	CTV3	B715.	Benign neoplasm: [liver & biliary ducts] or [biliary system]	
Liver disease	Mild	CTV3	B7150	Benign tumour of liver	
Liver disease	Mild	CTV3	J601z	Subacute necrosis of liver NOS	
Liver disease	Mild	CTV3	J614.	Chronic hepatitis	

Liver disease	Mild	CTV3	J61y.	Other non-alcoholic chronic liver disease	
Liver disease	Mild	CTV3	J61y3	Portal fibrosis without cirrhosis	
Liver disease	Mild	CTV3	J61z.	Chronic liver disease NOS	
Liver disease	Mild	CTV3	J62..	Liver abscess and sequelae of chronic liver disease	
Liver disease	Mild	CTV3	J634.	Infarction of liver	
Liver disease	Mild	CTV3	J6353	Toxic liver disease with chronic persistent hepatitis	
Liver disease	Mild	CTV3	J6354	Toxic liver disease with chronic lobular hepatitis	
Liver disease	Mild	CTV3	J6355	Toxic liver disease with chronic active hepatitis	
Liver disease	Mild	CTV3	J6356	Toxic liver disease with fibrosis and cirrhosis of liver	
Liver disease	Mild	CTV3	J639.	Hepatic granulomas in berylliosis	
Liver disease	Mild	CTV3	J63y0	Hepatoptosis	
Liver disease	Mild	CTV3	Jyu71	[X]Other and unspecified cirrhosis of liver	
Liver disease	Mild	CTV3	Jyu74	[X]Liver disorders in infectious and parasitic diseases CE	
Liver disease	Mild	CTV3	Jyu75	[X]Liver disorders in other diseases classified elsewhere	
Liver disease	Mild	CTV3	Jyu76	[X]Toxic liver disease, unspecified	
Liver disease	Mild	CTV3	Jyu77	[X]Granulomatous hepatitis, not elsewhere classified	
Liver disease	Mild	CTV3	PB62.	(Congenital cystic liver disease) or (congenit hepatic cyst)	
Liver disease	Mild	CTV3	X306T	Inflammatory liver disease	X306U (Nonspecific reactive hepatitis)
Liver disease	Mild	CTV3	X306x	Peliosis hepatis	
Liver disease	Mild	CTV3	X3071	Alcoholic liver disease	X3073 (Alcoholic hepatic failure)
Liver disease	Mild	CTV3	X307L	Cirrhosis of liver	
Liver disease	Mild	CTV3	X307v	Fatty change of liver	
Liver disease	Mild	CTV3	Xa0lo	Focal nodular hyperplasia of liver	
Liver disease	Mild	CTV3	Xa8De	Liver necrosis	
Liver disease	Mild	CTV3	XaREa	Liver disease due to cystic fibrosis	
Liver disease	Mild	CTV3	XE1L1	Congenital cystic liver disease	
Liver disease	Mild	CTV3	XE2xC	Benign neoplasm of liver and biliary ducts	

Liver disease	Moderate/Severe	ICD-10	I85	Esophageal varices
Liver disease	Moderate/Severe	ICD-10	I85	Esophageal varices
Liver disease	Moderate/Severe	ICD-10	I864	Gastric varices
Liver disease	Moderate/Severe	ICD-10	I864	Gastric varices
Liver disease	Moderate/Severe	ICD-10	I982	Oesophageal varices without bleeding in diseases classified elsewhere
Liver disease	Moderate/Severe	ICD-10	I982	Oesophageal varices without bleeding in diseases classified elsewhere
Liver disease	Moderate/Severe	ICD-10	K704	Alcoholic hepatic failure
Liver disease	Moderate/Severe	ICD-10	K710	Toxic liver disease with cholestasis
Liver disease	Moderate/Severe	ICD-10	K711	Toxic liver disease with hepatic necrosis
Liver disease	Moderate/Severe	ICD-10	K712	Toxic liver disease with acute hepatitis
Liver disease	Moderate/Severe	ICD-10	K720	Acute and subacute hepatic failure
Liver disease	Moderate/Severe	ICD-10	K721	Chronic hepatic failure
Liver disease	Moderate/Severe	ICD-10	K729	Hepatic failure, unspecified
Liver disease	Moderate/Severe	ICD-10	K751	Phlebitis of portal vein
Liver disease	Moderate/Severe	ICD-10	K752	Nonspecific reactive hepatitis
Liver disease	Moderate/Severe	ICD-10	K754	Autoimmune hepatitis
Liver disease	Moderate/Severe	ICD-10	K761	Chronic passive congestion of liver
Liver disease	Moderate/Severe	ICD-10	K765	Hepatic veno-occlusive disease
Liver disease	Moderate/Severe	ICD-10	K766	Portal hypertension
Liver disease	Moderate/Severe	ICD-10	K767	Hepatorenal syndrome
Liver disease	Moderate/Severe	READ	G850.	Oesophageal varices with bleeding
Liver disease	Moderate/Severe	READ	G851.	Oesophageal varices without bleeding
Liver disease	Moderate/Severe	READ	G852	Oesophageal varices in diseases EC
Liver disease	Moderate/Severe	READ	G857.	Gastric varices
Liver disease	Moderate/Severe	READ	G858.	Oesophageal varices NOS
Liver disease	Moderate/Severe	READ	J6000	Acute hepatic failure
Liver disease	Moderate/Severe	READ	J6010	Subacute hepatic failure

Liver disease	Moderate/Severe	READ	J6130	Alcoholic hepatic failure
Liver disease	Moderate/Severe	READ	J621.	Portal pyaemia
Liver disease	Moderate/Severe	READ	J622.	Hepatic coma
Liver disease	Moderate/Severe	READ	J623.	Portal hypertension
Liver disease	Moderate/Severe	READ	J624.	Hepatorenal syndrome
Liver disease	Moderate/Severe	READ	J625.	[X] Hepatic failure
Liver disease	Moderate/Severe	READ	J630.	Chronic passive liver congestion
Liver disease	Moderate/Severe	READ	J6350	Toxic liver disease with cholestasis
Liver disease	Moderate/Severe	READ	J6351	Toxic liver disease with hepatic necrosis
Liver disease	Moderate/Severe	READ	J6352	Toxic liver disease with acute hepatitis
Liver disease	Moderate/Severe	READ	J6357	Acute hepatic failure due to drugs
Liver disease	Moderate/Severe	READ	J637.	Hepatic veno-occlusive disease
Liver disease	Moderate/Severe	READ	J63B.	Autoimmune hepatitis
Liver disease	Moderate/Severe	READ	J63y1	Nonspecific reactive hepatitis
Liver disease	Moderate/Severe	SNOMED CT	28670008	Esophageal varices (disorder)
Liver disease	Moderate/Severe	SNOMED CT	28670008	Esophageal varices (disorder)
Liver disease	Moderate/Severe	SNOMED CT	34736002	Chronic passive congestion of liver (disorder)
Liver disease	Moderate/Severe	SNOMED CT	34742003	Portal hypertension (disorder)
Liver disease	Moderate/Severe	SNOMED CT	59927004	Hepatic failure (disorder)
Liver disease	Moderate/Severe	SNOMED CT	65617004	Veno-occlusive disease of the liver (disorder)
Liver disease	Moderate/Severe	SNOMED CT	85514005	Phlebitis of portal vein (disorder)
Liver disease	Moderate/Severe	SNOMED CT	91109007	Gastric varices (disorder)
Liver disease	Moderate/Severe	SNOMED CT	91109007	Gastric varices (disorder)
Liver disease	Moderate/Severe	SNOMED CT	197355005	Toxic liver disease with cholestasis (disorder)
Liver disease	Moderate/Severe	SNOMED CT	197356006	Toxic liver disease with hepatic necrosis (disorder)
Liver disease	Moderate/Severe	SNOMED CT	197358007	Toxic liver disease with acute hepatitis (disorder)
Liver disease	Moderate/Severe	SNOMED CT	235858002	Nonspecific reactive hepatitis (disorder)
Liver disease	Moderate/Severe	SNOMED CT	408335007	Autoimmune hepatitis (disorder)

Liver disease	Moderate/Severe	CTV3	G857.	Gastric varices
Liver disease	Moderate/Severe	CTV3	J623.	Portal hypertension
Liver disease	Moderate/Severe	CTV3	J62y.	(Hepat failure (& [NOS]) or (oth sequelae chronic liver dis)
Liver disease	Moderate/Severe	CTV3	J630.	Chronic passive congestion of liver
Liver disease	Moderate/Severe	CTV3	J6350	Toxic liver disease with cholestasis
Liver disease	Moderate/Severe	CTV3	J6351	Toxic liver disease with hepatic necrosis
Liver disease	Moderate/Severe	CTV3	J6352	Toxic liver disease with acute hepatitis
Liver disease	Moderate/Severe	CTV3	X2063	Oesophageal varices
Liver disease	Moderate/Severe	CTV3	X306U	Nonspecific reactive hepatitis
Liver disease	Moderate/Severe	CTV3	X306y	Hepatic veno-occlusive disease
Liver disease	Moderate/Severe	CTV3	X3073	Alcoholic hepatic failure
Liver disease	Moderate/Severe	CTV3	X3076	Hepatic failure
Liver disease	Moderate/Severe	CTV3	X307J	Autoimmune liver disease
Liver disease	Moderate/Severe	CTV3	Xa8Df	Yellow atrophy of the liver
Liver disease	Moderate/Severe	CTV3	XE0dB	(Acute/subacute necrosis of liver) or (acute liver failure)

Any incomplete ICD-10 code (less than four characters) or READ v2 code (less than five characters) implies inclusion of all codes starting with the prefix code. Any CTV3 or SNOMED CT code implies inclusion of all children codes, excluding those in the Exclusion column.
