# Supplementary material for: COVID-19 in patients with hepatobiliary and pancreatic diseases: a single-centre cross-sectional study in East London
Source: BMJ Open. 2021 Apr 19;11(4):e045077. doi: 10.1136/bmjopen-2020-045077 (PMC8057071; doi:10.1136/bmjopen-2020-045077)
Supplement: Supplementary data [file bmjopen-2020-045077supp002.pdf]

Supplemental Table 2 Codelist for COVID-19 diagnosis

Group	Terminology system	Code	Code description
Confirmed	ICD-10	U071	COVID-19, virus identified
Confirmed	SNOMED CT	1240751000000100	Disease caused by 2019 novel coronavirus (disorder)
Confirmed	SNOMED CT	1240381000000105	2019 novel coronavirus (organism)
Confirmed	SNOMED CT	1240581000000104	2019 novel coronavirus detected (finding)
Suspected	ICD-10	U072	COVID-19, virus not identified
Suspected	SNOMED CT	1240761000000102	Suspected coronavirus disease 19 caused by severe acute respiratory syndrome coronavirus 2 (situation)
Negative	SNOMED CT	1240591000000102	2019 novel coronavirus not detected (finding)

Any SNOMED CT code implies inclusion of all children codes, excluding those in the Exclusion column.
