# Supplementary material for: COVID-19 in patients with hepatobiliary and pancreatic diseases: a single-centre cross-sectional study in East London
Source: BMJ Open. 2021 Apr 19;11(4):e045077. doi: 10.1136/bmjopen-2020-045077 (PMC8057071; doi:10.1136/bmjopen-2020-045077)
Supplement: Supplementary data [file bmjopen-2020-045077supp003.pdf]

Supplemental Table 3 Differences in demographic, comorbidity, lifestyle, and medication use characteristics between COVID-19 infected and non-COVID-19 groups, stratified by mortality status.

	non-COVID-19 (15214)		COVID-19 (N=217)	
	Survivor (N=14845)	Deceased (N=369)	Survivor (N=164)	Deceased (N=53)
Demographics				
**Gender**				
Female	8406 (98.1%)	164 (1.9%)	82 (84.5%)	15 (15.5%)
Male	6439 (96.9%)	205 (3.1%)	82 (68.3%)	38 (31.7%)
**Ethnic origin**				
White	6712 (97.1%)	202 (2.9%)	74 (78.7%)	20 (21.3%)
South Asian	4319 (98.6%)	62 (1.4%)	48 (72.7%)	18 (27.3%)
Black	1591 (97.3%)	44 (2.7%)	22 (61.1%)	14 (38.9%)
Other	1814 (97.8%)	41 (2.2%)	18 (94.7%)	1 (5.3%)
Unknown	409 (95.3%)	20 (4.7%)	2 (100.0%)	0 (0.0%)
**Age group**				
18-40	2796 (99.8%)	7 (0.2%)	21 (95.5%)	1 (4.5%)
41-50	2690 (99.1%)	24 (0.9%)	23 (92.0%)	2 (8.0%)
51-60	3362 (98.7%)	45 (1.3%)	31 (93.9%)	2 (6.1%)
61-70	2885 (97.6%)	72 (2.4%)	33 (80.5%)	8 (19.5%)
71-80	1889 (95.4%)	91 (4.6%)	25 (61.0%)	16 (39.0%)
80+	1223 (90.4%)	130 (9.6%)	31 (56.4%)	24 (43.6%)
**HPB cancer**				
No	14484 (98.0%)	295 (2.0%)	161 (75.9%)	51 (24.1%)
Yes	361 (83.0%)	74 (17.0%)	3 (60.0%)	2 (40.0%)
**Pancreatic disease**				
No	12040 (98.2%)	224 (1.8%)	129 (79.6%)	33 (20.4%)
Acute	1194 (98.6%)	17 (1.4%)	14 (73.7%)	5 (26.3%)
Chronic	1250 (95.9%)	54 (4.1%)	18 (58.1%)	13 (41.9%)
**Biliary disease**				
No	7448 (98.1%)	141 (1.9%)	99 (79.2%)	26 (20.8%)
Acute	717 (97.2%)	21 (2.8%)	5 (50.0%)	5 (50.0%)
Chronic	6319 (97.9%)	133 (2.1%)	57 (74.0%)	20 (26.0%)
**Liver disease**				
No	6642 (98.0%)	139 (2.0%)	54 (66.7%)	27 (33.3%)
Mild	6867 (98.3%)	118 (1.7%)	88 (82.2%)	19 (17.8%)
Moderate/Severe	975 (96.2%)	38 (3.8%)	19 (79.2%)	5 (20.8%)
Comorbidities				
**Diabetes**				
No	9186 (98.1%)	174 (1.9%)	65 (85.5%)	11 (14.5%)
Yes	5659 (96.7%)	195 (3.3%)	99 (70.2%)	42 (29.8%)
**Hypertension**				
No	5415 (99.3%)	40 (0.7%)	32 (97.0%)	1 (3.0%)
Yes	9430 (96.6%)	329 (3.4%)	132 (71.7%)	52 (28.3%)
**High cholesterol**				
No	6850 (98.0%)	137 (2.0%)	58 (82.9%)	12 (17.1%)
Yes	7995 (97.2%)	232 (2.8%)	106 (72.1%)	41 (27.9%)

**Cardiovascular**				
No	10790 (98.7%)	141 (1.3%)	80 (88.9%)	10 (11.1%)
Yes	4055 (94.7%)	228 (5.3%)	84 (66.1%)	43 (33.9%)
**Renal**				
No	11905 (98.2%)	215 (1.8%)	97 (86.6%)	15 (13.4%)
Yes	2940 (95.0%)	154 (5.0%)	67 (63.8%)	38 (36.2%)
**Respiratory**				
No	10416 (97.9%)	224 (2.1%)	84 (76.4%)	26 (23.6%)
Yes	4429 (96.8%)	145 (3.2%)	80 (74.8%)	27 (25.2%)
**Number of comorbidities**				
None	2397 (99.5%)	13 (0.5%)	8 (100.0%)	0 (0.0%)
1	2885 (98.7%)	39 (1.3%)	13 (100.0%)	0 (0.0%)
2	2992 (98.5%)	47 (1.5%)	26 (96.3%)	1 (3.7%)
3 or more	6571 (96.1%)	270 (3.9%)	117 (69.2%)	52 (30.8%)
Lifestyle factors				
**Smoker**				
Not available	422 (96.8%)	14 (3.2%)	2 (100.0%)	0 (0.0%)
Never	6301 (98.1%)	124 (1.9%)	67 (82.7%)	14 (17.3%)
Past	4960 (97.1%)	150 (2.9%)	72 (66.1%)	37 (33.9%)
Current	3162 (97.5%)	81 (2.5%)	23 (92.0%)	2 (8.0%)
**Drinker**				
Not available	2451 (97.8%)	54 (2.2%)	21 (77.8%)	6 (22.2%)
Never	3772 (97.8%)	85 (2.2%)	44 (78.6%)	12 (21.4%)
Past	2077 (96.8%)	68 (3.2%)	32 (72.7%)	12 (27.3%)
Current	6545 (97.6%)	162 (2.4%)	67 (74.4%)	23 (25.6%)
**Substance user**				
Not available	7537 (98.1%)	149 (1.9%)	77 (80.2%)	19 (19.8%)
Never	3565 (98.9%)	41 (1.1%)	24 (85.7%)	4 (14.3%)
Past	380 (94.3%)	23 (5.7%)	10 (83.3%)	2 (16.7%)
Current	3363 (95.6%)	156 (4.4%)	53 (65.4%)	28 (34.6%)
**Obese**				
Not available	394 (97.0%)	12 (3.0%)	0 (0.0%)	1 (100.0%)
Never	6556 (97.6%)	159 (2.4%)	65 (78.3%)	18 (21.7%)
Past	2101 (95.5%)	98 (4.5%)	33 (68.8%)	15 (31.2%)
Current	5794 (98.3%)	100 (1.7%)	66 (77.6%)	19 (22.4%)
Prescription medication use				
**ACE inhibitor**				
Non-user	11751 (79.2%)	273 (74.0%)	122 (74.4%)	33 (62.3%)
Past user	487 (3.3%)	31 (8.4%)	20 (12.2%)	8 (15.1%)
Current user	2607 (17.6%)	65 (17.6%)	22 (13.4%)	12 (22.6%)
**Angiotensin receptor blocker**				
Non-user	13204 (88.9%)	326 (88.3%)	137 (83.5%)	43 (81.1%)
Past user	207 (1.4%)	20 (5.4%)	3 (1.8%)	2 (3.8%)
Current user	1434 (9.7%)	23 (6.2%)	24 (14.6%)	8 (15.1%)
**Aldosterone antagonist**				
Non-user	14316 (96.4%)	335 (90.8%)	150 (91.5%)	47 (88.7%)
Past user	130 (0.9%)	7 (1.9%)	6 (3.7%)	3 (5.7%)
Current user	399 (2.7%)	27 (7.3%)	8 (4.9%)	3 (5.7%)

**β-blocker**				
Non-user	11930 (80.4%)	231 (62.6%)	106 (64.6%)	32 (60.4%)
Past user	384 (2.6%)	26 (7.0%)	9 (5.5%)	3 (5.7%)
Current user	2531 (17.0%)	112 (30.4%)	49 (29.9%)	18 (34.0%)
**Calcium channel blocker**				
Non-user	11456 (77.2%)	258 (69.9%)	116 (70.7%)	35 (66.0%)
Past user	543 (3.7%)	38 (10.3%)	14 (8.5%)	2 (3.8%)
Current user	2846 (19.2%)	73 (19.8%)	34 (20.7%)	16 (30.2%)
**α-agonist**				
Non-user	14766 (99.5%)	365 (98.9%)	163 (99.4%)	53 (100.0%)
Past user	20 (0.1%)	3 (0.8%)	0 (0.0%)	0 (0.0%)
Current user	59 (0.4%)	1 (0.3%)	1 (0.6%)	0 (0.0%)
**Thiazide**				
Non-user	14763 (99.4%)	368 (99.7%)	163 (99.4%)	53 (100.0%)
Past user	32 (0.2%)	0 (0.0%)	0 (0.0%)	0 (0.0%)
Current user	50 (0.3%)	1 (0.3%)	1 (0.6%)	0 (0.0%)
**Antiplatelet**				
Non-user	12268 (82.6%)	244 (66.1%)	112 (68.3%)	28 (52.8%)
Past user	415 (2.8%)	31 (8.4%)	8 (4.9%)	2 (3.8%)
Current user	2162 (14.6%)	94 (25.5%)	44 (26.8%)	23 (43.4%)
**Antiarrhythmic**				
Non-user	14109 (95.0%)	331 (89.7%)	147 (89.6%)	43 (81.1%)
Past user	148 (1.0%)	8 (2.2%)	6 (3.7%)	1 (1.9%)
Current user	588 (4.0%)	30 (8.1%)	11 (6.7%)	9 (17.0%)
**Anticoagulant**				
Non-user	14272 (96.1%)	341 (92.4%)	152 (92.7%)	47 (88.7%)
Past user	133 (0.9%)	11 (3.0%)	4 (2.4%)	1 (1.9%)
Current user	440 (3.0%)	17 (4.6%)	8 (4.9%)	5 (9.4%)
**Glucocorticoid**				
Non-user	10644 (71.7%)	234 (63.4%)	95 (57.9%)	19 (35.8%)
Past user	1230 (8.3%)	48 (13.0%)	16 (9.8%)	6 (11.3%)
Current user	2971 (20.0%)	87 (23.6%)	53 (32.3%)	28 (52.8%)
**β2-agonist**				
Non-user	13132 (88.5%)	311 (84.3%)	131 (79.9%)	33 (62.3%)
Past user	276 (1.9%)	10 (2.7%)	6 (3.7%)	2 (3.8%)
Current user	1437 (9.7%)	48 (13.0%)	27 (16.5%)	18 (34.0%)
**Muscarinic antagonist**				
Non-user	13235 (89.2%)	296 (80.2%)	129 (78.7%)	39 (73.6%)
Past user	291 (2.0%)	9 (2.4%)	5 (3.0%)	4 (7.5%)
Current user	1319 (8.9%)	64 (17.3%)	30 (18.3%)	10 (18.9%)
**NSAID**				
Non-user	13368 (90.1%)	335 (90.8%)	146 (89.0%)	43 (81.1%)
Past user	743 (5.0%)	13 (3.5%)	8 (4.9%)	2 (3.8%)
Current user	734 (4.9%)	21 (5.7%)	10 (6.1%)	8 (15.1%)
**Vitamin D**				
Non-user	12295 (82.8%)	247 (66.9%)	109 (66.5%)	26 (49.1%)
Past user	543 (3.7%)	30 (8.1%)	12 (7.3%)	6 (11.3%)
Current user	2007 (13.5%)	92 (24.9%)	43 (26.2%)	21 (39.6%)

**Proton pump inhibitor**				
Non-user	8162 (55.0%)	170 (46.1%)	62 (37.8%)	18 (34.0%)
Past user	1137 (7.7%)	30 (8.1%)	11 (6.7%)	3 (5.7%)
Current user	5546 (37.4%)	169 (45.8%)	91 (55.5%)	32 (60.4%)
**Statin**				
Non-user	8945 (60.3%)	183 (49.6%)	72 (43.9%)	11 (20.8%)
Past user	547 (3.7%)	45 (12.2%)	12 (7.3%)	3 (5.7%)
Current user	5353 (36.1%)	141 (38.2%)	80 (48.8%)	39 (73.6%)
**Immunosuppressant**				
Non-user	14368 (96.8%)	354 (95.9%)	156 (95.1%)	48 (90.6%)
Past user	196 (1.3%)	8 (2.2%)	3 (1.8%)	2 (3.8%)
Current user	281 (1.9%)	7 (1.9%)	5 (3.0%)	3 (5.7%)

Values are n (%), unless otherwise specified. *Percentages are calculated row-wise across each strata of the exposure variable, as opposed to column-wise across the outcome variable, to demonstrate the differences in mortality due to COVID-19.
