# Supplementary material for: COVID-19 in patients with hepatobiliary and pancreatic diseases: a single-centre cross-sectional study in East London
Source: BMJ Open. 2021 Apr 19;11(4):e045077. doi: 10.1136/bmjopen-2020-045077 (PMC8057071; doi:10.1136/bmjopen-2020-045077)
Supplement: Supplementary data [file bmjopen-2020-045077supp004.pdf]

Supplemental Table 4 Odds ratio estimates of COVID-19 for HPB patients with specific demographic, comorbidity, lifestyle and medication use characteristics.

	Crude OR (95% CI)	P value	Adjusted OR (95% CI)	P value	Adjusted OR (+all comorbidity) (95% CI)	P value
**Demographics**						
Gender (ref=Female)						
Male	1.49 (1.14 to 1.93)	0.003	1.56 (1.2 to 2.04)	0.002	1.39 (1.06 to 1.82)	0.04
Ethnicity (ref=White)						
South Asian	1.11 (0.81 to 1.51)	0.526	1.35 (0.98 to 1.85)	0.102	1.07 (0.77 to 1.48)	0.742
Black	1.74 (1.2 to 2.53)	0.009	2.04 (1.39 to 2.95)	<0.001	1.82 (1.23 to 2.66)	0.006
Other	0.73 (0.44 to 1.2)	0.265	0.87 (0.52 to 1.4)	0.654	0.88 (0.52 to 1.43)	0.716
Age group (ref=18-40)						
41-50	1.17 (0.66 to 2.05)	0.59	1.12 (0.64 to 1.98)	0.694	0.83 (0.47 to 1.49)	0.644
51-60	1.25 (0.74 to 2.12)	0.486	1.19 (0.7 to 2.05)	0.632	0.64 (0.36 to 1.13)	0.219
61-70	1.73 (1.04 to 2.89)	0.053	1.71 (1.03 to 2.9)	0.075	0.66 (0.38 to 1.19)	0.273
71-80	2.65 (1.59 to 4.41)	<0.001	2.71 (1.63 to 4.6)	<0.001	0.81 (0.45 to 1.48)	0.633
80+	5.13 (3.15 to 8.37)	<0.001	5.21 (3.23 to 8.7)	<0.001	1.31 (0.73 to 2.39)	0.53
**HPB disease (ref=No)**						
Cancer						
Yes	0.93 (0.41 to 2.1)	0.855	1.11 (0.46 to 2.69)	0.822	1.04 (0.43 to 2.54)	0.931
Pancreatic disease						
Acute	1.13 (0.7 to 1.83)	0.815	1.35 (0.82 to 2.22)	0.323	1.24 (0.75 to 2.04)	0.582
Chronic	1.72 (1.16 to 2.53)	0.013	1.89 (1.25 to 2.85)	0.007	1.57 (1.04 to 2.38)	0.084
Liver disease						
Mild	1.34 (1 to 1.78)	0.062	1.52 (1.07 to 2.15)	0.039	1.32 (0.93 to 1.88)	0.237
Moderate/Severe	2.04 (1.3 to 3.21)	0.004	2.2 (1.35 to 3.59)	0.006	1.84 (1.12 to 3.02)	0.046
Biliary disease						
Acute	0.89 (0.48 to 1.66)	0.715	1.1 (0.58 to 2.09)	0.822	1.04 (0.55 to 1.98)	0.931
Chronic	0.76 (0.57 to 1)	0.108	1.05 (0.75 to 1.46)	0.822	0.96 (0.69 to 1.35)	0.925
**Comorbidities (ref=No)**						
Diabetes	3.03 (2.31 to 4.02)	<0.001	2.47 (1.85 to 3.32)	<0.001	1.78 (1.3 to 2.42)	<0.001
Hypertension	3.27 (2.29 to 4.82)	<0.001	2.35 (1.59 to 3.48)	<0.001	1.38 (0.9 to 2.1)	0.226
Cholesterol	1.89 (1.43 to 2.53)	<0.001	1.47 (1.09 to 1.98)	0.019	0.94 (0.69 to 1.29)	0.71
Cardiovascular	3.52 (2.7 to 4.6)	<0.001	2.77 (2.07 to 3.71)	<0.001	1.84 (1.35 to 2.5)	<0.001
Renal	3.71 (2.85 to 4.84)	<0.001	2.93 (2.2 to 3.89)	<0.001	2.13 (1.59 to 2.86)	<0.001
Respiratory	2.25 (1.72 to 2.92)	<0.001	2.06 (1.58 to 2.69)	<0.001	1.77 (1.35 to 2.33)	<0.001
Number of comorbidities	1.66 (1.53 to 1.81)	<0.001	1.62 (1.46 to 1.79)	<0.001		
**Lifestyle factors (ref=Never)**						
Smoker						
Past	1.71 (1.28 to 2.27)	<0.001	1.46 (1.08 to 1.98)	0.03	1.18 (0.86 to 1.61)	0.476
Current	0.61 (0.39 to 0.95)	0.04	0.65 (0.41 to 1.04)	0.116	0.58 (0.36 to 0.93)	0.053

Drinker						
Past	1.46 (0.99 to 2.15)	0.115	1.26 (0.85 to 1.88)	0.283	1.11 (0.74 to 1.66)	0.756
Current	0.92 (0.66 to 1.28)	0.63	0.81 (0.56 to 1.16)	0.283	0.87 (0.61 to 1.24)	0.639
Substance user						
Past	4.01 (2.07 to 7.78)	<0.001	3.43 (1.74 to 6.75)	<0.001	2.18 (1.1 to 4.34)	0.053
Current	3 (1.97 to 4.59)	<0.001	2.63 (1.68 to 4.1)	<0.001	1.96 (1.25 to 3.07)	0.008
Obese						
Past	1.83 (1.29 to 2.6)	0.001	1.61 (1.13 to 2.3)	0.016	1.21 (0.84 to 1.74)	0.438
Current	1.19 (0.88 to 1.61)	0.248	1.26 (0.93 to 1.72)	0.187	1.01 (0.74 to 1.38)	0.973
**Prescription medication use (ref=Non-user)**						
ACE inhibitor						
Past user	4.04 (2.68 to 6.09)	<0.001	3 (1.97 to 4.57)	<0.001	1.88 (1.22 to 2.89)	0.007
Current user	1.03 (0.72 to 1.48)	0.855	0.8 (0.55 to 1.15)	0.25	0.56 (0.39 to 0.82)	0.005
Angiotensin receptor blocker						
Past user	1.59 (0.65 to 3.89)	0.314	1.18 (0.48 to 2.91)	0.722	0.67 (0.27 to 1.66)	0.524
Current user	1.63 (1.12 to 2.37)	0.016	1.27 (0.86 to 1.86)	0.293	0.91 (0.62 to 1.34)	0.681
Aldosterone agonist						
Past user	4.69 (2.36 to 9.35)	<0.001	3.74 (1.86 to 7.5)	<0.001	2.24 (1.1 to 4.56)	0.056
Current user	2.01 (1.12 to 3.63)	0.02	1.61 (0.89 to 2.91)	0.151	1 (0.54 to 1.82)	0.986
β-blocker						
Past user	2.45 (1.35 to 4.46)	0.003	1.95 (1.07 to 3.56)	0.045	1.24 (0.67 to 2.31)	0.666
Current user	2.19 (1.64 to 2.93)	<0.001	1.62 (1.19 to 2.19)	0.004	0.93 (0.67 to 1.29)	0.711
Calcium channel blocker						
Past user	2.17 (1.31 to 3.6)	0.004	1.5 (0.89 to 2.51)	0.162	0.99 (0.59 to 1.68)	0.982
Current user	1.3 (0.94 to 1.78)	0.111	0.9 (0.64 to 1.25)	0.533	0.68 (0.49 to 0.96)	0.056
α-agonist						
Past user	0 (0 to 2.41e+253)	0.97	0 (0 to 2.56e+248)	0.969	0 (0 to 1.73e+242)	0.968
Current user	1.12 (0.16 to 8.12)	0.97	0.77 (0.1 to 5.61)	0.895	0.6 (0.08 to 4.43)	0.771
Thiazide						
Past user	0 (0 to 1.16e+214)	0.965	0 (0 to 8.76e+208)	0.971	0 (0 to 9.71e+205)	0.962
Current user	1.32 (0.18 to 9.58)	0.965	1.04 (0.14 to 7.59)	0.971	0.83 (0.11 to 6.17)	0.919
Antiplatelet						
Past user	1.91 (1 to 3.65)	0.05	1.41 (0.73 to 2.71)	0.345	0.79 (0.4 to 1.54)	0.661
Current user	2.6 (1.95 to 3.48)	<0.001	1.84 (1.35 to 2.51)	<0.001	0.95 (0.67 to 1.33)	0.754
Antiarrhythmic						
Past user	3.26 (1.51 to 7.03)	0.003	2.42 (1.11 to 5.27)	0.039	1.74 (0.79 to 3.83)	0.282
Current user	2.35 (1.47 to 3.75)	<0.001	1.85 (1.15 to 2.97)	0.021	1.19 (0.73 to 1.93)	0.602
Anticoagulant						
Past user	2.44 (0.99 to 6.01)	0.053	1.96 (0.79 to 4.86)	0.165	1.39 (0.55 to 3.48)	0.661
Current user	2 (1.13 to 3.53)	0.025	1.54 (0.86 to 2.73)	0.165	1 (0.56 to 1.8)	0.989
Glucocorticoid						
Past user	1.53 (0.97 to 2.43)	0.067	1.39 (0.88 to 2.2)	0.242	1.09 (0.68 to 1.74)	0.774
Current user	2.39 (1.8 to 3.17)	<0.001	2.07 (1.55 to 2.77)	<0.001	1.38 (1 to 1.92)	0.101

β2-agonist						
Past user	2.19 (1.07 to 4.48)	0.033	2.08 (1.01 to 4.27)	0.071	1.36 (0.64 to 2.87)	0.562
Current user	2.42 (1.74 to 3.37)	<0.001	2.07 (1.48 to 2.9)	<0.001	1.32 (0.89 to 1.96)	0.281
Muscarinic antagonist						
Past user	2.58 (1.35 to 4.92)	0.004	2.2 (1.14 to 4.22)	0.027	1.67 (0.86 to 3.24)	0.241
Current user	2.29 (1.62 to 3.24)	<0.001	1.85 (1.3 to 2.64)	0.001	1.25 (0.86 to 1.81)	0.365
NSAID						
Past user	0.92 (0.48 to 1.74)	0.787	0.81 (0.42 to 1.54)	0.517	0.79 (0.42 to 1.51)	0.598
Current user	1.65 (1.01 to 2.69)	0.066	1.56 (0.95 to 2.55)	0.139	1.57 (0.96 to 2.59)	0.14
Vitamin D						
Past user	2.83 (1.72 to 4.66)	<0.001	2.49 (1.5 to 4.13)	<0.001	1.84 (1.1 to 3.06)	0.033
Current user	2.97 (2.21 to 3.97)	<0.001	2.5 (1.84 to 3.4)	<0.001	1.79 (1.31 to 2.45)	<0.001
Proton pump inhibitor						
Past user	1.26 (0.72 to 2.19)	0.412	1.15 (0.66 to 2.01)	0.619	0.95 (0.54 to 1.66)	0.858
Current user	2.16 (1.64 to 2.85)	<0.001	1.78 (1.34 to 2.38)	<0.001	1.18 (0.87 to 1.59)	0.447
Statin						
Past user	2.87 (1.67 to 4.92)	<0.001	2.1 (1.21 to 3.65)	0.013	1.24 (0.69 to 2.21)	0.643
Current user	2.4 (1.82 to 3.16)	<0.001	1.77 (1.3 to 2.4)	<0.001	0.94 (0.66 to 1.33)	0.758
Immunosuppressant						
Past user	1.69 (0.69 to 4.16)	0.25	1.42 (0.58 to 3.51)	0.448	1.28 (0.52 to 3.18)	0.686
Current user	1.92 (0.94 to 3.93)	0.111	1.7 (0.83 to 3.5)	0.191	1.45 (0.7 to 2.99)	0.481

	Adjusted OR (+Diabetes) (95% CI)	P value	Adjusted OR (+Hypertension) (95% CI)	P value	Adjusted OR (+Cholesterol) (95% CI)	P value
**Demographics**						
Gender (ref=Female)						
Male	1.48 (1.14 to 1.94)	0.009	1.52 (1.16 to 1.99)	0.005	1.55 (1.19 to 2.02)	0.004
Ethnicity (ref=White)						
South Asian	1.04 (0.75 to 1.44)	0.868	1.3 (0.94 to 1.78)	0.18	1.29 (0.93 to 1.77)	0.209
Black	1.76 (1.19 to 2.56)	0.009	1.95 (1.32 to 2.82)	0.002	2.06 (1.4 to 2.99)	<0.001
Other	0.78 (0.46 to 1.26)	0.503	0.88 (0.52 to 1.42)	0.75	0.87 (0.51 to 1.4)	0.705
Age group (ref=18-40)						
41-50	0.95 (0.54 to 1.69)	0.868	0.97 (0.55 to 1.72)	0.914	1.02 (0.58 to 1.83)	0.933
51-60	0.9 (0.52 to 1.56)	0.83	0.92 (0.54 to 1.61)	0.835	1.04 (0.61 to 1.82)	0.933
61-70	1.16 (0.69 to 1.99)	0.789	1.2 (0.71 to 2.09)	0.664	1.45 (0.86 to 2.5)	0.235
71-80	1.75 (1.03 to 3.03)	0.08	1.79 (1.05 to 3.14)	0.071	2.25 (1.33 to 3.92)	0.008
80+	3.39 (2.06 to 5.76)	<0.001	3.36 (2.01 to 5.8)	<0.001	4.32 (2.61 to 7.39)	<0.001
**HPB disease (ref=No)**						
Cancer						
Yes	1 (0.41 to 2.43)	0.994	1.04 (0.43 to 2.54)	0.926	1.11 (0.46 to 2.71)	0.836

Pancreatic disease						
Acute	1.28 (0.78 to 2.1)	0.453	1.29 (0.78 to 2.12)	0.429	1.32 (0.8 to 2.17)	0.369
Chronic	1.69 (1.11 to 2.56)	0.033	1.77 (1.17 to 2.66)	0.017	1.84 (1.22 to 2.78)	0.011
Liver disease						
Mild	1.4 (0.99 to 1.98)	0.112	1.44 (1.01 to 2.04)	0.08	1.47 (1.04 to 2.09)	0.058
Moderate/Severe	2.04 (1.25 to 3.33)	0.013	2.09 (1.28 to 3.42)	0.01	2.16 (1.33 to 3.53)	0.008
Biliary disease						
Acute	1.08 (0.57 to 2.05)	0.941	1.07 (0.56 to 2.03)	0.926	1.07 (0.57 to 2.04)	0.836
Chronic	1.03 (0.74 to 1.44)	0.941	1.02 (0.73 to 1.42)	0.926	1.04 (0.74 to 1.45)	0.836
**Comorbidities (ref=No)**						
Diabetes	2.47 (1.85 to 3.32)	<0.001	2.19 (1.62 to 2.96)	<0.001	2.37 (1.76 to 3.2)	<0.001
Hypertension	1.87 (1.25 to 2.8)	0.005	2.35 (1.59 to 3.48)	<0.001	2.21 (1.48 to 3.29)	<0.001
Cholesterol	1.21 (0.9 to 1.64)	0.275	1.29 (0.96 to 1.75)	0.142	1.47 (1.09 to 1.98)	0.019
Cardiovascular	2.38 (1.77 to 3.2)	<0.001	2.45 (1.82 to 3.29)	<0.001	2.66 (1.98 to 3.58)	<0.001
Renal	2.55 (1.91 to 3.4)	<0.001	2.64 (1.98 to 3.52)	<0.001	2.83 (2.12 to 3.77)	<0.001
Respiratory	1.95 (1.49 to 2.54)	<0.001	1.98 (1.51 to 2.58)	<0.001	2.02 (1.54 to 2.64)	<0.001
**Lifestyle factors (ref=Never)**						
Smoker						
Past	1.39 (1.02 to 1.89)	0.064	1.42 (1.05 to 1.93)	0.044	1.42 (1.05 to 1.93)	0.052
Current	0.66 (0.41 to 1.04)	0.118	0.64 (0.4 to 1.02)	0.094	0.65 (0.41 to 1.02)	0.099
Drinker						
Past	1.19 (0.8 to 1.78)	0.446	1.21 (0.81 to 1.81)	0.394	1.23 (0.82 to 1.84)	0.38
Current	0.82 (0.57 to 1.17)	0.388	0.8 (0.56 to 1.14)	0.345	0.8 (0.56 to 1.14)	0.335
Substance user						
Past	3.08 (1.56 to 6.08)	0.002	3.14 (1.6 to 6.19)	0.002	3.29 (1.67 to 6.48)	0.001
Current	2.5 (1.6 to 3.89)	<0.001	2.47 (1.58 to 3.85)	<0.001	2.56 (1.64 to 3.99)	<0.001
Obese						
Past	1.38 (0.96 to 1.97)	0.148	1.47 (1.03 to 2.1)	0.061	1.56 (1.09 to 2.22)	0.033
Current	1.1 (0.81 to 1.5)	0.6	1.16 (0.85 to 1.57)	0.391	1.23 (0.9 to 1.67)	0.233
**Prescription medication use (ref=Non-user)**						
ACE inhibitor						
Past user	2.52 (1.65 to 3.85)	<0.001	2.54 (1.66 to 3.87)	<0.001	2.86 (1.88 to 4.36)	<0.001
Current user	0.64 (0.44 to 0.93)	0.029	0.67 (0.46 to 0.97)	0.047	0.75 (0.52 to 1.09)	0.19
Angiotensin receptor blocker						
Past user	0.98 (0.4 to 2.43)	0.981	1.04 (0.42 to 2.57)	0.928	1.13 (0.46 to 2.78)	0.799
Current user	1.1 (0.75 to 1.62)	0.784	1.1 (0.75 to 1.61)	0.707	1.2 (0.82 to 1.77)	0.427
Aldosterone agonist						
Past user	3.47 (1.73 to 6.99)	0.001	3.5 (1.74 to 7.02)	0.001	3.62 (1.8 to 7.27)	<0.001
Current user	1.49 (0.82 to 2.71)	0.232	1.49 (0.82 to 2.69)	0.217	1.56 (0.86 to 2.83)	0.199
β-blocker						
Past user	1.79 (0.98 to 3.28)	0.083	1.78 (0.97 to 3.25)	0.087	1.87 (1.02 to 3.42)	0.06
Current user	1.4 (1.04 to 1.9)	0.048	1.44 (1.06 to 1.95)	0.03	1.54 (1.14 to 2.09)	0.013

Calcium channel blocker						
Past user	1.33 (0.79 to 2.24)	0.309	1.24 (0.74 to 2.09)	0.459	1.42 (0.84 to 2.38)	0.236
Current user	0.79 (0.57 to 1.11)	0.245	0.73 (0.52 to 1.03)	0.119	0.86 (0.62 to 1.21)	0.437
α-agonist						
Past user	0 (0 to 1.19e+246)	0.978	0 (0 to 2.77e+247)	0.969	0 (0 to 2.45e+248)	0.969
Current user	0.68 (0.09 to 4.93)	0.874	0.72 (0.1 to 5.26)	0.831	0.73 (0.1 to 5.32)	0.838
Thiazide						
Past user	0 (0 to 3.39e+207)	0.986	0 (0 to 8.99e+209)	0.963	0 (0 to 1.01e+209)	0.989
Current user	0.91 (0.12 to 6.66)	0.986	0.92 (0.13 to 6.76)	0.963	1.01 (0.14 to 7.43)	0.989
Antiplatelet						
Past user	1.2 (0.62 to 2.32)	0.655	1.27 (0.66 to 2.44)	0.48	1.34 (0.7 to 2.59)	0.423
Current user	1.54 (1.13 to 2.11)	0.013	1.64 (1.2 to 2.23)	0.004	1.73 (1.27 to 2.38)	0.002
Antiarrhythmic						
Past user	2.4 (1.1 to 5.24)	0.04	2.24 (1.03 to 4.87)	0.06	2.32 (1.07 to 5.07)	0.048
Current user	1.71 (1.06 to 2.75)	0.04	1.72 (1.07 to 2.77)	0.041	1.78 (1.11 to 2.87)	0.031
Anticoagulant						
Past user	1.91 (0.77 to 4.75)	0.233	1.83 (0.74 to 4.54)	0.237	1.89 (0.76 to 4.69)	0.204
Current user	1.44 (0.81 to 2.57)	0.269	1.44 (0.81 to 2.57)	0.237	1.48 (0.83 to 2.63)	0.204
Glucocorticoid						
Past user	1.29 (0.81 to 2.05)	0.313	1.33 (0.84 to 2.11)	0.304	1.36 (0.86 to 2.16)	0.276
Current user	1.89 (1.41 to 2.52)	<0.001	1.96 (1.46 to 2.61)	<0.001	2 (1.5 to 2.68)	<0.001
β2-agonist						
Past user	1.93 (0.94 to 3.98)	0.107	1.94 (0.94 to 4)	0.103	2.04 (0.99 to 4.2)	0.076
Current user	1.92 (1.37 to 2.69)	<0.001	1.97 (1.41 to 2.76)	<0.001	2 (1.43 to 2.81)	<0.001
Muscarinic antagonist						
Past user	2.05 (1.06 to 3.94)	0.046	2.11 (1.1 to 4.06)	0.036	2.13 (1.11 to 4.09)	0.034
Current user	1.74 (1.22 to 2.47)	0.004	1.75 (1.23 to 2.48)	0.004	1.8 (1.27 to 2.57)	0.003
NSAID						
Past user	0.81 (0.42 to 1.53)	0.569	0.78 (0.41 to 1.49)	0.506	0.8 (0.42 to 1.52)	0.492
Current user	1.53 (0.93 to 2.5)	0.154	1.51 (0.92 to 2.46)	0.173	1.53 (0.94 to 2.51)	0.148
Vitamin D						
Past user	2.27 (1.37 to 3.76)	0.003	2.35 (1.42 to 3.9)	0.002	2.4 (1.45 to 3.99)	0.001
Current user	2.29 (1.68 to 3.11)	<0.001	2.34 (1.72 to 3.18)	<0.001	2.42 (1.78 to 3.29)	<0.001
Proton pump inhibitor						
Past user	1.1 (0.63 to 1.91)	0.754	1.11 (0.64 to 1.93)	0.715	1.11 (0.64 to 1.94)	0.704
Current user	1.55 (1.16 to 2.07)	0.007	1.62 (1.21 to 2.17)	0.002	1.69 (1.26 to 2.27)	0.001
Statin						
Past user	1.62 (0.93 to 2.85)	0.152	1.84 (1.06 to 3.21)	0.045	1.95 (1.1 to 3.45)	0.036
Current user	1.3 (0.94 to 1.8)	0.16	1.49 (1.09 to 2.05)	0.02	1.64 (1.17 to 2.29)	0.01
Immunosuppressant						
Past user	1.4 (0.57 to 3.46)	0.519	1.36 (0.55 to 3.35)	0.507	1.38 (0.56 to 3.4)	0.489
Current user	1.59 (0.77 to 3.27)	0.297	1.65 (0.8 to 3.39)	0.242	1.69 (0.82 to 3.47)	0.231

	Adjusted OR (+Cardiovascular) (95% CI)	P value	Adjusted OR (+Renal) (95% CI)	P value	Adjusted OR (+Respiratory) (95% CI)	P value
**Demographics**						
Gender (ref=Female)						
Male	1.39 (1.06 to 1.82)	0.042	1.53 (1.17 to 2)	0.004	1.57 (1.21 to 2.06)	0.002
Ethnicity (ref=White)						
South Asian	1.3 (0.94 to 1.78)	0.186	1.28 (0.93 to 1.76)	0.22	1.37 (1 to 1.88)	0.087
Black	2.01 (1.36 to 2.91)	<0.001	1.93 (1.31 to 2.8)	0.002	2.17 (1.47 to 3.15)	<0.001
Other	0.89 (0.53 to 1.44)	0.792	0.86 (0.51 to 1.39)	0.675	0.93 (0.55 to 1.5)	0.779
Age group (ref=18-40)						
41-50	1.03 (0.58 to 1.82)	0.925	1.04 (0.59 to 1.84)	0.944	1.11 (0.63 to 1.97)	0.779
51-60	0.96 (0.56 to 1.66)	0.925	1.02 (0.6 to 1.76)	0.944	1.12 (0.66 to 1.94)	0.779
61-70	1.18 (0.7 to 2.05)	0.712	1.29 (0.77 to 2.21)	0.466	1.55 (0.94 to 2.64)	0.141
71-80	1.66 (0.97 to 2.9)	0.139	1.75 (1.03 to 3.04)	0.086	2.37 (1.43 to 4.05)	0.002
80+	2.84 (1.68 to 4.93)	<0.001	2.88 (1.71 to 4.99)	<0.001	4.58 (2.83 to 7.66)	<0.001
**HPB disease (ref=No)**						
Cancer						
Yes	1.13 (0.46 to 2.74)	0.888	1.11 (0.45 to 2.7)	0.902	1.1 (0.45 to 2.68)	0.872
Pancreatic disease						
Acute	1.33 (0.81 to 2.18)	0.356	1.33 (0.81 to 2.18)	0.363	1.33 (0.81 to 2.19)	0.348
Chronic	1.77 (1.17 to 2.67)	0.017	1.78 (1.18 to 2.69)	0.015	1.81 (1.2 to 2.74)	0.011
Liver disease						
Mild	1.46 (1.03 to 2.08)	0.07	1.48 (1.04 to 2.09)	0.054	1.46 (1.03 to 2.07)	0.067
Moderate/Severe	2.08 (1.27 to 3.41)	0.011	2.07 (1.26 to 3.39)	0.012	2.12 (1.3 to 3.46)	0.008
Biliary disease						
Acute	1.07 (0.57 to 2.04)	0.888	1.07 (0.56 to 2.03)	0.902	1.11 (0.59 to 2.11)	0.86
Chronic	1.01 (0.73 to 1.42)	0.935	1.01 (0.72 to 1.4)	0.975	1.03 (0.74 to 1.43)	0.872
**Comorbidities (ref=No)**						
Diabetes	2.1 (1.56 to 2.83)	<0.001	2.12 (1.57 to 2.86)	<0.001	2.35 (1.75 to 3.15)	<0.001
Hypertension	1.83 (1.22 to 2.75)	0.008	1.93 (1.3 to 2.89)	0.004	2.21 (1.49 to 3.27)	<0.001
Cholesterol	1.24 (0.91 to 1.68)	0.22	1.28 (0.95 to 1.74)	0.155	1.39 (1.03 to 1.88)	0.044
Cardiovascular	2.77 (2.07 to 3.71)	<0.001	2.26 (1.67 to 3.05)	<0.001	2.57 (1.92 to 3.45)	<0.001
Renal	2.4 (1.79 to 3.22)	<0.001	2.93 (2.2 to 3.89)	<0.001	2.81 (2.11 to 3.74)	<0.001
Respiratory	1.86 (1.42 to 2.44)	<0.001	1.96 (1.5 to 2.56)	<0.001	2.06 (1.58 to 2.69)	<0.001
**Lifestyle factors (ref=Never)**						
Smoker						
Past	1.34 (0.99 to 1.82)	0.096	1.37 (1.01 to 1.86)	0.08	1.31 (0.96 to 1.78)	0.137
Current	0.62 (0.39 to 0.98)	0.092	0.68 (0.43 to 1.08)	0.158	0.56 (0.36 to 0.9)	0.029
Drinker						
Past	1.23 (0.82 to 1.84)	0.432	1.22 (0.82 to 1.82)	0.405	1.2 (0.8 to 1.8)	0.407
Current	0.86 (0.6 to 1.24)	0.471	0.84 (0.58 to 1.2)	0.405	0.8 (0.56 to 1.14)	0.305

Substance user						
Past	2.74 (1.38 to 5.42)	0.007	2.91 (1.47 to 5.75)	0.004	3.02 (1.53 to 5.96)	0.003
Current	2.21 (1.41 to 3.46)	0.001	2.41 (1.54 to 3.76)	<0.001	2.38 (1.52 to 3.72)	<0.001
Obese						
Past	1.49 (1.05 to 2.13)	0.05	1.46 (1.02 to 2.08)	0.071	1.53 (1.07 to 2.19)	0.034
Current	1.2 (0.88 to 1.63)	0.264	1.21 (0.89 to 1.64)	0.261	1.21 (0.89 to 1.65)	0.264
**Prescription medication use (ref=Non-user)**						
ACE inhibitor						
Past user	2.38 (1.56 to 3.64)	<0.001	2.43 (1.59 to 3.72)	<0.001	2.88 (1.89 to 4.4)	<0.001
Current user	0.67 (0.46 to 0.97)	0.055	0.72 (0.5 to 1.03)	0.105	0.78 (0.54 to 1.13)	0.215
Angiotensin receptor blocker						
Past user	0.93 (0.38 to 2.31)	0.884	0.87 (0.35 to 2.15)	0.758	1.1 (0.45 to 2.73)	0.834
Current user	1.1 (0.75 to 1.62)	0.689	1.09 (0.74 to 1.6)	0.742	1.21 (0.83 to 1.78)	0.409
Aldosterone agonist						
Past user	2.76 (1.37 to 5.56)	0.009	2.9 (1.44 to 5.86)	0.006	3.56 (1.77 to 7.15)	<0.001
Current user	1.17 (0.64 to 2.13)	0.612	1.29 (0.71 to 2.34)	0.455	1.5 (0.82 to 2.72)	0.228
β-blocker						
Past user	1.4 (0.76 to 2.58)	0.353	1.6 (0.87 to 2.94)	0.184	1.99 (1.09 to 3.63)	0.037
Current user	1.09 (0.79 to 1.5)	0.614	1.32 (0.97 to 1.79)	0.132	1.6 (1.18 to 2.16)	0.005
Calcium channel blocker						
Past user	1.24 (0.74 to 2.09)	0.466	1.25 (0.74 to 2.1)	0.452	1.43 (0.85 to 2.41)	0.225
Current user	0.83 (0.6 to 1.16)	0.346	0.8 (0.57 to 1.11)	0.229	0.9 (0.64 to 1.25)	0.579
α-agonist						
Past user	0 (0 to 1.39e+247)	0.969	0 (0 to 4.77e+244)	0.968	0 (0 to 1.01e+246)	0.969
Current user	0.69 (0.09 to 5.08)	0.799	0.66 (0.09 to 4.86)	0.762	0.81 (0.11 to 5.9)	0.926
Thiazide						
Past user	0 (0 to 1.82e+207)	0.962	0 (0 to 4.41e+206)	0.962	0 (0 to 1.47e+209)	0.986
Current user	1.1 (0.15 to 8.06)	0.962	0.9 (0.12 to 6.63)	0.962	1.02 (0.14 to 7.47)	0.986
Antiplatelet						
Past user	0.9 (0.46 to 1.75)	0.751	1.18 (0.61 to 2.27)	0.632	1.34 (0.69 to 2.58)	0.431
Current user	1.11 (0.79 to 1.56)	0.624	1.53 (1.12 to 2.1)	0.015	1.7 (1.24 to 2.32)	0.002
Antiarrhythmic						
Past user	1.87 (0.85 to 4.08)	0.177	2.02 (0.92 to 4.42)	0.13	2.28 (1.04 to 4.98)	0.055
Current user	1.34 (0.83 to 2.18)	0.256	1.5 (0.93 to 2.42)	0.139	1.75 (1.09 to 2.82)	0.036
Anticoagulant						
Past user	1.49 (0.6 to 3.7)	0.494	1.69 (0.68 to 4.2)	0.327	1.9 (0.76 to 4.72)	0.23
Current user	1.15 (0.64 to 2.05)	0.647	1.25 (0.7 to 2.23)	0.498	1.45 (0.81 to 2.58)	0.23
Glucocorticoid						
Past user	1.29 (0.81 to 2.05)	0.317	1.32 (0.83 to 2.1)	0.299	1.21 (0.76 to 1.93)	0.475
Current user	1.88 (1.41 to 2.52)	<0.001	1.93 (1.44 to 2.58)	<0.001	1.57 (1.13 to 2.19)	0.011
β2-agonist						
Past user	1.84 (0.89 to 3.8)	0.141	2 (0.97 to 4.12)	0.089	1.42 (0.68 to 3.01)	0.393
Current user	1.84 (1.31 to 2.59)	0.001	2.02 (1.44 to 2.84)	<0.001	1.38 (0.93 to 2.05)	0.153

Muscarinic antagonist						
Past user	1.95 (1.01 to 3.76)	0.066	2.15 (1.11 to 4.14)	0.032	1.82 (0.94 to 3.52)	0.101
Current user	1.61 (1.13 to 2.3)	0.017	1.74 (1.22 to 2.48)	0.005	1.39 (0.96 to 2.03)	0.101
NSAID						
Past user	0.81 (0.42 to 1.54)	0.567	0.83 (0.43 to 1.58)	0.565	0.77 (0.4 to 1.47)	0.477
Current user	1.55 (0.95 to 2.55)	0.136	1.64 (1 to 2.69)	0.085	1.5 (0.91 to 2.45)	0.156
Vitamin D						
Past user	2.22 (1.34 to 3.69)	0.004	2.15 (1.3 to 3.58)	0.005	2.32 (1.4 to 3.85)	0.002
Current user	2.18 (1.6 to 2.97)	<0.001	2.13 (1.56 to 2.91)	<0.001	2.3 (1.69 to 3.13)	<0.001
Proton pump inhibitor						
Past user	1.08 (0.62 to 1.88)	0.797	1.05 (0.6 to 1.84)	0.855	1.1 (0.63 to 1.92)	0.738
Current user	1.46 (1.09 to 1.97)	0.023	1.58 (1.18 to 2.12)	0.005	1.61 (1.2 to 2.16)	0.002
Statin						
Past user	1.68 (0.96 to 2.95)	0.098	1.75 (1 to 3.06)	0.072	1.99 (1.14 to 3.46)	0.022
Current user	1.35 (0.98 to 1.86)	0.098	1.46 (1.07 to 2)	0.03	1.65 (1.21 to 2.25)	0.003
Immunosuppressant						
Past user	1.37 (0.55 to 3.39)	0.549	1.33 (0.54 to 3.28)	0.542	1.39 (0.56 to 3.43)	0.531
Current user	1.69 (0.82 to 3.48)	0.222	1.56 (0.76 to 3.22)	0.286	1.59 (0.77 to 3.28)	0.257

Odds ratios (ORs), except the crude ones, are mutually adjusted for gender, ethnicity, and age group, and also for additional conditions when mentioned inside the parenthesis. Dichotomous age groups (over and under 60) are used for controlling for all categories except demographics. All P values presented, except for the crude odds ratios, are Benjamini-Hochberg corrected.
