# Supplementary material for: COVID-19 in patients with hepatobiliary and pancreatic diseases: a single-centre cross-sectional study in East London
Source: BMJ Open. 2021 Apr 19;11(4):e045077. doi: 10.1136/bmjopen-2020-045077 (PMC8057071; doi:10.1136/bmjopen-2020-045077)
Supplement: Supplementary data [file bmjopen-2020-045077supp005.pdf]

Supplemental Table 5 Association between HPB disease and COVID-19 risk factors according to HPB disease subtypes

	Pancreatic disease			
	No (N=12434)	Acute (N=1230)	Chronic (N=1335)	
	OR (95% CI)	OR (95% CI)	OR (95% CI)	P-het
**Demographics**				
Gender (ref=Female)				0.33
Male	1.58 (1.15 to 2.15)	0.74 (0.29 to 1.88)	1.7 (0.79 to 3.63)	
Ethnicity (ref=White)				0.83
South Asian	1.36 (0.94 to 1.96)	1.82 (0.59 to 5.61)	1.29 (0.51 to 3.29)	
Black	2.07 (1.34 to 3.2)	4.14 (1.1 to 15.61)	2.02 (0.76 to 5.37)	
Other	0.92 (0.53 to 1.62)	1.37 (0.26 to 7.07)	0.74 (0.17 to 3.3)	
Age group (ref=18-40)				0.87
41-50	0.98 (0.5 to 1.9)	0.68 (0.11 to 4.27)	1.86 (0.45 to 7.59)	
51-60	1.26 (0.69 to 2.3)	0.79 (0.13 to 4.91)	0.62 (0.12 to 3.16)	
61-70	1.51 (0.83 to 2.76)	1.33 (0.26 to 6.85)	1.71 (0.42 to 7.02)	
71-80	2.76 (1.52 to 5.02)	3.14 (0.68 to 14.57)	2.02 (0.47 to 8.65)	
80+	5.9 (3.32 to 10.48)	6.07 (1.38 to 26.64)	4.63 (1.18 to 18.13)	
**HPB disease (ref=No)**				
Liver disease				0.15
Mild	1.8 (1.14 to 2.84)	2.27 (0.8 to 6.41)	1.65 (0.73 to 3.73)	
Moderate/Severe	2.82 (1.56 to 5.12)	4.9 (0.97 to 24.82)	1.54 (0.34 to 6.93)	
Biliary disease				0.04
Acute	1.05 (0.49 to 2.29)	0 (0 to Inf)	2.15 (0.61 to 7.64)	
Chronic	1.17 (0.76 to 1.78)	0.61 (0.22 to 1.66)	0.84 (0.33 to 2.15)	
**Comorbidities (ref=No)**				
Diabetes	2.34 (1.67 to 3.27)	3.2 (1.07 to 9.57)	3.39 (1.4 to 8.24)	0.18
Hypertension	2.22 (1.43 to 3.44)	2.67 (0.58 to 12.35)	1.91 (0.62 to 5.88)	0.65
Cholesterol	1.51 (1.07 to 2.13)	1.1 (0.38 to 3.18)	1.2 (0.53 to 2.68)	0.91
Cardiovascular	2.55 (1.83 to 3.55)	3.9 (1.36 to 11.21)	3.07 (1.35 to 6.98)	0.91
Renal	3 (2.16 to 4.16)	2.56 (0.93 to 7.06)	3.56 (1.6 to 7.94)	0.36
Respiratory	1.98 (1.46 to 2.7)	2.39 (0.94 to 6.07)	1.81 (0.87 to 3.76)	0.67
**Lifestyle factors (ref=Never)**				
Smoker				0.04
Past	1.52 (1.07 to 2.16)	0.74 (0.26 to 2.15)	1.32 (0.57 to 3.07)	
Current	0.71 (0.42 to 1.2)	0 (0 to Inf)	0.45 (0.14 to 1.42)	
Drinker				0.49
Past	1.44 (0.91 to 2.3)	0.24 (0.03 to 2.07)	0.91 (0.32 to 2.61)	
Current	0.87 (0.57 to 1.34)	0.56 (0.17 to 1.82)	0.43 (0.17 to 1.11)	
Substance user				0.4
Past	2.85 (1.24 to 6.58)	16.83 (1.95 to 145.12)	1.95 (0.43 to 8.95)	
Current	2.35 (1.39 to 3.95)	4.99 (0.96 to 26.04)	1.1 (0.37 to 3.25)	
Obese				0.59
Past	1.27 (0.83 to 1.96)	4.03 (1.23 to 13.24)	2.5 (1.05 to 5.92)	
Current	1.22 (0.87 to 1.73)	1.85 (0.56 to 6.1)	1.39 (0.56 to 3.46)	
**Prescription medication use (ref=Non-user)**				
ACE inhibitor				0.78
Past user	3 (1.86 to 4.82)	4.12 (1.02 to 16.7)	2.48 (0.68 to 9.02)	
Current user	0.7 (0.45 to 1.08)	1.11 (0.34 to 3.63)	1.22 (0.5 to 3)	

Angiotensin receptor blocker				0.36
Past user	1.52 (0.61 to 3.78)	0 (0 to Inf)	0 (0 to Inf)	
Current user	1.13 (0.71 to 1.77)	0.93 (0.2 to 4.29)	3.21 (1.26 to 8.15)	
Aldosterone agonist				0.33
Past user	3.45 (1.55 to 7.66)	0 (0 to Inf)	5.33 (1.06 to 26.73)	
Current user	1.16 (0.56 to 2.42)	2.47 (0.28 to 21.87)	1.19 (0.15 to 9.21)	
**β**-blocker				0.7
Past user	1.46 (0.67 to 3.19)	1.82 (0.21 to 15.44)	4.7 (1.47 to 15.02)	
Current user	1.63 (1.15 to 2.3)	1.76 (0.6 to 5.13)	1.21 (0.49 to 3.02)	
Calcium channel blocker				0.25
Past user	1.55 (0.86 to 2.8)	0 (0 to Inf)	2.54 (0.69 to 9.38)	
Current user	0.94 (0.64 to 1.38)	0.99 (0.33 to 2.95)	0.89 (0.36 to 2.24)	
**α**-agonist				0.93
Past user	0 (0 to 7.13e+251)	NA	NA	
Current user	1.2 (0.16 to 8.83)	0 (0 to Inf)	0 (0 to Inf)	
Thiazide				1
Past user	0 (0 to 3.25e+232)	0 (0 to Inf)	0 (0 to Inf)	
Current user	1.22 (0.17 to 8.97)	0 (0 to Inf)	0 (0 to Inf)	
Antiplatelet				0.82
Past user	1.63 (0.78 to 3.4)	1.79 (0.21 to 15.05)	0.77 (0.1 to 6.06)	
Current user	1.89 (1.32 to 2.71)	2.69 (0.95 to 7.67)	1.77 (0.75 to 4.15)	
Antiarrhythmic				0.23
Past user	1.39 (0.43 to 4.46)	3.17 (0.34 to 29.87)	3.29 (0.69 to 15.7)	
Current user	1.86 (1.09 to 3.16)	3.22 (0.65 to 15.87)	0.65 (0.08 to 4.96)	
Anticoagulant				0.33
Past user	1.72 (0.54 to 5.53)	5.22 (0.58 to 46.91)	0 (0 to Inf)	
Current user	1.55 (0.81 to 3)	1.91 (0.23 to 16.18)	0.71 (0.09 to 5.54)	
Glucocorticoid				0.88
Past user	1.41 (0.83 to 2.4)	0.69 (0.09 to 5.51)	1.29 (0.36 to 4.56)	
Current user	2.06 (1.47 to 2.88)	1.47 (0.52 to 4.11)	1.96 (0.88 to 4.38)	
**β**2-agonist				0.08
Past user	1.69 (0.68 to 4.2)	9.51 (2.34 to 38.57)	0 (0 to Inf)	
Current user	2.02 (1.37 to 2.99)	1.93 (0.52 to 7.23)	1.67 (0.65 to 4.28)	
Muscarinic antagonist				0.86
Past user	2.32 (1.12 to 4.83)	0 (0 to Inf)	2.79 (0.61 to 12.77)	
Current user	1.77 (1.17 to 2.68)	1.62 (0.44 to 5.96)	2.54 (1.07 to 5.99)	
NSAID				0.53
Past user	0.62 (0.27 to 1.41)	2.48 (0.52 to 11.86)	1.17 (0.26 to 5.15)	
Current user	1.44 (0.81 to 2.58)	3.08 (0.81 to 11.74)	0.87 (0.11 to 6.73)	
Vitamin D				0.41
Past user	1.93 (1.02 to 3.65)	3.91 (0.77 to 19.82)	2.63 (0.84 to 8.18)	
Current user	2.44 (1.71 to 3.48)	2.16 (0.71 to 6.62)	1.62 (0.68 to 3.88)	
Proton pump inhibitor				0.32
Past user	1.25 (0.69 to 2.28)	0 (0 to Inf)	0.55 (0.07 to 4.42)	
Current user	1.51 (1.08 to 2.1)	2.8 (0.95 to 8.29)	2.03 (0.88 to 4.68)	
Statin				0.89
Past user	2.25 (1.18 to 4.27)	2.21 (0.41 to 11.97)	1.77 (0.37 to 8.53)	
Current user	1.8 (1.26 to 2.57)	1.34 (0.45 to 3.96)	2.15 (0.92 to 5)	
Immunosuppressant				0.76
Past user	1.14 (0.36 to 3.65)	2.49 (0.27 to 22.9)	1.91 (0.24 to 15.09)	
Current user	1.85 (0.85 to 4)	2.82 (0.34 to 23.76)	0 (0 to Inf)	

	Liver disease			
	No (N=6863)	Mild (N=7098)	Moderate/Severe (N=1038)	
	OR (95% CI)	OR (95% CI)	OR (95% CI)	P-het
**Demographics**				
Gender (ref=Female)				0.41
Male	1.48 (0.94 to 2.31)	1.62 (1.1 to 2.37)	0.94 (0.42 to 2.11)	
Ethnicity (ref=White)				0.78
South Asian	1.29 (0.76 to 2.22)	1.51 (0.96 to 2.37)	1.07 (0.39 to 2.94)	
Black	1.9 (1.02 to 3.56)	2.58 (1.52 to 4.38)	1.87 (0.58 to 6.06)	
Other	0.65 (0.25 to 1.66)	1.09 (0.55 to 2.14)	1.12 (0.31 to 4.08)	
Age group (ref=18-40)				0.24
41-50	1.05 (0.35 to 3.13)	1.02 (0.5 to 2.07)	1.1 (0.18 to 6.81)	
51-60	0.79 (0.25 to 2.52)	1.21 (0.63 to 2.33)	0.71 (0.12 to 4.36)	
61-70	2.21 (0.86 to 5.68)	1.04 (0.51 to 2.1)	2.38 (0.5 to 11.33)	
71-80	3.57 (1.44 to 8.82)	2.08 (1.04 to 4.18)	3.34 (0.63 to 17.88)	
80+	9.14 (4 to 20.85)	3.45 (1.67 to 7.12)	3.99 (0.64 to 24.98)	
**HPB disease (ref=No)**				
Pancreatic disease				0.02
Acute	1.32 (0.64 to 2.75)	1.6 (0.69 to 3.73)	1.89 (0.42 to 8.58)	
Chronic	1.96 (0.97 to 3.98)	1.96 (1 to 3.83)	1.12 (0.25 to 5.06)	
Biliary disease				0.01
Acute	1.15 (0.43 to 3.03)	0.93 (0.29 to 3)	1.07 (0.14 to 8.46)	
Chronic	1.03 (0.52 to 2.05)	0.93 (0.57 to 1.52)	1.79 (0.74 to 4.32)	
**Comorbidities (ref=No)**				
Diabetes	2.73 (1.66 to 4.5)	2.36 (1.56 to 3.56)	2.41 (0.98 to 5.93)	0.14
Hypertension	2.08 (1.05 to 4.14)	1.87 (1.13 to 3.1)	8.74 (1.14 to 66.9)	0.1
Cholesterol	1.16 (0.71 to 1.91)	1.43 (0.94 to 2.16)	2.06 (0.79 to 5.4)	0.76
Cardiovascular	2.19 (1.34 to 3.57)	2.85 (1.9 to 4.27)	3.93 (1.6 to 9.66)	0.79
Renal	2.61 (1.63 to 4.18)	3.98 (2.64 to 5.99)	1.56 (0.67 to 3.63)	0.16
Respiratory	2.29 (1.47 to 3.58)	1.92 (1.31 to 2.8)	1.47 (0.65 to 3.35)	0.39
**Lifestyle factors (ref=Never)**				
Smoker				0.15
Past	1.36 (0.84 to 2.22)	1.35 (0.88 to 2.09)	1.63 (0.61 to 4.3)	
Current	0.29 (0.1 to 0.85)	0.7 (0.39 to 1.28)	0.71 (0.2 to 2.55)	
Drinker				0.66
Past	0.94 (0.47 to 1.9)	1.33 (0.76 to 2.3)	1.8 (0.46 to 7)	
Current	0.97 (0.55 to 1.72)	0.6 (0.36 to 1.01)	1 (0.29 to 3.45)	
Substance user				0.04
Past	3.43 (1.14 to 10.3)	3.53 (1.46 to 8.51)	0 (0 to Inf)	
Current	2.84 (1.37 to 5.87)	1.45 (0.78 to 2.69)	7.26 (0.9 to 58.68)	
Obese				0.55
Past	2.01 (1.14 to 3.54)	1.14 (0.65 to 1.98)	2.59 (0.97 to 6.94)	
Current	1.43 (0.84 to 2.42)	1.17 (0.77 to 1.77)	1.78 (0.65 to 4.86)	
**Prescription medication use (ref=Non-user)**				
ACE inhibitor				0.31
Past user	3.67 (1.89 to 7.1)	2.05 (1.04 to 4.05)	5.91 (2.11 to 16.55)	
Current user	0.93 (0.51 to 1.67)	0.66 (0.39 to 1.12)	1.07 (0.34 to 3.36)	
Angiotensin receptor blocker				0.08
Past user	1.24 (0.29 to 5.2)	0 (0 to Inf)	5.41 (1.36 to 21.48)	
Current user	1.09 (0.56 to 2.1)	1.61 (0.97 to 2.67)	0.48 (0.06 to 3.67)	

Aldosterone agonist				0.17
Past user	4.13 (1.18 to 14.45)	3.9 (1.38 to 11.03)	2.26 (0.49 to 10.39)	
Current user	0.5 (0.07 to 3.64)	0.9 (0.28 to 2.87)	2.36 (0.89 to 6.25)	
**β**-blocker				0.33
Past user	0.79 (0.19 to 3.29)	1.79 (0.71 to 4.52)	6.03 (1.96 to 18.56)	
Current user	1.32 (0.8 to 2.18)	1.94 (1.26 to 2.97)	1.28 (0.5 to 3.23)	
Calcium channel blocker				0.08
Past user	1.91 (0.92 to 3.98)	1.45 (0.65 to 3.24)	0 (0 to Inf)	
Current user	0.57 (0.31 to 1.06)	1.29 (0.83 to 2)	0.9 (0.32 to 2.53)	
**α**-agonist				0.93
Past user	0 (0 to Inf)	0 (0 to Inf)	0 (0 to Inf)	
Current user	1.8 (0.23 to 13.81)	0 (0 to Inf)	0 (0 to Inf)	
Thiazide				0.76
Past user	0 (0 to Inf)	0 (0 to Inf)	NA	
Current user	0 (0 to Inf)	2.14 (0.28 to 16.16)	0 (0 to Inf)	
Antiplatelet				0.77
Past user	1.43 (0.5 to 4.08)	1.56 (0.62 to 3.94)	1.28 (0.16 to 10.39)	
Current user	1.82 (1.1 to 3.01)	1.93 (1.23 to 3.03)	2.39 (0.96 to 5.92)	
Antiarrhythmic				0.13
Past user	3.69 (1.28 to 10.63)	1.28 (0.3 to 5.4)	0 (0 to Inf)	
Current user	1.35 (0.57 to 3.19)	2.35 (1.23 to 4.51)	1.13 (0.25 to 5.05)	
Anticoagulant				0.5
Past user	1.9 (0.45 to 8.04)	1.67 (0.4 to 7.06)	0 (0 to Inf)	
Current user	1.46 (0.58 to 3.71)	1.63 (0.7 to 3.82)	0.8 (0.1 to 6.32)	
Glucocorticoid				0.56
Past user	1.3 (0.58 to 2.92)	1.48 (0.8 to 2.73)	0.56 (0.07 to 4.41)	
Current user	2.3 (1.43 to 3.7)	1.8 (1.18 to 2.73)	1.76 (0.74 to 4.18)	
**β**2-agonist				0.28
Past user	2.86 (0.87 to 9.43)	2.04 (0.81 to 5.13)	0 (0 to Inf)	
Current user	2.22 (1.3 to 3.8)	1.73 (1.04 to 2.88)	1.89 (0.67 to 5.29)	
Muscarinic antagonist				0.44
Past user	1.22 (0.29 to 5.11)	2.91 (1.31 to 6.46)	2.27 (0.28 to 18.28)	
Current user	2.3 (1.35 to 3.91)	1.4 (0.8 to 2.45)	2.25 (0.8 to 6.33)	
NSAID				0.17
Past user	1.42 (0.6 to 3.34)	0.42 (0.13 to 1.32)	0.94 (0.12 to 7.49)	
Current user	2.49 (1.22 to 5.11)	1.21 (0.58 to 2.53)	0 (0 to Inf)	
Vitamin D				0.06
Past user	4.19 (2.1 to 8.36)	1.36 (0.54 to 3.42)	0.58 (0.07 to 4.54)	
Current user	2.28 (1.36 to 3.82)	2.51 (1.61 to 3.9)	1.5 (0.61 to 3.69)	
Proton pump inhibitor				0.73
Past user	0.78 (0.3 to 2.02)	1.4 (0.65 to 3.03)	0.62 (0.07 to 5.22)	
Current user	1.12 (0.7 to 1.8)	2.1 (1.38 to 3.19)	2.17 (0.87 to 5.42)	
Statin				0.35
Past user	3.23 (1.45 to 7.2)	1.3 (0.5 to 3.33)	2.04 (0.41 to 10.12)	
Current user	1.7 (1 to 2.9)	1.76 (1.15 to 2.69)	2.08 (0.85 to 5.1)	
Immunosuppressant				0.65
Past user	2.45 (0.74 to 8.08)	1.09 (0.26 to 4.53)	0 (0 to Inf)	
Current user	1.25 (0.3 to 5.23)	1.66 (0.6 to 4.6)	3.04 (0.66 to 14.08)	

	Biliary disease			
	No (N=7716)	Acute (N=749)	Chronic (N=6534)	
	OR (95% CI)	OR (95% CI)	OR (95% CI)	P-het
**Demographics**				
Gender (ref=Female)				0.72
Male	1.35 (0.94 to 1.94)	1.35 (0.38 to 4.83)	1.58 (1.01 to 2.47)	
Ethnicity (ref=White)				0.6
South Asian	1.33 (0.86 to 2.03)	1.67 (0.43 to 6.57)	1.42 (0.83 to 2.42)	
Black	1.78 (1.06 to 2.98)	2.26 (0.41 to 12.54)	3.2 (1.75 to 5.86)	
Other	0.98 (0.52 to 1.84)	0 (0 to Inf)	0.99 (0.43 to 2.26)	
Age group (ref=18-40)				0.13
41-50	1.1 (0.57 to 2.09)	2.4 (0.21 to 27.67)	0.63 (0.15 to 2.63)	
51-60	1.01 (0.54 to 1.89)	1.44 (0.12 to 17.27)	1.36 (0.44 to 4.22)	
61-70	1.07 (0.56 to 2.05)	0.95 (0.06 to 15.97)	3.37 (1.23 to 9.29)	
71-80	1.71 (0.88 to 3.33)	1.18 (0.07 to 20.15)	6.46 (2.39 to 17.44)	
80+	4.16 (2.19 to 7.89)	6.53 (0.66 to 64.15)	10.15 (3.86 to 26.68)	
**HPB disease (ref=No)**				
Pancreatic disease				0.93
Acute	1.92 (0.97 to 3.82)	0 (0 to Inf)	1.04 (0.44 to 2.43)	
Chronic	2.2 (1.22 to 3.98)	3.68 (0.87 to 15.57)	1.25 (0.53 to 2.96)	
Liver disease				0.59
Mild	2.05 (1.09 to 3.88)	1.22 (0.29 to 5.17)	1.55 (0.92 to 2.61)	
Moderate/Severe	2.63 (1.22 to 5.66)	1.45 (0.15 to 13.66)	3.95 (1.8 to 8.67)	
**Comorbidities (ref=No)**				
Diabetes	2.58 (1.75 to 3.82)	2.49 (0.64 to 9.69)	2.25 (1.36 to 3.71)	0.22
Hypertension	2.16 (1.33 to 3.5)	0.87 (0.18 to 4.14)	2.5 (1.15 to 5.45)	0.16
Cholesterol	1.3 (0.89 to 1.9)	1.49 (0.36 to 6.16)	1.48 (0.87 to 2.52)	0.59
Cardiovascular	3.31 (2.26 to 4.86)	1.04 (0.26 to 4.19)	2.09 (1.28 to 3.41)	0.45
Renal	3.21 (2.19 to 4.72)	2.73 (0.73 to 10.11)	2.73 (1.7 to 4.38)	0.23
Respiratory	1.96 (1.37 to 2.8)	4.25 (1.2 to 15.04)	1.77 (1.13 to 2.77)	0.43
**Lifestyle factors (ref=Never)**				
Smoker				0.14
Past	1.2 (0.8 to 1.81)	2.38 (0.54 to 10.62)	1.58 (0.97 to 2.58)	
Current	0.55 (0.31 to 0.97)	1.42 (0.21 to 9.7)	0.38 (0.13 to 1.09)	
Drinker				0.9
Past	1.38 (0.79 to 2.42)	0.41 (0.04 to 3.97)	1.15 (0.61 to 2.16)	
Current	0.75 (0.46 to 1.23)	0.71 (0.16 to 3.12)	0.76 (0.42 to 1.37)	
Substance user				0.03
Past	2.6 (1.03 to 6.53)	0 (0 to Inf)	4.95 (1.68 to 14.58)	
Current	1.41 (0.78 to 2.57)	0.97 (0.19 to 4.91)	4.5 (2.08 to 9.74)	
Obese				0.84
Past	1.58 (0.98 to 2.56)	0.73 (0.14 to 3.88)	1.83 (1.01 to 3.31)	
Current	1.23 (0.82 to 1.84)	0.68 (0.16 to 2.93)	1.65 (0.96 to 2.81)	
**Prescription medication use (ref=Non-user)**				
ACE inhibitor				0.88
Past user	3.04 (1.72 to 5.38)	4.61 (0.79 to 26.78)	2.84 (1.44 to 5.61)	
Current user	0.76 (0.46 to 1.25)	1.09 (0.21 to 5.75)	0.8 (0.44 to 1.43)	
Angiotensin receptor blocker				0.74
Past user	0.77 (0.19 to 3.16)	0 (0 to Inf)	1.73 (0.52 to 5.77)	
Current user	1.37 (0.82 to 2.3)	3.39 (0.77 to 14.93)	1.01 (0.52 to 1.95)	

Aldosterone agonist				0.31
Past user	4.05 (1.7 to 9.64)	0 (0 to Inf)	2.62 (0.76 to 9.05)	
Current user	1.32 (0.57 to 3.09)	0 (0 to Inf)	1.27 (0.45 to 3.61)	
**β**-blocker				0.23
Past user	2.42 (1.15 to 5.09)	8.19 (0.78 to 86.4)	1.1 (0.33 to 3.6)	
Current user	1.52 (1 to 2.31)	0.39 (0.05 to 3.31)	1.77 (1.1 to 2.85)	
Calcium channel blocker				0.12
Past user	1.19 (0.51 to 2.79)	2.22 (0.24 to 20.37)	1.64 (0.79 to 3.42)	
Current user	1.28 (0.84 to 1.95)	2.19 (0.55 to 8.83)	0.49 (0.26 to 0.91)	
**α**-agonist				0.75
Past user	0 (0 to Inf)	NA	0 (0 to Inf)	
Current user	0 (0 to Inf)	0 (0 to Inf)	2.38 (0.3 to 18.73)	
Thiazide				0.81
Past user	0 (0 to Inf)	NA	0 (0 to Inf)	
Current user	1.94 (0.26 to 14.41)	0 (0 to Inf)	0 (0 to Inf)	
Antiplatelet				0.4
Past user	1.59 (0.68 to 3.7)	0 (0 to Inf)	1.36 (0.47 to 3.91)	
Current user	1.77 (1.15 to 2.73)	1.38 (0.32 to 5.95)	2.11 (1.29 to 3.45)	
Antiarrhythmic				0.34
Past user	2.07 (0.64 to 6.74)	0 (0 to Inf)	2.15 (0.64 to 7.22)	
Current user	1.95 (1.03 to 3.7)	2.71 (0.31 to 23.91)	1.41 (0.63 to 3.16)	
Anticoagulant				0.49
Past user	1.43 (0.34 to 5.94)	0 (0 to Inf)	2.01 (0.47 to 8.59)	
Current user	1.31 (0.56 to 3.03)	5.28 (0.58 to 48.33)	1.31 (0.51 to 3.36)	
Glucocorticoid				0.35
Past user	1.69 (0.96 to 2.99)	1.11 (0.13 to 9.69)	0.83 (0.33 to 2.13)	
Current user	1.77 (1.19 to 2.64)	1.27 (0.31 to 5.22)	2.38 (1.49 to 3.8)	
**β**2-agonist				0.46
Past user	2.46 (1.06 to 5.75)	0 (0 to Inf)	1.52 (0.36 to 6.36)	
Current user	1.89 (1.18 to 3.02)	0.85 (0.1 to 7.22)	2.09 (1.22 to 3.57)	
Muscarinic antagonist				0.52
Past user	3.51 (1.66 to 7.41)	0 (0 to Inf)	1.04 (0.25 to 4.33)	
Current user	1.72 (1.05 to 2.83)	2.14 (0.42 to 10.99)	2.06 (1.19 to 3.56)	
NSAID				0.65
Past user	0.58 (0.21 to 1.59)	0 (0 to Inf)	1.23 (0.52 to 2.91)	
Current user	1.7 (0.88 to 3.3)	1.97 (0.23 to 17.04)	1.22 (0.52 to 2.86)	
Vitamin D				0.15
Past user	1.87 (0.89 to 3.94)	6.13 (1.03 to 36.35)	2.34 (1.03 to 5.32)	
Current user	2.35 (1.54 to 3.58)	0.56 (0.07 to 4.71)	2.45 (1.5 to 4.01)	
Proton pump inhibitor				0.42
Past user	1.03 (0.46 to 2.29)	0.94 (0.11 to 7.98)	1.09 (0.44 to 2.67)	
Current user	1.87 (1.28 to 2.74)	0.39 (0.09 to 1.72)	1.53 (0.93 to 2.51)	
Statin				0.22
Past user	1.71 (0.76 to 3.84)	0 (0 to Inf)	3.31 (1.47 to 7.47)	
Current user	1.82 (1.22 to 2.72)	1.06 (0.28 to 4.03)	1.72 (1 to 2.96)	
Immunosuppressant				0.36
Past user	0.49 (0.07 to 3.56)	0 (0 to Inf)	3.28 (1.15 to 9.38)	
Current user	2 (0.8 to 4.99)	6.18 (0.65 to 59.04)	0.98 (0.23 to 4.07)	

Odds ratios (ORs) are mutually adjusted for gender, ethnicity, age group, and other HPB diagnoses.
Dichotomous age groups (over and under 60) are used for controlling for all categories except demographics.
All P values presented are Benjamini-Hochberg corrected.
