# Supplementary material for: COVID-19 in patients with hepatobiliary and pancreatic diseases: a single-centre cross-sectional study in East London
Source: BMJ Open. 2021 Apr 19;11(4):e045077. doi: 10.1136/bmjopen-2020-045077 (PMC8057071; doi:10.1136/bmjopen-2020-045077)
Supplement: Supplementary data [file bmjopen-2020-045077supp006.pdf]

Supplemental Table 6 Odds ratio estimates of COVID-19 mortality for HPB patients with specific demographic, comorbidity, lifestyle, medication use, and post diagnosis complication characteristics.

	Crude OR (95% CI)	P value	Adjusted OR (95% CI)	P value	Adjusted OR (+all comorbidity) (95% CI)	P value
**Demographics**						
Gender (ref=Female)						
Male	2.53 (1.29 to 4.96)	0.007	3.54 (1.68 to 7.85)	0.007	3.43 (1.61 to 7.67)	0.008
Ethnicity (ref=White)						
South Asian	1.39 (0.67 to 2.89)	0.477	2.08 (0.91 to 4.88)	0.143	1.52 (0.64 to 3.62)	0.492
Black	2.35 (1.02 to 5.41)	0.109	3.77 (1.38 to 10.7)	0.023	3.51 (1.27 to 10)	0.053
Other	0.21 (0.03 to 1.63)	0.225	0.29 (0.02 to 1.82)	0.371	0.18 (0.01 to 1.19)	0.259
Age group (ref=18-40)						
41-50	1.83 (0.15 to 21.6)	0.76	2.24 (0.19 to 52.1)	0.65	1.86 (0.13 to 48.8)	0.858
51-60	1.35 (0.12 to 15.9)	0.809	1.92 (0.16 to 44.3)	0.674	1.48 (0.11 to 37.4)	0.938
61-70	5.09 (0.59 to 43.7)	0.207	6.73 (1.03 to 134)	0.143	4.12 (0.51 to 90.6)	0.379
71-80	13.4 (1.64 to 110)	0.031	18.6 (3.12 to 361)	0.022	8.8 (1.12 to 192)	0.204
80+	16.3 (2.04 to 130)	0.025	25.4 (4.32 to 491)	0.012	13.2 (1.76 to 283)	0.13
**HPB disease (ref=No)**						
Cancer						
Yes	2.1 (0.34 to 12.9)	0.422	1.18 (0.14 to 10.2)	0.951	1.35 (0.13 to 13.7)	0.959
Pancreatic disease						
Acute	1.4 (0.47 to 4.15)	0.549	1.52 (0.44 to 5.24)	0.596	1.16 (0.32 to 4.23)	0.959
Chronic	2.82 (1.26 to 6.34)	0.024	3.26 (1.13 to 9.44)	0.082	2.65 (0.84 to 8.32)	0.283
Liver disease						
Mild	0.43 (0.22 to 0.85)	0.03	0.51 (0.19 to 1.35)	0.347	0.43 (0.15 to 1.19)	0.283
Moderate/Severe	0.53 (0.18 to 1.56)	0.33	0.6 (0.16 to 2.22)	0.568	0.47 (0.12 to 1.81)	0.454
Biliary disease						
Acute	3.81 (1.02 to 14.1)	0.092	2.44 (0.38 to 15.9)	0.544	3.98 (0.46 to 34.6)	0.42
Chronic	1.34 (0.68 to 2.61)	0.395	0.67 (0.26 to 1.72)	0.568	0.62 (0.24 to 1.62)	0.513
**Comorbidities (ref=No)**						
Diabetes	2.51 (1.24 to 5.44)	0.014	1.88 (0.81 to 4.38)	0.227	1.71 (0.71 to 4.11)	0.38
Hypertension	12.6 (2.6 to 227)	0.014	6.3 (0.74 to 53.8)	0.148	5.57 (0.57 to 54.3)	0.259
Cholesterol	1.87 (0.93 to 3.97)	0.088	1.25 (0.56 to 2.82)	0.67	0.95 (0.4 to 2.22)	0.979
Cardiovascular	4.1 (2 to 9.13)	<0.001	2.82 (1.21 to 6.59)	0.026	2.66 (1.05 to 6.79)	0.104
Renal	3.67 (1.9 to 7.38)	<0.001	1.72 (0.8 to 3.7)	0.248	1.07 (0.47 to 2.47)	0.979
Respiratory	1.09 (0.59 to 2.03)	0.784	0.85 (0.42 to 1.73)	0.743	0.83 (0.38 to 1.78)	0.815
**Lifestyle factors (ref=Never)**						
Smoker						
Past	2.46 (1.22 to 4.95)	0.023	1.87 (0.83 to 4.19)	0.219	1.86 (0.79 to 4.36)	0.274
Current	0.42 (0.09 to 1.97)	0.359	0.45 (0.08 to 2.49)	0.451	0.44 (0.08 to 2.58)	0.533
Drinker						
Past	1.38 (0.55 to 3.45)	0.761	1.2 (0.41 to 3.49)	0.847	1.07 (0.36 to 3.22)	0.964
Current	1.26 (0.57 to 2.79)	0.761	1.38 (0.52 to 3.64)	0.736	1.44 (0.52 to 3.96)	0.774

Substance user						
Past	1.2 (0.19 to 7.64)	0.847	0.62 (0.08 to 4.94)	0.81	0.31 (0.03 to 2.87)	0.488
Current	3.17 (1 to 10)	0.1	2.01 (0.53 to 7.64)	0.436	1.34 (0.32 to 5.55)	0.842
Obese						
Past	1.64 (0.74 to 3.66)	0.453	1.46 (0.58 to 3.65)	0.603	0.97 (0.36 to 2.59)	0.995
Current	1.04 (0.5 to 2.16)	0.986	1.03 (0.44 to 2.4)	0.991	0.91 (0.37 to 2.2)	0.995
**Prescription medication use (ref=Non-user)**						
ACE inhibitor						
Past user	1.48 (0.6 to 3.66)	0.397	1.02 (0.38 to 2.75)	0.988	0.84 (0.29 to 2.42)	0.889
Current user	2.02 (0.9 to 4.5)	0.13	2.25 (0.88 to 5.73)	0.161	1.97 (0.73 to 5.32)	0.337
Angiotensin receptor blocker						
Past user	2.12 (0.34 to 13.1)	0.626	1.07 (0.14 to 8.29)	0.988	0.78 (0.1 to 6.34)	0.987
Current user	1.06 (0.44 to 2.54)	0.892	0.86 (0.32 to 2.32)	0.988	0.81 (0.28 to 2.35)	0.946
Aldosterone agonist						
Past user	1.6 (0.38 to 6.63)	0.78	1.29 (0.28 to 5.82)	0.838	0.88 (0.19 to 4.1)	0.989
Current user	1.2 (0.3 to 4.69)	0.797	1.29 (0.28 to 5.97)	0.838	0.97 (0.2 to 4.67)	0.989
β-blocker						
Past user	1.1 (0.28 to 4.32)	0.887	0.72 (0.16 to 3.24)	0.746	0.29 (0.06 to 1.46)	0.204
Current user	1.22 (0.62 to 2.38)	0.848	0.66 (0.3 to 1.45)	0.385	0.26 (0.1 to 0.67)	0.017
Calcium channel blocker						
Past user	0.47 (0.1 to 2.18)	0.338	0.16 (0.03 to 0.89)	0.065	0.07 (0.01 to 0.45)	0.016
Current user	1.56 (0.77 to 3.15)	0.324	0.92 (0.4 to 2.12)	0.948	0.52 (0.21 to 1.29)	0.243
α-agonist						
Past user	0 (0 to Inf)	0.988	0 (0 to Inf)	0.992	0 (0 to Inf)	0.994
Thiazide						
Past user	0 (0 to Inf)	0.988	0 (0 to Inf)	0.993	0 (0 to Inf)	0.995
Current user	1 (0.2 to 4.97)	1	0.68 (0.12 to 3.7)	0.836	0.53 (0.09 to 3.17)	0.659
Antiplatelet						
Past user	2.09 (1.09 to 4.02)	0.04	0.99 (0.45 to 2.17)	0.988	0.68 (0.29 to 1.58)	0.56
Antiarrhythmic						
Past user	0.57 (0.07 to 4.86)	0.607	0.53 (0.06 to 4.82)	0.641	0.31 (0.03 to 3.1)	0.529
Current user	2.8 (1.09 to 7.19)	0.049	2.12 (0.72 to 6.26)	0.261	1.66 (0.52 to 5.3)	0.531
Anticoagulant						
Past user	0.81 (0.09 to 7.41)	0.851	0.48 (0.05 to 4.89)	0.691	0.34 (0.03 to 3.57)	0.555
Current user	2.02 (0.63 to 6.48)	0.354	1.18 (0.32 to 4.39)	0.904	0.94 (0.24 to 3.66)	0.989
Glucocorticoid						
Past user	1.88 (0.65 to 5.41)	0.245	1.47 (0.44 to 4.87)	0.599	1.62 (0.46 to 5.68)	0.564
Current user	2.64 (1.35 to 5.18)	0.007	2.79 (1.26 to 6.22)	0.021	3.66 (1.49 to 9.02)	0.014
β2-agonist						
Past user	1.32 (0.26 to 6.86)	0.739	1.96 (0.32 to 12)	0.523	5.41 (0.65 to 45.1)	0.222
Current user	2.65 (1.3 to 5.37)	0.011	2.72 (1.18 to 6.25)	0.034	5.24 (1.78 to 15.4)	0.01
Muscarinic antagonist						
Past user	2.65 (0.68 to 10.3)	0.242	1.58 (0.32 to 7.7)	0.739	1.85 (0.32 to 10.5)	0.733
Current user	1.1 (0.5 to 2.45)	0.811	1.11 (0.45 to 2.76)	0.919	1.16 (0.44 to 3.09)	0.95

NSAID						
Past user	0.85 (0.17 to 4.15)	0.84	0.58 (0.11 to 3.05)	0.589	0.7 (0.12 to 3.99)	0.795
Current user	2.72 (1.01 to 7.31)	0.072	4.13 (1.19 to 14.3)	0.045	4.24 (1.15 to 15.6)	0.089
Vitamin D						
Past user	2.1 (0.72 to 6.11)	0.175	1.8 (0.55 to 5.92)	0.376	1.51 (0.43 to 5.34)	0.653
Current user	2.05 (1.04 to 4.02)	0.056	1.58 (0.7 to 3.53)	0.345	1.5 (0.61 to 3.7)	0.625
Proton pump inhibitor						
Past user	0.94 (0.24 to 3.73)	0.929	1.46 (0.29 to 7.34)	0.83	0.95 (0.17 to 5.35)	0.989
Current user	1.21 (0.62 to 2.35)	0.855	0.88 (0.41 to 1.89)	0.83	0.51 (0.22 to 1.23)	0.27
Statin						
Past user	1.64 (0.4 to 6.74)	0.495	0.82 (0.18 to 3.77)	0.902	0.4 (0.07 to 2.25)	0.494
Current user	3.19 (1.52 to 6.69)	0.003	1.51 (0.62 to 3.66)	0.47	0.82 (0.27 to 2.47)	0.882
Immunosuppressant						
Past user	2.17 (0.35 to 13.3)	0.405	1.3 (0.17 to 10)	0.904	1.63 (0.19 to 14.1)	0.819
Current user	1.95 (0.45 to 8.46)	0.405	2.44 (0.44 to 13.7)	0.4	3.41 (0.56 to 20.6)	0.341
**Complications post diagnosis (ref=No)**						
**Cardiovascular**						
Recurrent	3.75 (1.71 to 8.25)	0.001	2.53 (1.04 to 6.12)	0.071	2.37 (0.9 to 6.26)	0.231
Novel	0.52 (0.06 to 4.47)	0.555	0.44 (0.04 to 4.47)	0.547	0.42 (0.04 to 4.7)	0.668
**Respiratory**						
Recurrent	3.31 (1.19 to 9.16)	0.021	2.53 (0.84 to 7.63)	0.15	2.69 (0.84 to 8.63)	0.188
Novel	5.88 (2.02 to 17.1)	0.002	5.77 (1.75 to 19)	0.009	6.55 (1.88 to 22.9)	0.011
**Renal**						
Recurrent	4.75 (2.06 to 10.9)	<0.001	1.72 (0.66 to 4.46)	0.34	0.8 (0.27 to 2.35)	0.797
Novel	1.95 (0.65 to 5.88)	0.233	1 (0.3 to 3.34)	0.997	0.58 (0.16 to 2.16)	0.585

	**Adjusted OR (+Diabetes) (95% CI)**	**P value**	**Adjusted OR (+Hypertension) (95% CI)**	**P value**	**Adjusted OR (+Cholesterol) (95% CI)**	**P value**
**Demographics**						
Gender (ref=Female)						
Male	3.31 (1.6 to 7.19)	0.005	3.35 (1.62 to 7.24)	0.004	3.18 (1.55 to 6.84)	0.006
Ethnicity (ref=White)						
South Asian	1.55 (0.67 to 3.6)	0.349	1.79 (0.79 to 4.06)	0.216	1.8 (0.8 to 4.08)	0.252
Black	3.23 (1.22 to 8.82)	0.039	3.67 (1.4 to 9.97)	0.018	3.55 (1.36 to 9.57)	0.021
Other	0.24 (0.01 to 1.48)	0.267	0.26 (0.01 to 1.61)	0.261	0.27 (0.01 to 1.63)	0.314
Age group (ref=18-40)						
41-50	1.88 (0.16 to 44.3)	0.753	2.43 (0.2 to 58.2)	0.597	2.1 (0.17 to 50.1)	0.76
51-60	1.47 (0.12 to 34.8)	0.836	2.01 (0.17 to 47.3)	0.646	1.81 (0.15 to 42.7)	0.778
61-70	4.91 (0.71 to 100)	0.287	6.1 (0.9 to 124)	0.196	6.34 (0.93 to 129)	0.184
71-80	12.4 (1.91 to 249)	0.063	15.3 (2.45 to 301)	0.035	17.3 (2.69 to 347)	0.028
80+	19.9 (3.26 to 391)	0.029	20 (3.25 to 395)	0.029	23.8 (3.83 to 473)	0.019

**HPB disease (ref=No)**						
Cancer						
Yes	1.41 (0.16 to 12.8)	0.811	1.04 (0.12 to 9.04)	0.988	1.16 (0.13 to 10.3)	0.957
Pancreatic disease						
Acute	1.5 (0.43 to 5.18)	0.605	1.37 (0.4 to 4.74)	0.715	1.5 (0.43 to 5.18)	0.653
Chronic	2.88 (0.97 to 8.55)	0.171	3.19 (1.09 to 9.34)	0.102	3.19 (1.1 to 9.27)	0.099
Liver disease						
Mild	0.49 (0.19 to 1.31)	0.39	0.48 (0.18 to 1.27)	0.26	0.5 (0.19 to 1.32)	0.346
Moderate/Severe	0.58 (0.16 to 2.12)	0.509	0.53 (0.14 to 1.97)	0.471	0.59 (0.16 to 2.19)	0.591
Biliary disease						
Acute	2.45 (0.37 to 16.5)	0.496	3.05 (0.38 to 24.2)	0.437	2.4 (0.36 to 15.9)	0.591
Chronic	0.65 (0.25 to 1.66)	0.496	0.67 (0.26 to 1.7)	0.499	0.67 (0.26 to 1.71)	0.591
**Comorbidities (ref=No)**						
Diabetes	1.88 (0.81 to 4.38)	0.227	1.87 (0.8 to 4.38)	0.222	1.86 (0.8 to 4.33)	0.271
Hypertension	6.35 (0.73 to 55.4)	0.17	6.3 (0.74 to 53.8)	0.148	6.09 (0.7 to 52.7)	0.181
Cholesterol	1.21 (0.53 to 2.74)	0.734	1.1 (0.48 to 2.52)	0.914	1.25 (0.56 to 2.82)	0.67
Cardiovascular	2.76 (1.17 to 6.48)	0.036	2.66 (1.13 to 6.27)	0.046	2.8 (1.19 to 6.62)	0.034
Renal	1.61 (0.74 to 3.5)	0.291	1.53 (0.71 to 3.31)	0.312	1.7 (0.79 to 3.66)	0.287
Respiratory	0.93 (0.45 to 1.94)	0.963	0.9 (0.44 to 1.84)	0.86	0.84 (0.41 to 1.72)	0.719
**Lifestyle factors (ref=Never)**						
Smoker						
Past	1.92 (0.84 to 4.37)	0.218	1.86 (0.82 to 4.2)	0.222	1.85 (0.82 to 4.17)	0.252
Current	0.42 (0.08 to 2.37)	0.401	0.46 (0.08 to 2.58)	0.458	0.45 (0.08 to 2.5)	0.498
Drinker						
Past	1.19 (0.41 to 3.48)	0.824	1.21 (0.41 to 3.53)	0.806	1.18 (0.4 to 3.45)	0.84
Current	1.42 (0.54 to 3.75)	0.657	1.29 (0.49 to 3.43)	0.806	1.37 (0.52 to 3.62)	0.812
Substance user						
Past	0.65 (0.08 to 5.32)	0.837	0.53 (0.07 to 4.35)	0.68	0.57 (0.07 to 4.69)	0.745
Current	2.17 (0.56 to 8.41)	0.361	1.65 (0.43 to 6.32)	0.634	1.92 (0.5 to 7.37)	0.538
Obese						
Past	1.33 (0.52 to 3.39)	0.749	1.34 (0.53 to 3.39)	0.733	1.43 (0.57 to 3.61)	0.704
Current	0.92 (0.39 to 2.18)	0.991	1.05 (0.45 to 2.46)	0.994	1.03 (0.44 to 2.4)	0.991
**Prescription medication use (ref=Non-user)**						
ACE inhibitor						
Past user	0.97 (0.36 to 2.61)	0.988	0.93 (0.34 to 2.53)	0.986	0.99 (0.36 to 2.74)	0.988
Current user	2.07 (0.8 to 5.34)	0.25	1.96 (0.76 to 5.01)	0.234	2.19 (0.84 to 5.69)	0.218
Angiotensin receptor blocker						
Past user	0.95 (0.12 to 7.27)	0.988	1 (0.13 to 7.75)	0.998	1.16 (0.15 to 8.83)	0.988
Current user	0.82 (0.3 to 2.24)	0.877	0.8 (0.3 to 2.17)	0.823	0.82 (0.3 to 2.23)	0.866
Aldosterone agonist						
Past user	1.26 (0.28 to 5.72)	0.86	1.15 (0.26 to 5.2)	0.947	1.24 (0.28 to 5.64)	0.892
Current user	1.25 (0.27 to 5.78)	0.86	1.18 (0.25 to 5.49)	0.947	1.22 (0.26 to 5.75)	0.892
β-blocker						
Past user	0.68 (0.15 to 3.04)	0.679	0.6 (0.13 to 2.71)	0.561	0.73 (0.16 to 3.32)	0.758

Current user	0.6 (0.27 to 1.35)	0.298	0.56 (0.25 to 1.26)	0.229	0.61 (0.27 to 1.38)	0.397
Calcium channel blocker						
Past user	0.14 (0.02 to 0.83)	0.06	0.13 (0.02 to 0.76)	0.047	0.14 (0.02 to 0.82)	0.058
Current user	0.89 (0.38 to 2.07)	0.876	0.79 (0.34 to 1.84)	0.656	0.88 (0.38 to 2.05)	0.852
α-agonist						
Past user	0 (0 to Inf)	0.992	0 (0 to Inf)	0.995	0 (0 to Inf)	0.992
Thiazide						
Past user	0 (0 to Inf)	0.993	0 (0 to Inf)	0.995	0 (0 to Inf)	0.993
Current user	0.69 (0.12 to 3.89)	0.844	0.66 (0.12 to 3.67)	0.79	0.64 (0.12 to 3.57)	0.764
Antiplatelet						
Past user	0.96 (0.44 to 2.11)	0.988	0.96 (0.44 to 2.11)	0.988	0.96 (0.44 to 2.12)	0.988
Antiarrhythmic						
Past user	0.44 (0.05 to 4.11)	0.519	0.47 (0.05 to 4.3)	0.56	0.53 (0.06 to 4.89)	0.688
Current user	2.14 (0.72 to 6.43)	0.288	2.02 (0.67 to 6.08)	0.302	2.1 (0.71 to 6.18)	0.296
Anticoagulant						
Past user	0.48 (0.05 to 4.78)	0.669	0.44 (0.04 to 4.48)	0.614	0.48 (0.05 to 4.94)	0.753
Current user	1.16 (0.31 to 4.39)	0.918	1.13 (0.3 to 4.35)	0.95	1.16 (0.31 to 4.31)	0.917
Glucocorticoid						
Past user	1.45 (0.43 to 4.9)	0.609	1.34 (0.4 to 4.49)	0.709	1.45 (0.43 to 4.88)	0.683
Current user	2.86 (1.28 to 6.4)	0.021	2.62 (1.17 to 5.86)	0.038	2.77 (1.23 to 6.24)	0.028
β2-agonist						
Past user	2.3 (0.37 to 14.3)	0.464	2.04 (0.32 to 13.1)	0.504	1.91 (0.31 to 11.8)	0.61
Current user	3.06 (1.3 to 7.22)	0.021	2.66 (1.15 to 6.15)	0.045	2.69 (1.16 to 6.2)	0.041
Muscarinic antagonist						
Past user	1.51 (0.3 to 7.58)	0.775	1.84 (0.35 to 9.65)	0.59	1.53 (0.31 to 7.5)	0.768
Current user	1.16 (0.46 to 2.89)	0.838	1.13 (0.45 to 2.84)	0.877	1.11 (0.45 to 2.75)	0.918
NSAID						
Past user	0.64 (0.12 to 3.39)	0.663	0.55 (0.1 to 2.94)	0.542	0.56 (0.11 to 2.92)	0.541
Current user	4.47 (1.28 to 15.6)	0.038	4.06 (1.15 to 14.3)	0.059	4.28 (1.24 to 14.7)	0.042
Vitamin D						
Past user	1.81 (0.54 to 6.09)	0.421	1.59 (0.48 to 5.25)	0.496	1.81 (0.55 to 5.98)	0.415
Current user	1.73 (0.76 to 3.93)	0.278	1.41 (0.62 to 3.17)	0.496	1.57 (0.7 to 3.51)	0.392
Proton pump inhibitor						
Past user	1.58 (0.3 to 8.28)	0.698	1.34 (0.26 to 6.98)	0.807	1.4 (0.28 to 7.17)	0.758
Current user	0.83 (0.38 to 1.8)	0.698	0.78 (0.36 to 1.7)	0.663	0.83 (0.38 to 1.82)	0.758
Statin						
Past user	0.64 (0.13 to 3.08)	0.719	0.7 (0.15 to 3.29)	0.729	0.79 (0.16 to 3.85)	0.952
Current user	1.23 (0.49 to 3.13)	0.731	1.29 (0.52 to 3.2)	0.719	1.44 (0.52 to 3.96)	0.684
Immunosuppressant						
Past user	1.39 (0.16 to 11.8)	0.847	1.23 (0.16 to 9.64)	0.936	1.24 (0.16 to 9.65)	0.93
Current user	2.37 (0.42 to 13.5)	0.473	2.33 (0.4 to 13.4)	0.429	2.34 (0.42 to 13.3)	0.479
**Complications post diagnosis (ref=No)**						
**Cardiovascular**						
Recurrent	2.41 (0.99 to 5.89)	0.107	2.44 (1 to 5.96)	0.101	2.5 (1.02 to 6.14)	0.091
Novel	0.38 (0.04 to 3.97)	0.465	0.49 (0.04 to 5.47)	0.627	0.44 (0.04 to 4.47)	0.604

**Respiratory**						
Recurrent	2.82 (0.92 to 8.71)	0.118	2.74 (0.9 to 8.38)	0.125	2.51 (0.83 to 7.6)	0.173
Novel	5.82 (1.75 to 19.3)	0.01	6.02 (1.8 to 20.2)	0.009	5.71 (1.73 to 18.9)	0.011
**Renal**						
Recurrent	1.67 (0.64 to 4.37)	0.419	1.33 (0.5 to 3.54)	0.716	1.68 (0.65 to 4.39)	0.408
Novel	1.08 (0.32 to 3.65)	0.988	0.75 (0.22 to 2.6)	0.722	0.98 (0.29 to 3.29)	0.988

	**Adjusted OR (+Cardiovascular) (95% CI)**	**P value**	**Adjusted OR (+Renal) (95% CI)**	**P value**	**Adjusted OR (+Respiratory) (95% CI)**	**P value**
**Demographics**						
Gender (ref=Female)						
Male	3.26 (1.57 to 7.13)	0.006	2.99 (1.44 to 6.47)	0.011	3.16 (1.54 to 6.8)	0.006
Ethnicity (ref=White)						
South Asian	1.8 (0.79 to 4.13)	0.207	1.73 (0.77 to 3.93)	0.248	1.85 (0.82 to 4.17)	0.218
Black	3.82 (1.45 to 10.4)	0.015	3.14 (1.18 to 8.61)	0.046	3.52 (1.34 to 9.51)	0.022
Other	0.23 (0.01 to 1.41)	0.207	0.26 (0.01 to 1.63)	0.263	0.26 (0.01 to 1.62)	0.305
Age group (ref=18-40)						
41-50	2.01 (0.16 to 47.4)	0.712	2.03 (0.17 to 47.6)	0.702	2.13 (0.18 to 49.8)	0.663
51-60	1.54 (0.13 to 35.9)	0.807	1.74 (0.15 to 40.5)	0.728	1.94 (0.16 to 45)	0.663
61-70	5.21 (0.77 to 105)	0.221	5.86 (0.87 to 118)	0.208	6.75 (1.03 to 135)	0.156
71-80	12.8 (2.07 to 252)	0.053	15.2 (2.36 to 305)	0.047	19.2 (3.19 to 373)	0.022
80+	16.7 (2.69 to 328)	0.035	20.5 (3.18 to 411)	0.031	26.8 (4.52 to 522)	0.011
**HPB disease (ref=No)**						
Cancer						
Yes	1.26 (0.13 to 12.2)	0.899	1.69 (0.18 to 15.5)	0.688	1.19 (0.14 to 10.1)	0.937
Pancreatic disease						
Acute	1.28 (0.36 to 4.59)	0.818	1.47 (0.42 to 5.14)	0.635	1.53 (0.44 to 5.29)	0.599
Chronic	2.97 (0.98 to 8.98)	0.134	3.11 (1.06 to 9.13)	0.118	3.22 (1.11 to 9.37)	0.096
Liver disease						
Mild	0.47 (0.17 to 1.29)	0.27	0.49 (0.18 to 1.3)	0.312	0.5 (0.19 to 1.33)	0.357
Moderate/Severe	0.53 (0.14 to 2.01)	0.482	0.66 (0.18 to 2.45)	0.635	0.6 (0.16 to 2.19)	0.592
Biliary disease						
Acute	3.25 (0.48 to 22.2)	0.36	2.53 (0.38 to 16.6)	0.501	2.56 (0.38 to 17)	0.552
Chronic	0.65 (0.25 to 1.71)	0.482	0.67 (0.26 to 1.7)	0.539	0.67 (0.26 to 1.7)	0.592
**Comorbidities (ref=No)**						
Diabetes	1.78 (0.76 to 4.19)	0.236	1.76 (0.75 to 4.13)	0.291	1.86 (0.79 to 4.38)	0.283
Hypertension	5.73 (0.62 to 52.9)	0.185	5.62 (0.64 to 49.5)	0.216	6.16 (0.72 to 52.7)	0.174
Cholesterol	1.05 (0.45 to 2.42)	0.989	1.21 (0.53 to 2.75)	0.731	1.26 (0.56 to 2.84)	0.719
Cardiovascular	2.82 (1.21 to 6.59)	0.026	2.6 (1.06 to 6.34)	0.065	3.01 (1.27 to 7.14)	0.022
Renal	1.28 (0.57 to 2.88)	0.623	1.72 (0.8 to 3.7)	0.248	1.73 (0.8 to 3.73)	0.271
Respiratory	0.72 (0.34 to 1.51)	0.428	0.83 (0.41 to 1.71)	0.695	0.85 (0.42 to 1.73)	0.743

**Lifestyle factors (ref=Never)**						
Smoker						
Past	1.75 (0.76 to 4.02)	0.269	1.79 (0.79 to 4.07)	0.298	1.92 (0.84 to 4.36)	0.22
Current	0.45 (0.08 to 2.48)	0.436	0.44 (0.08 to 2.48)	0.429	0.48 (0.08 to 2.73)	0.56
Drinker						
Past	1.09 (0.37 to 3.26)	0.958	1.27 (0.43 to 3.76)	0.783	1.19 (0.41 to 3.47)	0.879
Current	1.5 (0.55 to 4.09)	0.588	1.6 (0.59 to 4.33)	0.492	1.37 (0.52 to 3.61)	0.826
Substance user						
Past	0.33 (0.04 to 2.9)	0.437	0.48 (0.06 to 4.03)	0.61	0.65 (0.08 to 5.26)	0.843
Current	1.44 (0.36 to 5.87)	0.744	1.77 (0.45 to 6.91)	0.564	2.12 (0.56 to 8.07)	0.428
Obese						
Past	1.14 (0.44 to 2.96)	0.996	1.4 (0.55 to 3.52)	0.658	1.45 (0.58 to 3.63)	0.679
Current	1 (0.42 to 2.36)	0.996	1.05 (0.45 to 2.45)	0.991	1.03 (0.44 to 2.39)	0.991
**Prescription medication use (ref=Non-user)**						
ACE inhibitor						
Past user	0.93 (0.34 to 2.57)	0.989	0.92 (0.34 to 2.52)	0.967	1.02 (0.38 to 2.75)	0.988
Current user	2.25 (0.89 to 5.73)	0.146	2.07 (0.81 to 5.31)	0.26	2.32 (0.9 to 5.93)	0.16
Angiotensin receptor blocker						
Past user	0.86 (0.11 to 6.75)	0.982	1.03 (0.14 to 7.79)	0.988	1.14 (0.15 to 8.91)	0.988
Current user	0.9 (0.32 to 2.55)	0.982	0.84 (0.31 to 2.27)	0.915	0.85 (0.31 to 2.29)	0.928
Aldosterone agonist						
Past user	0.93 (0.2 to 4.24)	0.989	1.07 (0.23 to 4.96)	0.988	1.34 (0.29 to 6.13)	0.805
Current user	1.03 (0.22 to 4.77)	0.989	1.32 (0.28 to 6.08)	0.906	1.32 (0.28 to 6.19)	0.805
β-blocker						
Past user	0.44 (0.09 to 2.13)	0.341	0.55 (0.12 to 2.59)	0.502	0.69 (0.15 to 3.12)	0.699
Current user	0.38 (0.16 to 0.92)	0.053	0.54 (0.24 to 1.24)	0.208	0.64 (0.29 to 1.43)	0.396
Calcium channel blocker						
Past user	0.09 (0.01 to 0.56)	0.016	0.13 (0.02 to 0.75)	0.045	0.16 (0.03 to 0.91)	0.078
Current user	0.64 (0.27 to 1.55)	0.364	0.76 (0.32 to 1.8)	0.587	0.91 (0.39 to 2.12)	0.977
α-agonist						
Past user	0 (0 to Inf)	0.992	0 (0 to Inf)	0.992	0 (0 to Inf)	0.992
Thiazide						
Past user	0 (0 to Inf)	0.993	0 (0 to Inf)	0.993	0 (0 to Inf)	0.993
Current user	0.46 (0.08 to 2.63)	0.44	0.73 (0.13 to 3.97)	0.816	0.7 (0.13 to 3.93)	0.862
Antiplatelet						
Past user	0.7 (0.3 to 1.61)	0.44	0.87 (0.39 to 1.94)	0.816	1 (0.46 to 2.2)	0.993
Antiarrhythmic						
Past user	0.41 (0.04 to 3.95)	0.493	0.44 (0.05 to 4.14)	0.525	0.51 (0.05 to 4.69)	0.618
Current user	1.66 (0.55 to 5.01)	0.466	1.9 (0.64 to 5.68)	0.328	2.17 (0.73 to 6.46)	0.272
Anticoagulant						
Past user	0.37 (0.04 to 3.85)	0.506	0.47 (0.05 to 4.68)	0.651	0.48 (0.05 to 4.84)	0.76
Current user	0.96 (0.26 to 3.57)	0.989	1.1 (0.29 to 4.18)	0.982	1.19 (0.32 to 4.46)	0.881
Glucocorticoid						
Past user	1.54 (0.46 to 5.18)	0.537	1.56 (0.46 to 5.23)	0.524	1.54 (0.46 to 5.21)	0.538
Current user	2.97 (1.31 to 6.76)	0.018	2.82 (1.26 to 6.31)	0.024	3.21 (1.38 to 7.45)	0.013

β2-agonist						
Past user	2 (0.3 to 13.1)	0.523	1.86 (0.3 to 11.7)	0.563	3.7 (0.53 to 25.8)	0.25
Current user	2.54 (1.09 to 5.93)	0.052	2.69 (1.17 to 6.21)	0.041	4.8 (1.7 to 13.6)	0.008
Muscarinic antagonist						
Past user	1.67 (0.33 to 8.45)	0.671	1.61 (0.32 to 7.99)	0.703	1.56 (0.32 to 7.58)	0.766
Current user	1 (0.4 to 2.51)	0.998	1.03 (0.41 to 2.59)	0.988	1.19 (0.46 to 3.08)	0.796
NSAID						
Past user	0.64 (0.12 to 3.47)	0.676	0.65 (0.12 to 3.45)	0.683	0.6 (0.11 to 3.14)	0.643
Current user	3.81 (1.08 to 13.5)	0.063	4.66 (1.29 to 16.8)	0.047	4.23 (1.22 to 14.7)	0.046
Vitamin D						
Past user	1.52 (0.45 to 5.1)	0.552	1.67 (0.51 to 5.49)	0.449	1.96 (0.58 to 6.6)	0.344
Current user	1.38 (0.6 to 3.15)	0.552	1.42 (0.62 to 3.26)	0.449	1.75 (0.75 to 4.05)	0.344
Proton pump inhibitor						
Past user	1.05 (0.21 to 5.37)	0.989	1.23 (0.24 to 6.21)	0.894	1.39 (0.27 to 7.2)	0.838
Current user	0.61 (0.26 to 1.4)	0.306	0.78 (0.36 to 1.73)	0.685	0.88 (0.41 to 1.91)	0.838
Statin						
Past user	0.62 (0.13 to 3.04)	0.699	0.68 (0.14 to 3.28)	0.701	0.81 (0.18 to 3.74)	0.879
Current user	1.17 (0.47 to 2.95)	0.816	1.31 (0.52 to 3.27)	0.701	1.52 (0.62 to 3.69)	0.511
Immunosuppressant						
Past user	1.59 (0.21 to 11.9)	0.725	1.31 (0.16 to 10.5)	0.889	1.25 (0.16 to 9.91)	0.923
Current user	3.31 (0.59 to 18.5)	0.259	2.18 (0.38 to 12.6)	0.481	2.5 (0.45 to 13.9)	0.425
**Complications post diagnosis (ref=No)**						
**Cardiovascular**						
Recurrent	2.53 (1.04 to 6.12)	0.071	2.33 (0.92 to 5.88)	0.149	2.7 (1.1 to 6.68)	0.062
Novel	0.44 (0.04 to 4.47)	0.547	0.44 (0.04 to 4.52)	0.607	0.46 (0.05 to 4.65)	0.569
**Respiratory**						
Recurrent	2.24 (0.72 to 6.97)	0.205	2.49 (0.82 to 7.55)	0.179	2.53 (0.84 to 7.63)	0.15
Novel	6.17 (1.81 to 21)	0.009	5.85 (1.76 to 19.4)	0.01	5.77 (1.75 to 19)	0.009
**Renal**						
Recurrent	1.06 (0.37 to 3.02)	0.989	1.72 (0.66 to 4.46)	0.34	1.75 (0.67 to 4.57)	0.358
Novel	0.7 (0.2 to 2.51)	0.734	1 (0.3 to 3.34)	0.997	1.02 (0.3 to 3.44)	0.988

Odds ratios (ORs), except the crude ones, are mutually adjusted for gender, ethnicity, and age group, and also for additional conditions when mentioned inside the parenthesis. Dichotomous age groups (over and under 60) are used for controlling for all categories except demographics. All P values presented, except for the crude odds ratios, are Benjamini-Hochberg corrected.
