# Supplementary material for: COVID-19 in patients with hepatobiliary and pancreatic diseases: a single-centre cross-sectional study in East London
Source: BMJ Open. 2021 Apr 19;11(4):e045077. doi: 10.1136/bmjopen-2020-045077 (PMC8057071; doi:10.1136/bmjopen-2020-045077)
Supplement: Author's manuscript [file bmjopen-2020-045077.draft_revisions.pdf]

BMJ Open is committed to open peer review. As part of this commitment we make the peer review history of every article we publish publicly available.

When an article is published we post the peer reviewers' comments and the authors' responses online. We also post the versions of the paper that were used during peer review. These are the versions that the peer review comments apply to.

The versions of the paper that follow are the versions that were submitted during the peer review process. They are not the versions of record or the final published versions. They should not be cited or distributed as the published version of this manuscript.

BMJ Open is an open access journal and the full, final, typeset and author-corrected version of record of the manuscript is available on our site with no access controls, subscription charges or pay-per-view fees (http://bmjopen.bmj.com).

If you have any questions on BMJ Open's open peer review process please email info.bmjopen@bmj.com

BMJ Open

COVID-19 in patients with hepatobiliary and pancreatic diseases: A single-centre cohort study in East London

Journal:	*BMJ Open*
Manuscript ID	bmjopen-2020-045077
Article Type:	Original research
Date Submitted by the Author:	22-Sep-2020
Complete List of Authors:	Dayem Ullah, Abu; Barts Cancer Institute, Centre for Cancer Biomarker and Biotherapeutics; Barts Health NHS Trust<br>Kocher, Hemant ; Barts Cancer Institute, Centre for Tumour Biology; Barts Health NHS Trust<br>Chelala, Claude; Barts Cancer Institute, Centre for Cancer Biomarker and Biotherapeutics<br>Sivapalan, Lavanya; Barts Cancer Institute, Centre for Cancer Biomarker and Biotherapeutics
Keywords:	Hepatobiliary disease < GASTROENTEROLOGY, Pancreatic disease < GASTROENTEROLOGY, COVID-19

SCHOLARONE™
Manuscripts

I, the Submitting Author has the right to grant and does grant on behalf of all authors of the Work (as defined in the below author licence), an exclusive licence and/or a non-exclusive licence for contributions from authors who are: i) UK Crown employees; ii) where BMJ has agreed a CC-BY licence shall apply, and/or iii) in accordance with the terms applicable for US Federal Government officers or employees acting as part of their official duties; on a worldwide, perpetual, irrevocable, royalty-free basis to BMJ Publishing Group Ltd ("BMJ") its licensees and where the relevant Journal is co-owned by BMJ to the co-owners of the Journal, to publish the Work in this journal and any other BMJ products and to exploit all rights, as set out in our licence.

The Submitting Author accepts and understands that any supply made under these terms is made by BMJ to the Submitting Author unless you are acting as an employee on behalf of your employer or a postgraduate student of an affiliated institution which is paying any applicable article publishing charge ("APC") for Open Access articles. Where the Submitting Author wishes to make the Work available on an Open Access basis (and intends to pay the relevant APC), the terms of reuse of such Open Access shall be governed by a Creative Commons licence – details of these licences and which Creative Commons licence will apply to this Work are set out in our licence referred to above.

Other than as permitted in any relevant BMJ Author's Self Archiving Policies, I confirm this Work has not been accepted for publication elsewhere, is not being considered for publication elsewhere and does not duplicate material already published. I confirm all authors consent to publication of this Work and authorise the granting of this licence.

For peer review only

COVID-19 in patients with hepatobiliary and pancreatic diseases: A single-centre cohort study in East London

Abu Z M Dayem Ullah, UKRI/Rutherford research fellow[1 2], Hemant M Kocher, professor of liver and pancreas surgery[1 2], Claude Chelala, professor of bioinformatics[1 3], Lavanya Sivapalan, PhD student[1],

Author affiliations

[1]Barts Cancer Institute, Queen Mary University of London, London, UK
[2]Barts Health NHS Trust, London, UK
[3]Life Sciences Institute, Queen Mary University of London, London, UK

Correspondence to:

Abu Z M Dayem Ullah
Barts Cancer Institute, Queen Mary University of London, London EC1M 6BQ, UK
Email: d.ullah@qmul.ac.uk

For peer review only - http://bmjopen.bmj.com/site/about/guidelines.xhtml

ABSTRACT

Objective To explore risk factors associated with COVID-19 susceptibility and survival in patients with pre-existing hepato-pancreato-biliary (HPB) conditions.

Design Retrospective cohort study.

Setting East London Pancreatic Cancer Epidemiology (EL-PaC-Epidem) study at Barts Health NHS Trust, UK. Linked electronic health records were interrogated on a cohort of participants (age ≥ 18 years), diagnosed or reported with at least one HPB condition between April 2008 and 6 March 2020. The censored data collection date was 12 June 2020.

Participants EL-PaC-Epidem study participants, alive on 12 February 2020, and living in East London within the previous six months (n=15 586). The cohort represents a diverse multi-ethnic population with 51.6% belonging to the non-White background.

Main outcome measure COVID-19 incidence and mortality.

Results Some 212 (1.4%) participants had confirmed COVID-19 diagnosis, with an increased risk for men (RR 1.59; 95% CI 1.21 to 2.09) and Black ethnicity (2.2; 95% CI 1.5 to 3.18) amongst demographic features. Each additional comorbidity increased the risk of infection by 60%. Substance mis-users were at more risk of infection, so were patients on Vitamin D treatment. The higher risks associated with South Asian ethnicity, patients with pre-existing non-malignant pancreatic or liver conditions, age>70, and past smokers were due to co-existing comorbidities. Surprisingly, current smokers were associated with a lower risk. Increased mortality risk was observed for Black ethnicity (2.4; 95% CI 1.35 to 3.48), and patients with a pre-existing kidney condition - particularly when accompanied with an acute episode of renal complications (2.74; 95% CI 1.32 to 5.13).

Conclusions In this large population-based study of HPB patients, male gender, Black ethnicity, medical co-morbidities, substance mis-use, and a history of Vitamin D treatment independently posed a higher risk of acquiring COVID-19. Particular attention should be paid to patients with a pre-existing kidney disease for further renal insult to prevent fatality.

For peer review only - http://bmjopen.bmj.com/site/about/guidelines.xhtml

STRENGTHS AND LIMITATIONS OF THIS STUDY

- First multi-ethnic population-based study on COVID-19 in the hepato-pancreato-biliary group of diseases as a whole.
- Systematic identification of the effect, or the lack of it, of individual demographic and clinical factors on the infection and mortality of COVID-19 in a large cohort of over 15000 patients, robustly corrected for potential confounders in their evaluation.
- Access to longitudinal data from linked primary and secondary care electronic health records, and use of rule-based phenotyping algorithms allowed for improved completeness and correctness of the explored variables.
- Despite the reasonable measures for identifying all cases with confirmed COVID-19 in the study cohort, some could still be missed in the less likely event of diagnosis outside the study site.
- The effects reported in the study could be influenced by the relatively smaller size of COVID-19 cases within the large cohort.

For peer review only - http://bmjopen.bmj.com/site/about/guidelines.xhtml

INTRODUCTION

COVID-19 is a novel infectious disease caused by severe acute respiratory syndrome coronavirus 2 (SARS-CoV-2), with a wide-ranging disease course. Infection and mortality rates of the COVID-19 pandemic have varied widely among nations and demographics,[1] while risks are still being explored, identified and categorised according to the severity.[2 3] There are several confirmed risk factors of COVID-19 and severe outcomes, including old age,[2 4 5] chronic pulmonary disease,[2 4-6] cardiovascular disease,[2 5 6] hypertension,[5] chronic kidney disease,[2 4 6] diabetes mellitus,[2 5] obesity,[2 6 7] haematological diseases,[2 4] malignancy,[2 4-6 8] and immuno-compromised state such as HIV infection.[2 4 9] Medical complications following hospitalisation, including acute episodes of cardiovascular, respiratory, neurological, renal, or hepatic failure, have also been linked to severe outcomes.[10] There are also other risk factors reported, such as smoking[11 12] or being from a Black, Asian and minority ethnic (BAME) group,[13-15] the effects of which are either mixed or the reasoning is not clearly understood.[4] Concerns have also been raised regarding the use of various medications with respect to the risk or protective effect to COVID-19.[16-18]

Patients with diseases of the liver, pancreas or biliary tract (hepato-pancreato-biliary; HPB) are considered, in general, to be at risk of developing serious medical conditions. Expression of the ACE2 gene – a receptor for the SARS-CoV-2 virus - along the gastrointestinal tract is well documented, which suggests the digestive system is a potential route for COVID-19,[18] making patients with a diseased HPB system susceptible to this novel infection. The prevalence of COVID-19 among patients with hepatic conditions has been explored,[6 15 19] indicating severe liver disease as a moderate risk factor for COVID-19.[2] In contrast, very limited data is available on the prevalence of COVID-19 among patients with pancreatic or biliary conditions,[20] although pancreatic manifestations of the disease are rare.[21 22] It is important that clinical characteristics of COVID-19 are investigated for the HPB group as a whole, not only because these diseases demonstrate similar clinical-biologic behaviours,[23] but also since they are commonly seen by a single clinical unit with specialist expertise in the management of these diseases.

The United Kingdom (UK) has been the worst affected country in Europe by COVID-19, with a reported death toll of 44819 as of 30 June 2020.[24] At the same time, London had the highest incidence and mortality rates, with 33775 confirmed cases and 8438 deaths.[25 26] Barts Health NHS Trust (BHNT) is the largest National Health Service (NHS) Trust in England and acts as provider of district general hospital facilities for around 2.5 million population of East London as well as a range of tertiary care services.[27] Between March 1 and June 30, the three boroughs in East London - Tower Hamlets, Waltham Forest and Newham - had a combined age-standardised COVID-19 related mortality rate of 195 per 100 000 people. This was significantly higher than the rest of London where the age-standardised COVID-19 related mortality rate was 156 per 100 000 people.[25] East London is also one of the most ethnically diverse local areas in the country where an estimated 57% residents belong to a BAME group.[28] Significant health inequalities exist within the local population including higher rates of cancer, diabetes and obesity,[29] compared to the wider population. These conditions are not only known to be a precursor or consequence to HPB diseases, but also linked to COVID-19 and severe outcomes. In this study, we integrated primary, secondary and tertiary electronic healthcare records (EHRs) of HPB patients in East London. We

For peer review only - http://bmjopen.bmj.com/site/about/guidelines.xhtml

inspected the demographics, lifestyle, comorbidities and associated medication use of these patients, and any possible links with SARS-CoV-2 infection. We also evaluated whether the effect of these prevalent factors as well as clinical observations during COVID-19 related hospitalisation are associated with mortality. This study will inform the management of this specific cohort of patients.

For peer review only

METHODS

Study setting and data sources

All data utilised for this study were collected and processed under the East London Pancreatic Cancer Epidemiology (EL-PaC-Epidem) study at BHNT. In brief, EL-PaC-Epidem is an ongoing study that ascertains patients diagnosed or reported with HPB diseases including cancers, as well as control patients (e.g., small intestine, hernia), within five BHNT hospital sites (The Royal London Hospital, Newham University Hospital, St Bartholomew's Hospital, Whipps Cross University Hospital, Mile End Hospital) between 2008 and 2021. The EL-PaC-Epidem study was approved by the East of England - Essex Research Ethics Committee (19/EE/0163; 17 May 2019) and supported by the NHS Confidentiality Advisory Group for collecting and processing confidential patient information without consent (19/CAG/0219; 17 January 2020). The study is limited to the secondary use of a specified subset of patients' retrospective EHR generated during the course of normal care of these patients. It links EHRs from different data sources (via UK unique individual NHS numbers), including primary care through General Practitioners (GP) (Discovery East London Programme data service [DDS]) and secondary or tertiary care through hospitals (BHNT Consolidated Data Extract [CDE]). Patients, who have previously informed their GPs or NHS to stop sharing their personal and health records for purposes other than their individual care, were automatically excluded. The current EL-PaC-Epidem study cohort consists of 27324 adult patients (aged 18 years or over), diagnosed or reported with at least one of the HPB conditions (supplemental table 1) between April 2008 and 6 March 2020. The censored data collection date for this cohort was 12 June 2020.

Study design and population

This is a retrospective single-centre cohort study utilising the linked EHR data of patients with a history of HPB diseases. Within this specific patient group, the study focused on the incidence of COVID-19, and examined the association of SARS-CoV-2 infection with major comorbidities, lifestyle factors, and use of medication.

As the first case of COVID-19 in London was reported on 12 February 2020, we used this as the start date for this study and extracted data on a subgroup of the EL-PaC-Epidem study cohort until 12 June 2020 (figure 1). Eligible individuals were a resident in East London and alive on the study start date (EL-HPB). Residency of East London was inferred if a patient had at least one appointment or prescription issued from a GP in East London boroughs or had a scheduled or unscheduled visit to one of the BHNT hospitals within the last six months (after 12 August 2019). Patients with *confirmed* SARS-CoV-2 infection were identified by: i) the presence of International Classification of Diseases 10th edition (ICD-10) or Systematized Nomenclature of Medicine Clinical Terms (SNOMED CT) codes for confirmed COVID-19 or SARS diagnosis assigned in their hospital encounters during the observation period between February 12 and June 12, 2020 (supplemental table 2) OR ii) positive record of SARS-CoV-2 RNA through BHNT oral and/or nasal swabs test during the same period. For confirmed COVID-19 cases, the earliest date of diagnosis or positive swab test was considered as the *index date,* whereas 12 February 2020 was considered as *index date* for rest of the cohort. Patients, who were assigned an ICD-10 diagnosis code for *suspected* COVID-19 but

For peer review only - http://bmjopen.bmj.com/site/about/guidelines.xhtml

were neither reassigned to confirmed diagnosis nor positive RNA test, were not considered as COVID-19 patients.

We also examined the onset-to-death distribution within the patient group with a confirmed COVID-19 diagnosis (EL-HPB-COVID). The death of a patient with a confirmed COVID-19 diagnosis within the observation period is considered as a COVID-19 related death. The onset-to-death distribution was analysed in the context of collated comorbidities, lifestyle and regular medication use, as well as medical complications during hospital care.

<<Figure 1 here>>

Figure 1 Selection of patients for the retrospective cohort study.

Procedures

All patient data were obtained from retrospective EHR, harmonised across hospital and GP coding systems where applicable, and organised into 38 primary variables across six categories corresponding to the focus of the study (table 1). BHNT CDE uses 2011 UK census grouping to record ethnicity, ICD-10 or SNOMED diagnosis codes for clinically relevant diagnoses, and Office of the Population Censuses and Surveys Classification of Interventions and Procedures version 4 (OPCS-4) procedural codes for treatments and procedures. Physiological observations (weight, body-mass index [BMI], blood pressure) and laboratory tests results are available in locally developed terms. Free text entries such as discharge summaries, past medical history and a lifestyle questionnaire collected during the pre-operative assessment, and presenting symptoms from scheduled or unscheduled hospital visits are also available. All GP records via DDS were available in Read Codes v2 or Clinical Terminology Version 3 (CTV3) codes, except the prescribed medication records which were available in SNOMED codes. For each variable, we consulted ICD-10, SNOMED, Read, CTV3 or OPCS-4 dictionaries as appropriate to construct the mapping *codelists.* For some variables, codelists also included keywords to search for within free text as well as local laboratory test and physiological observation terms.

Rule-based phenotyping algorithms were developed for each categorical variable to characterise patients, integrating information from multiple sources where available to counteract bias. HPB diseases were grouped into four categories (supplemental table 1): *any* malignant disease, and non-malignant diseases of liver, pancreas or biliary tract. A patient can either be assigned to a malignant disease category or any of the non-malignant disease categories. Patients with non-malignant diagnoses for multiple organs were represented in all the respective non-malignant categories. Ethnicity was grouped into four categories - White, South Asian, Black, and Other. White and Black ethnic groups were defined based on the 2011 UK census classification; Indian, Pakistani and Bangladeshi origin from the Asian group represented South Asian, while the rest (i.e., Mixed, Chinese, other Asian and other ethnic group) were represented in the Other group. The ethnic category recorded at the GP took precedence over hospital records.

For peer review only - http://bmjopen.bmj.com/site/about/guidelines.xhtml

Table 1 Variables and outcomes explored in this study.

Category	Variables	Levels/Units
Demographic	Gender	Female, Male
	Ethnicity	White, South Asian, Black, Other [, Not available]
	Age (continuous)	years
	Age group*	18-40, 41-50, 51-60, 61-70, 71-80, 80+
	Binary age group*	18-60, 60+
	HPB diagnosis	Cancer, Pancreatic disease, Liver disease, Biliary disease
Comorbidity	Diabetes	No, Yes
	Hypertension	No, Yes
	High cholesterol	No, Yes
	Cardiovascular disease	No, Yes
	Respiratory disease	No, Yes
	Kidney disease	No, Yes
	Number of comorbidities*	None, 1, 2, 3 or more
Lifestyle factors	Smoker	Never, Past, Current [, Not available]
	Alcohol drinker	Never, Past, Current [, Not available]
	Substance user	Never, Past, Current [, Not available]
	Obese	Never, Past, Current [, Not available]
Medication use	Angiotensin-converting enzyme [ACE] inhibitors	Non-, Past, Current user
	Angiotensin II receptor blockers inhibitors [ARBs]	Non-, Past, Current user
	Aldosterone antagonists [MCRA]	Non-, Past, Current user
	Beta-adrenergic blocking agents [β-blockers]	Non-, Past, Current user
	Calcium channel blockers [CCBs]	Non-, Past, Current user
	Alpha agonist	Non-, Past, Current user
	Thiazide	Non-, Past, Current user
	Antiplatelet	Non-, Past, Current user
	Antiarrhythmic	Non-, Past, Current user
	Anticoagulant	Non-, Past, Current user
	Glucocorticoid	Non-, Past, Current user
	Beta-2 adrenergic receptor agonists [β_2-agonists]	Non-, Past, Current user
	Muscarinic antagonist	Non-, Past, Current user
	Non-steroidal inflammatory drugs [NSAIDs]	Non-, Past, Current user
	Vitamin D	Non-, Past, Current user
	Proton pump inhibitors [PPIs]	Non-, Past, Current user
	Statin	Non-, Past, Current user
	Immunosuppressant	Non-, Past, Current user
Complications	Cardiovascular	No, Recurrent, Novel
	Respiratory	No, Recurrent, Novel
	Renal	No, Recurrent, Novel
	Number of recurrent complications*	None, 1, 2, 3
	Number of novel complications*	None, 1, 2, 3
Outcome	COVID-19 incidence	non-COVID-19, COVID-19
	COVID-19 mortality	Survivor, Deceased

All variables are categorical, unless otherwise stated. For categorical variables, the first value represents the reference level. Each HPB diagnosis groups are independent binary categorical variables. * Derived variables.

For peer review only

For peer review only - http://bmjopen.bmj.com/site/about/guidelines.xhtml

Phenotyping algorithms defining the comorbidities were based on diagnosis codes (presence) or free text search (presence or absence), with the additional inclusion of procedural codes (presence), some observation or laboratory test results (presence) and related medication use (at least three prescriptions). Patients were considered to have or have had a specific medical condition if they met at least one criterion indicating the presence of the condition before the *index date*, otherwise they were considered negative for the condition.

Phenotyping algorithms defining the lifestyle factors were based on the longitudinal entries (current, past or never) derived from diagnosis codes and free text search, with the additional inclusion of BMI observation for obesity. Obesity was defined as BMI of 30 kg/m^2 or more. Patients assigned *never* status at any point but having a record of *current* or *past* status before that date were reassigned to *past* status. The most recent lifestyle record before or on the *index date* was then used to assign *current*, *past* or *never* status to the patients. Patients with no record of a specific lifestyle factor were classified as having missing dataPatients were assigned *current*, *past* or *non-user* status for medication use variables based on the number of GP prescriptions issued in the last two years for the medicines under specific medication groups. Patients with no record of prescription for particular medications were assigned *non-user* status. With at least three prescriptions issued, a patient was assigned *current user* status if the latest issue was within three months preceding the *index date,* and *past user* status otherwise. Patients with record of less than three prescriptions were classified as *non-user.* Patients with COVID-19 were considered to have a specific complication during admitted patient care if at least one of the hospital diagnosis codes from the complications *codelist* was recorded during the observation period after *index date*, otherwise they were considered negative for the complication. A patient was considered to have a *recurrent* complication if they had a history of that particular comorbidity, otherwise it was considered as a *novel* complication.

Selection of study variables, *codelist* construction, and phenotyping algorithm development were done in consultation with a panel of clinicians and scientists (HMK, CC, LS). A comprehensive list of codelists and phenotyping algorithms for the study variables are available on the EL-PaC-Epidem portal.

Statistical analysis

We conducted descriptive analyses for the EL-HPB cohort as a whole, by group for patients with confirmed SARS-CoV-2 infection and the rest (herein referred to as COVID-19 and non-COVID-19 respectively). Differences in demographic and clinical characteristics between the groups were assessed with Pearson's Chi-square test, Fisher’s Exact test and Kruskal-Wallis rank sum test, as appropriate. P values less than 0.05 were considered significant. Similar descriptive analyses were performed for the EL-HPB-COVID cohort, and by survivor and deceased groups.

To explore the risk factors associated with COVID-19 susceptibility and subsequent survival, the effect size for each variable under investigation was evaluated with risk ratios (RRs) with 95% confidence intervals (CI), using regression models with a binomial distribution and log link function. Crude RRs were obtained from univariable regression models, and then simultaneously adjusted for a fixed set of potential confounders (gender, ethnicity, age group, HPB diagnoses) using multivariable

For peer review only - http://bmjopen.bmj.com/site/about/guidelines.xhtml

regression models with Benjamini-Hochberg correction for P values adjustment. The median age of the overall EL-HPB cohort being 57, a simplified binary age grouping (18-60, 61+) was used in multivariable regression models for comorbidity, lifestyle, medication use and post-diagnosis complication analyses. We also conducted more in depth post hoc analysis for demographic, lifestyle and medication categories by adding comorbidity covariates individually.

Patients with missing data for individual variables were included in the descriptive analyses but were automatically excluded in regression models for effect estimation. All statistical analyses and visualisations were performed in R (version 3.5.1).

Patient and public involvement

Patients and the public were involved in evaluating the design of the umbrella study (EL-PaC-Epidem), particularly the notion of collection and processing of retrospective patient data without their consent. The support from NHS Confidentiality Advisory Group was obtained based on the positive opinion posed by patient and the public.

peer review only

RESULTS

Population characteristics

The final EL-HPB cohort consisted of 15 586 patients, after applying the eligibility criteria and excluding 22 suspected but unconfirmed COVID-19 cases. By 12 June 2020, 212 (1.4%) confirmed cases of COVID-19 were reported in this cohort (figure 1). More than half of the COVID-19 cases had some form of liver disease (n=122, 57.5%); however, when comparing confirmed COVID-19 cases with the non-COVID-19 cases, we observed a disproportionate infection frequency in patients with pancreatic conditions (P=0.016). We also observed differences in gender, ethnic origin, and age group between COVID and non-COVID-19 cases (table 2). The proportion of males was significantly higher in the COVID-19 group compared to the baseline non-COVID-19 group (55.2% *vs* 43.7%, P<0.001). The same trend was observed for Black (18.4% vs 10.7%) and South Asian population (32.1% vs 28.7%). COVID-19 patients were older than non-COVID-19 patients (median 66.7 years, interquartile range 54.2 to 80.5 years *vs* 57.1 years, 44.8 to 69.2 years, P<0.001), with a steady increase in infection frequency with age. 79% of COVID-19 patients had three or more comorbidities, with hypertension being the most common comorbidity (86.3%), followed by high cholesterol and diabetes (table 2). Only five COVID-19 patients had no additional comorbidities. In general, COVID-19 patients had a higher rate of history (current or past) of smoking, drinking, substance mis-use and obesity compared to the non-COVID-19 group. Consistent with the underlying prevalent comorbidities of the COVID-19 group, history of prescription drugs use associated with managing hypertension or cardiovascular disease (ACE inhibitor, calcium channel blocker, β-blocker, aldosterone antagonists, antiplatelet, anticoagulant), cholesterol (statin), inflammation (glucocorticoid, β2-agonists) or background HPB condition (proton pump inhibitor) were higher in COVID-19 patients (table 2). Intake of vitamin D was also higher in COVID-19 patients.

As of 12 June, 54 (25.5%) of the 212 patients with COVID-19 had died; the death rate in the non-COVID-19 group during the same period was 1.7%. When analysing the 54 deceased and 158 surviving patients with confirmed SARS-CoV-2 infection, we found no differences in gender, but deceased patients were older than the survivors (median 77 years, interquartile range 66.4 to 82.5 years *vs* 63.5 years, 50 to 78.1 years; P<0.001) with steady increase in death with age becoming prominent in those above 70 years of age. The death rate was higher amongst Black (27.8% vs 15.2%) and South Asian (37% vs 30.4%) populations. The median survival period for the deceased patients from the date of confirmed COVID-19 diagnosis was 16 days (interquartile range 10 to 25.7 years). Kidney, hypertension, diabetes and cardiovascular conditions were observed to be associated with mortality in COVID-19 patients. 96.3% of deceased patients had at least three additional comorbidities, compared to 73.4% of patients who survived. A history of smoking was also associated with death (P=0.014). No overall differences were observed for other lifestyle factors, although half of the deceased were current substance mis-users. Notable differences were observed in the use of ACE inhibitors, vitamin D, statins, and antiplatelet medications. The frequency of renal complications was significantly higher in the deceased group compared to survivors (87% vs 58.2%, P<0.001).

For peer review only - http://bmjopen.bmj.com/site/about/guidelines.xhtml

Table 2 Differences in demographic, comorbidity, lifestyle, and medication use characteristics between COVID-19 infected and non-COVID-19 groups.

	non-COVID-19 (n=15 374)	COVID-19 (n=212)	Total (n=15 586)	P value
Demographics				
**Gender**				<0.001
Female	8651 (56.3%)	95 (44.8%)	8746 (56.1%)	
Male	6723 (43.7%)	117 (55.2%)	6840 (43.9%)	
**Ethnic origin**				<0.001
Not available	444 (2.9%)	2 (0.9%)	446 (2.9%	
White	7001 (45.5%)	87 (41.0%)	7088 (45.5%)	
South Asian	4407 (28.7%)	68 (32.1%)	4475 (28.7%)	
Black	1647 (10.7%)	39 (18.4%)	1686 (10.8%)	
Other	1875 (12.2%)	16 (7.5%)	1891 (12.1%)	
**HPB diagnosis***				
Cancer	422 (2.7%)	3 (1.4%)	425 (2.7%)	0.291
Non-cancer				
Pancreatic disease	2583 (16.8%)	49 (23.1%)	2632 (16.9%)	0.016
Liver disease	8098 (52.7%)	122 (57.5%)	8220 (52.7%)	0.152
Biliary disease	7331 (47.7%)	98 (46.2%)	7429 (47.7%)	0.676
**Age**				<0.001
Median	57.07	66.68	57.18	
IQR	44.75, 69.21	54.21, 80.51	44.85, 69.42	
**Age group**				<0.001
18-40	2837 (18.5%)	21 (9.9%)	2858 (18.3%)	
41-50	2743 (17.8%)	26 (12.3%)	2769 (17.8%)	
51-60	3439 (22.4%)	35 (16.5%)	3474 (22.3%)	
61-70	2981 (19.4%)	35 (16.5%)	3016 (19.4%)	
71-80	2000 (13.0%)	43 (20.3%)	2043 (13.1%)	
80+	1374 (8.9%)	52 (24.5%)	1426 (9.1%)	
**Mortality**				<0.001
Deceased	268 (1.7%)	54 (25.5%)	322 (2.1%)	
Survived	15106 (98.3%)	158 (74.5%)	15264 (97.9%)	
Comorbidities				
**Hypertension**	9984 (64.9%)	183 (86.3%)	10167 (65.2%)	<0.001
**Cholesterol**	8070 (52.5%)	150 (70.8%)	8220 (52.7%)	<0.001
**Diabetes**	5906 (38.4%)	141 (66.5%)	6047 (38.8%)	<0.001
**Cardiovascular**	4304 (28.0%)	117 (55.2%)	4421 (28.4%)	<0.001
**Kidney**	4014 (26.1%)	114 (53.8%)	4128 (26.5%)	<0.001
**Respiratory**	5010 (32.6%)	112 (52.8%)	5122 (32.9%)	<0.001
**Number of comorbidities**				<0.001
None	2259 (14.7%)	5 (2.4%)	2264 (14.5%)	
1	2943 (19.1%)	12 (5.7%)	2955 (19.0%)	
2	2984 (19.4%)	27 (12.7%)	3011 (19.3%)	
3 or more	7188 (46.8%)	168 (79.2%)	7356 (47.2%)	
Lifestyle factors				
**Smoker**				<0.001
Not available	896 (5.8%)	6 (2.8%)	902 (5.8%)	
Never	6459 (42.0%)	80 (37.7%)	6539 (42.0%)	
Past	4751 (30.9%)	102 (48.1%)	4853 (31.1%)	
Current	3268 (21.3%)	24 (11.3%)	3292 (21.1%)	

For peer review only

	non-COVID-19 (n=15 374)	COVID-19 (n=212)	Total (n=15 586)	P value
**Drinker**				0.007
Not available	2888 (18.8%)	29 (13.7%)	2917 (18.7%)	
Never	3755 (24.4%)	49 (23.1%)	3804 (24.4%)	
Past	2140 (13.9%)	46 (21.7%)	2186 (14.0%)	
Current	6591 (42.9%)	88 (41.5%)	6679 (42.9%)	
**Substance user**				<0.001
Not available	7784 (50.6%)	95 (44.8%)	7879 (50.6%)	
Never	3785 (24.6%)	29 (13.7%)	3814 (24.5%)	
Past	404 (2.6%)	13 (6.1%)	417 (2.7%)	
Current	3401 (22.1%)	75 (35.4%)	3476 (22.3%)	
**Obese**				0.01
Not available	645 (4.2%)	2 (0.9%)	647 (4.2%)	
Never	2327 (15.1%)	22 (10.4%)	2349 (15.1%)	
Past	6594 (42.9%)	107 (50.5%)	6701 (43.0%)	
Current	5808 (37.8%)	81 (38.2%)	5889 (37.8%)	
Prescription medication use				
**ACE inhibitor**				<0.001
Non-user	12649 (82.3%)	159 (75.0%)	12808 (82.2%)	
Past user	582 (3.8%)	24 (11.3%)	606 (3.9%)	
Current user	2143 (13.9%)	29 (13.7%)	2172 (13.9%)	
**Angiotensin receptor blocker**				0.08
Non-user	13903 (90.4%)	182 (85.8%)	14085 (90.4%)	
Past user	256 (1.7%)	5 (2.4%)	261 (1.7%)	
Current user	1215 (7.9%)	25 (11.8%)	1240 (8.0%)	
**Aldosterone agonist**				0.001
Non-user	14825 (96.4%)	196 (92.5%)	15021 (96.4%)	
Past user	156 (1.0%)	7 (3.3%)	163 (1.0%)	
Current user	393 (2.6%)	9 (4.2%)	402 (2.6%)	
**β-blocker**				<0.001
Non-user	12821 (83.4%)	148 (69.8%)	12969 (83.2%)	
Past user	501 (3.3%)	13 (6.1%)	514 (3.3%)	
Current user	2052 (13.3%)	51 (24.1%)	2103 (13.5%)	
**Calcium channel blocker**				0.023
Non-user	12332 (80.2%)	158 (74.5%)	12490 (80.1%)	
Past user	628 (4.1%)	16 (7.5%)	644 (4.1%)	
Current user	2414 (15.7%)	38 (17.9%)	2452 (15.7%)	
**Alpha agonist**				0.587
Non-user	15297 (99.5%)	212 (100.0%)	15509 (99.5%)	
Past user	22 (0.1%)	0 (0.0%)	22 (0.1%)	
Current user	55 (0.4%)	0 (0.0%)	55 (0.4%)	
**Thiazide**				0.728
Non-user	15297 (99.5%)	211 (99.5%)	15508 (99.5%)	
Past user	31 (0.2%)	0 (0.0%)	31 (0.2%)	
Current user	46 (0.3%)	1 (0.5%)	47 (0.3%)	
**Antiplatelet**				<0.001
Non-user	13261 (86.3%)	151 (71.2%)	13412 (86.1%)	
Past user	495 (3.2%)	18 (8.5%)	513 (3.3%)	
Current user	1618 (10.5%)	43 (20.3%)	1661 (10.7%)	
**Antiarrhythmic**				0.049
Non-user	15208 (98.9%)	206 (97.2%)	15414 (98.9%)	
Past user	47 (0.3%)	2 (0.9%)	49 (0.3%)	
Current user	119 (0.8%)	4 (1.9%)	123 (0.8%)	
**Anticoagulant**				0.005
Non-user	14837 (96.5%)	196 (92.5%)	15033 (96.5%)	
Past user	140 (0.9%)	5 (2.4%)	145 (0.9%)	

For peer review only - http://bmjopen.bmj.com/site/about/guidelines.xhtml

	non-COVID-19 (n=15 374)	COVID-19 (*N*=212)	Total (n=15 586)	P value
Current user	397 (2.6%)	11 (5.2%)	408 (2.6%)	
**Glucocorticoid**				<0.001
Non-user	11437 (74.4%)	122 (57.5%)	11559 (74.2%)	
Past user	1259 (8.2%)	22 (10.4%)	1281 (8.2%)	
Current user	2678 (17.4%)	68 (32.1%)	2746 (17.6%)	
**β2-agonist**				<0.001
Non-user	13734 (89.3%)	167 (78.8%)	13901 (89.2%)	
Past user	284 (1.8%)	8 (3.8%)	292 (1.9%)	
Current user	1356 (8.8%)	37 (17.5%)	1393 (8.9%)	
**Muscarinic antagonist**				<0.001
Non-user	13860 (90.2%)	170 (80.2%)	14030 (90.0%)	
Past user	318 (2.1%)	9 (4.2%)	327 (2.1%)	
Current user	1196 (7.8%)	33 (15.6%)	1229 (7.9%)	
**NSAID**				0.139
Non-user	13928 (90.6%)	185 (87.3%)	14113 (90.5%)	
Past user	724 (4.7%)	11 (5.2%)	735 (4.7%)	
Current user	722 (4.7%)	16 (7.5%)	738 (4.7%)	
**Vitamin D**				<0.001
Non-user	13137 (85.4%)	144 (67.9%)	13281 (85.2%)	
Past user	609 (4.0%)	20 (9.4%)	629 (4.0%)	
Current user	1628 (10.6%)	48 (22.6%)	1676 (10.8%)	
**Proton pump inhibitor**				<0.001
Non-user	9575 (62.3%)	103 (48.6%)	9678 (62.1%)	
Past user	1353 (8.8%)	22 (10.4%)	1375 (8.8%)	
Current user	4446 (28.9%)	87 (41.0%)	4533 (29.1%)	
**Statin**				<0.001
Non-user	10261 (66.7%)	104 (49.1%)	10365 (66.5%)	
Past user	853 (5.5%)	26 (12.3%)	879 (5.6%)	
Current user	4260 (27.7%)	82 (38.7%)	4342 (27.9%)	
**Immuno-suppressant**				0.044
Non-user	14929 (97.1%)	200 (94.3%)	15129 (97.1%)	
Past user	185 (1.2%)	4 (1.9%)	189 (1.2%)	
Current user	260 (1.7%)	8 (3.8%)	268 (1.7%)	

Values are n (%), unless otherwise specified. *Percentages total more than 100% because patients might have had non-malignant diseases in multiple organs.

For peer review only

Table 3 Differences in demographic, comorbidity, lifestyle, medication use, and post diagnosis complications characteristics between COVID-19 survivor and deceased groups.

	Survivor (n=158)	Deceased (n=54)	Total (n=212)	P value
Demographics				
**Gender**				0.207
Female	75 (47.5%)	20 (37.0%)	95 (44.8%)	
Male	83 (52.5%)	34 (63.0%)	117 (55.2%)	
**Ethnicity**				0.065
Not available	2 (1.3%)	0 (0.0%)	2 (0.9%)	
White	69 (43.7%)	18 (33.3%)	87 (41.0%)	
South Asian	48 (30.4%)	20 (37.0%)	68 (32.1%)	
Black	24 (15.2%)	15 (27.8%)	39 (18.4%)	
Other	15 (9.5%)	1 (1.9%)	16 (7.5%)	
**HPB diagnosis***				
Cancer	1 (0.6%)	2 (3.7%)	3 (1.4%)	0.160
Non-cancer				
Pancreatic disease	34 (21.5%)	15 (27.8%)	49 (23.1%)	0.355
Liver disease	95 (60.1%)	27 (50.0%)	122 (57.5%)	0.206
Biliary disease	68 (43.0%)	30 (55.6%)	98 (46.2%)	0.117
**Age**				<0.001
Median	63.55	76.99	66.81	
IQR	50.00, 78.06	66.44, 82.52	53.95, 80.65	
**Age group**				0.002
18-40	20 (12.7%)	2 (3.7%)	22 (10.4%)	
41-50	22 (13.9%)	3 (5.6%)	25 (11.8%)	
51-60	31 (19.6%)	4 (7.4%)	35 (16.5%)	
61-70	27 (17.1%)	8 (14.8%)	35 (16.5%)	
71-80	25 (15.8%)	17 (31.5%)	42 (19.8%)	
80+	33 (20.9%)	20 (37.0%)	53 (25.0%)	
**Survival/censoring**				< 0.001
Median	73	16	66	
IQR	62.00, 81.75	10.00, 25.75	30.75, 77.00	
Comorbidities				
**Hypertension**	130 (82.3%)	53 (98.1%)	183 (86.3%)	0.002
**Diabetes**	97 (61.4%)	44 (81.5%)	141 (66.5%)	0.007
**Cholesterol**	108 (68.4%)	42 (77.8%)	150 (70.8%)	0.227
**Kidney**	73 (46.2%)	41 (75.9%)	114 (53.8%)	<0.001
**Cardiovascular**	80 (50.6%)	37 (68.5%)	117 (55.2%)	0.027
**Respiratory**	85 (53.8%)	27 (50.0%)	112 (52.8%)	0.639
**Number of comorbidities**				0.003
None	5 (3.2%)	0 (0.0%)	5 (2.4%)	
1	12 (7.6%)	0 (0.0%)	12 (5.7%)	
2	25 (15.8%)	2 (3.7%)	27 (12.7%)	
3 or more	116 (73.4%)	52 (96.3%)	168 (79.2%)	

For peer review only

	Survivor (n=158)	Deceased (n=54)	Total (n=212)	P value
Lifestyle factors				
**Smoker**				0.014
Not available	6 (3.8%)	0 (0.0%)	6 (2.8%)	
Never	63 (39.9%)	17 (31.5%)	80 (37.7%)	
Past	67 (42.4%)	35 (64.8%)	102 (48.1%)	
Current	22 (13.9%)	2 (3.7%)	24 (11.3%)	
**Drinker**				0.951
Not available	22 (13.9%)	7 (13.0%)	29 (13.7%)	
Never	38 (24.1%)	11 (20.4%)	49 (23.1%)	
Past	34 (21.5%)	12 (22.2%)	46 (21.7%)	
Current	64 (40.5%)	24 (44.4%)	88 (41.5%)	
**Substance user**				0.087
Not available	76 (48.1%)	19 (35.2%)	95 (44.8%)	
Never	23 (14.6%)	6 (11.1%)	29 (13.7%)	
Past	11 (7.0%)	2 (3.7%)	13 (6.1%)	
Current	48 (30.4%)	27 (50.0%)	75 (35.4%)	
**Obese**				0.708
Not available	1 (0.6%)	1 (1.9%)	2 (0.9%)	
Never	17 (10.8%)	5 (9.3%)	22 (10.4%)	
Past	78 (49.4%)	29 (53.7%)	107 (50.5%)	
Current	62 (39.2%)	19 (35.2%)	81 (38.2%)	
Prescription medication use				
**ACE inhibitor**				0.025
Non-user	126 (79.7%)	33 (61.1%)	159 (75.0%)	
Past user	15 (9.5%)	9 (16.7%)	24 (11.3%)	
Current user	17 (10.8%)	12 (22.2%)	29 (13.7%)	
**Angiotensin receptor blocker**				0.162
Non-user	138 (87.3%)	44 (81.5%)	182 (85.8%)	
Past user	2 (1.3%)	3 (5.6%)	5 (2.4%)	
Current user	18 (11.4%)	7 (13.0%)	25 (11.8%)	
**Aldosterone agonist**				0.81
Non-user	147 (93.0%)	49 (90.7%)	196 (92.5%)	
Past user	5 (3.2%)	2 (3.7%)	7 (3.3%)	
Current user	6 (3.8%)	3 (5.6%)	9 (4.2%)	
**β-blocker**				0.891
Non-user	111 (70.3%)	37 (68.5%)	148 (69.8%)	
Past user	9 (5.7%)	4 (7.4%)	13 (6.1%)	
Current user	38 (24.1%)	13 (24.1%)	51 (24.1%)	
**Calcium channel blocker**				0.217
Non-user	121 (76.6%)	37 (68.5%)	158 (74.5%)	
Past user	13 (8.2%)	3 (5.6%)	16 (7.5%)	
Current user	24 (15.2%)	14 (25.9%)	38 (17.9%)	
**Alpha agonist**				NA
Non-user	158 (100.0%)	54 (100.0%)	212 (100.0%)	

For peer review only

	Survivor (n=158)	Deceased (n=54)	Total (n=212)	P value
**Thiazide**				1
Non-user	157 (99.4%)	54 (100.0%)	211 (99.5%)	
Current user	1 (0.6%)	0 (0.0%)	1 (0.5%)	
**Antiplatelet**				0.052
Non-user	119 (75.3%)	32 (59.3%)	151 (71.2%)	
Past user	13 (8.2%)	5 (9.3%)	18 (8.5%)	
Current user	26 (16.5%)	17 (31.5%)	43 (20.3%)	
**Antiarrhythmic**				0.597
Non-user	154 (97.5%)	52 (96.3%)	206 (97.2%)	
Past user	1 (0.6%)	1 (1.9%)	2 (0.9%)	
Current user	3 (1.9%)	1 (1.9%)	4 (1.9%)	
**Anticoagulant**				0.793
Non-user	147 (93.0%)	49 (90.7%)	196 (92.5%)	
Past user	4 (2.5%)	1 (1.9%)	5 (2.4%)	
Current user	7 (4.4%)	4 (7.4%)	11 (5.2%)	
**Glucocorticoid**				0.384
Non-user	95 (60.1%)	27 (50.0%)	122 (57.5%)	
Past user	15 (9.5%)	7 (13.0%)	22 (10.4%)	
Current user	48 (30.4%)	20 (37.0%)	68 (32.1%)	
**β2-agonist**				0.373
Non-user	128 (81.0%)	39 (72.2%)	167 (78.8%)	
Past user	5 (3.2%)	3 (5.6%)	8 (3.8%)	
Current user	25 (15.8%)	12 (22.2%)	37 (17.5%)	
**Muscarinic antagonist**				0.853
Non-user	127 (80.4%)	43 (79.6%)	170 (80.2%)	
Past user	6 (3.8%)	3 (5.6%)	9 (4.2%)	
Current user	25 (15.8%)	8 (14.8%)	33 (15.6%)	
**NSAID**				0.489
Non-user	139 (88.0%)	46 (85.2%)	185 (87.3%)	
Past user	9 (5.7%)	2 (3.7%)	11 (5.2%)	
Current user	10 (6.3%)	6 (11.1%)	16 (7.5%)	
**Vitamin D**				0.013
Non-user	116 (73.4%)	28 (51.9%)	144 (67.9%)	
Past user	13 (8.2%)	7 (13.0%)	20 (9.4%)	
Current user	29 (18.4%)	19 (35.2%)	48 (22.6%)	
**Proton pump inhibitor**				1
Non-user	76 (48.1%)	27 (50.0%)	103 (48.6%)	
Past user	17 (10.8%)	5 (9.3%)	22 (10.4%)	
Current user	65 (41.1%)	22 (40.7%)	87 (41.0%)	

For peer review only

	Survivor (n=158)	Deceased (n=54)	Total (n=212)	p value
**Statin**				0.023
Non-user	86 (54.4%)	18 (33.3%)	104 (49.1%)	
Past user	18 (11.4%)	8 (14.8%)	26 (12.3%)	
Current user	54 (34.2%)	28 (51.9%)	82 (38.7%)	
**Immuno-suppressant**				0.657
Non-user	150 (94.9%)	50 (92.6%)	200 (94.3%)	
Past user	3 (1.9%)	1 (1.9%)	4 (1.9%)	
Current user	5 (3.2%)	3 (5.6%)	8 (3.8%)	
Complications post diagnosis				
**Cardiovascular**				0.072
No	62 (39.2%)	14 (25.9%)	76 (35.8%)	
Recurrent	80 (50.6%)	37 (68.5%)	117 (55.2%)	
Novel	16 (10.1%)	3 (5.6%)	19 (9.0%)	
**Respiratory**				0.723
No	43 (27.2%)	14 (25.9%)	57 (26.9%)	
Recurrent	85 (53.8%)	27 (50.0%)	112 (52.8%)	
Novel	30 (19.0%)	13 (24.1%)	43 (20.3%)	
**Renal**				< 0.001
No	66 (41.8%)	7 (13.0%)	73 (34.4%)	
Recurrent	73 (46.2%)	41 (75.9%)	114 (53.8%)	
Novel	19 (12.0%)	6 (11.1%)	25 (11.8%)	
**Recurrent complications**				0.06
None	37 (23.4%)	5 (9.3%)	42 (19.8%)	
1	41 (25.9%)	11 (20.4%)	52 (24.5%)	
2	43 (27.2%)	20 (37.0%)	63 (29.7%)	
3	37 (23.4%)	18 (33.3%)	55 (25.9%)	
**Novel complications**				0.507
None	103 (65.2%)	38 (70.4%)	141 (66.5%)	
1	46 (29.1%)	11 (20.4%)	57 (26.9%)	
2	8 (5.1%)	4 (7.4%)	12 (5.7%)	
3	1 (0.6%)	1 (1.9%)	2 (0.9%)	

Values are n (%), unless otherwise specified. *Percentages total more than 100% because patients might have had non-malignant diseases in multiple organs.

Risks of SARS-CoV-2 infection

The risk analyses showed a greater risk of COVID-19 for men, the elderly (over 70s) and the Black community (figure 2). Patients from the South Asian ethnic group and those with pancreatic and liver conditions were also associated with a higher risk of infection (risk ratio 1.49, 95% confidence interval 1.08 to 2.04, P=0.025; 1.61, 1.13 to 2.24, P=0.015; 1.55, 1.1 to 2.18, P=0.024). However, post-hoc adjustment for comorbidities returned a reduced non-significant positive risk (1.19, 0.85 to 1.64, P=0.436; 1.36, 0.96 to 1.9, P=0.174; 1.32, 0.93 to 1.85, P=0.206), with diabetes principally responsible for this reduction (supplemental table 3). Patients with pre-existing kidney conditions were at the highest risk of COVID-19 (2.61, 1.97 to 3.47, P<0.001), followed by a more than two-fold increased risk for patients with

For peer review only

hypertension, diabetes, cardiovascular or respiratory disease (figure 2). However, the independent effects of hypertension and high cholesterol were absent when adjusted for other comorbidities (supplemental table 3).

<<Figure 2 here>>

Figure 2 Risk ratio estimates of COVID-19 for HPB patients with specific demographic, comorbidity, lifestyle and medication use characteristics. Risk ratio estimates for demographic characteristics are mutually adjusted for each other, i.e., gender, ethnicity, age group and HPB diagnosis. For comorbidity, lifestyle and medication use characteristics, risk ratio estimates are adjusted for gender, ethnicity, simplified age group (under and over 60) and HPB diagnosis.

Risks of COVID-19 related death

The risk analyses showed an increased risk of COVID-19 related death for elderly patients (over 70s), individuals from the Black community and patients with recurrent renal complications (figure 3). COVID-19 patients with recurrent renal complications were associated with a higher risk of death, whereas those with prevalent kidney conditions or recent users of ACE inhibitors showed a trend towards significance. However, post-hoc adjustment for comorbidities returned a non-significant positive risk for all these risk factors (supplemental table 4). No HPB sub-groups were particularly more vulnerable to COVID-related death. No other comorbidities, lifestyle factors, medication history or post-diagnosis complications had a significant effect on the COVID-19 patients' outcome (figure 3).

<<Figure 3 here>>

Figure 3 Risk ratio estimates of COVID-19 related death for HPB patients with specific demographic, comorbidity, lifestyle, medication use and post COVID-19 diagnosis complication characteristics. Risk ratio estimates for demographic characteristics are mutually adjusted for each other, i.e., gender, ethnicity, age group and HPB diagnosis. For comorbidity, lifestyle, medication use and post diagnosis. complication characteristics, risk ratio estimates are adjusted for gender, ethnicity, simplified age group (under and over 60) and HPB diagnosis.

For peer review only

For peer review only - http://bmjopen.bmj.com/site/about/guidelines.xhtml

DISCUSSION

We present, for the first time, data on a large, single-centre, multi-ethnic cohort of HPB patients, where primary, secondary and tertiary care EHRs were integrated to investigate the incidence and outcome of COVID-19, to demonstrate how key demographic characteristics and a range of comorbidities, lifestyle factors and medications are associated with SARS-CoV-2 infection and poor outcomes in HPB patients.

Strengths and limitations of the study

A key strength of our study is that we have systematically identified the effect, or the lack of it, of individual demographic and clinical factors on the infection and mortality of COVID-19 in a cohort of over 15000 patients, robustly corrected for potential confounders in their evaluation. Our large population is highly representative of HPB patients from diverse ethnic groups, which contributes to the generalisability of our findings. Another strength is our use of linked electronic health records, harmonised for variations in coding that exist between different EHR systems. We ascertained patient demographics, lifestyle, comorbidities and medications by linking hospital records with pseudo-anonymized longitudinal primary care records, which substantially enrich the data that are recorded on hospital visits.

Retrospective EHR-based COVID-19 studies often suffer from incomplete or missing data on patient characteristics, including key variables such as BMI, ethnicity, smoking or pre-existing comorbidities.[4 50] The missing data is particularly applicable to otherwise healthy COVID-19 patients with low use of healthcare services in the past. However, our patient cohort had already been treated or managed at BHNT hospitals at least once, and often referred through primary care, which led to near-complete data for this study, an added advantage of this study. For instance, ethnicity, a common demographic feature, is missing only for 2.9% of cases in our cohort while the rate is significantly higher in other studies (up to 20% of cases).[4 50] The only variable with missing data frequency over 20% in our study is substance mis-use behaviour (50.6%). This is a unique lifestyle risk variable which is not yet explored - understandably due to a lack of recorded data as people often do not disclose this information to their clinicians,[51] unless manifested in physical or mental disorders. Yet, the substance mis-use history of over 7700 patients included in this study provide a good indication of the impact of COVID-19 on this under-studied group.

Our study also has some important limitations. In our cohort definition, only patients visiting BHNT hospitals and who were given a confirmed COVID-19 diagnosis code or tested positive for SARS-CoV-2 RNA were considered as having COVID-19. This may have resulted in some patients being incorrectly identified as not having COVID-19, particularly if they had a positive swab test via NHS coronavirus test sites or using a home test kit. However, our cohort had specific medical conditions with a high prevalence of comorbidities listed under the NHS categorisation of higher risk populations.[3] Any manifestation of COVID-19 related symptoms would have likely resulted in these patients receiving treatment at one of the BHNT hospitals, and hence we were fairly confident on identifying all clinically relevant SARS-CoV-2 infections in our cohort within the three East London boroughs.

For peer review only - http://bmjopen.bmj.com/site/about/guidelines.xhtml

A related limitation is associated with the confirmation of East London residency for the study cohort. Patients' addresses (current or historic) are not collected under the umbrella study, which considers patients with HPB conditions (with the exception of cancer) treated or managed at BHNT hospitals as East London residents during the time of their care. The Royal London Hospital hosts one of the largest HPB centres in England, and supports suspected or confirmed HPB cancer patients from nearby geographical areas. As the umbrella study cohort is historic, we acknowledged the probability of people moving away from East London in the meantime. In absence of a patient's current address to confirm their residency at the outset of COVID-19 pandemic in the UK, we relied on an indirect measure to infer residency. We used a strict six-month window preceding the study to identify a patient's interaction with East London GPs or BHNT hospitals. Any supposed reduction in the cohort size due to unaccounted change of residency within that window should have affected the COVID and non-COVID group in equal proportion.

Due to the rarity of the outcome (SARS-CoV-2 infection) in the full HPB cohort, the effects reported in the study could be influenced by the smaller cohort size of COVID-19 cases. We recognise that larger sample sizes of COVID-19 patients are needed to fully understand the effect of SARS-CoV-2 in patients with HPB conditions. Our results are the first step towards this and require validation in similar national and international cohorts.

Comparison with other studies

We noted a higher risk of COVID-19 in patients with prior pancreatic and liver conditions. The higher risk associated with liver condition is consistent with earlier findings.[6 19] We can speculate that reduced pancreatic function, leading to altered digestion, and therefore gut flora, may make patients more susceptible to pathogens with an enteric route of infection:[30] an alternative mode of infection for SARS-CoV2, in addition to the well-characterised respiratory mode. Surprisingly the most vulnerable cancer patients had a low COVID-19 incidence rate, which may reflect the effectiveness of public health interventions such as shielding.[31] However, at the same time, we noted a 12.7% death rate in this cohort (not due to COVID-19) in just four months, perhaps indicating the unintended, but potentially inevitable, negative sequelae of social distancing and reduced healthcare provisions for this group of patients as resources were diverted to COVID-19 affected patients.

Men had a higher risk of infection than women, which is consistent with previous reports,[1 14] and could be due to a favourable genetic predisposition to the virus,[31] and/or gender differences in risk behaviours. Our study also affirms older age, particularly over 70, as an established risk factor for COVID-19 incidence and mortality;[2 4 5] however, this can be largely explained by the presence of multiple comorbidities in the older age groups.[32]

COVID-19 statistics have highlighted a disproportionate effect on BAME ethnic groups with an increased risk of infection and poor outcomes.[13-15] Our results confirm that Black and South Asian communities are at a higher risk of COVID-19 compared to the White ethnic group, with added mortality risk for the Black community. However, the increased risk noted in the South Asian group is likely to be associated with the

For peer review only - http://bmjopen.bmj.com/site/about/guidelines.xhtml

underlying comorbidities in this group, in particular the higher prevalence of diabetes. Only a small part of the excess risk in the Black community is explained by multiple comorbidities. Therefore, further variables such as deprivation, occupational exposure, and living conditions might be useful to explore as potential factors behind the apparent vulnerability of the Black population to COVID-19.

All comorbidities such as diabetes, hypertension, high cholesterol, cardiovascular disease, kidney, and respiratory disease, were independently associated with an increased risk of COVID-19, whereas presence of kidney disease contributed to an added risk of death. These findings largely concur with previously reported cohort studies.[4-6 11] Our results particularly highlight that for patients with an underlying kidney disease, a subsequent renal complication due to SARS-CoV-2 infection could be fatal. Acute kidney injury (AKI) and the presence of underlying kidney disease on admission have been associated with increased in-hospital deaths of COVID-19 patients.[4 6 33] Therefore, patients with weaker kidney function, i.e., history of chronic kidney disease and/or on dialysis, need to be carefully managed to prevent further renal insult due to COVID-19.

The link between smoking and the susceptibility to COVID-19 is controversial in that current smoking status appeared to have a protective effect in our cohort, as has been observed by others, an aspect which cannot be mechanistically explained.[5 34 35] Smoking leads to severe health consequences, which explains the greater risk observed in our cohort of past smokers with high prevalence of respiratory and cardiovascular diseases. Carefully designed analyses are needed to explore the association and causality between smoking status (both current and past), associated comorbidities and COVID-19.

Although substance mis-use leads to a plethora of cardio-respiratory and metabolic problems, its role in COVID-19 remains unexplored. To date, this is the first study providing a concrete measure of the risk of COVID-19 for substance mis-users. Our initial results showing that substance mis-users are at a heightened risk for COVID-19 irrespective of the comorbidities warrants a strong case for considering it as an independent risk factor for COVID-19, and may be related to high-risk behavioural patterns. [36,37]

Previous studies have found a significant relationship between obesity and an increased risk of COVID-19[,7] and subsequent hospitalisation,[38 39] advanced levels of treatment,[15,27] and death.[4 6] However, our study does not suggest any particular effect of obesity on COVID-19 for patients with HPB conditions, who have a much higher prevalence rate of obesity (37.8%) compared to the UK general population (26%).[28] With 91% of patients in our cohort having a history of obesity (current or past), the difference in effects for potential susceptibility to COVID-19 are more detectible for other less prevalent factors – such as type 2 diabetes, cardiovascular disease or hypertension[40 41] – which in turn might be the consequences of obesity in these patients' lifetime.

Concerns have been raised regarding the use of various medications with respect to the risk of COVID-19 and the subsequent outcome; and, our analyses contribute to that discussion for some of the widely used prescription drugs. An important finding from our study is the significant risk observed for vitamin D users, suggestive of a

For peer review only - http://bmjopen.bmj.com/site/about/guidelines.xhtml

causal relationship between vitamin D deficiency or specific medical conditions (such as kidney failure) where Vitamin D prescription is prevalent and development of COVID-19.[42 43] Given that BAME communities are observed to be at a high risk of COVID-19, and there is evidence that vitamin D deficiency is particularly common in these ethnic groups,[42] further research on the relationship between vitamin D and COVID-19 is required, with a need to exclude confounding factors such as kidney disease. Our result also suggest that patients currently taking PPIs are more susceptible to SARS-CoV-2 infection, which concurs with a large population-based online survey conducted in the US.[44] The use of PPIs is highly prevalent in HPB patients for the management of gastrointestinal acid-related disorders, and the finding here supports the hypothesis that current use of PPIs might influence the susceptibility to SARS-CoV-2 infection in the gastrointestinal tract through reduction of stomach acid.[44 45]

The literature is conflicted on the potential impact of antihypertensive drugs on COVID-19, particularly those that act as inhibitors to the renin–angiotensin–aldosterone system (RAAS) and upregulate ACE2 expression, suggesting these drugs may be potential risk factors for infection,[46 47] but also as having a protective effect on outcome.[48] However, recent studies found no underlying association between the use of different classes of antihypertensive drugs and the risk of developing COVID-19.[16] With a high percentage of patients with hypertension in the study cohort, our finding that a high risk of COVID-19 is associated with past intake of ACE inhibitors or aldosterone agonists is suggestive of the potential risk of switching from one class of antihypertensive drug to another. This contributes to the timely debate of whether discontinuation of RAAS inhibitors and considering alternative antihypertensive therapy in times of COVID-19 would be a good practice or not.[49] A marginal association of current use of ACE inhibitors with COVID-19 related death suggests that any increased risk of mortality is likely to be small and will need to be scrutinised in future as more data accumulates.

Conclusions

We believe that the findings from this single-centre study, focusing on patients with a particular medical condition and in an ethnically diverse area, highlight some considerations that could guide clinical care while we await an effective antiviral strategy for COVID-19. The current findings reinforce our understanding of some of the important risk factors for SARS-CoV-2 infection but with regards to pre-existing HPB conditions, and allows stratification for risk, thereby providing a tool for policy makers to divert prevention as well as treatment to a clearly identified vulnerable population.

For peer review only - http://bmjopen.bmj.com/site/about/guidelines.xhtml

ACKNOWLEDGEMENTS

ADU is supported by Health Data Research UK (HDR-UK) to conduct the umbrella study EL-PaC-Epidem, which is funded by the UK Medical Research Council. We gratefully acknowledge support provided by Pancreatic Cancer Research Fund (PCRF), for conducting public-patient engagement activity and facilitating ethical approval for EL-PaC-Epidem. We thank Dr Charles Gutteridge, Chief Clinical Information Officer at Barts Health NHS Trust, members of the Discovery East London Programme Board, and developers at Learning Health Solutions Ltd for their support in facilitating collection of primary, secondary and tertiary care patient records. Finally, we acknowledge the contribution to the research made by several members of the PCRF Tissue Bank team, Bioinformatics Unit and clinical research fellows at Barts Cancer Institute through insightful medical and scientific discussion.

FOOTNOTES

Contributors: ADU designed the study, and was responsible for undertaking and completing data collection, processing and analysis. HMK and CC oversaw the conduct and management of the study. All the authors contributed to the selection of study variables and interpretation post analysis. ADU wrote the first drafts of the report and all the authors made critical revisions.

Funding: The study is conducted under an umbrella study, focusing on the epidemiology of pancreatic and other hepatobiliary cancers in East London (EL-PaC-Epidem), funded by Medical Research Council UK (Ref: MR/S003835/1) as a UKRI/Rutherford Fellowship to the corresponding author. No additional funding has been received for this study.

Competing interests: All authors declare no competing interests.

Ethics approval: All data utilised for this study were collected and processed under the EL-PaC-Epidem study at Barts Health NHS Trust. The study was approved by the East of England - Essex Research Ethics Committee (19/EE/0163; 17 May 2019) and supported by the NHS Confidentiality Advisory Group for collecting and processing confidential patient information without consent (19/CAG/0219; 17 January 2020).

Data sharing: All statistical data relevant to the study are included in the article or uploaded as supplementary information. Only the corresponding author had full access to all the participants' data in the study. The authors confirm that researchers seeking the completely anonymised final analysis dataset for this work can submit a data request to the corresponding author.

Transparency statement: The corresponding author affirms that the manuscript is an honest, accurate, and transparent account of the study being reported; that no important aspects of the study have been omitted; and that any discrepancies from the study as planned (and, if relevant, registered) have been explained.

Dissemination to participants and related patient and public communities: Key findings will be disseminated in the EL-PaC-Epidem study website as well as in the corresponding author's institute website.

For peer review only - http://bmjopen.bmj.com/site/about/guidelines.xhtml

REFERENCES

1 World Health Organization. Coronavirus disease (COVID-19): Situation Reports. https://www.who.int/emergencies/diseases/novel-coronavirus-2019/situation-reports (accessed July 30, 2020).

2 Centers for Disease Control and Prevention. Coronavirus Disease 2019 (COVID-19): People at Increased Risk and Other People Who Need to Take Extra Precautions. https://www.cdc.gov/coronavirus/2019-ncov/need-extra-precautions/index.html (accessed August 10, 2020).

3 NHS England. Coronavirus (COVID-19): People at Higher Risk from Coronavirus. https://www.nhs.uk/conditions/coronavirus-covid-19/people-at-higher-risk/whos-at-higher-risk-from-coronavirus/ (accessed July 30, 2020)

4 Williamson EJ, Walker AJ, Bhaskaran K, et al. Factors associated with COVID-19-related death using OpenSAFELY. *Nature* 2020; **584** (7821): 430–36.

5 Deng G, Yin M, Chen X, Zeng F. Clinical determinants for fatality of 44,672 patients with COVID-19. *Crit Care* 2020; **24**: 179.

6 Docherty AB, Harrison EM, Green CA, et al. Features of 20 133 UK patients in hospital with covid-19 using the ISARIC WHO Clinical Characterisation Protocol: prospective observational cohort study. *BMJ* 2020; **369**: m1985.

7 Lighter J, Phillips M, Hochman S, et al. Obesity in Patients Younger Than 60 Years Is a Risk Factor for COVID-19 Hospital Admission. *Clin Infect Dis* 2020; **71** (15): 896–7.

8 Dai M, Liu D, Liu M, et al. Patients with Cancer Appear More Vulnerable to SARS-CoV-2: A Multicenter Study during the COVID-19 Outbreak. *Cancer Discov* 2020; **10** (6): 783–91.

9 Blanco JL, Ambrosioni J, Garcia F, et al. COVID-19 in patients with HIV: clinical case series. *Lancet HIV* 2020; **7** (5): e314–6.

10 BMJ Best Practice. Coronavirus disease 2019 (COVID-19) Complications. https://bestpractice.bmj.com/topics/en-gb/3000168/complications (accessed July 23, 2020)

11 Guan W-J, Liang W-H, Zhao Y, et al. Comorbidity and its impact on 1590 patients with COVID-19 in China: a nationwide analysis. *Eur Respir J* 2020; **55** (5): 2000547.

12 Miyara, M. et al. Low incidence of daily active tobacco smoking in patients with symptomatic COVID-19. *Qeios* 2020; doi: 10.32388/WPP19W.3

13 Platt L, Warwick R. Are some ethnic groups more vulnerable to COVID-19 than others? Institute for Fiscal Studies, May, 2020; https://www.ifs.org.uk/inequality/wp-content/uploads/2020/04/Are-some-ethnic-groups-more-vulnerable-to-COVID-19-than-others-V2-IFS-Briefing-Note.pdf (accessed July 23, 2020)

14 Public Health England. Disparities in the risk and outcomes of COVID-19. https://assets.publishing.service.gov.uk/government/uploads/system/uploads/attachment_data/file/889195/disparities_review.pdf August, 2020. (accessed August 14, 2020).

15 Intensive Care National Audit and Research Centre. ICNARC report on COVID-19 in critical care. https://www.icnarc.org/DataServices/Attachments/Download/af7be2d4-bdcd-ea11-9127-00505601089b July 24, 2020. (accessed August 2, 2020).

16 National Institute for Health and Care Excellence. Coronavirus (COVID-19) Rapid Evidence Summaries. https://www.nice.org.uk/covid-19#rapid-es (accessed July 16, 2020)

For peer review only - http://bmjopen.bmj.com/site/about/guidelines.xhtml

17 Brenner EJ, Ungaro RC, Gearry RB, et al., Corticosteroids, but not TNF Antagonists, are Associated with Adverse COVID-19 Outcomes in Patients With Inflammatory Bowel Diseases: Results from an International Registry. *Gastroenterology* 2020; **159** (2): 481–91.
18 Zhang H, Kang Z, Gong H, et al. Digestive system is a potential route of COVID-19: an analysis of single-cell coexpression pattern of key proteins in viral entry process. *Gut* 2020; **69**: 1010–8.
19 Mantovani A, Beatrice G, Dalbeni A. Coronavirus disease 2019 and prevalence of chronic liver disease: A meta-analysis. *Liver Int* 2020; **40**(6): 1316–20.
20 Gubatan J, Levitte S, Patel A, et al. Prevalence, risk factors and clinical outcomes of COVID-19 in patients with a history of pancreatitis in Northern California. *Gut* 2020; Published Online: June 3, 2020. doi: 10.1136/gutjnl-2020-321772.
21 McNabb-Baltar J, Jin DX, Grover AS, et al. Lipase elevation in patients with COVID-19. *Am J Gastroenterol* 2020; Published Online: Jun 3, 2020. doi: 10.14309/ajg.0000000000000732.
22 Hadi A, Werge M, Kristiansen KT, et al. Coronavirus Disease-19 (COVID-19) associated with severe acute pancreatitis: case report on three family members. *Pancreatology* 2020; **20** (4): 665–7.
23 Katabathina VS, Flaherty EM, Dasyam AK, et al. "Biliary Diseases with Pancreatic Counterparts": Cross-sectional Imaging Findings. *Radiographics* 2016; **36** (2): 374–92.
24 UK Government. Coronavirus cases in the UK: daily updated statistics. https://www.gov.uk/guidance/coronavirus-covid-19-information-for-the-public (last accessed August 2, 2020)
25 London Datastore. Coronavirus (COVID-19) Cases: Greater London Authority (GLA). https://data.london.gov.uk/dataset/coronavirus--covid-19--cases (accessed August 4, 2020)
26 Office for National Statistics. Deaths involving COVID-19 by local area and socioeconomic deprivation: deaths occurring between 1 March and 30 June 2020. https://www.ons.gov.uk/peoplepopulationandcommunity/birthsdeathsandmarriages/deaths/bulletins/deathsinvolvingcovid19bylocalareasanddeprivation/deathsoccurringbetween1marchand30june2020 July 24, 2020. (accessed August 4, 2020)
27 Barts Health NHS Trust. https://www.bartshealth.nhs.uk/about-us (accessed August 10, 2020)
28 London Datastore. Ethnic Groups by Borough. https://data.london.gov.uk/dataset/ethnic-groups-borough (accessed August 4, 2020)
29 East London Health & Care Partnership. https://www.eastlondonhcp.nhs.uk/aboutus/ (accessed August 4, 2020)
30 Thaweerat W. Current evidence on pancreatic involvement in SARS-CoV-2 infection. *Pancrteatology* 2020; **20** (5): 1013–4.
31 Public Health England. Guidance on shielding and protecting people who are clinically extremely vulnerable from COVID-19. https://www.gov.uk/government/publications/guidance-on-shielding-and-protecting-extremely-vulnerable-persons-from-covid-19/guidance-on-shielding-and-protecting-extremely-vulnerable-persons-from-covid-19 (accessed July 16, 2020).
32 Gibson WT, Evans DM, An J, Jones SJM. ACE 2 Coding Variants: A Potential X-linked Risk Factor for COVID-19 Disease. Preprint published online: April 14, 2020. doi: https://doi.org/10.1101/2020.04.05.026633
33 World Health Organization, Europe. Statement – Older people are at highest risk from COVID-19, but all must act to prevent community spread.

For peer review only - http://bmjopen.bmj.com/site/about/guidelines.xhtml

https://www.euro.who.int/en/health-topics/health-emergencies/coronavirus-covid-19/statements/statement-older-people-are-at-highest-risk-from-covid-19,-but-all-must-act-to-prevent-community-spread April 2, 2020. (accessed June 12, 2020)

34 Cheng Y, Luo R, Wang K, et al. Kidney disease is associated with in-hospital death of patients with COVID-19. *Kidney Int* 2020; **97** (5): 829–38.

35. Rentsch CT, Kidwai-Khan F, Tate JP, et al. Covid-19 testing, hospital admission, and intensive care among 2,026,227 United States veterans aged 54–75 years. Preprint at *medRxiv* April 14, 2020; doi: https://doi.org/10.1101/2020.04.09.20059964.

36 Ornell F, Moura HF, Scherer JN, et al. The COVID-19 pandemic and its impact on substance use: Implications for prevention and treatment. *Psychiatry Res* 2020; **289**: 113096.

37 Dubey MJ, Ghosh R, Chatterjee S, Biswas P, Chattergee S, Dubey S. COVID-19 and addiction. *Diabetes Metab Syndr* 2020; **14** (5): 817–23.

38. Khawaja AP, Warwick AN, Hysi PG, et al. Associations with COVID-19 hospitalisation amongst 406,793 adults: the UK Biobank prospective cohort study. Preprint at *medRxiv* May 11, 2020; doi: https://doi.org/10.1101/2020.05.06.20092957

39 Petrilli CM, Jones SA, Yang J, et al. Factors associated with hospitalization and critical illness among 4,103 patients with COVID-19 disease in New York City. Preprint at *medRxiv* April 11, 2020. doi: 10.1101/2020.04.08.20057794

40 Public Health England. Excess Weight and COVID-19: Insights from new evidence https://assets.publishing.service.gov.uk/government/uploads/system/uploads/attachment_data/file/907966/PHE_insight_Excess_weight_and_COVID-19__FINAL.pdf July, 2020 (accessed August 5, 2020)

41 World Health Organization. Fact sheets: Obesity and overweight https://www.who.int/en/news-room/fact-sheets/detail/obesity-and-overweight (accessed August 5, 2020)

42 Hastie CE, Mackay DF, Ho F, et al. Vitamin D concentrations and COVID-19 infection in UK Biobank. *Diabetes Metab Syndr* 2020; **14** (4): 561–5.

43 D'Avolio A, Avataneo V, Manca A, et al. 25-Hydroxyvitamin D Concentrations Are Lower in Patients with Positive PCR for SARS-CoV-2 *Nutrients* 2020; **12** (5):1359.

44 Almario CV, Chey WD,Spiegel BMR. Increased risk of COVID-19 among users of proton-pump inhibitors. *Am J Gastroenterol* 2020; Preprint at: https://journals.lww.com/ajg/Documents/AJG-20-1811_R1(PUBLISH%20AS%20WEBPART).pdf

45 Lee SW, Yeniova AO, Moon SY, et al. Severe clinical outcomes of COVID-19 associated with proton pump inhibitors: a nationwide cohort study with propensity score matching. *Gut* 2020; Preprint Published Online: July 30, 2020. doi: 10.1136/gutjnl-2020-322248.

46. O'Mara GJ. Could ACE inhibitors and particularly ARBs increase susceptibility to COVID-19 infection? *BMJ* 2020; **368**: m406.

47 Fang L, Karakiulakis G, Roth M. Are patients with hypertension and diabetes mellitus at increased risk for COVID-19 infection? *Lancet Respir Med* 2020; **8** (4): e21.

48 Gurwitz D. Angiotensin receptor blockers as tentative SARS-CoV-2 therapeutics. *Drug Dev Res* 2020; **81** (5): 537–40.

49 Ciulla MM. Switching to another antihypertensive effective drug when using ACEIs/ARBs to treat arterial hypertension during COVID-19. *Eur Heart J* 2020; **41** (19): 1856.

For peer review only - http://bmjopen.bmj.com/site/about/guidelines.xhtml

50 Perez-Guzman PN, Daunt A, Mukherjee S, et al. Clinical characteristics and predictors of outcomes of hospitalized patients with COVID-19 in a multi-ethnic London NHS Trust: a retrospective cohort study. *Clin Infect Dis* 2020; Preprint published online: August 7, 2020. doi: 10.1093/cid/ciaa1091.
51 McNeely J, Kumar PC, Rieckmann T, et al. Barriers and facilitators affecting the implementation of substance use screening in primary care clinics: a qualitative study of patients, providers, and staff. *Addict Sci Clin Pract* 2020; **13** (1): 8.

For peer review only

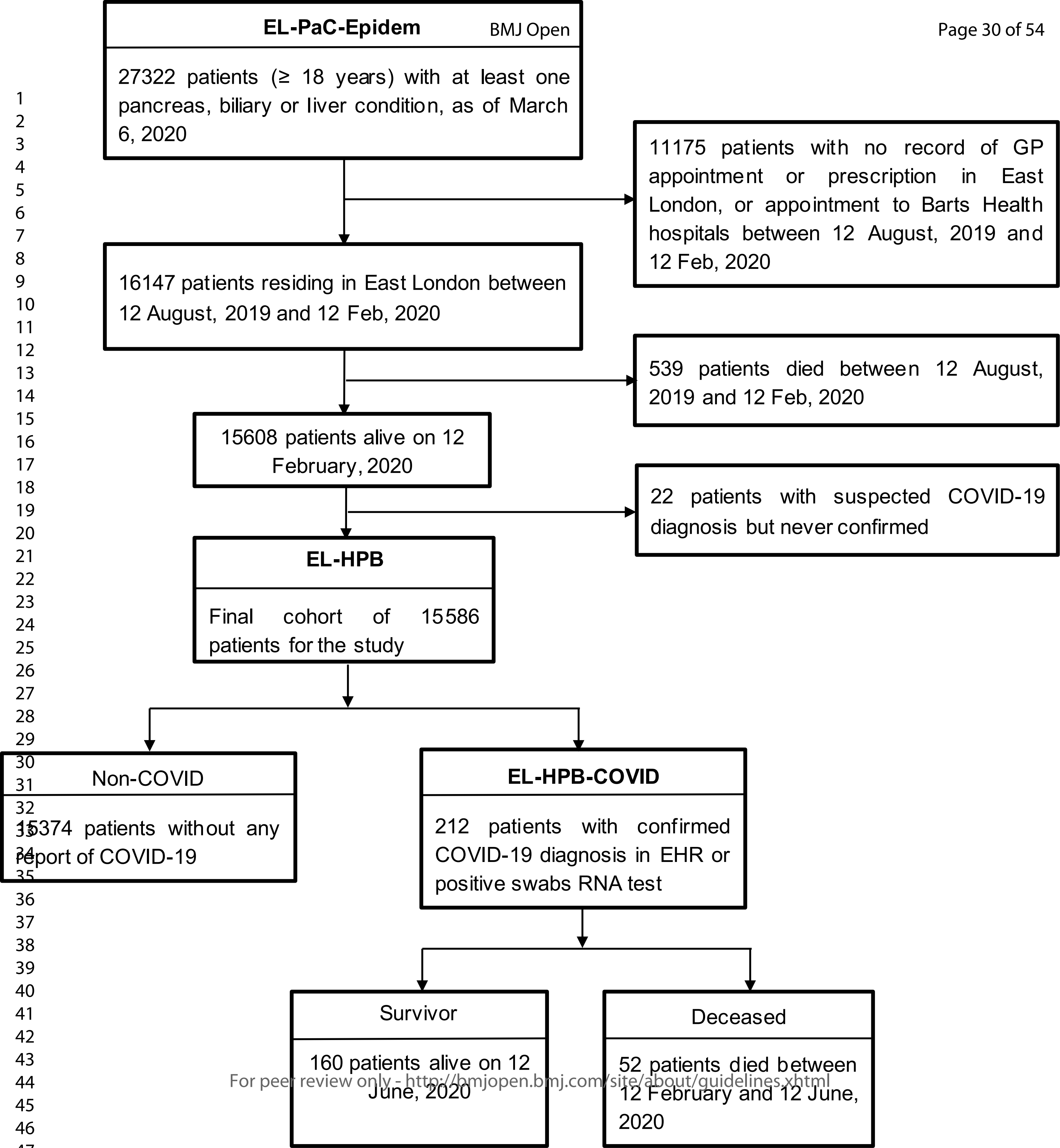
EL-PaC-Epidem
27322 patients (≥ 18 years) with at least one pancreas, biliary or liver condition, as of March 6, 2020
11175 patients with no record of GP appointment or prescription in East London, or appointment to Barts Health hospitals between 12 August, 2019 and 12 Feb, 2020
16147 patients residing in East London between 12 August, 2019 and 12 Feb, 2020
539 patients died between 12 August, 2019 and 12 Feb, 2020
15608 patients alive on 12 February, 2020
22 patients with suspected COVID-19 diagnosis but never confirmed
EL-HPB
Final cohort of 15586 patients for the study
Non-COVID
15374 patients without any report of COVID-19
EL-HPB-COVID
212 patients with confirmed COVID-19 diagnosis in EHR or positive swabs RNA test
Survivor
160 patients alive on 12 June, 2020
Deceased
52 patients died between 12 February and 12 June, 2020

	RR	95% CI	P-val
**Demographics**			
**Gender (ref=Female)**			
Male	1.59	(1.21 to 2.09)	0.003
**Ethnicity (ref=White)**			
South Asian	1.49	(1.08 to 2.04)	0.025
Black	2.20	(1.50 to 3.18)	<0.001
Other	0.82	(0.47 to 1.36)	0.525
**HPB diagnosis**			
Cancer	0.66	(0.16 to 1.84)	0.525
Pancreatic disease	1.61	(1.13 to 2.24)	0.015
Liver disease	1.55	(1.10 to 2.18)	0.024
Bliary disese	1.18	(0.84 to 1.62)	0.462
**Age group (ref=18-40)**			
41-50	1.20	(0.68 to 2.14)	0.541
51-60	1.27	(0.74 to 2.22)	0.496
61-70	1.46	(0.85 to 2.56)	0.272
71-80	3.00	(1.80 to 5.16)	<0.001
80+	5.28	(3.23 to 8.95)	<0.001
**Comorbidities**			
Diabetes	2.42	(1.79 to 3.28)	<0.001
Hypertension	2.43	(1.62 to 3.75)	<0.001
Cholesterol	1.65	(1.22 to 2.26)	0.004
Respiratory	2.10	(1.60 to 2.76)	<0.001
Cardiovascular	2.46	(1.83 to 3.31)	<0.001
Kidney	2.61	(1.97 to 3.47)	<0.001
Multi-morbidity	1.60	(1.45 to 1.76)	<0.001
**Lifestyle (ref=Never)**			
**Smoker**			
Past	1.47	(1.08 to 2.02)	0.024
Current	0.58	(0.35 to 0.93)	0.039
**Drinker**			
Past	1.43	(0.95 to 2.15)	0.114
Current	0.92	(0.63 to 1.34)	0.657
**Substance user**			
Past	3.65	(1.81 to 7.00)	<0.001
Current	2.50	(1.58 to 4.05)	<0.001
**Obese**			
Past	1.47	(0.95 to 2.38)	0.126
Current	1.47	(0.94 to 2.40)	0.126

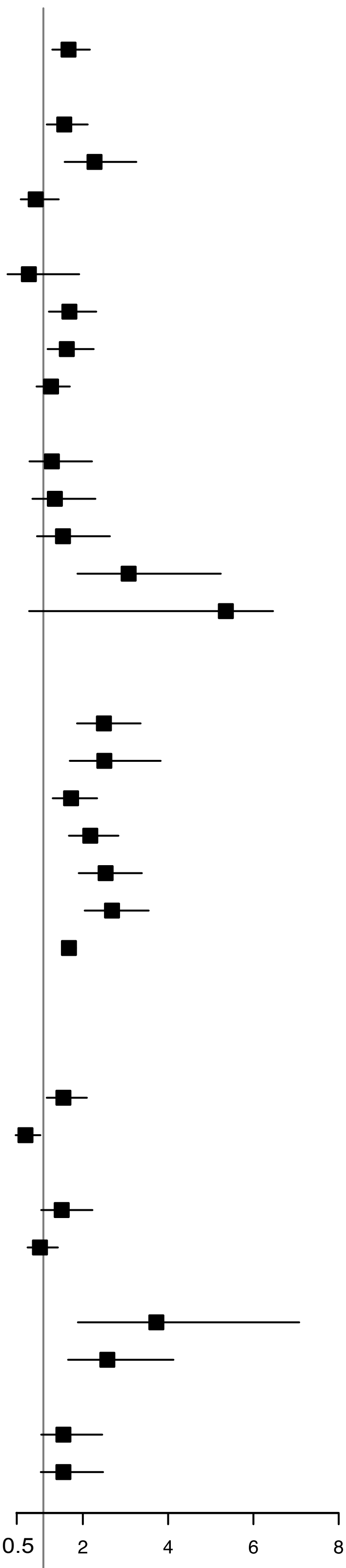

	RR	95% CI	P-val
**Prescription medication use (ref=Non-user)**			
**ACE inhibitor**			
Past user	2.32	(1.48 to 3.49)	<0.001
Current user	0.86	(0.57 to 1.26)	0.47
**Angiotensin receptor blocker**			
Past user	1.11	(0.40 to 2.40)	0.818
Current user	1.22	(0.78 to 1.82)	0.402
**Aldosterone agonist**			
Past user	2.58	(1.11 to 4.97)	0.019
Current user	1.39	(0.66 to 2.53)	0.347
**beta-blocker**			
Past user	1.71	(0.92 to 2.88)	0.072
Current user	1.60	(1.15 to 2.20)	0.007
**Calcium channel blocker**			
Past user	1.36	(0.78 to 2.21)	0.32
Current user	0.87	(0.60 to 1.24)	0.467
**Antiplatelet**			
Past user	2.20	(1.30 to 3.50)	0.003
Current user	1.66	(1.16 to 2.33)	0.006
**Antiarrhythmic**			
Past user	2.43	(0.41 to 7.22)	0.234
Current user	2.07	(0.65 to 4.79)	0.191
**Anticoagulant**			
Past user	2.11	(0.76 to 4.52)	0.124
Current user	1.62	(0.83 to 2.81)	0.136
**Glucocorticoid**			
Past user	1.47	(0.91 to 2.25)	0.112
Current user	2.04	(1.51 to 2.74)	<0.001
**beta2-agonist**			
Past user	2.12	(0.97 to 3.98)	0.039
Current user	1.91	(1.32 to 2.69)	0.001
**Muscarinic antagonist**			
Past user	1.91	(0.91 to 3.48)	0.062
Current user	1.80	(1.22 to 2.58)	0.003
**NSAID**			
Past user	1.00	(0.51 to 1.74)	0.998
Current user	1.55	(0.90 to 2.49)	0.119
**Vitamin D**			
Past user	2.55	(1.55 to 3.95)	<0.001
Current user	2.26	(1.60 to 3.12)	<0.001
**Proton pump inhibitor**			
Past user	1.31	(0.81 to 2.04)	0.278
Current user	1.53	(1.14 to 2.04)	0.006
**Statin**			
Past user	2.10	(1.33 to 3.21)	0.002
Current user	1.42	(1.04 to 1.92)	0.033
**Immunosuppressant**			
Past user	1.32	(0.41 to 3.07)	0.575
Current user	2.01	(0.92 to 3.75)	0.066

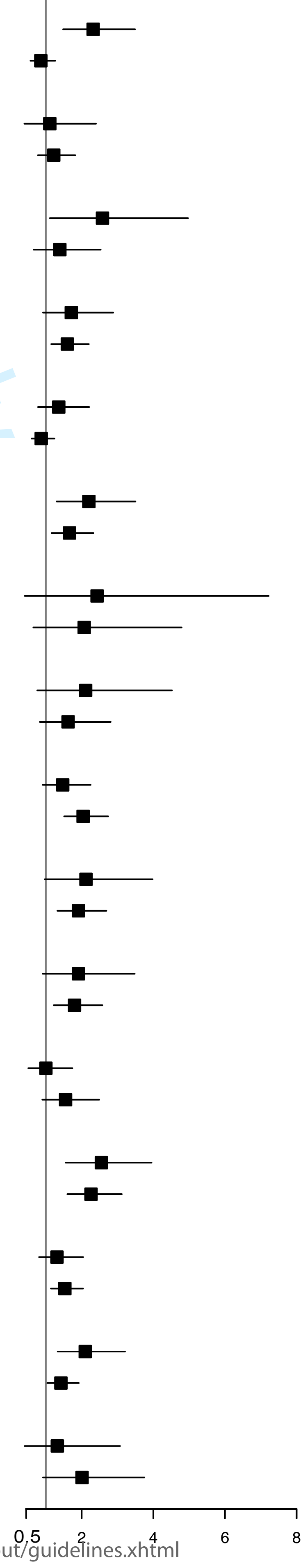

For peer review only

	RR	95% CI	P-val
**Demographics**			
**Gender (ref=Female)**			
Male	1.66	(1.00 to 2.5)	0.156
**Ethnicity (ref=White)**			
South Asian	1.70	(0.94 to 2.7)	0.182
Black	2.40	(1.35 to 3.5)	0.035
Other	0.41	(0.02 to 1.8)	0.446
**HPB diagnosis**			
Cancer	2.69	(0.55 to 3.9)	0.272
Pancreatic disease	1.51	(0.81 to 2.4)	0.272
Liver disease	1.16	(0.62 to 1.8)	0.612
Bliary disese	1.36	(0.72 to 2.3)	0.446
**Age group (ref=18-40)**			
41-50	1.86	(0.30 to 7.1)	0.535
51-60	1.77	(0.33 to 6.8)	0.535
61-70	2.92	(0.73 to 8.1)	0.272
71-80	5.27	(1.87 to 9.6)	0.035
80+	5.17	(1.84 to 9.5)	0.035
**Comorbidities**			
Diabetes	2.00	(1.03 to 3.5)	0.114
Hypertension	5.58	(1.20 to 23.1)	0.169
Cholesterol	1.13	(0.59 to 2.0)	0.765
Respiratory	0.70	(0.39 to 1.2)	0.293
Cardiovascular	1.27	(0.68 to 2.2)	0.477
Kidney	2.13	(1.16 to 3.5)	0.053
Multi-morbidity	1.20	(0.99 to 1.4)	0.157
**Lifestyle (ref=Never)**			
**Smoker**			
Past	1.42	(0.77 to 2.3)	0.421
Current	0.48	(0.07 to 1.6)	0.421
**Drinker**			
Past	1.21	(0.50 to 2.4)	0.642
Current	1.53	(0.74 to 2.6)	0.419
**Substance user**			
Past	0.53	(0.06 to 2.2)	0.55
Current	1.60	(0.59 to 3.2)	0.55
**Obese**			
Past	1.13	(0.43 to 2.5)	0.872
Current	1.00	(0.35 to 2.3)	0.993
**Prescription medication use (ref=Non-user)**			
**ACE inhibitor**			
Past user	1.42	(0.64 to 2.5)	0.395
Current user	2.10	(1.12 to 3.2)	0.071
**Angiotensin receptor blocker**			
Past user	1.87	(0.42 to 3.6)	0.465
Current user	1.23	(0.50 to 2.3)	0.654
**Aldosterone agonist**			
Past user	1.06	(0.18 to 2.6)	0.935
Current user	1.48	(0.36 to 3.0)	0.589
**beta-blocker**			
Past user	1.08	(0.31 to 2.3)	0.887
Current user	0.81	(0.39 to 1.4)	0.627
**Calcium channel blocker**			
Past user	0.44	(0.10 to 1.3)	0.362
Current user	1.40	(0.72 to 2.3)	0.384
**Antiplatelet**			
Past user	1.05	(0.35 to 2.2)	0.92
Current user	1.32	(0.67 to 2.2)	0.509
**Antiarrhythmic**			
Past user	2.35	(0.16 to 3.9)	0.485
Current user	1.06	(0.07 to 3.0)	0.946
**Anticoagulant**			
Past user	0.35	(0.01 to 2.0)	0.465
Current user	1.10	(0.32 to 2.4)	0.86
**Glucocorticoid**			
Past user	1.20	(0.47 to 2.3)	0.729
Current user	1.24	(0.67 to 2.0)	0.562
**beta2-agonist**			
Past user	1.51	(0.38 to 3.1)	0.578
Current user	1.19	(0.57 to 2.0)	0.671
**Muscarinic antagonist**			
Past user	1.15	(0.27 to 2.6)	0.81
Current user	0.86	(0.36 to 1.6)	0.808
**NSAID**			
Past user	0.53	(0.08 to 1.6)	0.378
Current user	1.56	(0.60 to 2.8)	0.378
**Vitamin D**			
Past user	1.60	(0.64 to 3.0)	0.396
Current user	1.70	(0.92 to 2.7)	0.2
**Proton pump inhibitor**			
Past user	0.79	(0.25 to 1.7)	0.618
Current user	0.70	(0.36 to 1.2)	0.356
**Statin**			
Past user	1.20	(0.47 to 2.4)	0.697
Current user	1.42	(0.76 to 2.4)	0.36
**Immunosuppressant**			
Past user	0.70	(0.04 to 2.6)	0.75
Current user	1.62	(0.43 to 3.1)	0.46
**Complications post diagnosis (ref=No)**			
**Cardiovascular**			
Recurrent	1.26	(0.65 to 2.2)	0.568
Novel	0.96	(0.22 to 2.5)	0.954
**Respiratory**			
Recurrent	0.69	(0.33 to 1.3)	0.387
Novel	0.98	(0.43 to 1.9)	0.961
**Renal**			
Recurrent	2.74	(1.32 to 5.1)	0.056
Novel	2.04	(0.60 to 5.0)	0.329

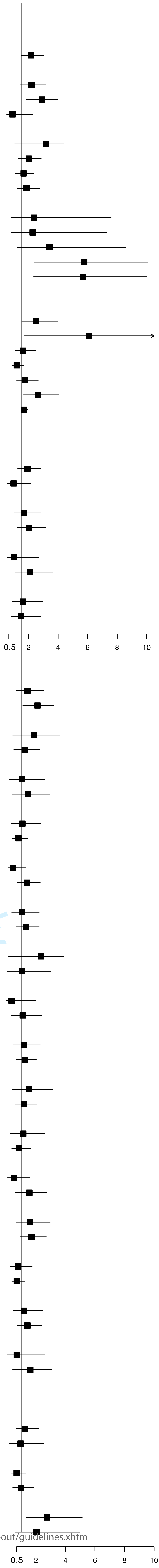

For peer review only

Supplemental Table 1 Codelist for hepato-pancreato-biliary diagnosis groups

Group	Terminology system	Code	Code description	Exclusion
Cancer	ICD-10	C22	Malignant neoplasm of liver and intrahepatic bile ducts	
Cancer	ICD-10	C23	Malignant neoplasm of gallbladder	
Cancer	ICD-10	C24	Malignant neoplasm of other and unspecified parts of biliary tract	
Cancer	ICD-10	C25	Malignant neoplasm of pancreas	
Cancer	ICD-10	D015	Carcinoma in situ of Liver, gallbladder and bile ducts	
Cancer	ICD-10	D017	Carcinoma in situ of Other specified digestive organs incl. Pancreas	
Cancer	ICD-10	D376	Neoplasm of uncertain or unknown behaviour of Liver, gallbladder and bile ducts	
Cancer	SNOMED CT	363418001	Malignant tumor of pancreas (disorder)	94459006 (metastasis to pancreas)
Cancer	SNOMED CT	92672004	Carcinoma in situ of pancreas (disorder)	
Cancer	SNOMED CT	94978003	Neoplasm of uncertain behavior of pancreas (disorder)	
Cancer	SNOMED CT	93870000	Malignant neoplasm of liver (disorder)	94381002 (metastasis to liver)
Cancer	SNOMED CT	92644006	Carcinoma in situ of liver (disorder)	
Cancer	SNOMED CT	94910002	Neoplasm of uncertain behavior of liver (disorder)	
Cancer	SNOMED CT	363415003	Malignant tumor of biliary tract (disorder)	94185003 (metastasis to biliary tract)
Cancer	SNOMED CT	92545000	Carcinoma in situ of biliary tract (disorder)	
Cancer	SNOMED CT	255064003	Neoplasm of uncertain behavior of biliary system (disorder)	
Cancer	READ	B15	Malignant neoplasm of liver and intrahepatic bile ducts	
Cancer	READ	B16	Malignant neoplasm gallbladder and extrahepatic bile ducts	
Cancer	READ	B17	Malignant neoplasm of pancreas	
Cancer	READ	B808.	Carcinoma in situ of liver and biliary system	
Cancer	READ	B8080	Carcinoma in situ of liver	
Cancer	READ	B8081	Carcinoma in situ of intrahepatic bile ducts	
Cancer	READ	B8082	Carcinoma in situ of hepatic duct	
Cancer	READ	B8083	Carcinoma in situ of gall bladder	
Cancer	READ	B8085	Carcinoma in situ of common bile duct	
Cancer	READ	B8086	Carcinoma in situ of ampulla of Vater	
Cancer	READ	B8087	Carcinoma in situ of sphincter of Oddi	
Cancer	READ	B80z0	Carcinoma in situ of pancreas	

For peer review only

Cancer	READ	B903.	Neoplasm of uncertain behaviour of liver and biliary passage	
Cancer	READ	B9030	Neoplasm of uncertain behaviour of liver	
Cancer	READ	B9031	Neoplasm of uncertain behaviour of intra-hepatic bile ducts	
Cancer	READ	B9032	Neoplasm of uncertain behaviour of hepatic duct	
Cancer	READ	B9033	Neoplasm of uncertain behaviour of gall bladder	
Cancer	READ	B9034	Neoplasm of uncertain behaviour of cystic duct	
Cancer	READ	B9035	Neoplasm of uncertain behaviour of common bile duct	
Cancer	READ	B9036	Neoplasm of uncertain behaviour of ampulla of Vater	
Cancer	READ	B9037	Neoplasm of uncertain behaviour of sphincter of Oddi	
Cancer	READ	B9051	Neoplasm of uncertain behaviour of pancreas	
Cancer	READ	Byu10	[X]Other sarcomas of the liver	
Cancer	READ	Byu11	[X]Other specified carcinomas of liver	
Cancer	READ	Byu12	[X]Malignant neoplasm of intestinal tract, part unspecified	
Cancer	CTV3	B15..	Malignant neoplasm of liver and intrahepatic bile ducts	
Cancer	CTV3	B16..	Malignant tumour of biliary tract	
Cancer	CTV3	B162.	Malignant tumour of ampulla of Vater	
Cancer	CTV3	B17..	Malignant tumour of pancreas	B162. (ampullary tumour)<br>X78kd (metastasis to pancreas)
Cancer	CTV3	B80z0	Carcinoma in situ of pancreas	B8086 (ampullary carcinoma in situ)
Cancer	CTV3	B9030	Neoplasm of uncertain behaviour of liver	
Cancer	CTV3	B9031	Neoplasm of uncertain behaviour of intrahepatic bile ducts	
Cancer	CTV3	B9032	Neoplasm of uncertain behaviour of hepatic duct	
Cancer	CTV3	B9033	Neoplasm of uncertain behaviour of gallbladder	
Cancer	CTV3	B9034	Neoplasm of uncertain behaviour of cystic duct	
Cancer	CTV3	B9035	Neoplasm of uncertain behaviour of common bile duct	
Cancer	CTV3	B9036	Neoplasm of uncertain behaviour of ampulla of Vater	
Cancer	CTV3	B9037	Neoplasm of uncertain behaviour of sphincter of Oddi	
Cancer	CTV3	B903z	Neop of uncertain behaviour of liver or biliary passages NOS	
Cancer	CTV3	B9051	Neoplasm of uncertain behaviour of pancreas	
Cancer	CTV3	X78ed	Neoplasm of uncertain behaviour of biliary system	
Cancer	CTV3	X78mC	Carcinoma in situ of biliary tract	
Cancer	CTV3	Xa97q	Malignant tumour of liver	

For peer review only

Cancer	CTV3	XE2ve	Neoplasm of uncertain behaviour of liver and biliary passage	
Pancreatic disease	ICD-10	D136	Benign neoplasm of Pancreas excl. Endocrine pancreas	
Pancreatic disease	ICD-10	D137	Benign neoplasm of Endocrine pancreas	
Pancreatic disease	ICD-10	K85	Acute pancreatitis	
Pancreatic disease	ICD-10	K86	Other diseases of pancreas	
Pancreatic disease	ICD-10	K871	Disorders of pancreas in diseases classified elsewhere	
Pancreatic disease	SNOMED CT	3855007	Disorder of pancreas (disorder)	363418001,92672004,94978003 (cancer)
Pancreatic disease	READ	B716	Benign neoplasm of pancreas, excluding islets of Langerhans	
Pancreatic disease	READ	B717	Benign neoplasm of islets of Langerhans	
Pancreatic disease	READ	J67	Diseases of pancreas	
Pancreatic disease	CTV3	J67..	Disorder of pancreas	X309Y (tumour)
Pancreatic disease	CTV3	X78oE	Benign tumour of pancreas	
Liver disease	ICD-10	D134	Benign neoplasm of Liver	
Liver disease	ICD-10	K70	Alcoholic liver disease	
Liver disease	ICD-10	K71	Toxic liver disease	
Liver disease	ICD-10	K72	Hepatic failure, not elsewhere classified	
Liver disease	ICD-10	K73	Chronic hepatitis, not elsewhere classified	
Liver disease	ICD-10	K74	Fibrosis and cirrhosis of liver	
Liver disease	ICD-10	K75	Other inflammatory liver diseases	
Liver disease	ICD-10	K76	Other diseases of liver	
Liver disease	SNOMED CT	235856003	Disorder of liver (disorder)	93870000,92644006,94910002 (cancer)
Liver disease	READ	B715.	Benign neoplasm of liver and biliary ducts	
Liver disease	READ	B7150	Benign neoplasm of liver	
Liver disease	READ	B7151	Benign neoplasm of intrahepatic bile ducts	
Liver disease	READ	B7154	Benign neoplasm of hepatic duct	
Liver disease	READ	B7158	Focal nodular hyperplasia of liver	
Liver disease	READ	J60	Acute and subacute liver necrosis	
Liver disease	READ	J61	Cirrhosis and chronic liver disease	
Liver disease	READ	J62	Liver abscess and sequelae of chronic liver disease	
Liver disease	READ	J634	Hepatic infarction	
Liver disease	READ	J635	Toxic liver disease	
Liver disease	READ	J636	Central haemorrhagic necrosis of liver	

For peer review only

Liver disease	READ	J637	Hepatic veno-occlusive disease
Liver disease	READ	J638	Peliosis hepatis
Liver disease	READ	J639	Hepatic granulomas in berylliosis
Liver disease	READ	J63A	Hepatic granulomas in sarcoidosis
Liver disease	READ	J63X	Granulomatous hepatitis, not elsewhere classified
Liver disease	READ	J63y	Other specified liver disorder
Liver disease	READ	J63z.	Liver disorder NOS
Liver disease	CTV3	B715.	Benign neoplasm: [liver & biliary ducts] or [biliary system]
Liver disease	CTV3	B7150	Benign tumour of liver
Liver disease	CTV3	J614.	Chronic hepatitis
Liver disease	CTV3	J61y.	Other non-alcoholic chronic liver disease
Liver disease	CTV3	J61y3	Portal fibrosis without cirrhosis
Liver disease	CTV3	J61z.	Chronic liver disease NOS
Liver disease	CTV3	J62y.	(Hepat failure (& [NOS]) or (oth sequelae chronic liver dis)
Liver disease	CTV3	Jyu70	[X]Toxic liver disease with other disorders of liver
Liver disease	CTV3	Jyu71	[X]Other and unspecified cirrhosis of liver
Liver disease	CTV3	Jyu72	[X]Other specified inflammatory liver diseases
Liver disease	CTV3	Jyu76	[X]Toxic liver disease, unspecified
Liver disease	CTV3	Jyu77	[X]Granulomatous hepatitis, not elsewhere classified
Liver disease	CTV3	X306T	Inflammatory liver disease
Liver disease	CTV3	X3071	Alcoholic liver disease
Liver disease	CTV3	X307L	Cirrhosis of liver
Liver disease	CTV3	X307v	Fatty change of liver
Liver disease	CTV3	XaREa	Liver disease due to cystic fibrosis
Liver disease	CTV3	XE0bC	Other sequelae of chronic liver disease
Liver disease	CTV3	XE0dB	(Acute/subacute necrosis of liver) or (acute liver failure)
Liver disease	CTV3	XE0dD	(Cirrhos &/or chron liver dis) or (alcoholic liver disease)
Liver disease	CTV3	XE2xC	Benign neoplasm of liver and biliary ducts
Biliary disease	ICD-10	D135	Benign neoplasm of Extrahepatic bile ducts
Biliary disease	ICD-10	K80	Cholelithiasis
Biliary disease	ICD-10	K81	Cholecystitis
Biliary disease	ICD-10	K82	Other diseases of gallbladder

For peer review only

Biliary disease	ICD-10	K83	Other diseases of biliary tract	
Biliary disease	ICD-10	K870	Disorders of gallbladder and biliary tract in diseases classified elsewhere	
Biliary disease	SNOMED CT	105997008	Disorder of biliary tract (disorder)	363415003,92545000,255064003 (cancer)
Biliary disease	READ	B715.	Benign neoplasm of liver and biliary ducts	
Biliary disease	READ	B7152	Benign neoplasm of gallbladder	
Biliary disease	READ	B7155	Benign neoplasm of bile duct	
Biliary disease	READ	B7156	Benign neoplasm of sphincter of Oddi	
Biliary disease	READ	B7157	Benign neoplasm of ampulla of Vater	
Biliary disease	READ	J64	Cholelithiasis	
Biliary disease	READ	J65	Other gallbladder disorders	
Biliary disease	READ	J66	Other biliary tract disorders	
Biliary disease	CTV3	B715.	Benign neoplasm: [liver & biliary ducts] or [biliary system]	
Biliary disease	CTV3	X3081	Disorder of biliary tract	X308Y (tumour)
Biliary disease	CTV3	X78oB	Benign tumour of biliary tract	
Biliary disease	CTV3	XE2xC	Benign neoplasm of liver and biliary ducts	

Any incomplete ICD-10 code (less than four characters) or READ v2 code (less than five characters) implies inclusion of all codes starting with the prefix code.
Any CTV3 or SNOMED CT code implies inclusion of all children codes, excluding those in the Exclusion column.

For peer review only

Supplemental Table 2 Codelist for confirmed and suspected COVID-19 diagnosis

Group	Terminology system	Code	Code description
Confirmed	ICD-10	B342	Coronavirus infection, unspecified site
Confirmed	ICD-10	U049	Severe acute respiratory syndrome [SARS], unspecified
Confirmed	ICD-10	U071	COVID-19, virus identified
Confirmed	SNOMED CT	186747009	Coronavirus infection (disorder)
Suspected	ICD-10	U072	COVID-19, virus not identified

Any SNOMED CT code implies inclusion of all children codes, excluding those in the Exclusion column.

For peer review only

Supplemental Table 3 Risk ratio estimates of COVID-19 for HPB patients with specific demographic, comorbidity, lifestyle and medication use characteristics.

	Crude Risk Ratio (RR) (95% CI)	P value	Adjusted RR (95% CI)	P value	Adjusted RR (+all comorbidity) (95% CI)	P value	Adjusted RR (+diabetes) (95% CI)	P value	Adjusted RR (+hypertension) (95% CI)	P value
Demographics										
**Gender**										
Female										
Male	1.57 (1.2 to 2.06)	0.001	1.59 (1.21 to 2.09)	0.003	1.46 (1.11 to 1.92)	0.023	1.53 (1.17 to 2.02)	0.007	1.56 (1.18 to 2.05)	0.005
**Ethnic origin**										
White										
South Asian	1.24 (0.9 to 1.69)	0.184	1.49 (1.08 to 2.04)	0.025	1.19 (0.85 to 1.64)	0.436	1.17 (0.84 to 1.62)	0.505	1.45 (1.05 to 1.99)	0.042
Black	1.88 (1.28 to 2.72)	0.002	2.2 (1.5 to 3.18)	<0.001	2.01 (1.36 to 2.9)	0.001	1.92 (1.31 to 2.77)	0.003	2.11 (1.43 to 3.04)	<0.001
Other	0.69 (0.39 to 1.14)	0.184	0.82 (0.47 to 1.36)	0.525	0.83 (0.47 to 1.37)	0.58	0.74 (0.42 to 1.22)	0.447	0.84 (0.48 to 1.38)	0.642
**HPB diagnosis**										
Cancer	0.51 (0.13 to 1.33)	0.25	0.66 (0.16 to 1.84)	0.525	0.62 (0.15 to 1.72)	0.567	0.6 (0.14 to 1.68)	0.505	0.63 (0.15 to 1.75)	0.603
Non-cancer										
Pancreatic disease	1.48 (1.07 to 2.01)	0.015	1.61 (1.13 to 2.24)	0.015	1.36 (0.96 to 1.9)	0.174	1.48 (1.03 to 2.06)	0.058	1.51 (1.06 to 2.11)	0.04
Liver disease	1.21 (0.93 to 1.6)	0.16	1.55 (1.1 to 2.18)	0.024	1.32 (0.93 to 1.85)	0.206	1.44 (1.02 to 2.03)	0.068	1.47 (1.04 to 2.07)	0.045
Biliary disease	0.94 (0.72 to 1.23)	0.67	1.18 (0.84 to 1.62)	0.462	1.09 (0.78 to 1.5)	0.665	1.16 (0.83 to 1.59)	0.505	1.15 (0.82 to 1.58)	0.603
**Age group**										
18-40										
41-50	1.28 (0.72 to 2.29)	0.401	1.2 (0.68 to 2.14)	0.541	0.87 (0.49 to 1.58)	0.682	1.03 (0.58 to 1.86)	0.961	1.04 (0.59 to 1.87)	0.952
51-60	1.37 (0.81 to 2.39)	0.301	1.27 (0.74 to 2.22)	0.496	0.69 (0.39 to 1.24)	0.307	0.97 (0.57 to 1.71)	0.961	1 (0.58 to 1.76)	0.991
61-70	1.58 (0.93 to 2.75)	0.144	1.46 (0.85 to 2.56)	0.272	0.59 (0.33 to 1.09)	0.174	1.01 (0.58 to 1.81)	0.961	1.05 (0.6 to 1.88)	0.952
71-80	2.86 (1.73 to 4.9)	<0.001	3 (1.8 to 5.16)	<0.001	0.97 (0.54 to 1.79)	0.927	1.99 (1.17 to 3.49)	0.032	2.04 (1.19 to 3.6)	0.028
80+	4.96 (3.05 to 8.38)	<0.001	5.28 (3.23 to 8.95)	<0.001	1.48 (0.83 to 2.73)	0.307	3.5 (2.09 to 6.04)	<0.001	3.5 (2.08 to 6.12)	<0.001
Comorbidities										
**Diabetes**	3.13(2.37 to 4.18)	<0.001	2.42 (1.79 to 3.28)	<0.001	1.73 (1.26 to 2.38)	0.002			2.14 (1.58 to 2.93)	<0.001
**Hypertension**	3.36(2.32 to 5.07)	<0.001	2.43 (1.62 to 3.75)	<0.001	1.45 (0.94 to 2.3)	0.177	1.97 (1.31 to 3.07)	0.005		
**Cholesterol**	2.17(1.62 to 2.93)	<0.001	1.65 (1.22 to 2.26)	0.004	1.09 (0.8 to 1.52)	0.593	1.38 (1.01 to 1.9)	0.075	1.46 (1.08 to 2.01)	0.034
**Respiratory**	2.29(1.75 to 2.99)	<0.001	2.1 (1.6 to 2.76)	<0.001	1.76 (1.34 to 2.32)	<0.001	1.97 (1.5 to 2.59)	<0.001	2.01 (1.53 to 2.64)	<0.001
**Cardiovascular**	3.11(2.38 to 4.07)	<0.001	2.46 (1.83 to 3.31)	<0.001	1.62 (1.19 to 2.21)	0.006	2.12 (1.58 to 2.87)	<0.001	2.17 (1.61 to 2.93)	<0.001
**Kidney**	3.23(2.47 to 4.23)	<0.001	2.61 (1.97 to 3.47)	<0.001	1.97 (1.47 to 2.63)	<0.001	2.32 (1.75 to 3.09)	<0.001	2.38 (1.79 to 3.17)	<0.001
**Number of comorbidities**	1.65(1.52 to 1.8)	<0.001	1.6 (1.45 to 1.76)	<0.001						

For peer review only

Lifestyle factors										
**Smoker**										
Never										
Past	1.72 (1.29 to 2.3)	<0.001	1.47 (1.08 to 2.02)	0.024	1.17 (0.85 to 1.61)	0.425	1.4 (1.03 to 1.92)	0.045	1.44 (1.05 to 1.97)	0.031
Current	0.6 (0.37 to 0.92)	0.026	0.58 (0.35 to 0.93)	0.039	0.52 (0.31 to 0.83)	0.016	0.59 (0.35 to 0.95)	0.045	0.57 (0.34 to 0.91)	0.031
**Drinker**										
Never										
Past	1.63 (1.09 to 2.44)	0.024	1.43 (0.95 to 2.15)	0.114	1.19 (0.79 to 1.79)	0.506	1.33 (0.89 to 2)	0.251	1.35 (0.9 to 2.03)	0.188
Current	1.02 (0.73 to 1.46)	0.898	0.92 (0.63 to 1.34)	0.657	0.94 (0.65 to 1.37)	0.737	0.92 (0.63 to 1.34)	0.647	0.89 (0.62 to 1.31)	0.56
**Substance user**										
Never										
Past	4.1 (2.08 to 7.66)	<0.001	3.65 (1.81 to 7)	<0.001	2.59 (1.27 to 5.01)	0.018	3.4 (1.69 to 6.54)	0.001	3.4 (1.68 to 6.52)	0.001
Current	2.84 (1.88 to 4.41)	<0.001	2.5 (1.58 to 4.05)	<0.001	2 (1.26 to 3.26)	0.015	2.42 (1.53 to 3.93)	0.001	2.37 (1.5 to 3.84)	0.001
**Obese**										
Never										
Past	1.7 (1.1 to 2.76)	0.033	1.47 (0.95 to 2.38)	0.126	1.15 (0.74 to 1.87)	0.629	1.27 (0.82 to 2.07)	0.393	1.36 (0.88 to 2.2)	0.245
Current	1.47 (0.94 to 2.4)	0.108	1.47 (0.94 to 2.4)	0.126	1.05 (0.66 to 1.73)	0.845	1.2 (0.76 to 1.97)	0.514	1.29 (0.82 to 2.12)	0.324
Prescription medication use										
**ACE inhibitor**										
Non-user										
Past user	3.19 (2.04 to 4.76)	<0.001	2.32 (1.48 to 3.49)	<0.001	1.5 (0.96 to 2.26)	0.086	1.93 (1.22 to 2.9)	0.005	1.98 (1.26 to 2.98)	0.003
Current user	1.08 (0.71 to 1.57)	0.72	0.86 (0.57 to 1.26)	0.47	0.64 (0.42 to 0.94)	0.052	0.72 (0.47 to 1.05)	0.13	0.73 (0.48 to 1.07)	0.131
**Angiotensin receptor blocker**										
Non-user										
Past user	1.48 (0.53 to 3.2)	0.38	1.11 (0.4 to 2.4)	0.818	0.65 (0.23 to 1.42)	0.482	0.93 (0.33 to 2.02)	0.875	0.98 (0.35 to 2.12)	0.959
Current user	1.56 (1.01 to 2.31)	0.053	1.22 (0.78 to 1.82)	0.402	0.88 (0.56 to 1.31)	0.545	1.07 (0.69 to 1.6)	0.844	1.06 (0.68 to 1.58)	0.897
**Aldosterone agonist**										
Non-user										
Past user	3.29 (1.42 to 6.34)	0.002	2.58 (1.11 to 4.97)	0.019	1.65 (0.72 to 3.18)	0.277	2.37 (1.02 to 4.55)	0.033	2.41 (1.04 to 4.64)	0.029
Current user	1.72 (0.82 to 3.12)	0.109	1.39 (0.66 to 2.53)	0.347	0.9 (0.43 to 1.64)	0.757	1.3 (0.62 to 2.37)	0.474	1.28 (0.61 to 2.33)	0.464
**β-blocker**										
Non-user										
Past user	2.22 (1.2 to 3.72)	0.005	1.71 (0.92 to 2.88)	0.072	1.11 (0.6 to 1.88)	0.767	1.53 (0.83 to 2.57)	0.18	1.54 (0.84 to 2.6)	0.147
Current user	2.13 (1.54 to 2.89)	<0.001	1.6 (1.15 to 2.2)	0.007	1.05 (0.75 to 1.47)	0.767	1.42 (1.02 to 1.95)	0.053	1.44 (1.03 to 1.97)	0.043
**Calcium channel blocker**										

For peer review only

Non-user										
Past user	1.96 (1.13 to 3.16)	0.014	1.36 (0.78 to 2.21)	0.32	0.9 (0.52 to 1.46)	0.695	1.2 (0.69 to 1.94)	0.494	1.14 (0.66 to 1.85)	0.612
Current user	1.23 (0.85 to 1.72)	0.258	0.87 (0.6 to 1.24)	0.467	0.69 (0.48 to 0.98)	0.069	0.79 (0.54 to 1.12)	0.252	0.72 (0.5 to 1.02)	0.099
**Antiplatelet**										
Non-user										
Past user	3.12 (1.86 to 4.89)	<0.001	2.2 (1.3 to 3.5)	0.003	1.32 (0.77 to 2.11)	0.438	1.84 (1.09 to 2.93)	0.023	1.98 (1.17 to 3.15)	0.01
Current user	2.3 (1.63 to 3.18)	<0.001	1.66 (1.16 to 2.33)	0.006	1 (0.69 to 1.42)	0.982	1.42 (0.99 to 2)	0.061	1.48 (1.03 to 2.08)	0.037
**Antiarrhythmic**										
Non-user										
Past user	3.05 (0.52 to 9.13)	0.11	2.43 (0.41 to 7.22)	0.234	1.3 (0.22 to 3.8)	0.71	2.08 (0.35 to 6.16)	0.332	2.24 (0.38 to 6.68)	0.28
Current user	2.43 (0.76 to 5.6)	0.11	2.07 (0.65 to 4.79)	0.191	1.32 (0.41 to 3.06)	0.622	1.88 (0.59 to 4.34)	0.268	1.94 (0.61 to 4.48)	0.235
**Anticoagulant**										
Non-user										
Past user	2.64 (0.95 to 5.66)	0.029	2.11 (0.76 to 4.52)	0.124	1.46 (0.53 to 3.11)	0.552	2.05 (0.74 to 4.37)	0.16	1.95 (0.7 to 4.18)	0.17
Current user	2.07 (1.07 to 3.58)	0.026	1.62 (0.83 to 2.81)	0.136	1.05 (0.54 to 1.83)	0.881	1.49 (0.77 to 2.6)	0.23	1.51 (0.78 to 2.63)	0.203
**Glucocorticoid**										
Non-user										
Past user	1.63 (1.01 to 2.5)	0.034	1.47 (0.91 to 2.25)	0.112	1.09 (0.68 to 1.69)	0.698	1.35 (0.84 to 2.08)	0.217	1.4 (0.87 to 2.16)	0.159
Current user	2.35 (1.74 to 3.14)	<0.001	2.04 (1.51 to 2.74)	<0.001	1.35 (0.97 to 1.86)	0.126	1.85 (1.37 to 2.49)	<0.001	1.91 (1.41 to 2.57)	<0.001
**β2 to agonist**										
Non-user										
Past user	2.28 (1.04 to 4.28)	0.021	2.12 (0.97 to 3.98)	0.039	1.36 (0.61 to 2.59)	0.51	1.97 (0.9 to 3.69)	0.072	1.98 (0.9 to 3.71)	0.062
Current user	2.21 (1.53 to 3.1)	<0.001	1.91 (1.32 to 2.69)	0.001	1.18 (0.79 to 1.73)	0.51	1.75 (1.21 to 2.46)	0.004	1.81 (1.25 to 2.55)	0.002
**Muscarinic antagonist**										
Non-user										
Past user	2.27 (1.08 to 4.14)	0.015	1.91 (0.91 to 3.48)	0.062	1.34 (0.64 to 2.45)	0.483	1.75 (0.84 to 3.19)	0.122	1.83 (0.88 to 3.33)	0.081
Current user	2.22 (1.51 to 3.16)	<0.001	1.8 (1.22 to 2.58)	0.003	1.22 (0.82 to 1.77)	0.479	1.69 (1.15 to 2.42)	0.009	1.69 (1.14 to 2.42)	0.009
**NSAID**										
Non-user										
Past user	1.14 (0.59 to 1.99)	0.667	1 (0.51 to 1.74)	0.998	0.93 (0.48 to 1.62)	0.819	0.98 (0.51 to 1.71)	0.953	0.96 (0.49 to 1.68)	0.9
Current user	1.65 (0.96 to 2.65)	0.077	1.55 (0.9 to 2.49)	0.119	1.48 (0.86 to 2.36)	0.201	1.51 (0.87 to 2.42)	0.168	1.5 (0.87 to 2.4)	0.154
**Vitamin D**										
Non-user										
Past user	2.93 (1.79 to 4.53)	<0.001	2.55 (1.55 to 3.95)	<0.001	1.9 (1.16 to 2.95)	0.01	2.28 (1.39 to 3.54)	0.001	2.39 (1.45 to 3.7)	<0.001
Current user	2.64 (1.89 to 3.62)	<0.001	2.26 (1.6 to 3.12)	<0.001	1.66 (1.18 to 2.29)	0.007	2.07 (1.47 to 2.86)	<0.001	2.11 (1.5 to 2.91)	<0.001

For peer review only

**Proton pump inhibitor**										
Non-user										
Past user	1.5 (0.93 to 2.32)	0.08	1.31 (0.81 to 2.04)	0.278	1.01 (0.63 to 1.57)	0.951	1.22 (0.75 to 1.89)	0.443	1.24 (0.77 to 1.93)	0.388
Current user	1.8 (1.36 to 2.39)	<0.001	1.53 (1.14 to 2.04)	0.006	1.13 (0.84 to 1.51)	0.545	1.37 (1.03 to 1.84)	0.047	1.4 (1.05 to 1.88)	0.032
**Statin**										
Non-user										
Past user	2.95 (1.89 to 4.43)	<0.001	2.1 (1.33 to 3.21)	0.002	1.28 (0.81 to 1.97)	0.432	1.65 (1.04 to 2.53)	0.039	1.85 (1.17 to 2.82)	0.009
Current user	1.88 (1.41 to 2.51)	<0.001	1.42 (1.04 to 1.92)	0.033	0.92 (0.67 to 1.27)	0.623	1.12 (0.82 to 1.52)	0.543	1.23 (0.9 to 1.67)	0.21
**Immunosuppressant**										
Non-user										
Past user	1.6 (0.5 to 3.72)	0.346	1.32 (0.41 to 3.07)	0.575	1.07 (0.34 to 2.48)	0.886	1.31 (0.41 to 3.02)	0.594	1.25 (0.39 to 2.91)	0.652
Current user	2.26 (1.03 to 4.22)	0.033	2.01 (0.92 to 3.75)	0.066	1.63 (0.75 to 3.03)	0.26	1.84 (0.84 to 3.42)	0.13	1.92 (0.88 to 3.58)	0.086

	Adjusted RR (+cholesterol) (95% CI)	P value	Adjusted RR (+respiratory) (95% CI)	P value	Adjusted RR (+cardiovascular) (95% CI)	P value	Adjusted RR (+kidney) (95% CI)	P value
Demographics								
**Gender**								
Female								
Male	1.56 (1.19 to 2.06)	0.005	1.6 (1.22 to 2.11)	0.002	1.45 (1.1 to 1.91)	0.025	1.56 (1.19 to 2.05)	0.004
**Ethnic origin**								
White								
South Asian	1.41 (1.02 to 1.94)	0.061	1.5 (1.09 to 2.06)	0.022	1.45 (1.05 to 1.99)	0.038	1.43 (1.04 to 1.97)	0.044
Black	2.23 (1.52 to 3.21)	<0.001	2.33 (1.59 to 3.36)	<0.001	2.15 (1.46 to 3.09)	<0.001	2.14 (1.46 to 3.08)	<0.001
Other	0.82 (0.46 to 1.35)	0.629	0.87 (0.49 to 1.44)	0.609	0.84 (0.47 to 1.38)	0.668	0.82 (0.46 to 1.35)	0.598
**HPB diagnosis**								
Cancer	0.67 (0.16 to 1.87)	0.634	0.65 (0.16 to 1.82)	0.599	0.69 (0.16 to 1.92)	0.668	0.65 (0.16 to 1.82)	0.598
Non-cancer								
Pancreatic disease	1.55 (1.09 to 2.17)	0.026	1.55 (1.09 to 2.17)	0.022	1.56 (1.1 to 2.18)	0.027	1.54 (1.08 to 2.15)	0.028
Liver disease	1.5 (1.06 to 2.11)	0.039	1.5 (1.06 to 2.1)	0.036	1.5 (1.06 to 2.1)	0.038	1.48 (1.05 to 2.08)	0.044
Biliary disease	1.15 (0.83 to 1.59)	0.595	1.17 (0.84 to 1.61)	0.475	1.15 (0.82 to 1.58)	0.624	1.14 (0.82 to 1.57)	0.598
**Age group**								
18-40								
41-50	1.07 (0.6 to 1.93)	0.821	1.18 (0.67 to 2.12)	0.602	1.11 (0.63 to 1.99)	0.837	1.11 (0.62 to 1.98)	0.733
51-60	1.08 (0.62 to 1.9)	0.821	1.19 (0.7 to 2.08)	0.602	1.06 (0.62 to 1.86)	0.837	1.12 (0.66 to 1.97)	0.726

For peer review only

For peer review only

61-70	1.19 (0.68 to 2.12)	0.642	1.31 (0.76 to 2.31)	0.475	1.06 (0.61 to 1.9)	0.837	1.16 (0.67 to 2.04)	0.704
71-80	2.39 (1.4 to 4.2)	0.005	2.6 (1.55 to 4.48)	0.001	1.97 (1.14 to 3.48)	0.036	2.11 (1.24 to 3.68)	0.017
80+	4.18 (2.49 to 7.25)	<0.001	4.56 (2.79 to 7.75)	<0.001	3.17 (1.85 to 5.59)	<0.001	3.3 (1.96 to 5.74)	<0.001
Comorbidities								
**Diabetes**	2.25 (1.66 to 3.08)	<0.001	2.28 (1.69 to 3.09)	<0.001	2.09 (1.54 to 2.85)	<0.001	2.12 (1.56 to 2.89)	<0.001
**Hypertension**	2.22 (1.48 to 3.45)	0.001	2.27 (1.52 to 3.51)	<0.001	1.97 (1.3 to 3.09)	0.006	2.04 (1.36 to 3.17)	0.003
**Cholesterol**			1.55 (1.14 to 2.13)	0.011	1.43 (1.05 to 1.97)	0.043	1.47 (1.08 to 2.01)	0.033
**Respiratory**	2.03 (1.54 to 2.67)	<0.001			1.91 (1.46 to 2.52)	<0.001	1.97 (1.5 to 2.59)	<0.001
**Cardiovascular**	2.31 (1.72 to 3.12)	<0.001	2.26 (1.68 to 3.05)	<0.001			2.06 (1.53 to 2.79)	<0.001
**Kidney**	2.49 (1.87 to 3.31)	<0.001	2.48 (1.87 to 3.29)	<0.001	2.25 (1.68 to 3.01)	<0.001		
**Number of comorbidities**								
Lifestyle factors								
**Smoker**								
Never								
Past	1.41 (1.03 to 1.93)	0.04	1.31 (0.96 to 1.81)	0.102	1.36 (1 to 1.87)	0.069	1.38 (1.01 to 1.89)	0.054
Current	0.57 (0.34 to 0.91)	0.037	0.5 (0.3 to 0.81)	0.009	0.56 (0.33 to 0.89)	0.029	0.6 (0.36 to 0.97)	0.054
**Drinker**								
Never								
Past	1.37 (0.91 to 2.06)	0.169	1.35 (0.9 to 2.04)	0.187	1.36 (0.9 to 2.04)	0.177	1.36 (0.91 to 2.05)	0.172
Current	0.89 (0.62 to 1.31)	0.557	0.91 (0.63 to 1.34)	0.676	0.96 (0.66 to 1.41)	0.839	0.94 (0.65 to 1.38)	0.759
**Substance user**								
Never								
Past	3.52 (1.74 to 6.77)	<0.001	3.24 (1.6 to 6.22)	0.001	3.16 (1.56 to 6.1)	0.002	3.21 (1.59 to 6.15)	0.001
Current	2.44 (1.55 to 3.97)	<0.001	2.26 (1.43 to 3.69)	0.001	2.23 (1.4 to 3.64)	0.002	2.36 (1.5 to 3.81)	0.001
**Obese**								
Never								
Past	1.37 (0.88 to 2.23)	0.229	1.45 (0.94 to 2.36)	0.139	1.41 (0.91 to 2.28)	0.183	1.35 (0.87 to 2.19)	0.223
Current	1.36 (0.86 to 2.23)	0.231	1.41 (0.9 to 2.31)	0.171	1.39 (0.88 to 2.27)	0.196	1.36 (0.87 to 2.23)	0.223
Prescription medication use								
**ACE inhibitor**								
Non-user								
Past user	2.14 (1.36 to 3.22)	0.001	2.21 (1.41 to 3.32)	<0.001	1.91 (1.21 to 2.87)	0.006	1.96 (1.25 to 2.95)	0.003
Current user	0.8 (0.53 to 1.17)	0.31	0.86 (0.57 to 1.26)	0.508	0.76 (0.5 to 1.11)	0.198	0.79 (0.52 to 1.16)	0.287
**Angiotensin receptor blocker**								

Non-user								
Past user	1.03 (0.37 to 2.23)	0.952	1.01 (0.36 to 2.19)	0.979	0.89 (0.32 to 1.93)	0.799	0.88 (0.32 to 1.92)	0.784
Current user	1.14 (0.73 to 1.7)	0.606	1.17 (0.75 to 1.74)	0.547	1.07 (0.69 to 1.6)	0.799	1.07 (0.69 to 1.6)	0.784
**Aldosterone agonist**								
Non-user								
Past user	2.47 (1.06 to 4.75)	0.025	2.47 (1.06 to 4.74)	0.02	1.96 (0.85 to 3.79)	0.094	2.1 (0.91 to 4.03)	0.062
Current user	1.33 (0.63 to 2.42)	0.403	1.27 (0.61 to 2.31)	0.5	1.04 (0.49 to 1.9)	0.911	1.17 (0.56 to 2.13)	0.644
**β to blocker**								
Non-user								
Past user	1.58 (0.85 to 2.67)	0.127	1.71 (0.93 to 2.87)	0.071	1.26 (0.68 to 2.15)	0.422	1.43 (0.78 to 2.42)	0.239
Current user	1.5 (1.08 to 2.07)	0.021	1.58 (1.13 to 2.16)	0.009	1.18 (0.83 to 1.64)	0.422	1.39 (1 to 1.92)	0.067
**Calcium channel blocker**								
Non-user								
Past user	1.25 (0.72 to 2.04)	0.388	1.29 (0.74 to 2.1)	0.418	1.15 (0.66 to 1.86)	0.596	1.13 (0.65 to 1.83)	0.649
Current user	0.83 (0.57 to 1.18)	0.388	0.87 (0.6 to 1.24)	0.502	0.83 (0.57 to 1.17)	0.379	0.8 (0.55 to 1.13)	0.284
**Antiplatelet**								
Non-user								
Past user	2.01 (1.19 to 3.21)	0.008	2.04 (1.2 to 3.24)	0.007	1.56 (0.91 to 2.51)	0.109	1.89 (1.12 to 3)	0.018
Current user	1.52 (1.06 to 2.14)	0.026	1.54 (1.07 to 2.16)	0.021	1.13 (0.77 to 1.62)	0.526	1.45 (1.01 to 2.04)	0.046
**Antiarrhythmic**								
Non-user								
Past user	2.27 (0.38 to 6.72)	0.271	2.1 (0.36 to 6.15)	0.323	1.76 (0.3 to 5.26)	0.428	1.79 (0.3 to 5.3)	0.405
Current user	2.01 (0.63 to 4.63)	0.209	1.85 (0.58 to 4.28)	0.275	1.49 (0.46 to 3.45)	0.428	1.75 (0.54 to 4.02)	0.339
**Anticoagulant**								
Non-user								
Past user	2 (0.72 to 4.28)	0.154	1.96 (0.71 to 4.17)	0.168	1.62 (0.58 to 3.48)	0.359	1.8 (0.65 to 3.85)	0.238
Current user	1.52 (0.78 to 2.65)	0.196	1.49 (0.76 to 2.58)	0.222	1.23 (0.63 to 2.14)	0.512	1.31 (0.68 to 2.29)	0.374
**Glucocorticoid**								
Non-user								
Past user	1.41 (0.88 to 2.17)	0.152	1.24 (0.76 to 1.92)	0.404	1.36 (0.84 to 2.09)	0.206	1.36 (0.85 to 2.1)	0.201
Current user	1.93 (1.42 to 2.6)	<0.001	1.53 (1.1 to 2.13)	0.016	1.88 (1.39 to 2.52)	<0.001	1.88 (1.39 to 2.53)	<0.001
**β2-agonist**								
Non-user								
Past user	2.05 (0.93 to 3.84)	0.057	1.42 (0.64 to 2.72)	0.379	1.88 (0.86 to 3.52)	0.086	2.04 (0.93 to 3.8)	0.051
Current user	1.81 (1.25 to 2.55)	0.002	1.24 (0.83 to 1.83)	0.363	1.73 (1.19 to 2.44)	0.006	1.82 (1.26 to 2.56)	0.002

For peer review only

**Muscarinic antagonist**								
Non-user								
Past user	1.82 (0.87 to 3.32)	0.083	1.56 (0.74 to 2.85)	0.214	1.72 (0.82 to 3.13)	0.121	1.76 (0.84 to 3.19)	0.103
Current user	1.73 (1.17 to 2.48)	0.006	1.32 (0.88 to 1.94)	0.205	1.59 (1.08 to 2.28)	0.023	1.69 (1.15 to 2.43)	0.009
**NSAID**								
Non-user								
Past user	0.98 (0.5 to 1.7)	0.944	0.95 (0.49 to 1.65)	0.861	0.99 (0.51 to 1.72)	0.968	0.99 (0.51 to 1.73)	0.98
Current user	1.51 (0.87 to 2.42)	0.146	1.48 (0.86 to 2.37)	0.166	1.54 (0.89 to 2.46)	0.123	1.55 (0.9 to 2.48)	0.115
**Vitamin D**								
Non-user								
Past user	2.37 (1.44 to 3.69)	<0.001	2.38 (1.45 to 3.69)	<0.001	2.34 (1.43 to 3.63)	0.001	2.21 (1.34 to 3.42)	0.001
Current user	2.14 (1.52 to 2.97)	<0.001	2.03 (1.44 to 2.82)	<0.001	2.03 (1.44 to 2.81)	<0.001	1.98 (1.41 to 2.74)	<0.001
**Proton pump inhibitor**								
Non-user								
Past user	1.23 (0.76 to 1.91)	0.382	1.23 (0.76 to 1.91)	0.421	1.22 (0.75 to 1.89)	0.395	1.17 (0.72 to 1.82)	0.497
Current user	1.42 (1.06 to 1.9)	0.029	1.39 (1.04 to 1.86)	0.038	1.36 (1.02 to 1.82)	0.057	1.4 (1.05 to 1.87)	0.034
**Statin**								
Non-user								
Past user	1.8 (1.13 to 2.78)	0.016	1.93 (1.22 to 2.94)	0.005	1.77 (1.12 to 2.71)	0.019	1.81 (1.14 to 2.76)	0.012
Current user	1.22 (0.89 to 1.68)	0.254	1.35 (0.99 to 1.82)	0.063	1.21 (0.89 to 1.65)	0.242	1.27 (0.93 to 1.72)	0.144
**Immunosuppressant**								
Non-user								
Past user	1.26 (0.39 to 2.92)	0.643	1.21 (0.38 to 2.81)	0.698	1.21 (0.38 to 2.8)	0.703	1.21 (0.38 to 2.8)	0.7
Current user	1.97 (0.9 to 3.67)	0.085	1.85 (0.84 to 3.44)	0.108	1.94 (0.89 to 3.62)	0.079	1.83 (0.84 to 3.4)	0.113

Risk ratios, except the crude ones, are mutually adjusted for gender, ethnicity, age group and HPB diagnosis, and also for additional conditions when mentioned inside the parenthesis. Simplified binary age group (over and under 60) are used for adjustment for all categories except demographics. All P values presented, except for the crude risk ratios, are Benjamini-Hochberg corrected.

For peer review only

Supplemental Table 4 Risk ratio estimates of COVID-19 related death for HPB patients with specific demographic, comorbidity, lifestyle, medication use and post COVID-19 diagnosis complication characteristics.

	Crude Risk Ratio (RR) (95% CI)	P value	Adjusted RR (95% CI)	P value	Adjusted RR (+all comorbidity) (95% CI)	P value	Adjusted RR (+diabetes) (95% CI)	P value	Adjusted RR (+hypertension) (95% CI)	P value
Demographics										
**Gender**										
Female										
Male	1.38 (0.85 to 2.08)	0.18	1.66 (1 to 2.5)	0.156	1.61 (0.94 to 2.48)	0.385	1.71 (1.03 to 2.57)	0.142	1.69 (1.02 to 2.54)	0.143
**Ethnic origin**										
White										
South Asian	1.42 (0.8 to 2.26)	0.212	1.7 (0.94 to 2.68)	0.182	1.52 (0.79 to 2.53)	0.4	1.51 (0.79 to 2.49)	0.354	1.67 (0.91 to 2.66)	0.236
Black	1.86 (1.03 to 2.85)	0.077	2.4 (1.35 to 3.48)	0.035	2.2 (1.14 to 3.37)	0.223	2.3 (1.26 to 3.41)	0.079	2.41 (1.35 to 3.5)	0.044
Other	0.3 (0.02 to 1.29)	0.212	0.41 (0.02 to 1.76)	0.446	0.33 (0.02 to 1.58)	0.413	0.36 (0.02 to 1.63)	0.401	0.4 (0.02 to 1.74)	0.449
**HPB diagnosis**										
Cancer	2.68 (0.64 to 3.93)	0.15	2.69 (0.55 to 3.91)	0.272	3.32 (0.93 to 3.95)	0.385	3.12 (0.8 to 3.94)	0.195	2.55 (0.47 to 3.89)	0.361
Non-cancer										
Pancreatic disease	1.28 (0.74 to 1.96)	0.350	1.51 (0.81 to 2.36)	0.272	1.44 (0.74 to 2.33)	0.413	1.46 (0.78 to 2.32)	0.354	1.43 (0.76 to 2.28)	0.369
Liver disease	0.74 (0.44 to 1.15)	0.195	1.16 (0.62 to 1.84)	0.612	1.13 (0.58 to 1.84)	0.812	1.16 (0.62 to 1.85)	0.686	1.1 (0.57 to 1.79)	0.754
Biliary disease	1.45 (0.91 to 2.15)	0.110	1.36 (0.72 to 2.26)	0.446	1.4 (0.72 to 2.35)	0.421	1.4 (0.73 to 2.31)	0.401	1.33 (0.7 to 2.23)	0.449
**Age group**										
18-40										
41-50	1.32 (0.22 to 5.8)	0.780	1.86 (0.3 to 7.09)	0.535	1.54 (0.21 to 6.85)	0.812	1.53 (0.23 to 6.58)	0.686	2.11 (0.34 to 7.57)	0.469
51-60	1.26 (0.25 to 5.48)	0.780	1.77 (0.33 to 6.76)	0.535	1.36 (0.22 to 6.26)	0.819	1.42 (0.25 to 6.17)	0.686	1.88 (0.35 to 7.04)	0.493
61-70	2.51 (0.68 to 7.46)	0.297	2.92 (0.73 to 8.08)	0.272	1.83 (0.34 to 7.05)	0.65	2.07 (0.44 to 7.18)	0.462	2.89 (0.69 to 8.14)	0.313
71-80	4.45 (1.58 to 9.04)	0.046	5.27 (1.87 to 9.57)	0.035	3.1 (0.71 to 8.52)	0.4	3.94 (1.11 to 8.93)	0.142	4.86 (1.59 to 9.42)	0.083
80+	4.15 (1.47 to 8.82)	0.046	5.17 (1.84 to 9.51)	0.035	3.21 (0.76 to 8.55)	0.4	4.23 (1.29 to 9.07)	0.142	4.62 (1.49 to 9.27)	0.083
Comorbidity										
**Diabetes**	2.22 (1.28 to 3.58)	0.009	2 (1.03 to 3.5)	0.114	1.71 (0.83 to 3.19)	0.298			1.96 (1 to 3.46)	0.139
**Hypertension**	8.4 (2.24 to 25.53)	0.018	5.58 (1.2 to 23.1)	0.169	4.67 (0.89 to 22.33)	0.296	5.48 (1.15 to 23.05)	0.167		
**Cholesterol**	1.45 (0.84 to 2.34)	0.190	1.13 (0.59 to 2.01)	0.765	0.96 (0.47 to 1.82)	0.917	1.07 (0.54 to 1.93)	0.84	1.04 (0.53 to 1.89)	0.908
**Respiratory**	0.89 (0.54 to 1.38)	0.630	0.7 (0.39 to 1.17)	0.293	0.74 (0.39 to 1.27)	0.432	0.77 (0.42 to 1.27)	0.391	0.72 (0.39 to 1.2)	0.331
**Cardiovascular**	1.77 (1.09 to 2.66)	0.024	1.27 (0.68 to 2.16)	0.477	0.93 (0.43 to 1.82)	0.917	1.22 (0.65 to 2.1)	0.568	1.18 (0.62 to 2.04)	0.659
**Kidney**	2.71 (1.68 to 4.06)	<0.001	2.13 (1.16 to 3.55)	0.053	1.98 (0.98 to 3.55)	0.232	2.01 (1.08 to 3.42)	0.130	1.96 (1.05 to 3.34)	0.118
**Number of comorbidities**	1.35 (1.15 to 1.57)	<0.001	1.2 (0.99 to 1.45)	0.157						

For peer review only

Lifestyle factors										
**Smoker**										
Never										
Past	1.61 (1 to 2.4)	0.082	1.42 (0.77 to 2.34)	0.421	1.36 (0.7 to 2.31)	0.443	1.39 (0.74 to 2.32)	0.423	1.37 (0.74 to 2.28)	0.435
Current	0.39 (0.06 to 1.23)	0.167	0.48 (0.07 to 1.62)	0.421	0.48 (0.07 to 1.69)	0.443	0.47 (0.07 to 1.62)	0.423	0.49 (0.07 to 1.68)	0.435
**Drinker**										
Never										
Past	1.16 (0.54 to 2.13)	0.679	1.21 (0.5 to 2.35)	0.642	0.99 (0.36 to 2.13)	0.978	1.09 (0.43 to 2.2)	0.842	1.17 (0.48 to 2.3)	0.713
Current	1.21 (0.64 to 2.08)	0.679	1.53 (0.74 to 2.64)	0.419	1.69 (0.77 to 2.94)	0.328	1.48 (0.7 to 2.6)	0.412	1.39 (0.65 to 2.51)	0.616
**Substance user**										
Never										
Past	0.74 (0.11 to 2.35)	0.690	0.53 (0.06 to 2.19)	0.550	0.52 (0.05 to 2.53)	0.691	0.59 (0.07 to 2.42)	0.598	0.47 (0.06 to 2.08)	0.517
Current	1.74 (0.85 to 3.03)	0.210	1.6 (0.59 to 3.16)	0.550	1.44 (0.39 to 3.24)	0.691	1.6 (0.57 to 3.18)	0.458	1.41 (0.47 to 3.01)	0.595
**Obese**										
Never										
Past	1.19 (0.52 to 2.41)	0.943	1.13 (0.43 to 2.46)	0.872	0.76 (0.24 to 2.01)	0.740	1.01 (0.37 to 2.31)	0.98	1.06 (0.38 to 2.39)	0.986
Current	1.03 (0.42 to 2.23)	0.943	1 (0.35 to 2.34)	0.993	0.74 (0.23 to 2.01)	0.740	0.87 (0.29 to 2.15)	0.845	1 (0.34 to 2.35)	0.999
Prescription medication use										
**ACE inhibitor**										
Non-user										
Past user	1.81 (0.91 to 2.87)	0.074	1.42 (0.64 to 2.53)	0.395	1.24 (0.51 to 2.37)	0.722	1.28 (0.55 to 2.36)	0.573	1.35 (0.59 to 2.45)	0.468
Current user	1.99 (1.12 to 2.98)	0.029	2.1 (1.12 to 3.2)	0.071	1.99 (1.01 to 3.14)	0.199	2.04 (1.07 to 3.15)	0.102	1.93 (1 to 3.04)	0.162
**Angiotensin receptor blocker**										
Non-user										
Past user	2.48 (0.8 to 3.81)	0.140	1.87 (0.42 to 3.61)	0.465	1.92 (0.43 to 3.64)	0.509	1.69 (0.36 to 3.52)	0.537	1.76 (0.39 to 3.55)	0.521
Current user	1.16 (0.52 to 2.02)	0.680	1.23 (0.5 to 2.26)	0.654	1.15 (0.45 to 2.19)	0.846	1.16 (0.46 to 2.18)	0.778	1.19 (0.48 to 2.22)	0.714
**Aldosterone agonist**										
Non-user										
Past user	1.14 (0.21 to 2.63)	0.830	1.06 (0.18 to 2.61)	0.935	0.94 (0.15 to 2.53)	0.934	1.13 (0.2 to 2.72)	0.849	0.98 (0.17 to 2.51)	0.973
Current user	1.33 (0.37 to 2.65)	0.830	1.48 (0.36 to 2.95)	0.589	1.12 (0.22 to 2.71)	0.934	1.36 (0.31 to 2.86)	0.709	1.44 (0.35 to 2.94)	0.622
**Β-blocker**										
Non-user										
Past user	1.23 (0.41 to 2.37)	0.940	1.08 (0.31 to 2.34)	0.887	0.67 (0.16 to 1.8)	0.589	0.91 (0.24 to 2.14)	0.86	0.99 (0.28 to 2.22)	0.989
Current user	1.02 (0.55 to 1.65)	0.940	0.81 (0.39 to 1.45)	0.627	0.59 (0.25 to 1.19)	0.316	0.76 (0.36 to 1.38)	0.479	0.74 (0.35 to 1.36)	0.483
**Calcium channel blocker**										
Non-user										

For peer review only

Past user	0.8 (0.21 to 1.85)	0.670	0.44 (0.1 to 1.29)	0.362	0.33 (0.07 to 1.09)	0.281	0.4 (0.08 to 1.22)	0.272	0.37 (0.08 to 1.15)	0.254
Current user	1.57 (0.91 to 2.36)	0.140	1.4 (0.72 to 2.28)	0.384	1.13 (0.54 to 2.01)	0.861	1.33 (0.67 to 2.22)	0.463	1.27 (0.64 to 2.14)	0.547
**Antiplatelet**										
Non-user										
Past user	1.31 (0.49 to 2.48)	0.525	1.05 (0.35 to 2.22)	0.920	1.26 (0.41 to 2.61)	0.813	1.07 (0.36 to 2.25)	0.885	1.03 (0.34 to 2.2)	0.953
Current user	1.87 (1.13 to 2.71)	0.025	1.32 (0.67 to 2.22)	0.509	1.15 (0.53 to 2.1)	0.813	1.27 (0.64 to 2.17)	0.541	1.33 (0.67 to 2.24)	0.5
**Antiarrhythmic**										
Non-user										
Past user	1.98 (0.15 to 3.81)	0.670	2.35 (0.16 to 3.85)	0.485	3.24 (0.2 to 3.92)	0.484	2.41 (0.14 to 3.87)	0.468	3.05 (0.21 to 3.91)	0.484
Current user	0.99 (0.06 to 2.88)	0.990	1.06 (0.07 to 3)	0.946	1.67 (0.12 to 3.48)	0.657	1.67 (0.12 to 3.46)	0.586	1 (0.06 to 2.94)	0.996
**Anticoagulant**										
Non-user										
Past user	0.8 (0.05 to 2.54)	0.800	0.35 (0.01 to 1.96)	0.465	0.28 (0.01 to 1.69)	0.435	0.34 (0.01 to 1.83)	0.417	0.33 (0.01 to 1.87)	0.448
Current user	1.45 (0.51 to 2.66)	0.610	1.1 (0.32 to 2.38)	0.860	1.05 (0.28 to 2.46)	0.97	1.12 (0.32 to 2.44)	0.833	1.08 (0.31 to 2.42)	0.923
**Glucocorticoid**										
Non-user										
Past user	1.44 (0.64 to 2.49)	0.330	1.2 (0.47 to 2.3)	0.729	1.22 (0.45 to 2.42)	0.79	1.17 (0.45 to 2.28)	0.775	1.13 (0.44 to 2.23)	0.84
Current user	1.33 (0.79 to 2.03)	0.330	1.24 (0.67 to 2.03)	0.562	1.28 (0.66 to 2.16)	0.553	1.21 (0.64 to 2)	0.623	1.21 (0.65 to 2.01)	0.613
**β2-agonist**										
Non-user										
Past user	1.61 (0.45 to 3.08)	0.370	1.51 (0.38 to 3.14)	0.578	2.1 (0.55 to 3.65)	0.386	1.58 (0.4 to 3.19)	0.51	1.58 (0.38 to 3.27)	0.545
Current user	1.39 (0.76 to 2.17)	0.370	1.19 (0.57 to 2.05)	0.671	1.56 (0.71 to 2.67)	0.395	1.27 (0.6 to 2.19)	0.533	1.18 (0.56 to 2.06)	0.683
**Muscarinic antagonist**										
Non-user										
Past user	1.32 (0.37 to 2.63)	0.890	1.15 (0.27 to 2.59)	0.810	0.94 (0.2 to 2.42)	0.925	1.15 (0.26 to 2.6)	0.821	1.08 (0.25 to 2.53)	0.897
Current user	0.96 (0.45 to 1.68)	0.900	0.86 (0.36 to 1.64)	0.808	0.88 (0.35 to 1.76)	0.925	0.9 (0.37 to 1.72)	0.821	0.86 (0.36 to 1.64)	0.808
**NSAID**										
Non-user										
Past user	0.73 (0.13 to 1.91)	0.620	0.53 (0.08 to 1.6)	0.378	0.63 (0.1 to 1.92)	0.614	0.58 (0.09 to 1.74)	0.473	0.5 (0.08 to 1.55)	0.372
Current user	1.51 (0.66 to 2.54)	0.410	1.56 (0.6 to 2.75)	0.378	1.71 (0.63 to 2.97)	0.399	1.64 (0.63 to 2.84)	0.374	1.63 (0.62 to 2.85)	0.372
**Vitamin D**										
Non-user										
Past user	1.8 (0.81 to 3.04)	0.119	1.6 (0.64 to 2.96)	0.396	1.6 (0.61 to 3.04)	0.443	1.62 (0.64 to 3.01)	0.411	1.48 (0.58 to 2.83)	0.448
Current user	2.04 (1.25 to 2.94)	0.009	1.7 (0.92 to 2.71)	0.200	1.75 (0.9 to 2.86)	0.307	1.83 (1 to 2.88)	0.11	1.58 (0.84 to 2.57)	0.315
**Proton pump inhibitor**										
Non-user										
Past user	0.87 (0.31 to 1.73)	0.880	0.79 (0.25 to 1.74)	0.618	0.63 (0.18 to 1.55)	0.452	0.69 (0.21 to 1.61)	0.5	0.8 (0.25 to 1.78)	0.705

For peer review only

Current user	0.96 (0.57 to 1.5)	0.880	0.7 (0.36 to 1.23)	0.356	0.57 (0.26 to 1.08)	0.209	0.61 (0.29 to 1.12)	0.221	0.69 (0.35 to 1.23)	0.337
**Statin**										
Non-user										
Past user	1.78 (0.8 to 3.1)	0.130	1.2 (0.47 to 2.44)	0.697	1.08 (0.39 to 2.38)	0.876	1.04 (0.39 to 2.22)	0.939	1.1 (0.42 to 2.3)	0.869
Current user	1.97 (1.21 to 2.95)	0.014	1.42 (0.76 to 2.39)	0.360	1.25 (0.62 to 2.26)	0.661	1.28 (0.66 to 2.23)	0.529	1.34 (0.7 to 2.3)	0.457
**Immunosuppressant**										
Non-user										
Past user	1 (0.06 to 2.91)	1.000	0.7 (0.04 to 2.62)	0.750	0.78 (0.04 to 2.86)	0.937	0.82 (0.04 to 2.88)	0.836	0.69 (0.04 to 2.6)	0.761
Current user	1.5 (0.43 to 2.87)	0.650	1.62 (0.43 to 3.07)	0.460	1.52 (0.35 to 3.11)	0.636	1.56 (0.4 to 3.05)	0.51	1.57 (0.4 to 3.07)	0.512
Complications post diagnosis										
**Cardiovascular**										
No										
Recurrent	1.72 (1.03 to 2.65)	0.066	1·26 (0·65 to 2·19)	0·568	0.95 (0.43 to 1.89)	0.912	1.2 (0.61 to 2.12)	0.69	1.21 (0.62 to 2.14)	0.655
Novel	0.86 (0.21 to 2.17)	0.789	0·96 (0·22 to 2·54)	0·954	1.12 (0.23 to 3.04)	0.912	0.9 (0.19 to 2.47)	0.87	1.23 (0.27 to 3.11)	0.808
**Respiratory**										
No										
Recurrent	0.98 (0.54 to 1.65)	0.948	0·69 (0·33 to 1·3)	0·387	0.76 (0.35 to 1.45)	0.561	0.75 (0.36 to 1.41)	0.476	0.72 (0.34 to 1.35)	0.418
Novel	1.23 (0.61 to 2.09)	0.792	0·98 (0·43 to 1·85)	0·961	1.06 (0.46 to 1.99)	0.913	0.99 (0.43 to 1.86)	0.97	1.01 (0.44 to 1.91)	0.971
**Renal**										
No										
Recurrent	3.75 (2.08 to 6.17)	<0.001	2·74 (1·32 to 5·13)	0·056	2.54 (1.1 to 5.15)	0.218	2.63 (1.25 to 5)	0.11	2.45 (1.15 to 4.76)	0.096
Novel	2.5 (0.88 to 5.38)	0.076	2·04 (0·6 to 4·98)	0·329	1.9 (0.52 to 4.94)	0.437	2.18 (0.63 to 5.25)	0.31	1.81 (0.51 to 4.66)	0.385

	Adjusted RR (+cholesterol) (95% CI)	P value	Adjusted RR (+respiratory) (95% CI)	P value	Adjusted RR (+cardiovascular) (95% CI)	P value	Adjusted RR (+kidney) (95% CI)	P value
Demographics								
**Gender**								
Female								
Male	1.66 (1 to 2.5)	0.167	1.64 (0.98 to 2.49)	0.185	1.65 (0.99 to 2.5)	0.173	1.58 (0.93 to 2.43)	0.209
**Ethnic origin**								
White								
South Asian	1.7 (0.93 to 2.68)	0.204	1.71 (0.94 to 2.71)	0.191	1.69 (0.93 to 2.68)	0.204	1.67 (0.91 to 2.66)	0.209
Black	2.39 (1.34 to 3.48)	0.045	2.4 (1.35 to 3.49)	0.027	2.41 (1.36 to 3.5)	0.039	2.24 (1.21 to 3.36)	0.105

For peer review only

Other	0.41 (0.02 to 1.75)	0.475	0.37 (0.02 to 1.66)	0.408	0.41 (0.02 to 1.76)	0.477	0.39 (0.02 to 1.71)	0.409
**HPB diagnosis**								
Cancer	2.67 (0.53 to 3.9)	0.303	2.91 (0.67 to 3.92)	0.233	2.68 (0.54 to 3.9)	0.319	2.97 (0.69 to 3.93)	0.219
Non cancer								
Pancreatic disease	1.5 (0.81 to 2.36)	0.303	1.54 (0.83 to 2.41)	0.233	1.49 (0.79 to 2.34)	0.319	1.49 (0.79 to 2.36)	0.324
Liver disease	1.16 (0.62 to 1.84)	0.664	1.17 (0.63 to 1.85)	0.593	1.14 (0.6 to 1.83)	0.658	1.15 (0.61 to 1.84)	0.667
Biliary disease	1.36 (0.71 to 2.26)	0.475	1.36 (0.71 to 2.26)	0.408	1.37 (0.72 to 2.27)	0.473	1.41 (0.74 to 2.33)	0.409
**Age group**								
18-40								
41-50	1.83 (0.29 to 7.09)	0.599	1.99 (0.32 to 7.37)	0.474	1.86 (0.3 to 7.08)	0.596	1.57 (0.24 to 6.58)	0.667
51-60	1.74 (0.32 to 6.78)	0.599	1.92 (0.36 to 7.08)	0.474	1.73 (0.32 to 6.69)	0.596	1.45 (0.26 to 6.13)	0.667
61-70	2.87 (0.7 to 8.09)	0.303	3.14 (0.78 to 8.35)	0.233	2.78 (0.67 to 7.98)	0.319	2.18 (0.49 to 7.25)	0.409
71-80	5.2 (1.77 to 9.58)	0.048	5.63 (2.03 to 9.77)	0.027	5.02 (1.67 to 9.49)	0.067	3.87 (1.11 to 8.85)	0.203
80+	5.1 (1.74 to 9.52)	0.048	5.63 (2.05 to 9.76)	0.027	4.87 (1.59 to 9.43)	0.067	3.69 (1.03 to 8.73)	0.203
Comorbidities								
**Diabetes**	1.99 (1.02 to 3.49)	0.126	1.9 (0.95 to 3.4)	0.181	1.97 (1.01 to 3.47)	0.132	1.85 (0.93 to 3.32)	0.150
**Hypertension**	5.53 (1.17 to 23.07)	0.192	5.42 (1.15 to 22.95)	0.199	5.44 (1.14 to 23)	0.202	4.85 (0.96 to 22.48)	0.231
**Cholesterol**			1.15 (0.6 to 2.04)	0.728	1.09 (0.55 to 1.96)	0.827	1.03 (0.52 to 1.89)	0.922
**Respiratory**	0.7 (0.38 to 1.17)	0.313			0.67 (0.36 to 1.13)	0.260	0.66 (0.36 to 1.13)	0.236
**Cardiovascular**	1.25 (0.66 to 2.15)	0.564	1.37 (0.73 to 2.31)	0.373			0.91 (0.43 to 1.75)	0.803
**Kidney**	2.12 (1.15 to 3.55)	0.063	2.2 (1.2 to 3.67)	0.061	2.2 (1.13 to 3.8)	0.079		
**Number of comorbidities**								
Lifestyle factors								
**Smoker**								
Never								
Past	1.42 (0.76 to 2.34)	0.46	1.46 (0.79 to 2.39)	0.329	1.39 (0.75 to 2.31)	0.46	1.34 (0.7 to 2.27)	0.402
Current	0.48 (0.07 to 1.63)	0.46	0.54 (0.08 to 1.76)	0.46	0.48 (0.07 to 1.64)	0.469	0.42 (0.06 to 1.52)	0.371
**Drinker**								
Never								
Past	1.21 (0.5 to 2.35)	0.695	1.14 (0.46 to 2.27)	0.757	1.24 (0.51 to 2.4)	0.667	1.21 (0.49 to 2.38)	0.656
Current	1.53 (0.74 to 2.65)	0.454	1.5 (0.71 to 2.62)	0.45	1.63 (0.78 to 2.78)	0.301	1.81 (0.88 to 2.98)	0.177
**Substance user**								
Never								
Past	0.53 (0.06 to 2.23)	0.608	0.58 (0.07 to 2.35)	0.658	0.4 (0.04 to 1.95)	0.517	0.39 (0.04 to 1.91)	0.457
Current	1.61 (0.58 to 3.19)	0.601	1.75 (0.65 to 3.32)	0.468	1.35 (0.43 to 2.98)	0.68	1.27 (0.4 to 2.84)	0.708
**Obese**								

For peer review only

Never								
Past	1.12 (0.42 to 2.45)	0.883	1.05 (0.39 to 2.36)	0.933	1.1 (0.41 to 2.42)	0.912	0.96 (0.33 to 2.27)	0.929
Current	1 (0.35 to 2.33)	0.994	0.96 (0.33 to 2.27)	0.933	1 (0.35 to 2.33)	0.993	0.89 (0.29 to 2.21)	0.894
Prescription medication use								
**ACE inhibitor**								
Non-user								
Past user	1.44 (0.64 to 2.57)	0.421	1.45 (0.65 to 2.57)	0.382	1.4 (0.62 to 2.5)	0.451	1.34 (0.58 to 2.45)	0.491
Current user	2.12 (1.11 to 3.24)	0.082	2.16 (1.15 to 3.26)	0.063	2.07 (1.09 to 3.18)	0.089	2.03 (1.07 to 3.14)	0.084
**Angiotensin receptor blocker**								
Non-user								
Past user	1.91 (0.43 to 3.62)	0.471	2.05 (0.47 to 3.7)	0.361	1.77 (0.38 to 3.57)	0.584	1.95 (0.47 to 3.63)	0.387
Current user	1.21 (0.49 to 2.24)	0.713	1.21 (0.49 to 2.24)	0.69	1.25 (0.51 to 2.28)	0.627	1.28 (0.52 to 2.33)	0.584
**Aldosterone agonist**								
Non-user								
Past user	1.04 (0.18 to 2.6)	0.947	1.17 (0.2 to 2.77)	0.809	0.98 (0.16 to 2.54)	0.98	0.81 (0.13 to 2.29)	0.82
Current user	1.45 (0.35 to 2.93)	0.677	1.56 (0.38 to 3.03)	0.515	1.38 (0.32 to 2.89)	0.687	1.13 (0.24 to 2.66)	0.844
**β-blocker**								
Non-user								
Past user	1.07 (0.3 to 2.33)	0.903	1.04 (0.29 to 2.29)	0.94	0.95 (0.25 to 2.21)	0.921	0.88 (0.24 to 2.08)	0.81
Current user	0.79 (0.38 to 1.43)	0.614	0.81 (0.39 to 1.45)	0.612	0.72 (0.33 to 1.36)	0.444	0.69 (0.32 to 1.3)	0.368
**Calcium channel blocker**								
Non-user								
Past user	0.42 (0.09 to 1.26)	0.353	0.46 (0.1 to 1.33)	0.369	0.41 (0.09 to 1.25)	0.342	0.38 (0.08 to 1.18)	0.241
Current user	1.37 (0.7 to 2.26)	0.455	1.34 (0.67 to 2.22)	0.437	1.35 (0.68 to 2.23)	0.47	1.32 (0.67 to 2.19)	0.427
**Antiplatelet**								
Non-user								
Past user	1.03 (0.34 to 2.21)	0.95	1.13 (0.38 to 2.36)	0.797	0.98 (0.31 to 2.17)	0.964	1.11 (0.38 to 2.3)	0.819
Current user	1.3 (0.65 to 2.22)	0.577	1.32 (0.67 to 2.23)	0.505	1.23 (0.59 to 2.19)	0.693	1.09 (0.52 to 1.95)	0.819
**Antiarrhythmic**								
Non-user								
Past user	2.31 (0.15 to 3.85)	0.558	2.6 (0.2 to 3.87)	0.407	2.23 (0.14 to 3.84)	0.54	2.38 (0.14 to 3.86)	0.478
Current user	1.05 (0.07 to 3)	0.952	1.26 (0.08 to 3.18)	0.794	1 (0.06 to 2.95)	0.997	1.03 (0.06 to 2.98)	0.97
**Anticoagulant**								
Non-user								
Past user	0.36 (0.01 to 1.98)	0.539	0.32 (0.01 to 1.89)	0.441	0.32 (0.01 to 1.88)	0.458	0.34 (0.01 to 1.88)	0.401
Current user	1.09 (0.32 to 2.37)	0.87	1.13 (0.33 to 2.43)	0.811	1.05 (0.3 to 2.33)	0.929	1 (0.28 to 2.3)	0.999

**Glucocorticoid**								
Non-user								
Past user	1.19 (0.47 to 2.3)	0.793	1.3 (0.51 to 2.47)	0.574	1.22 (0.48 to 2.34)	0.686	1.21 (0.47 to 2.35)	0.713
Current user	1.22 (0.65 to 2.02)	0.652	1.39 (0.74 to 2.25)	0.385	1.25 (0.67 to 2.04)	0.541	1.22 (0.65 to 2.01)	0.61
**β2-agonist**								
Non-user								
Past user	1.52 (0.38 to 3.14)	0.623	1.98 (0.53 to 3.52)	0.324	1.49 (0.36 to 3.12)	0.597	1.53 (0.36 to 3.21)	0.576
Current user	1.18 (0.56 to 2.05)	0.741	1.58 (0.74 to 2.64)	0.324	1.17 (0.56 to 2.04)	0.698	1.13 (0.53 to 1.99)	0.731
**Muscarinic antagonist**								
Non-user								
Past user	1.12 (0.26 to 2.57)	0.846	1.14 (0.26 to 2.58)	0.9	1.14 (0.27 to 2.58)	0.827	1.02 (0.23 to 2.45)	0.977
Current user	0.85 (0.36 to 1.63)	0.83	0.95 (0.4 to 1.8)	0.901	0.83 (0.34 to 1.61)	0.744	0.76 (0.3 to 1.51)	0.569
**NSAID**								
Non-user								
Past user	0.51 (0.08 to 1.57)	0.421	0.53 (0.08 to 1.6)	0.402	0.54 (0.08 to 1.63)	0.431	0.61 (0.09 to 1.84)	0.516
Current user	1.57 (0.6 to 2.75)	0.421	1.49 (0.55 to 2.69)	0.402	1.53 (0.58 to 2.73)	0.431	1.63 (0.62 to 2.84)	0.322
**Vitamin D**								
Non-user								
Past user	1.59 (0.63 to 2.96)	0.45	1.79 (0.73 to 3.2)	0.322	1.56 (0.62 to 2.93)	0.43	1.53 (0.6 to 2.89)	0.36
Current user	1.69 (0.91 to 2.71)	0.23	1.97 (1.07 to 3.06)	0.081	1.67 (0.9 to 2.68)	0.253	1.56 (0.81 to 2.57)	0.34
**Proton pump inhibitor**								
Non-user								
Past user	0.77 (0.24 to 1.71)	0.62	0.72 (0.21 to 1.67)	0.56	0.77 (0.24 to 1.7)	0.626	0.76 (0.24 to 1.7)	0.577
Current user	0.67 (0.33 to 1.2)	0.315	0.71 (0.36 to 1.26)	0.384	0.68 (0.35 to 1.21)	0.346	0.64 (0.31 to 1.15)	0.219
**Statin**								
Non-user								
Past user	1.2 (0.46 to 2.48)	0.76	1.23 (0.48 to 2.51)	0.681	1.15 (0.44 to 2.39)	0.758	1.23 (0.48 to 2.51)	0.69
Current user	1.42 (0.73 to 2.46)	0.42	1.4 (0.75 to 2.37)	0.361	1.38 (0.73 to 2.35)	0.443	1.38 (0.73 to 2.36)	0.372
**Immunosuppressant**								
Non-user								
Past user	0.68 (0.04 to 2.6)	0.779	0.73 (0.04 to 2.71)	0.747	0.71 (0.04 to 2.62)	0.776	0.65 (0.04 to 2.55)	0.72
Current user	1.59 (0.42 to 3.06)	0.527	1.73 (0.47 to 3.18)	0.424	1.69 (0.46 to 3.11)	0.454	1.5 (0.37 to 3)	0.58
Complications post diagnosis								
**Cardiovascular**								
No								
Recurrent	1.24 (0.63 to 2.19)	0.67	1.36 (0.71 to 2.36)	0.444	1.26 (0.65 to 2.19)	0.568	0.9 (0.41 to 1.78)	0.854
Novel	0.96 (0.21 to 2.53)	0.95	1 (0.23 to 2.6)	0.997	0.96 (0.22 to 2.54)	0.954	0.94 (0.2 to 2.54)	0.92

For peer review only

**Respiratory**								
No								
Recurrent	0.68 (0.33 to 1.29)	0.408	0.69 (0.33 to 1.3)	0.387	0.64 (0.3 to 1.24)	0.374	0.66 (0.31 to 1.26)	0.328
Novel	0.97 (0.42 to 1.84)	0.93	0.98 (0.43 to 1.85)	0.961	0.96 (0.42 to 1.82)	0.91	1.01 (0.44 to 1.92)	0.978
**Renal**								
No								
Recurrent	2.73 (1.31 to 5.14)	0.062	2.79 (1.34 to 5.22)	0.058	2.91 (1.31 to 5.59)	0.057	2.74 (1.32 to 5.13)	0.056
Novel	2.04 (0.6 to 4.98)	0.366	1.91 (0.55 to 4.81)	0.326	2.09 (0.61 to 5.09)	0.343	2.04 (0.6 to 4.98)	0.329

Risk ratios, except the crude ones, are mutually adjusted for gender, ethnicity, age group and HPB diagnosis, and also for additional conditions when mentioned inside the parenthesis. Simplified binary age group (over and under 60) are used for adjustment for all categories except demographics. All P values presented, except for the crude risk ratios, are Benjamini-Hochberg corrected.

For peer review only

STROBE Statement—Checklist of items that should be included in reports of ***cohort studies***

	Item No	Recommendation	Page No
**Title and abstract**	1	(*a*) Indicate the study's design with a commonly used term in the title or the abstract	1,2
		(*b*) Provide in the abstract an informative and balanced summary of what was done and what was found	2
**Introduction**			
Background/rationale	2	Explain the scientific background and rationale for the investigation being reported	4,5
Objectives	3	State specific objectives, including any prespecified hypotheses	5
**Methods**			
Study design	4	Present key elements of study design early in the paper	6
Setting	5	Describe the setting, locations, and relevant dates, including periods of recruitment, exposure, follow-up, and data collection	6,7
Participants	6	(*a*) Give the eligibility criteria, and the sources and methods of selection of participants. Describe methods of follow-up	6,7
		(*b*) For matched studies, give matching criteria and number of exposed and unexposed	
Variables	7	Clearly define all outcomes, exposures, predictors, potential confounders, and effect modifiers. Give diagnostic criteria, if applicable	7-9, Table 1
Data sources/ measurement	8*	For each variable of interest, give sources of data and details of methods of assessment (measurement). Describe comparability of assessment methods if there is more than one group	6-9, Supplemental Tables 1,2
Bias	9	Describe any efforts to address potential sources of bias	7
Study size	10	Explain how the study size was arrived at	6,7
Quantitative variables	11	Explain how quantitative variables were handled in the analyses. If applicable, describe which groupings were chosen and why	9,10
Statistical methods	12	(*a*) Describe all statistical methods, including those used to control for confounding	9, 10
		(*b*) Describe any methods used to examine subgroups and interactions	
		(*c*) Explain how missing data were addressed	
		(*d*) If applicable, explain how loss to follow-up was addressed	
		(*e*) Describe any sensitivity analyses	
**Results**			
Participants	13*	(a) Report numbers of individuals at each stage of study—eg numbers potentially eligible, examined for eligibility, confirmed eligible, included in the study, completing follow-up, and analysed	11, Figure 1
		(b) Give reasons for non-participation at each stage	
		(c) Consider use of a flow diagram	
Descriptive data	14*	(a) Give characteristics of study participants (eg demographic, clinical, social) and information on exposures and potential confounders	11-18, Tables 2,3
		(b) Indicate number of participants with missing data for each variable of interest	
		(c) Summarise follow-up time (eg, average and total amount)	
Outcome data	15*	Report numbers of outcome events or summary measures over time	11

For peer review only - http://bmjopen.bmj.com/site/about/guidelines.xhtml

Main results	16	(*a*) Give unadjusted estimates and, if applicable, confounder-adjusted estimates and their precision (eg, 95% confidence interval). Make clear which confounders were adjusted for and why they were included<br>(*b*) Report category boundaries when continuous variables were categorized<br>(*c*) If relevant, consider translating estimates of relative risk into absolute risk for a meaningful time period	18, 19, Figures 2,3, Supplementary tables 3,4
Other analyses	17	Report other analyses done—eg analyses of subgroups and interactions, and sensitivity analyses	18, 19, Supplementary tables 3,4
**Discussion**			
Key results	18	Summarise key results with reference to study objectives	20, 23
Limitations	19	Discuss limitations of the study, taking into account sources of potential bias or imprecision. Discuss both direction and magnitude of any potential bias	20, 21
Interpretation	20	Give a cautious overall interpretation of results considering objectives, limitations, multiplicity of analyses, results from similar studies, and other relevant evidence	21-23
Generalisability	21	Discuss the generalisability (external validity) of the study results	20
**Other information**			
Funding	22	Give the source of funding and the role of the funders for the present study and, if applicable, for the original study on which the present article is based	24

*Give information separately for exposed and unexposed groups.

Note: An Explanation and Elaboration article discusses each checklist item and gives methodological background and published examples of transparent reporting. The STROBE checklist is best used in conjunction with this article (freely available on the Web sites of PLoS Medicine at http://www.plosmedicine.org/, Annals of Internal Medicine at http://www.annals.org/, and Epidemiology at http://www.epidem.com/). Information on the STROBE Initiative is available at http://www.strobe-statement.org.

For peer review only

BMJ Open

COVID-19 in patients with hepatobiliary and pancreatic diseases: A single-centre cross-sectional study in East London

Journal:	*BMJ Open*
Manuscript ID	bmjopen-2020-045077.R1
Article Type:	Original research
Date Submitted by the Author:	06-Mar-2021
Complete List of Authors:	Dayem Ullah, Abu; Barts Cancer Institute, Centre for Cancer Biomarker and Biotherapeutics; Barts Health NHS Trust, Barts and the London HPB Centre, The Royal London Hospital<br>Sivapalan, Lavanya; Barts Cancer Institute, Centre for Cancer Biomarker and Biotherapeutics<br>Kocher, Hemant ; Barts Cancer Institute, Centre for Tumour Biology; Barts Health NHS Trust, Barts and the London HPB Centre, The Royal London Hospital<br>Chelala, Claude; Barts Cancer Institute, Centre for Cancer Biomarker and Biotherapeutics
**Primary Subject Heading**:	Gastroenterology and hepatology
Secondary Subject Heading:	Epidemiology, Health informatics, Public health
Keywords:	Hepatobiliary disease < GASTROENTEROLOGY, Pancreatic disease < GASTROENTEROLOGY, COVID-19

SCHOLARONE™
Manuscripts

I, the Submitting Author has the right to grant and does grant on behalf of all authors of the Work (as defined in the below author licence), an exclusive licence and/or a non-exclusive licence for contributions from authors who are: i) UK Crown employees; ii) where BMJ has agreed a CC-BY licence shall apply, and/or iii) in accordance with the terms applicable for US Federal Government officers or employees acting as part of their official duties; on a worldwide, perpetual, irrevocable, royalty-free basis to BMJ Publishing Group Ltd ("BMJ") its licensees and where the relevant Journal is co-owned by BMJ to the co-owners of the Journal, to publish the Work in this journal and any other BMJ products and to exploit all rights, as set out in our licence.

The Submitting Author accepts and understands that any supply made under these terms is made by BMJ to the Submitting Author unless you are acting as an employee on behalf of your employer or a postgraduate student of an affiliated institution which is paying any applicable article publishing charge ("APC") for Open Access articles. Where the Submitting Author wishes to make the Work available on an Open Access basis (and intends to pay the relevant APC), the terms of reuse of such Open Access shall be governed by a Creative Commons licence – details of these licences and which Creative Commons licence will apply to this Work are set out in our licence referred to above.

Other than as permitted in any relevant BMJ Author's Self Archiving Policies, I confirm this Work has not been accepted for publication elsewhere, is not being considered for publication elsewhere and does not duplicate material already published. I confirm all authors consent to publication of this Work and authorise the granting of this licence.

For peer review only

COVID-19 in patients with hepatobiliary and pancreatic diseases: A single-centre cross-sectional study in East London

Abu Z M Dayem Ullah, UKRI/Rutherford research fellow[1 2], Lavanya Sivapalan, PhD student[1], Hemant M Kocher, professor of liver and pancreas surgery[1 3], Claude Chelala, professor of bioinformatics[1]

Author affiliations

[1] Centre for Cancer Biomarkers and Therapeutics, Barts Cancer Institute, Queen Mary University of London, London, UK
[2] Barts and the London HPB Centre, The Royal London Hospital, Barts Health NHS Trust, London, UK
[3] Centre for Tumour Biology, Barts Cancer Institute, Queen Mary University of London, London, UK

Correspondence to:

Abu Z M Dayem Ullah
Centre for Cancer Biomarkers and Therapeutics, Barts Cancer Institute, Queen Mary University of London, London EC1M 6BQ, UK
Email: d.ullah@qmul.ac.uk

For peer review only - http://bmjopen.bmj.com/site/about/guidelines.xhtml

ABSTRACT

Objective To explore risk factors associated with COVID-19 susceptibility and survival in patients with pre-existing hepato-pancreato-biliary (HPB) conditions.

Design Cross-sectional study.

Setting East London Pancreatic Cancer Epidemiology (EL-PaC-Epidem) study at Barts Health NHS Trust, UK. Linked electronic health records were interrogated on a cohort of participants (age ≥ 18 years), reported with HPB conditions between 1 April 2008 and 6 March 2020.

Participants EL-PaC-Epidem study participants, alive on 12 February 2020, and living in East London within the previous six months (n=15 440). The cohort represents a multi-ethnic population with 51.7% belonging to the non-White background.

Main outcome measure COVID-19 incidence and mortality.

Results Some 226 (1.5%) participants had confirmed COVID-19 diagnosis between 12 February and 12 June 2020, with an increased odds for men (OR 1.56; 95% CI 1.2 to 2.04) and Black ethnicity (2.04; 1.39 to 2.95) as well as patients with moderate to severe liver disease (2.2; 1.35 to 3.59). Each additional comorbidity increased the odds of infection by 62%. Substance mis-users were at more risk of infection, so were patients on Vitamin D treatment. The higher odds ratios in patients with chronic pancreatic or mild liver conditions, age>70, and history of smoking or obesity were due to co-existing comorbidities. Increased odds of death were observed for men (3.54; 1.68 to 7.85) and Black ethnicity (3.77; 1.38 to 10.7). Patients having respiratory complications from COVID-19 without a history of chronic respiratory disease also had higher odds of death (5.77; 1.75 to 19).

Conclusions In this large population-based study of HPB patients, men, Black ethnicity, pre-existing moderate to severe liver conditions, six common medical multi-morbidities, substance mis-use, and a history of Vitamin D treatment independently posed higher odds of acquiring COVID-19 compared to their respective counterparts. The odds of death were significantly high for men and Black people.

For peer review only - http://bmjopen.bmj.com/site/about/guidelines.xhtml

STRENGTHS AND LIMITATIONS OF THIS STUDY

- First multi-ethnic population-based study on COVID-19 in patients with hepato-pancreato-biliary group of diseases.
- Systematic identification of the effect, or the lack of it, of individual demographic and clinical factors on the infection and mortality of COVID-19 in a large cohort of over 15 000 patients, robustly controlling for potential confounders in their evaluation.
- Access to longitudinal data from linked primary and secondary care electronic health records, and use of rule-based phenotyping algorithms allowed for improved completeness and accuracy of the explored variables.
- Some observed increased odds of SARS-CoV-2 infection and related death could be plausibly explained by unmeasured confounding.
- The effects reported in the study could be influenced by the relatively smaller size of COVID-19 cases within this cohort.

For peer review only - http://bmjopen.bmj.com/site/about/guidelines.xhtml

INTRODUCTION

COVID-19 is a novel infectious disease caused by severe acute respiratory syndrome coronavirus 2 (SARS-CoV-2), with a wide-ranging disease course. Infection and mortality rates of the COVID-19 pandemic have varied widely among nations and demographics,[1] while risks are still being explored, identified and categorised according to the severity.[2 3] There are several confirmed risk factors of COVID-19 and severe outcomes, including old age,[2 4 5] chronic pulmonary disease,[2 4-6] cardiovascular disease,[2 5 6] hypertension,[5] chronic kidney disease,[2 4 6] diabetes mellitus,[2 5] obesity,[2 6 7] haematological diseases,[2 4] malignancy,[2 4-6 8] and immuno-compromised state such as HIV infection.[2 4 9] Medical complications following hospitalisation, including acute episodes of cardiovascular, respiratory, neurological, renal, or hepatic failure, have also been linked to severe outcomes.[10] There are also other risk factors reported, such as smoking[11 12] or being from a Black, Asian and minority ethnic (BAME) group,[13-15] the effects of which are either mixed or the reasoning is not clearly understood.[4] Concerns have also been raised regarding the use of various medications with respect to the risk or protective effect to COVID-19.[16-18]

Patients with diseases of the liver, pancreas or biliary tract (hepato-pancreato-biliary; HPB) are considered, in general, to be at risk of developing serious medical conditions. Expression of the ACE2 gene – a receptor for the SARS-CoV-2 virus - along the gastrointestinal tract is well documented, which suggests the digestive system is a potential route for COVID-19,[18] making patients with a diseased HPB system susceptible to this novel infection. The prevalence of COVID-19 among patients with hepatic conditions has been explored,[6 15 19] indicating severe liver disease as a moderate risk factor for COVID-19.[2] In contrast, very limited data is available on the prevalence of COVID-19 among patients with pancreatic or biliary conditions,[20] although pancreatic manifestations of the disease are rare.[21 22] It is important that clinical characteristics of COVID-19 are investigated for the HPB group as a whole, not only because these diseases demonstrate similar clinical-biologic behaviours,[23] but also since they are commonly seen by a single clinical unit with specialist expertise in the management of these diseases.

The United Kingdom (UK) has been the worst affected country in Europe by COVID-19, with a reported death toll of 44819 as of 30 June 2020.[24] At the same time, London had the highest incidence and mortality rates, with 33775 confirmed cases and 8438 deaths.[25 26] Barts Health NHS Trust (BHNT) is the largest National Health Service (NHS) Trust in England and acts as provider of district general hospital facilities for around 2.5 million population of East London as well as a range of tertiary care services.[27] Between March 1 and June 30, the three boroughs in East London - Tower Hamlets, Waltham Forest and Newham - had a combined age-standardised COVID-19 related mortality rate of 195 per 100 000 people. This was significantly higher than the rest of London where the age-standardised COVID-19 related mortality rate was 156 per 100 000 people.[25] East London is also one of the most ethnically diverse local areas in the country where an estimated 57% residents belong to a BAME group.[28] Significant health inequalities exist within the local population including higher rates of cancer, diabetes and obesity,[29] compared to the wider population. These conditions are not only known to be a precursor or consequence to HPB diseases, but also linked to COVID-19 and severe outcomes. In this study, we integrated primary, secondary and tertiary electronic healthcare records (EHRs) of HPB patients in East London. We inspected the demographics, lifestyle, comorbidities and associated medication use of

For peer review only - http://bmjopen.bmj.com/site/about/guidelines.xhtml

these patients, and any possible links with SARS-CoV-2 infection. We also evaluated whether the effect of these prevalent factors as well as clinical observations during COVID-19 related hospitalisation are associated with mortality. This study will inform the management of this specific cohort of patients.

For peer review only

METHODS

Study setting and data sources

All data utilised for this study were collected and processed under the East London Pancreatic Cancer Epidemiology (EL-PaC-Epidem) study at BHNT. In brief, EL-PaC-Epidem is an ongoing study that ascertains patients diagnosed or reported with HPB diseases including cancers, as well as control patients (e.g., small intestine, hernia), within five BHNT hospital sites (The Royal London Hospital, Newham University Hospital, St Bartholomew's Hospital, Whipps Cross University Hospital, Mile End Hospital) between 2008 and 2021. The EL-PaC-Epidem study was approved by the East of England - Essex Research Ethics Committee (19/EE/0163; 17 May 2019) and supported by the NHS Confidentiality Advisory Group for collecting and processing confidential patient information without consent (19/CAG/0219; 17 January 2020). The study is limited to the secondary use of a specified subset of patients' retrospective EHR generated during the course of normal care of these patients. It links EHRs from different data sources (via UK unique individual NHS numbers), including primary care through General Practitioners (GP) (Discovery East London Programme data service [DDS]) and secondary or tertiary care through hospitals (BHNT Consolidated Data Extract [CDE]). Patients, who have previously informed their GPs or NHS to stop sharing their personal and health records for purposes other than their individual care, were automatically excluded. The current EL-PaC-Epidem study cohort consists of 27321 adult patients (aged 18 years or over), diagnosed or reported with at least one of the HPB conditions (supplemental table 1) between 1 April 2008 and 6 March 2020.

Study design and population

This is a single-centre cross-sectional study utilising the linked EHR data of patients with a history of HPB diseases. Within this specific patient group, the study focused on the incidence of COVID-19, and examined the association of SARS-CoV-2 infection with six common medical comorbidities (i.e., diabetes, hypertension, high cholesterol, cardiovascular disease, chronic respiratory disease, renal disease), lifestyle factors (i.e., smoking, alcohol use, substance misuse, obesity), and use of selected prescription medications.

As the first case of COVID-19 in London was reported on 12 February 2020, we used this as the start date for this study and extracted data on a subgroup of the EL-PaC-Epidem study cohort until 12 June 2020 (figure 1). Eligible individuals were a resident in East London and alive on the study start date (EL-HPB). Residency of East London was inferred if a patient had at least one appointment or prescription issued from a GP in East London boroughs or had a scheduled or unscheduled visit to one of the BHNT hospitals within the last six months (after 12 August 2019). Patients with *confirmed* SARS-CoV-2 infection were identified by: i) the presence of International Classification of Diseases 10th edition (ICD-10) or Systematized Nomenclature of Medicine Clinical Terms (SNOMED CT) codes for confirmed COVID-19 or SARS diagnosis assigned in their hospital encounters or GP records during the observation period between February 12 and June 12, 2020 (supplemental table 2) OR ii) positive record of SARS-CoV-2 RNA through BHNT oral and/or nasal swabs test during the same period. For confirmed COVID-19 cases, the earliest date of diagnosis or positive swab test was considered as the *index date,* whereas 12 February 2020 was considered as *index*

For peer review only - http://bmjopen.bmj.com/site/about/guidelines.xhtml

date for rest of the cohort. Patients, who were assigned an ICD-10 or SNOMED CT diagnosis code for *suspected* COVID-19 but were neither reassigned to confirmed diagnosis nor positive RNA test, were excluded from the analysis.

We also examined the onset-to-death distribution within the patient group with a confirmed COVID-19 diagnosis (EL-HPB-COVID). Mortality data was collected on 12 October 2020. Following the latest Public Health England (PHE) definition[30], the death of a patient within 28 days of the index date is considered as a COVID-19 related death. This is different from a 60-day window that was being used in the UK prior to 12 August 2020 to define COVID-19 related death. To ensure consistency, COVID-19 patients who survived beyond 60 days of index date are considered as survivor in the study; Nine patients who died between 29 and 60 days of diagnosis were excluded from the analysis. The onset-to-death distribution was analysed in the context of same set of comorbidities, lifestyle factors and medication use, as well as cardiovascular, respiratory and renal complications during hospital care.

<<Figure 1 here>>

Figure 1 Selection of patients for the cross-sectional study.

Procedures

All patient data were obtained from retrospective EHR, harmonised across hospital and GP coding systems where applicable, and organised into 40 primary variables across seven categories corresponding to the focus of the study (table 1). BHNT CDE uses 2011 UK census grouping to record ethnicity, ICD-10 or SNOMED diagnosis codes for clinically relevant diagnoses, and Office of the Population Censuses and Surveys Classification of Interventions and Procedures version 4 (OPCS-4) procedural codes for treatments and procedures. Physiological observations (weight, body-mass index [BMI], blood pressure) and laboratory tests results are available in locally developed terms. Semi-structured text entries such as discharge summaries, past medical history and a lifestyle questionnaire collected during the pre-operative assessment, and presenting symptoms from scheduled or unscheduled hospital visits are also available. All GP records via DDS were available in Read Codes v2 or Clinical Terminology Version 3 (CTV3) codes, except the prescribed medication records and COVID-19 diagnosis which were available in SNOMED codes. For each variable, we consulted ICD-10, SNOMED, Read, CTV3 or OPCS-4 dictionaries as appropriate to construct the mapping *codelists.* For some variables, codelists also included keywords to conduct automated sub-string search within semi-structured text as well as local laboratory test and physiological observation terms.

Rule-based phenotyping algorithms were developed for each categorical variable to characterise patients, integrating information from multiple sources where available to counteract bias. HPB diseases were grouped into four categories (supplemental table 1): *any* malignant disease, and non-malignant diseases of liver, pancreas or biliary tract. Non-malignant liver diseases were further divided into mild and moderate to severe subgroups, extending the definition from CDMF Charlson Comorbidity Index,[31] whereas non-malignant pancreas or biliary diseases were divided into acute and chronic disease subgroups (supplemental table 1). Within each disease category, a patient was assigned to chronic (or more severe) subgroup, when data indicated the history of both acute (or mild) and chronic (more severe) conditions. A patient can

either be assigned to a malignant disease category or any of the non-malignant disease subgroups. Ethnicity was grouped into four categories - White, South Asian, Black, and Other. White and Black ethnic groups were defined based on the 2011 UK census classification; Indian, Pakistani and Bangladeshi origin from the Asian group represented South Asian, while the rest (i.e., Mixed, Chinese, other Asian and other ethnic group) were represented in the Other group. The ethnic category recorded at the GP took precedence over hospital records.

Table 1 Variables and outcomes explored in this study.

Category	Variables	Levels/Units
Demographic	Gender	Female, Male
	Ethnicity	White, South Asian, Black, Other [, Not available]
	Age (continuous)	years
	Age group*	18-40, 41-50, 51-60, 61-70, 71-80, 80+
	Binary age group*	18-60, 60+
HPB disease	Cancer	No, Yes
	Pancreatic disease	Acute, Chronic
	Biliary disease	Acute, Chronic
	Liver disease	Mild, Moderate/Severe
Comorbidity	Diabetes	No, Yes
	Hypertension	No, Yes
	High cholesterol	No, Yes
	Cardiovascular disease	No, Yes
	Chronic Respiratory disease	No, Yes
	Renal disease	No, Yes
	Number of comorbidities*	None, 1, 2, 3 or more
Lifestyle factors	Smoker	Never, Past, Current [, Not available]
	Alcohol drinker	Never, Past, Current [, Not available]
	Substance user	Never, Past, Current [, Not available]
	Obese	Never, Past, Current [, Not available]
Medication use	Angiotensin-converting enzyme [ACE] inhibitors	Non-, Past, Current user
	Angiotensin II receptor blockers inhibitors [ARBs]	Non-, Past, Current user
	Aldosterone antagonists [MCRA]	Non-, Past, Current user
	Beta-adrenergic blocking agents [β-blockers]	Non-, Past, Current user
	Calcium channel blockers [CCBs]	Non-, Past, Current user
	Alpha agonist	Non-, Past, Current user
	Thiazide	Non-, Past, Current user
	Antiplatelet	Non-, Past, Current user
	Antiarrhythmic	Non-, Past, Current user
	Anticoagulant	Non-, Past, Current user
	Glucocorticoid	Non-, Past, Current user
	Beta-2 adrenergic receptor agonists [β_2-agonists]	Non-, Past, Current user
	Muscarinic antagonist	Non-, Past, Current user
	Non-steroidal inflammatory drugs [NSAIDs]	Non-, Past, Current user
	Vitamin D	Non-, Past, Current user
	Proton pump inhibitors [PPIs]	Non-, Past, Current user
	Statin	Non-, Past, Current user
	Immunosuppressant	Non-, Past, Current user
Complications	Cardiovascular	No, Recurrent, Novel
	Respiratory	No, Recurrent, Novel
	Renal	No, Recurrent, Novel
	Number of recurrent complications*	None, 1, 2, 3
	Number of novel complications*	None, 1, 2, 3
Outcome	COVID-19 incidence	non-COVID-19, COVID-19
	COVID-19 mortality	Survivor, Deceased

For peer review only - http://bmjopen.bmj.com/site/about/guidelines.xhtml

All variables are categorical, unless otherwise stated. For categorical variables, the first value represents the reference level. Each HPB diagnosis groups are independent binary categorical variables. * Derived variables.

Phenotyping algorithms defining the comorbidities were based on diagnosis codes (presence) or semi-structured text search (presence or absence), with the additional inclusion of procedural codes (presence), some observation or laboratory test results (presence) and related medication use (at least three prescriptions). Patients were considered to have or have had a specific medical condition if they met at least one criterion indicating the presence of the condition before the *index date*, otherwise they were considered negative for the condition.

Phenotyping algorithms defining the lifestyle factors were based on the longitudinal entries (current, past or never) derived from diagnosis codes and free text search, with the additional inclusion of BMI observation for obesity. Obesity was defined as BMI of 30 kg/m^2 or more. Patients assigned *never* status at any point but having a record of *current* or *past* status before that date were reassigned to *past* status. The most recent lifestyle record before or on the *index date* was then used to assign *current*, *past* or *never* status to the patients. Patients with no record of a specific lifestyle factor were classified as having missing data. Patients were assigned *current*, *past* or *non-user* status for medication use variables based on the number of GP prescriptions issued in the last two years for the medicines under specific medication groups. Patients with no record of prescription for particular medications were assigned *non-user* status. With at least three prescriptions issued, a patient was assigned *current user* status if the latest issue was within three months preceding the *index date,* and *past user* status otherwise. Patients with record of less than three prescriptions were classified as *non-user.* Patients with COVID-19 were considered to have a specific complication during admitted patient care if at least one of the hospital diagnosis codes from the complications *codelist* was recorded during the observation period after *index date*, otherwise they were considered negative for the complication. A patient was considered to have a *recurrent* complication if they had a history of that particular comorbidity, otherwise it was considered as a *novel* complication.

Selection of study variables, *codelist* construction, and phenotyping algorithm development were done in consultation with a panel of clinicians and scientists (HMK, CC, LS). A comprehensive list of codelists and phenotyping algorithms for the study variables are available on the EL-PaC-Epidem portal (https://pac-epidem-el.bcc.qmul.ac.uk/covid-19/).

Statistical analysis

We conducted descriptive analyses for the EL-HPB cohort as a whole, by group for patients with confirmed SARS-CoV-2 infection and the rest (herein referred to as COVID-19 and non-COVID-19 respectively). Differences in demographic and clinical characteristics between the groups were assessed with Pearson's Chi-square test, Fisher's Exact test and Kruskal-Wallis rank sum test, as appropriate. P values less than 0.05 were considered significant. Similar descriptive analyses were performed for the EL-HPB-COVID cohort, and by survivor and deceased groups.

To explore the risk factors associated with COVID-19 susceptibility and subsequent survival, the effect size for each variable under investigation was evaluated with odds

For peer review only - http://bmjopen.bmj.com/site/about/guidelines.xhtml

ratios (ORs) with 95% confidence intervals (CI), using regression models with a binomial distribution. Crude ORs were obtained from univariable regression models, and then simultaneously controlled for a fixed set of potential confounders (gender, ethnicity, age group) using multivariable regression models with Benjamini-Hochberg correction for P values adjustment. The median age of the overall EL-HPB cohort being 57, a simplified binary age grouping (18-60, 61+) was used in multivariable regression models for comorbidity, lifestyle, medication use and post-diagnosis complication analyses. Since a participant with non-malignant HPB diagnoses for multiple organs can be represented in multiple HPB subgroups, the effect estimation for individual HPB disease variables was further mutually controlled for other HPB diseases.

We also conducted more in depth post hoc analysis to evaluate the confounding effect of pre-existing medical conditions by adding comorbidity covariates individually in the multivariable regression models. Finally, effect modification by non-malignant HPB disease subgroups was evaluated by adding interaction terms in the models and comparing them with models lacking this interaction via the likelihood ratio test. Any potential association between HPB diseases and COVID-19 susceptibility/mortality risk factors were further evaluated in stratified analyses according to the disease subgroups.

Patients with missing data for individual categorical variables were included in the descriptive analyses and in regression models for effect estimation. All statistical analyses and visualisations were performed in R (version 3.5.1).

Patient and public involvement

Patients and the public were involved in evaluating the design of the umbrella study (EL-PaC-Epidem), particularly the notion of collection and processing of retrospective patient data without their consent. The support from NHS Confidentiality Advisory Group was obtained based on the positive opinion posed by patient and the public.

For peer review only

RESULTS

Population characteristics

The final EL-HPB cohort consisted of 15 540 patients, after applying the eligibility criteria and excluding 168 suspected but unconfirmed COVID-19 cases. By 12 June 2020, 226 (1.5%; 145 per 10 000 adult population) confirmed cases of COVID-19 were reported in this cohort (figure 1). This was more than three-times higher than in the general population of East London where prevalence of COVID-19 at the same time was 41 per 10 000 adult population.[25] More than half of the COVID-19 cases had some form of non-malignant liver diseases (N=138, 62.8%); however, when comparing confirmed COVID-19 cases with the non-COVID-19 cases, we observed a disproportionate infection frequency in patients with chronic pancreatic conditions (14.1% vs 8.8%) and moderate to severe liver conditions (11.4% vs 6.9%). We also observed differences in gender, ethnic origin, and age group between COVID-19 and non-COVID-19 cases (table 2). The proportion of males was higher in the COVID-19 group compared to the baseline non-COVID-19 group (53.5% *vs* 43.7%, P=0.005). The same trend was observed for Black population (17.7% vs 10.7%). COVID-19 patients were older than non-COVID-19 patients (median 67 years, interquartile range 55.1 to 80.9 years *vs* 57.1 years, 44.8 to 69.2 years, P<0.001), with a steady increase in infection frequency with age. Some 78.3% of COVID-19 patients had three or more comorbidities, with hypertension being the most common comorbidity (85.4%), followed by high cholesterol and diabetes (table 2). Only eight COVID-19 patients had none of the six medical conditions. In general, COVID-19 patients had a higher rate of past history of smoking, drinking, substance mis-use and obesity compared to the non-COVID-19 group. Consistent with the underlying prevalent comorbidities of the COVID-19 group, history of prescription drugs use associated with managing hypertension or cardiovascular disease (ACE inhibitor, calcium channel blocker, β-blocker, aldosterone antagonists, antiarrhythmic, antiplatelet, anticoagulant), cholesterol (statin), inflammation (glucocorticoid, β2-agonists) or background HPB condition (proton pump inhibitor) were higher in COVID-19 patients (table 2). Intake of vitamin D was also significantly higher in COVID-19 patients.

Between 12 February and 12 August 2020, the all-cause mortality rate in the non-COVID-19 group was 2.4%, whereas the rate in the COVID-19 group during the same period was 27.4% (table 2). When analysing the 53 deceased and 164 surviving patients with confirmed SARS-CoV-2 infection, we found differences in gender (P=0.005) and age (P<0.001); deceased patients were older than the survivors (median 80.4 years, interquartile range 71.7 to 85.1 years *vs* 62.9 years, 49.8 to 77.4 years) with steady increase in death with age becoming prominent in those above 70 years of age (table 3). We observed a higher mortality amongst South Asian (34% vs 29.3%) and Black (26.4% vs 13.4%) populations, which were even more pronounced when comparing with the all-cause mortality in the non-COVID-19 group (supplemental table 3). Higher mortality was observed for pancreatic and biliary disease patients in general, whereas liver disease patients had higher survival rate (table 3). The median survival period for the deceased patients from the date of confirmed COVID-19 diagnosis was 11 days (interquartile range 2 to 18 days). After stratifying patients according to the comorbidities investigated, the mortality for HPB patients with COVID-19 was at least six-times higher than that of HPB patients without COVID-19 (supplemental table 3). Diabetes, hypertension, cardiovascular and renal

conditions, in particular, were associated with mortality in COVID-19 patients (table 3). All except one deceased patient had at least three additional comorbidities, compared to 71.3% of patients who survived. The deceased group had higher proportion of patients with a history of past smoking and current substance mis-use, but no overall differences were observed for drinking and obesity. Notable differences were observed in the use of glucocorticoid, β2-agonists, and statins. Recurrent complications were more common in the deceased group compared to survivors, however frequency of novel respiratory complications was notably higher in the deceased group (39.6% vs 21.3%).

Table 2 Differences in demographic, comorbidity, lifestyle, and medication use characteristics between COVID-19 infected and non-COVID-19 groups.

	non-COVID-19 (N=15214)	COVID-19 (N=226)	Total (N=15440)	P value
Demographics				
**Gender**				0.005
Female	8570 (56.3%)	105 (46.5%)	8675 (56.2%)	
Male	6644 (43.7%)	121 (53.5%)	6765 (43.8%)	
**Ethnic origin**				0.004
White	6914 (45.4%)	97 (42.9%)	7011 (45.4%)	
South Asian	4381 (28.8%)	68 (30.1%)	4449 (28.8%)	
Black	1635 (10.7%)	40 (17.7%)	1675 (10.8%)	
Other	1855 (12.2%)	19 (8.4%)	1874 (12.1%)	
Unknown	429 (2.8%)	2 (0.9%)	431 (2.8%)	
**HPB cancer**				1
No	14779 (97.1%)	220 (97.3%)	14999 (97.1%)	
Yes	435 (2.9%)	6 (2.7%)	441 (2.9%)	
**Pancreatic disease***				0.017
No	12264 (83.0%)	170 (77.3%)	12434 (82.9%)	
Acute	1211 (8.2%)	19 (8.6%)	1230 (8.2%)	
Chronic	1304 (8.8%)	31 (14.1%)	1335 (8.9%)	
**Liver disease***				0.004
No	6781 (45.9%)	82 (37.3%)	6863 (45.8%)	
Mild	6985 (47.3%)	113 (51.4%)	7098 (47.3%)	
Moderate/Severe	1013 (6.9%)	25 (11.4%)	1038 (6.9%)	
**Biliary disease***				0.159
No	7589 (51.3%)	127 (57.7%)	7716 (51.4%)	
Acute	738 (5.0%)	11 (5.0%)	749 (5.0%)	
Chronic	6452 (43.7%)	82 (37.3%)	6534 (43.6%)	
**Age**				<0.001
Median	57.08	67.03	57.22	
Q1, Q3	44.76, 69.19	55.07, 80.93	44.86, 69.42	

For peer review only

For peer review only - http://bmjopen.bmj.com/site/about/guidelines.xhtml

**Age group**				<0.001
18-40	2803 (18.4%)	23 (10.2%)	2826 (18.3%)	
41-50	2714 (17.8%)	26 (11.5%)	2740 (17.7%)	
51-60	3407 (22.4%)	35 (15.5%)	3442 (22.3%)	
61-70	2957 (19.4%)	42 (18.6%)	2999 (19.4%)	
71-80	1980 (13.0%)	43 (19.0%)	2023 (13.1%)	
80+	1353 (8.9%)	57 (25.2%)	1410 (9.1%)	
**All-cause mortality**				<0.001
Survivor	14845 (97.6%)	164 (72.6%)	15009 (97.2%)	
Deceased	369 (2.4%)	62 (27.4%)	431 (2.8%)	
Comorbidities				
**Diabetes**	5854 (38.5%)	148 (65.5%)	6002 (38.9%)	<0.001
**Hypertension**	9759 (64.1%)	193 (85.4%)	9952 (64.5%)	<0.001
**Cholesterol**	8227 (54.1%)	156 (69.0%)	8383 (54.3%)	<0.001
**Cardiovascular**	4283 (28.2%)	131 (58.0%)	4414 (28.6%)	<0.001
**Renal**	3094 (20.3%)	110 (48.7%)	3204 (20.8%)	<0.001
**Respiratory**	4574 (30.1%)	111 (49.1%)	4685 (30.3%)	<0.001
**Number of comorbidities**				<0.001
None	2410 (15.8%)	8 (3.5%)	2418 (15.7%)	
1	2924 (19.2%)	13 (5.8%)	2937 (19.0%)	
2	3039 (20.0%)	28 (12.4%)	3067 (19.9%)	
3 or more	6841 (45.0%)	177 (78.3%)	7018 (45.5%)	
Lifestyle factors				
**Smoker**				<0.001
Not available	436 (2.9%)	2 (0.9%)	438 (2.8%)	
Never	6425 (42.2%)	84 (37.2%)	6509 (42.2%)	
Past	5110 (33.6%)	114 (50.4%)	5224 (33.8%)	
Current	3243 (21.3%)	26 (11.5%)	3269 (21.2%)	
**Drinker**				0.021
Not available	2505 (16.5%)	28 (12.4%)	2533 (16.4%)	
Never	3857 (25.4%)	58 (25.7%)	3915 (25.4%)	
Past	2145 (14.1%)	47 (20.8%)	2192 (14.2%)	
Current	6707 (44.1%)	93 (41.2%)	6800 (44.0%)	
**Substance user**				<0.001
Not available	7686 (50.5%)	99 (43.8%)	7785 (50.4%)	
Never	3606 (23.7%)	29 (12.8%)	3635 (23.5%)	
Past	403 (2.6%)	13 (5.8%)	416 (2.7%)	
Current	3519 (23.1%)	85 (37.6%)	3604 (23.3%)	
**Obese**				<0.001
Not available	406 (2.7%)	1 (0.4%)	407 (2.6%)	
Never	6715 (44.1%)	85 (37.6%)	6800 (44.0%)	
Past	2199 (14.5%)	51 (22.6%)	2250 (14.6%)	
Current	5894 (38.7%)	89 (39.4%)	5983 (38.8%)	

For peer review only

Prescription medication use				
**ACE inhibitor**				<0.001
Non-user	12024 (79.0%)	161 (71.2%)	12185 (78.9%)	
Past user	518 (3.4%)	28 (12.4%)	546 (3.5%)	
Current user	2672 (17.6%)	37 (16.4%)	2709 (17.5%)	
**Angiotensin receptor blocker**				0.024
Non-user	13530 (88.9%)	188 (83.2%)	13718 (88.8%)	
Past user	227 (1.5%)	5 (2.2%)	232 (1.5%)	
Current user	1457 (9.6%)	33 (14.6%)	1490 (9.7%)	
**Aldosterone antagonist**				<0.001
Non-user	14651 (96.3%)	205 (90.7%)	14856 (96.2%)	
Past user	137 (0.9%)	9 (4.0%)	146 (0.9%)	
Current user	426 (2.8%)	12 (5.3%)	438 (2.8%)	
**β-blocker**				<0.001
Non-user	12161 (79.9%)	145 (64.2%)	12306 (79.7%)	
Past user	410 (2.7%)	12 (5.3%)	422 (2.7%)	
Current user	2643 (17.4%)	69 (30.5%)	2712 (17.6%)	
**Calcium channel blocker**				0.005
Non-user	11714 (77.0%)	158 (69.9%)	11872 (76.9%)	
Past user	581 (3.8%)	17 (7.5%)	598 (3.9%)	
Current user	2919 (19.2%)	51 (22.6%)	2970 (19.2%)	
**α-agonist**				0.837
Non-user	15131 (99.5%)	225 (99.6%)	15356 (99.5%)	
Past user	23 (0.2%)	0 (0.0%)	23 (0.1%)	
Current user	60 (0.4%)	1 (0.4%)	61 (0.4%)	
**Thiazide**				0.759
Non-user	15131 (99.5%)	225 (99.6%)	15356 (99.5%)	
Past user	32 (0.2%)	0 (0.0%)	32 (0.2%)	
Current user	51 (0.3%)	1 (0.4%)	52 (0.3%)	
**Antiplatelet**				<0.001
Non-user	12512 (82.2%)	147 (65.0%)	12659 (82.0%)	
Past user	446 (2.9%)	10 (4.4%)	456 (3.0%)	
Current user	2256 (14.8%)	69 (30.5%)	2325 (15.1%)	
**Antiarrhythmic**				<0.001
Non-user	14440 (94.9%)	199 (88.1%)	14639 (94.8%)	
Past user	156 (1.0%)	7 (3.1%)	163 (1.1%)	
Current user	618 (4.1%)	20 (8.8%)	638 (4.1%)	
**Anticoagulant**				0.008
Non-user	14613 (96.0%)	208 (92.0%)	14821 (96.0%)	
Past user	144 (0.9%)	5 (2.2%)	149 (1.0%)	
Current user	457 (3.0%)	13 (5.8%)	470 (3.0%)	
**Glucocorticoid**				<0.001
Non-user	10878 (71.5%)	122 (54.0%)	11000 (71.2%)	
Past user	1278 (8.4%)	22 (9.7%)	1300 (8.4%)	

For peer review only

Current user	3058 (20.1%)	82 (36.3%)	3140 (20.3%)	
**β2-agonist**				<0.001
Non-user	13443 (88.4%)	172 (76.1%)	13615 (88.2%)	
Past user	286 (1.9%)	8 (3.5%)	294 (1.9%)	
Current user	1485 (9.8%)	46 (20.4%)	1531 (9.9%)	
**Muscarinic antagonist**				<0.001
Non-user	13531 (88.9%)	175 (77.4%)	13706 (88.8%)	
Past user	300 (2.0%)	10 (4.4%)	310 (2.0%)	
Current user	1383 (9.1%)	41 (18.1%)	1424 (9.2%)	
**NSAID**				0.117
Non-user	13703 (90.1%)	198 (87.6%)	13901 (90.0%)	
Past user	756 (5.0%)	10 (4.4%)	766 (5.0%)	
Current user	755 (5.0%)	18 (8.0%)	773 (5.0%)	
**Vitamin D**				<0.001
Non-user	12542 (82.4%)	139 (61.5%)	12681 (82.1%)	
Past user	573 (3.8%)	18 (8.0%)	591 (3.8%)	
Current user	2099 (13.8%)	69 (30.5%)	2168 (14.0%)	
**Proton pump inhibitor**				<0.001
Non-user	8332 (54.8%)	85 (37.6%)	8417 (54.5%)	
Past user	1167 (7.7%)	15 (6.6%)	1182 (7.7%)	
Current user	5715 (37.6%)	126 (55.8%)	5841 (37.8%)	
**Statin**				<0.001
Non-user	9128 (60.0%)	86 (38.1%)	9214 (59.7%)	
Past user	592 (3.9%)	16 (7.1%)	608 (3.9%)	
Current user	5494 (36.1%)	124 (54.9%)	5618 (36.4%)	
**Immunosuppressant**				0.103
Non-user	14722 (96.8%)	213 (94.2%)	14935 (96.7%)	
Past user	204 (1.3%)	5 (2.2%)	209 (1.4%)	
Current user	288 (1.9%)	8 (3.5%)	296 (1.9%)	

Values are n (%), unless otherwise specified. *Patients with HPB cancer were not included in the non-malignant disease groups.

For peer review only

For peer review only - http://bmjopen.bmj.com/site/about/guidelines.xhtml

Table 3 Differences in demographic, comorbidity, lifestyle, medication use, and post diagnosis complications characteristics between COVID-19 survivor and deceased groups.

	Survivor (N=164)	Deceased (N=53)	Total (N=217)	P value
Demographics				
**Gender**				0.005
Female	82 (50.0%)	15 (28.3%)	97 (44.7%)	
Male	82 (50.0%)	38 (71.7%)	120 (55.3%)	
**Ethnic origin**				0.053
White	74 (45.1%)	20 (37.7%)	94 (43.3%)	
South Asian	48 (29.3%)	18 (34.0%)	66 (30.4%)	
Black	22 (13.4%)	14 (26.4%)	36 (16.6%)	
Other	18 (11.0%)	1 (1.9%)	19 (8.8%)	
Unknown	2 (1.2%)	0 (0.0%)	2 (0.9%)	
**Age**				<0.001
Median	62.94	80.38	67.17	
Q1, Q3	49.81, 77.38	71.72, 85.12	55.00, 81.07	
**Age group**				<0.001
18-40	21 (12.8%)	1 (1.9%)	22 (10.1%)	
41-50	23 (14.0%)	2 (3.8%)	25 (11.5%)	
51-60	31 (18.9%)	2 (3.8%)	33 (15.2%)	
61-70	33 (20.1%)	8 (15.1%)	41 (18.9%)	
71-80	25 (15.2%)	16 (30.2%)	41 (18.9%)	
80+	31 (18.9%)	24 (45.3%)	55 (25.3%)	
**HPB cancer**				0.594
No	161 (98.2%)	51 (96.2%)	212 (97.7%)	
Yes	3 (1.8%)	2 (3.8%)	5 (2.3%)	
**Pancreatic disease***				0.039
No	129 (80.1%)	33 (64.7%)	162 (76.4%)	
Acute	14 (8.7%)	5 (9.8%)	19 (9.0%)	
Chronic	18 (11.2%)	13 (25.5%)	31 (14.6%)	
**Liver disease***				0.039
No	54 (33.5%)	27 (52.9%)	81 (38.2%)	
Mild	88 (54.7%)	19 (37.3%)	107 (50.5%)	
Moderate/Severe	19 (11.8%)	5 (9.8%)	24 (11.3%)	
**Biliary disease***				0.101
No	99 (61.5%)	26 (51.0%)	125 (59.0%)	
Acute	5 (3.1%)	5 (9.8%)	10 (4.7%)	
Chronic	57 (35.4%)	20 (39.2%)	77 (36.3%)	
**Survival**				<0.001
Median	47	11	35	
Q1, Q3	28.75, 66.00	2.00, 18.00	13.00, 59.00	

For peer review only

Comorbidities				
**Diabetes**	99 (60.4%)	42 (79.2%)	141 (65.0%)	0.012
**Hypertension**	132 (80.5%)	52 (98.1%)	184 (84.8%)	0.002
**Cholesterol**	106 (64.6%)	41 (77.4%)	147 (67.7%)	0.085
**Cardiovascular**	84 (51.2%)	43 (81.1%)	127 (58.5%)	<0.001
**Renal**	67 (40.9%)	38 (71.7%)	105 (48.4%)	<0.001
**Respiratory**	80 (48.8%)	27 (50.9%)	107 (49.3%)	0.784
**Number of comorbidities**				<0.001
None	8 (4.9%)	0 (0.0%)	8 (3.7%)	
1	13 (7.9%)	0 (0.0%)	13 (6.0%)	
2	26 (15.9%)	1 (1.9%)	27 (12.4%)	
3 or more	117 (71.3%)	52 (98.1%)	169 (77.9%)	
Lifestyle factors				
**Smoker**				0.008
Not available	2 (1.2%)	0 (0.0%)	2 (0.9%)	
Never	67 (40.9%)	14 (26.4%)	81 (37.3%)	
Past	72 (43.9%)	37 (69.8%)	109 (50.2%)	
Current	23 (14.0%)	2 (3.8%)	25 (11.5%)	
**Drinker**				0.897
Not available	21 (12.8%)	6 (11.3%)	27 (12.4%)	
Never	44 (26.8%)	12 (22.6%)	56 (25.8%)	
Past	32 (19.5%)	12 (22.6%)	44 (20.3%)	
Current	67 (40.9%)	23 (43.4%)	90 (41.5%)	
**Substance user**				0.055
Not available	77 (47.0%)	19 (35.8%)	96 (44.2%)	
Never	24 (14.6%)	4 (7.5%)	28 (12.9%)	
Past	10 (6.1%)	2 (3.8%)	12 (5.5%)	
Current	53 (32.3%)	28 (52.8%)	81 (37.3%)	
**Obese**				0.184
Not available	0 (0.0%)	1 (1.9%)	1 (0.5%)	
Never	65 (39.6%)	18 (34.0%)	83 (38.2%)	
Past	33 (20.1%)	15 (28.3%)	48 (22.1%)	
Current	66 (40.2%)	19 (35.8%)	85 (39.2%)	
Prescription medication use				
**ACE inhibitor**				0.196
Non-user	122 (74.4%)	33 (62.3%)	155 (71.4%)	
Past user	20 (12.2%)	8 (15.1%)	28 (12.9%)	
Current user	22 (13.4%)	12 (22.6%)	34 (15.7%)	
**Angiotensin receptor blocker**				0.708
Non-user	137 (83.5%)	43 (81.1%)	180 (82.9%)	
Past user	3 (1.8%)	2 (3.8%)	5 (2.3%)	
Current user	24 (14.6%)	8 (15.1%)	32 (14.7%)	
**Aldosterone antagonist**				0.791
Non-user	150 (91.5%)	47 (88.7%)	197 (90.8%)	
Past user	6 (3.7%)	3 (5.7%)	9 (4.1%)	
Current user	8 (4.9%)	3 (5.7%)	11 (5.1%)	

For peer review only

**β-blocker**				0.847
Non-user	106 (64.6%)	32 (60.4%)	138 (63.6%)	
Past user	9 (5.5%)	3 (5.7%)	12 (5.5%)	
Current user	49 (29.9%)	18 (34.0%)	67 (30.9%)	
**Calcium channel blocker**				0.233
Non-user	116 (70.7%)	35 (66.0%)	151 (69.6%)	
Past user	14 (8.5%)	2 (3.8%)	16 (7.4%)	
Current user	34 (20.7%)	16 (30.2%)	50 (23.0%)	
**α-agonist**				0.569
Non-user	163 (99.4%)	53 (100.0%)	216 (99.5%)	
Current user	1 (0.6%)	0 (0.0%)	1 (0.5%)	
**Thiazide**				0.569
Non-user	163 (99.4%)	53 (100.0%)	216 (99.5%)	
Current user	1 (0.6%)	0 (0.0%)	1 (0.5%)	
**Antiplatelet**				0.076
Non-user	112 (68.3%)	28 (52.8%)	140 (64.5%)	
Past user	8 (4.9%)	2 (3.8%)	10 (4.6%)	
Current user	44 (26.8%)	23 (43.4%)	67 (30.9%)	
**Antiarrhythmic**				0.07
Non-user	147 (89.6%)	43 (81.1%)	190 (87.6%)	
Past user	6 (3.7%)	1 (1.9%)	7 (3.2%)	
Current user	11 (6.7%)	9 (17.0%)	20 (9.2%)	
**Anticoagulant**				0.47
Non-user	152 (92.7%)	47 (88.7%)	199 (91.7%)	
Past user	4 (2.4%)	1 (1.9%)	5 (2.3%)	
Current user	8 (4.9%)	5 (9.4%)	13 (6.0%)	
**Glucocorticoid**				0.016
Non-user	95 (57.9%)	19 (35.8%)	114 (52.5%)	
Past user	16 (9.8%)	6 (11.3%)	22 (10.1%)	
Current user	53 (32.3%)	28 (52.8%)	81 (37.3%)	
**β2-agonist**				0.023
Non-user	131 (79.9%)	33 (62.3%)	164 (75.6%)	
Past user	6 (3.7%)	2 (3.8%)	8 (3.7%)	
Current user	27 (16.5%)	18 (34.0%)	45 (20.7%)	
**Muscarinic antagonist**				0.351
Non-user	129 (78.7%)	39 (73.6%)	168 (77.4%)	
Past user	5 (3.0%)	4 (7.5%)	9 (4.1%)	
Current user	30 (18.3%)	10 (18.9%)	40 (18.4%)	
**NSAID**				0.116
Non-user	146 (89.0%)	43 (81.1%)	189 (87.1%)	
Past user	8 (4.9%)	2 (3.8%)	10 (4.6%)	
Current user	10 (6.1%)	8 (15.1%)	18 (8.3%)	
**Vitamin D**				0.076
Non-user	109 (66.5%)	26 (49.1%)	135 (62.2%)	
Past user	12 (7.3%)	6 (11.3%)	18 (8.3%)	
Current user	43 (26.2%)	21 (39.6%)	64 (29.5%)	

For peer review only - http://bmjopen.bmj.com/site/about/guidelines.xhtml

**Proton pump inhibitor**				0.82
Non-user	62 (37.8%)	18 (34.0%)	80 (36.9%)	
Past user	11 (6.7%)	3 (5.7%)	14 (6.5%)	
Current user	91 (55.5%)	32 (60.4%)	123 (56.7%)	
**Statin**				0.006
Non-user	72 (43.9%)	11 (20.8%)	83 (38.2%)	
Past user	12 (7.3%)	3 (5.7%)	15 (6.9%)	
Current user	80 (48.8%)	39 (73.6%)	119 (54.8%)	
**Immunosuppressant**				0.476
Non-user	156 (95.1%)	48 (90.6%)	204 (94.0%)	
Past user	3 (1.8%)	2 (3.8%)	5 (2.3%)	
Current user	5 (3.0%)	3 (5.7%)	8 (3.7%)	
Complications post diagnosis				
**Cardiovascular**				<0.001
No	66 (40.2%)	9 (17.0%)	75 (34.6%)	
Recurrent	84 (51.2%)	43 (81.1%)	127 (58.5%)	
Novel	14 (8.5%)	1 (1.9%)	15 (6.9%)	
**Respiratory**				0.003
No	49 (29.9%)	5 (9.4%)	54 (24.9%)	
Recurrent	80 (48.8%)	27 (50.9%)	107 (49.3%)	
Novel	35 (21.3%)	21 (39.6%)	56 (25.8%)	
**Renal**				<0.001
No	67 (40.9%)	8 (15.1%)	75 (34.6%)	
Recurrent	67 (40.9%)	38 (71.7%)	105 (48.4%)	
Novel	30 (18.3%)	7 (13.2%)	37 (17.1%)	
**Recurrent complications**				<0.001
None	43 (26.2%)	2 (3.8%)	45 (20.7%)	
1	43 (26.2%)	10 (18.9%)	53 (24.4%)	
2	46 (28.0%)	25 (47.2%)	71 (32.7%)	
3	32 (19.5%)	16 (30.2%)	48 (22.1%)	
**Novel complications**				0.691
None	99 (60.4%)	28 (52.8%)	127 (58.5%)	
1	52 (31.7%)	21 (39.6%)	73 (33.6%)	
2	12 (7.3%)	4 (7.5%)	16 (7.4%)	
3	1 (0.6%)	0 (0.0%)	1 (0.5%)	

Values are n (%), unless otherwise specified. * Patients with HPB cancer were not included in the non-malignant disease groups.

Odds of SARS-CoV-2 infection

The risk analyses showed a greater odds of COVID-19 for men, the Black community, and those with moderate to severe liver disease (figure 2). Patients with chronic pancreatic and mild liver conditions were also associated with a higher odds of infection (OR 1.89, 95% CI 1.25 to 2.85, P=0.007; 1.52, 1.07 to 2.15, P=0.039); however, post-hoc adjustment for comorbidities returned a reduced non-significant positive odds (1.57, 1.04 to 2.38, P=0.084; 1.32, 0.93 to 1.88, P=0.237), with diabetes principally responsible for this reduction (supplemental table 4). The similar association was observed for elderly patients (over 70) with underlying multimorbidity

For peer review only

as confounding factor. Patients with pre-existing renal conditions had the highest odds of COVID-19 (2.93, 2.2 to 3.89, P<0.001), followed by a more than two-fold increased odds for patients with hypertension, diabetes, cardiovascular or chronic respiratory disease (figure 2). However, the independent effects of hypertension and high cholesterol were absent when controlled for other comorbidities (supplemental table 4).

Substance mis-users had higher odds of infection, but the higher odds observed for those with history of smoking or obesity were due to underlying comorbidities. Patients on Vitamin D treatment and past users of ACE inhibitors were associated with higher odds of infection. The slightly reduced yet significantly high odds remained after controlling for comorbidities, with renal (for Vitamin D users) and cardiovascular (for ACE inhibitor users) diseases being the principal source of the reduced estimates (supplemental table 4). Higher odds were also observed for users of proton pump inhibitors, glucocorticoid, β-blockers, β2-agonists, aldosterone antagonist, muscarinic antagonist, antiplatelet, antiarrhythmic, and statin compared to the non-users of these respective drugs; however, post-hoc adjustment for comorbidities returned non-significant positive odds for those.

<<Figure 2 here>>

Figure 2 Odds ratio estimates of COVID-19 for HPB patients with specific demographic, comorbidity, lifestyle and medication use characteristics. Odds ratio estimates for demographic characteristics are mutually controlled for each other, i.e., gender, ethnicity, and age group. Estimates for HPB disease subgroups are further controlled for each other. For comorbidity, lifestyle and medication use characteristics, estimates are controlled for gender, ethnicity, and dichotomous age group (under and over 60).

A small number of factors appeared to modify the association between HPB disease subgroups and risk of COVID-19 infection (supplemental table 5). In patients with mild liver disease, the odds of COVID-19 infection doubled for chronic pancreatic disease patients compared to patients with no pancreatic condition (P value for heterogeneity, P-het, by liver disease=0.02). A history of substance-misuse was associated with significantly higher odds of infection, particularly for patients with chronic biliary conditions (P-het by biliary disease=0.03), and mild liver conditions (P-het by liver disease=0.04).

Odds of COVID-19 related death

The risk analyses showed an increased odds of COVID-19 related death for men, individuals from the Black community and patients who had acute respiratory complications during admitted care without a history of long-standing respiratory problems (figure 3). Increased odds of death were also observed for the glucocorticoid and β2-agonists. No HPB disease subgroups were particularly more vulnerable to COVID-related death, although patients with chronic pancreatic condition showed a trend towards significance. Elderly patients (over 70), and recent users of ACE inhibitors and non-steroidal anti-inflammatory drugs were associated with a higher odds of death; however, post-hoc adjustment for comorbidities returned a non-significant positive odds for these risk factors (supplemental table 6). Stratified

For peer review only - http://bmjopen.bmj.com/site/about/guidelines.xhtml

analyses according to HPB disease subtypes did not reveal any meaningful effect modification, principally due to small EL-HPB-COVID sample size (data not shown).

<<Figure 3 here>>

Figure 3 Odds ratio estimates of COVID-19 related death for HPB patients with specific demographic, comorbidity, lifestyle, medication use and post COVID-19 diagnosis complication characteristics. Odds ratio estimates for demographic characteristics are mutually controlled for each other, i.e., gender, ethnicity, and age group. Estimates for HPB disease subgroups are further controlled for each other. For comorbidity, lifestyle, medication use and post diagnosis complication characteristics, estimates are controlled for gender, ethnicity, and dichotomous age group (under and over 60). Categories with odds ratio P>0.95 are not shown.

For peer review only - http://bmjopen.bmj.com/site/about/guidelines.xhtml

DISCUSSION

We present, for the first time, data on a large, single-centre, multi-ethnic cohort of HPB patients, where primary, secondary and tertiary care EHRs were integrated to investigate the incidence and outcome of COVID-19, to demonstrate how key demographic characteristics and a range of comorbidities, lifestyle factors and medications are associated with SARS-CoV-2 infection and outcomes in HPB patients.

Comparison with other studies

We noted a higher odds of COVID-19 in patients with prior pancreatic and liver conditions. The higher odds associated with liver conditions is consistent with earlier findings.[6,19] Patients with moderate to severe liver conditions had higher susceptibility to SARS-CoV2 infection than those with milder conditions, which could be due to the increase in abnormalities of immune function with severity of this disease group[32]. We speculate that reduced pancreatic function, particularly in individuals with chronic pancreatic conditions, leading to altered digestion, and therefore gut flora, may make patients more susceptible to pathogens with an enteric route of SARS-CoV2 infection[33,34], and also contribute to the magnitude of COVID-19 severity via modulating host immune responses[35]. Surprisingly the most vulnerable patients with cancer had a low COVID-19 incidence rate, which may reflect the effectiveness of public health interventions such as shielding.[36] However, at the same time, we noted a 17% death rate in this cohort (not due to COVID-19) in six months (supplemental table 3), perhaps indicating the unintended, but potentially inevitable, negative sequelae of social distancing and reduced healthcare provisions for this group of patients as resources were diverted to COVID-19 affected patients.

Men had a higher odds of infection and mortality than women, which is consistent with previous reports,[1,14] and could be due to a favourable genetic predisposition to the virus,[37] and/or gender differences in risk behaviours. Our study also affirms older age, particularly over 70, as an established risk factor for COVID-19 incidence and mortality;[2,4,5] however, this can be largely explained by the presence of multiple comorbidities in the older age groups.[38]

COVID-19 statistics have highlighted a disproportionate effect on BAME ethnic groups with an increased risk of infection and poor outcomes.[13-15] Our results confirm that people from Black community are at a higher risk of both COVID-19 infection and related mortality compared to the White ethnic group. Only a small part of the excess risk in the Black community is explained by multiple comorbidities. Therefore, further variables such as deprivation, occupational exposure, and living conditions might be useful to explore as potential factors behind the apparent vulnerability of the Black population to COVID-19.

All comorbidities such as diabetes, hypertension, high cholesterol, cardiovascular disease, kidney, and respiratory disease, were independently associated with an increased risk of COVID-19, whereas the presence of cardiovascular disease contributed to an added risk of death, concurring with previously reported cohort studies.[4-6,11] Interestingly, our results highlight that for patients without underlying respiratory issues, an acute respiratory episode due to SARS-CoV-2 infection could

For peer review only - http://bmjopen.bmj.com/site/about/guidelines.xhtml

be indicative of a worse outcome. This is in line with previous reports describing an unexpectedly lower prevalence of chronic respiratory conditions among those who had been admitted to hospital due to COVID-19;[39 40] whereas severe outcomes are often a result of respiratory complications, [41 42] such as acute respiratory distress syndrome (ARDS) and respiratory failure.

Smoking leads to severe health consequences, which explains the greater risk observed in our cohort of past smokers with high prevalence of respiratory and cardiovascular diseases. However, current smoking status appeared to have a protective effect in our cohort after adjusting for comorbidities, as has been observed by others, an aspect which cannot be mechanistically explained.[5 43] Carefully designed analyses are needed to explore the association and causality between smoking status (both current and past), associated comorbidities and COVID-19.

Although substance mis-use leads to a plethora of cardio-respiratory and metabolic problems, its role in COVID-19 remains unexplored. To date, this is the first study providing a concrete measure of the risk of COVID-19 for substance mis-users. Our initial results showing that substance mis-users are at a heightened risk for COVID-19 irrespective of the comorbidities warrants a strong case for considering it as an independent risk factor for COVID-19, and may be related to high-risk behavioural patterns. [44 45]

Previous studies have found a significant relationship between obesity and an increased risk of COVID-19,[7] and subsequent hospitalisation,[46] advanced levels of treatment,[15] and death.[4 6] However, our study does not suggest any particular effect of obesity on COVID-19 for patients with HPB conditions, who have a higher prevalence rate of obesity compared to the UK general population (38.8% vs 26%).[47] Our study suggest that the difference in effects for potential susceptibility to COVID-19 for patients with history of obesity are attributed more to other prevalent factors – such as cardiovascular or chronic renal disease [47 48] – which in turn might be the consequences of obesity in these patients' lifetime.

Concerns have been raised regarding the use of various medications with respect to the risk of COVID-19 and the subsequent outcome; and, our analyses contribute to that discussion for some of the widely used prescription drugs. The higher odds observed for the history of various prescription drugs use are consistent with the management of underlying prevalent comorbidities of the study cohort: cardiovascular conditions (ACE inhibitor, β-blocker, aldosterone antagonists, antiplatelet, antiarrhythmic), cholesterol (statin), chronic respiratory diseases (glucocorticoid, β2-agonists, muscarinic antagonist), or background HPB condition (proton pump inhibitor). An important finding from our study is the significant risk observed for vitamin D users, supportive of the possible association between development of COVID-19 and vitamin D deficiency, [49 50] or specific medical conditions (such as kidney failure) where Vitamin D prescription is prevalent. Given that BAME communities are observed to be at a high risk of COVID-19, and there is evidence that vitamin D deficiency is particularly common in these ethnic groups,[49 50] further research on the relationship between vitamin D and COVID-19 is required, with a need to exclude confounding factors such as patients' vitamin D level. Our result also suggest that patients currently taking PPIs are more susceptible to SARS-CoV-2 infection, which concurs with a large population-based online survey conducted in the US.[51] The use of PPIs is highly prevalent in HPB patients for the management of gastrointestinal

For peer review only - http://bmjopen.bmj.com/site/about/guidelines.xhtml

acid-related disorders, and the finding here supports the hypothesis that current use of PPIs might influence the susceptibility to SARS-CoV-2 infection in the gastrointestinal tract through reduction of stomach acid.[51 52]

The literature is conflicted on the potential impact of antihypertensive drugs on COVID-19, particularly those that act as inhibitors to the renin–angiotensin–aldosterone system (RAAS) and upregulate ACE2 expression, suggesting these drugs may be potential risk factors for infection,[53 54] but also as having a protective effect on outcome.[55] However, recent studies found no underlying association between the use of different classes of antihypertensive drugs and the risk of developing COVID-19.[16] With a high percentage of patients with hypertension in the study cohort, our finding that a high risk of COVID-19 is associated with past intake of ACE inhibitors or aldosterone agonists is suggestive of the potential risk of switching from one class of antihypertensive drug to another. This contributes to the debate of whether discontinuation of RAAS inhibitors and considering alternative antihypertensive therapy in times of COVID-19 would be a good practice or not.[56] A marginal association of current use of ACE inhibitors with COVID-19 related death suggests that any increased risk of mortality is likely to be small and will need to be scrutinised in future as more data accumulates.

Our study also shows that recent users of anti-inflammatory drugs, namely glucocorticoid and β2-agonists, had increased odds for COVID-19 and subsequent poor outcome. Controlling for comorbidities resulted in non-significant odds of infection for these patient subgroups, indicating underlying medical conditions - particularly those of respiratory system - to be responsible for the increased susceptibility. However, the observed harmful associations between these drugs and COVID-19-related death could not be explained by a simplified binary representation of underlying six common health conditions. Glucocorticoid drugs, for instance, are used to treat many other inflammatory conditions, notably inflammatory bowel disease (IBD), whereas HPB diseases constitute some of the most common extraintestinal manifestations of IBD. It has been shown that use of corticosteroids is associated with adverse COVID-19 outcomes among patients with IBD.[17] Had it been possible to successfully control for differences in respiratory disease severity or other medical comorbidities, we speculate to see different and possibly non-significant odds of death in these patient subgroups.

Strengths and limitations of the study

A key strength of our study is that we have systematically identified the effect, or the lack of it, of individual demographic and clinical factors on the infection and mortality of COVID-19 in a cohort of over 15000 patients, robustly corrected for potential confounders in their evaluation. Our large population is highly representative of HPB patients from diverse ethnic groups, which contributes to the generalisability of our findings. Another strength is our use of linked electronic health records, harmonised for variations in coding that exist between different EHR systems. We ascertained patient demographics, lifestyle, comorbidities and medications by linking hospital records with pseudo-anonymized longitudinal primary care records, which substantially enrich the data that are recorded on hospital visits.

Retrospective EHR-based COVID-19 studies often suffer from incomplete or missing data on patient characteristics, including key variables such as BMI, ethnicity, smoking

For peer review only - http://bmjopen.bmj.com/site/about/guidelines.xhtml

or pre-existing comorbidities.[4 57] The missing data is particularly applicable to otherwise healthy COVID-19 patients with low use of healthcare services in the past. However, our patient cohort had already been treated or managed at BHNT hospitals at least once, and often referred through primary care, which led to near-complete data for this study, an added advantage of this study. For instance, ethnicity, a common demographic feature, is missing only for 2.8% of cases in our cohort while the rate is significantly higher in other studies (up to 20% of cases).[4 57] The only variable with missing data frequency over 20% in our study is substance mis-use behaviour (50.4%). This is a unique lifestyle risk variable which is not yet explored - understandably due to a lack of recorded data as people often do not disclose this information to their clinicians,[58] unless manifested in physical or mental disorders. Yet, the substance mis-use history of over 7600 patients included in this study provide a good indication of the impact of COVID-19 on this under-studied group.

Our study also has some important limitations. One limitation is the risk of residual confounding or confounding by indication due to unmeasured or simplified binary representation of potential confounding variables. For example, the observed association between Vitamin D users and risk of COVID-19 may be different if participants' Vitamin D level/deficiency status had been taken into consideration. Similarly, the observed association between COVID-19 related death and recent use of glucocorticoid or β2-agonists may reduce or get amplified if respiratory disease severity or other indications for corticosteroid use were considered.

Another critical limitation is associated with the confirmation of East London residency for the study cohort. Patients' addresses (current or historic) are not collected under the umbrella study, which considers patients with HPB conditions (with the exception of cancer) treated or managed at BHNT hospitals as East London residents during the time of their care. The Royal London Hospital hosts one of the largest HPB centres in England, and supports suspected or confirmed HPB cancer patients from nearby geographical areas. As the umbrella study cohort is historic, we acknowledged the probability of people moving away from East London in the meantime. In absence of a patient's current address to confirm their residency at the outset of COVID-19 pandemic in the UK, we relied on an indirect measure to infer residency. We used a strict six-month window preceding the study to identify a patient's interaction with East London GPs or BHNT hospitals. Thus, we believe that any supposed reduction in the cohort size due to unaccounted change of residency within that window should have affected the COVID and non-COVID group in equal proportion, and hence unlikely to alter the findings we report here.

Due to the rarity of the outcome (SARS-CoV-2 infection) in the full HPB cohort, the effects reported in the study could be influenced by the smaller cohort size of COVID-19 cases. We recognise that larger sample sizes of COVID-19 patients are needed to fully understand the effect of SARS-CoV-2 in patients with HPB conditions. Our results are the first step towards this and require validation in similar national and international cohorts.

Conclusions

We believe that the findings from this single-centre study, focusing on patients with a particular medical condition and in an ethnically diverse area, highlight some considerations that could guide clinical care while we await an effective antiviral

For peer review only - http://bmjopen.bmj.com/site/about/guidelines.xhtml

strategy for COVID-19. The current findings reinforce our understanding of some of the important risk factors for SARS-CoV-2 infection but with regards to pre-existing HPB conditions, and allows stratification for risk, thereby providing a tool for policy makers to divert prevention as well as treatment to a clearly identified vulnerable population.

For peer review only

For peer review only - http://bmjopen.bmj.com/site/about/guidelines.xhtml

ACKNOWLEDGEMENTS

ADU is supported by Health Data Research UK (HDR-UK) to conduct the umbrella study EL-PaC-Epidem, which is funded by the UK Medical Research Council. We gratefully acknowledge support provided by Pancreatic Cancer Research Fund (PCRF), for conducting public-patient engagement activity and facilitating ethical approval for EL-PaC-Epidem. We thank Dr Charles Gutteridge, Chief Clinical Information Officer at Barts Health NHS Trust for his help with the collection of secondary and tertiary care data. We thank Dr Kambiz Boomla, Dr John Robson, Prof Carol Dezateux, and members of the Discovery East London Programme Board, and developers at Learning Health Solutions Ltd for their support in facilitating collection of primary care patient records. Finally, we acknowledge the contribution to the research made by several members of the PCRF Tissue Bank team, Bioinformatics Unit and clinical research fellows at Barts Cancer Institute through insightful medical and scientific discussion.

FOOTNOTES

Contributors: ADU designed the study, and was responsible for undertaking and completing data collection, processing and analysis. HMK and CC oversaw the conduct and management of the study. ADU, LS, HMK and CC contributed to the selection of study variables and interpretation post analysis. ADU wrote the first drafts of the report and all the authors made critical revisions.

Funding: The study is conducted under an umbrella study, focusing on the epidemiology of pancreatic and other hepatobiliary cancers in East London (EL-PaC-Epidem), funded by Medical Research Council UK (Ref: MR/S003835/1) as a UKRI/Rutherford Fellowship to the corresponding author. No additional funding has been received for this study.

Competing interests: All authors declare no competing interests.

Ethics approval: All data utilised for this study were collected and processed under the EL-PaC-Epidem study at Barts Health NHS Trust. The study was approved by the East of England - Essex Research Ethics Committee (19/EE/0163; 17 May 2019) and supported by the NHS Confidentiality Advisory Group for collecting and processing confidential patient information without consent (19/CAG/0219; 17 January 2020).

Data sharing: All statistical data relevant to the study are included in the article or uploaded as supplementary information. Only the corresponding author had full access to all the participants' data in the study. The authors confirm that researchers seeking the completely anonymised final analysis dataset for this work can submit a data request to the corresponding author.

Transparency statement: The corresponding author affirms that the manuscript is an honest, accurate, and transparent account of the study being reported; that no important aspects of the study have been omitted; and that any discrepancies from the study as planned (and, if relevant, registered) have been explained.

For peer review only - http://bmjopen.bmj.com/site/about/guidelines.xhtml

Dissemination to participants and related patient and public communities: Key findings will be disseminated in the EL-PaC-Epidem study website as well as in the corresponding author's institute website.

For peer review only

For peer review only - http://bmjopen.bmj.com/site/about/guidelines.xhtml

REFERENCES

1 World Health Organization. Coronavirus disease (COVID-19): Situation Reports. https://www.who.int/emergencies/diseases/novel-coronavirus-2019/situation-reports (accessed July 30, 2020).

2 Centers for Disease Control and Prevention. Coronavirus Disease 2019 (COVID-19): People at Increased Risk and Other People Who Need to Take Extra Precautions. https://www.cdc.gov/coronavirus/2019-ncov/need-extra-precautions/index.html (accessed August 10, 2020).

3 NHS England. Coronavirus (COVID-19): People at Higher Risk from Coronavirus. https://www.nhs.uk/conditions/coronavirus-covid-19/people-at-higher-risk/whos-at-higher-risk-from-coronavirus/ (accessed July 30, 2020)

4 Williamson EJ, Walker AJ, Bhaskaran K, et al. Factors associated with COVID-19-related death using OpenSAFELY. *Nature* 2020; **584** (7821): 430–36.

5 Deng G, Yin M, Chen X, Zeng F. Clinical determinants for fatality of 44,672 patients with COVID-19. *Crit Care* 2020; **24**: 179.

6 Docherty AB, Harrison EM, Green CA, et al. Features of 20 133 UK patients in hospital with covid-19 using the ISARIC WHO Clinical Characterisation Protocol: prospective observational cohort study. *BMJ* 2020; **369**: m1985.

7 Lighter J, Phillips M, Hochman S, et al. Obesity in Patients Younger Than 60 Years Is a Risk Factor for COVID-19 Hospital Admission. *Clin Infect Dis* 2020; **71** (15): 896–7.

8 Dai M, Liu D, Liu M, et al. Patients with Cancer Appear More Vulnerable to SARS-CoV-2: A Multicenter Study during the COVID-19 Outbreak. *Cancer Discov* 2020; **10** (6): 783–91.

9 Blanco JL, Ambrosioni J, Garcia F, et al. COVID-19 in patients with HIV: clinical case series. *Lancet HIV* 2020; **7** (5): e314–6.

10 BMJ Best Practice. Coronavirus disease 2019 (COVID-19) Complications. https://bestpractice.bmj.com/topics/en-gb/3000168/complications (accessed July 23, 2020)

11 Guan W-J, Liang W-H, Zhao Y, et al. Comorbidity and its impact on 1590 patients with COVID-19 in China: a nationwide analysis. *Eur Respir J* 2020; **55** (5): 2000547.

12 Miyara, M. et al. Low incidence of daily active tobacco smoking in patients with symptomatic COVID-19. *Qeios* 2020; doi: 10.32388/WPP19W.3

13 Platt L, Warwick R. Are some ethnic groups more vulnerable to COVID-19 than others? Institute for Fiscal Studies, May, 2020; https://www.ifs.org.uk/inequality/wp-content/uploads/2020/04/Are-some-ethnic-groups-more-vulnerable-to-COVID-19-than-others-V2-IFS-Briefing-Note.pdf (accessed July 23, 2020)

14 Public Health England. Disparities in the risk and outcomes of COVID-19. https://assets.publishing.service.gov.uk/government/uploads/system/uploads/attachment_data/file/889195/disparities_review.pdf August, 2020. (accessed August 14, 2020).

15 Intensive Care National Audit and Research Centre. ICNARC report on COVID-19 in critical care. https://www.icnarc.org/DataServices/Attachments/Download/af7be2d4-bdcd-ea11-9127-00505601089b July 24, 2020. (accessed August 2, 2020).

16 National Institute for Health and Care Excellence. Coronavirus (COVID-19) Rapid Evidence Summaries. https://www.nice.org.uk/covid-19#rapid-es (accessed July 16, 2020)

For peer review only - http://bmjopen.bmj.com/site/about/guidelines.xhtml

17 Brenner EJ, Ungaro RC, Gearry RB, et al., Corticosteroids, but not TNF Antagonists, are Associated with Adverse COVID-19 Outcomes in Patients With Inflammatory Bowel Diseases: Results from an International Registry. *Gastroenterology* 2020; **159** (2): 481–91.
18 Zhang H, Kang Z, Gong H, et al. Digestive system is a potential route of COVID-19: an analysis of single-cell coexpression pattern of key proteins in viral entry process. *Gut* 2020; **69**: 1010–8.
19 Mantovani A, Beatrice G, Dalbeni A. Coronavirus disease 2019 and prevalence of chronic liver disease: A meta-analysis. *Liver Int* 2020; **40**(6): 1316–20.
20 Gubatan J, Levitte S, Patel A, et al. Prevalence, risk factors and clinical outcomes of COVID-19 in patients with a history of pancreatitis in Northern California. *Gut* 2020; Published Online: June 3, 2020. doi: 10.1136/gutjnl-2020-321772.
21 McNabb-Baltar J, Jin DX, Grover AS, et al. Lipase elevation in patients with COVID-19. *Am J Gastroenterol* 2020; Published Online: Jun 3, 2020. doi: 10.14309/ajg.0000000000000732.
22 Hadi A, Werge M, Kristiansen KT, et al. Coronavirus Disease-19 (COVID-19) associated with severe acute pancreatitis: case report on three family members. *Pancreatology* 2020; **20** (4): 665–7.
23 Katabathina VS, Flaherty EM, Dasyam AK, et al. "Biliary Diseases with Pancreatic Counterparts": Cross-sectional Imaging Findings. *Radiographics* 2016; **36** (2): 374–92.
24 UK Government. Coronavirus cases in the UK: daily updated statistics. https://www.gov.uk/guidance/coronavirus-covid-19-information-for-the-public (last accessed August 2, 2020)
25 London Datastore. Coronavirus (COVID-19) Cases: Greater London Authority (GLA). https://data.london.gov.uk/dataset/coronavirus--covid-19--cases (accessed August 4, 2020)
26 Office for National Statistics. Deaths involving COVID-19 by local area and socioeconomic deprivation: deaths occurring between 1 March and 30 June 2020. https://www.ons.gov.uk/peoplepopulationandcommunity/birthsdeathsandmarriages/deaths/bulletins/deathsinvolvingcovid19bylocalareasanddeprivation/deathsoccurringbetween1marchand30june2020 July 24, 2020. (accessed August 4, 2020)
27 Barts Health NHS Trust. https://www.bartshealth.nhs.uk/about-us (accessed August 10, 2020)
28 London Datastore. Ethnic Groups by Borough. https://data.london.gov.uk/dataset/ethnic-groups-borough (accessed August 4, 2020)
29 East London Health & Care Partnership. https://www.eastlondonhcp.nhs.uk/aboutus/ (accessed August 4, 2020)
30 Public Health England. Technical summary: Public Health England data series on deaths in people with COVID-19. https://assets.publishing.service.gov.uk/government/uploads/system/uploads/attachment_data/file/908781/Technical_Summary_PHE_Data_Series_COVID-19_Deaths_20200812.pdf (accessed February 10, 2021)
31 Glasheen WP, Cordier T, Gumpina R, eet al. Charlson Comorbidity Index: ICD-9 Update and ICD-10 Translation. *Am Health Drug Benefits* 2019; **12** (4):188-197.
32 Leber B, Mayrhauser U, Rybczynski M, et al. Innate immune dysfunction in acute and chronic liver disease. *Wien Klin Wochenschr*. 2009; **121** (23-24): 732-44.
33 Thaweerat W. Current evidence on pancreatic involvement in SARS-CoV-2 infection. *Pancrteatology* 2020; **20** (5): 1013–4.

For peer review only - http://bmjopen.bmj.com/site/about/guidelines.xhtml

34 Gou W, Fu Y, Yue L, et al. Gut microbiota may underlie the predisposition of healthy individuals to COVID-19. Preprint at *medRxiv 25* April 2020; doi: 10.1101/2020.04.22.20076091.
35 Yeoh YK, Zuo T, Lui GCY, et al. Gut microbiota composition reflects disease severity and dysfunctional immune responses in patients with COVID-19. *Gut* 2021; Preprint published online: January 11, 2021. doi: 10.1136/gutjnl-2020-323020.
36 Public Health England. Guidance on shielding and protecting people who are clinically extremely vulnerable from COVID-19. https://www.gov.uk/government/publications/guidance-on-shielding-and-protecting-extremely-vulnerable-persons-from-covid-19/guidance-on-shielding-and-protecting-extremely-vulnerable-persons-from-covid-19 (accessed July 16, 2020).
37 Gibson WT, Evans DM, An J, Jones SJM. ACE 2 Coding Variants: A Potential X-linked Risk Factor for COVID-19 Disease. Preprint published online: April 14, 2020. doi: https://doi.org/10.1101/2020.04.05.026633
38 World Health Organization, Europe. Statement – Older people are at highest risk from COVID-19, but all must act to prevent community spread. https://www.euro.who.int/en/health-topics/health-emergencies/coronavirus-covid-19/statements/statement-older-people-are-at-highest-risk-from-covid-19,-but-all-must-act-to-prevent-community-spread April 2, 2020. (accessed June 12, 2020)
39 Halpin DMG, Faner R, Sibila O, et al. Do chronic respiratory diseases or their treatment affect the risk of SARS-CoV-2 infection? *Lancet Respir Med* 2020; **8** (5): 436-38.
40 Piroth L, Cottenet J, Mariet AS, et al. Comparison of the characteristics, morbidity, and mortality of COVID-19 and seasonal influenza: a nationwide, population-based retrospective cohort study. *Lancet Respir Med* 2020; Preprint published online: 17 Dec 2020; doi: 10.1016/s2213-2600(20)30527-0.
41 Tanne JH. Covid 19: Patients have many more complications than flu patients, finds US study. *BMJ* 2020; **371**:m4106.
42 Schultze A, Walker AJ, MacKenna B, et al. Risk of COVID-19-related death among patients with chronic obstructive pulmonary disease or asthma prescribed inhaled corticosteroids: an observational cohort study using the OpenSAFELY platform. *Lancet Respir Med* 2020; **8** (11): 1106-20.
43 Rentsch CT, Kidwai-Khan F, Tate JP, et al. Covid-19 testing, hospital admission, and intensive care among 2,026,227 United States veterans aged 54–75 years. Preprint at *medRxiv* April 14, 2020; doi: https://doi.org/10.1101/2020.04.09.20059964.
44 Ornell F, Moura HF, Scherer JN, et al. The COVID-19 pandemic and its impact on substance use: Implications for prevention and treatment. *Psychiatry Res* 2020; **289**: 113096.
45 Dubey MJ, Ghosh R, Chatterjee S, Biswas P, Chattergee S, Dubey S. COVID-19 and addiction. *Diabetes Metab Syndr* 2020; **14** (5): 817–23.
46 Khawaja AP, Warwick AN, Hysi PG, et al. Associations with COVID-19 hospitalisation amongst 406,793 adults: the UK Biobank prospective cohort study. Preprint at *medRxiv* May 11, 2020; doi: https://doi.org/10.1101/2020.05.06.20092957
47 Public Health England. Excess Weight and COVID-19: Insights from new evidence https://assets.publishing.service.gov.uk/government/uploads/system/uploads/attachment_data/file/907966/PHE_insight_Excess_weight_and_COVID-19__FINAL.pdf July, 2020 (accessed August 5, 2020)
48 Kovesdy CP, Furth SL, Zoccali C. Obesity and Kidney Disease. *Can J Kidney Health Dis* 2017; **4**: 2054358117698669.

49 Hastie CE, Mackay DF, Ho F, et al. Vitamin D concentrations and COVID-19 infection in UK Biobank. *Diabetes Metab Syndr* 2020; **14** (4): 561–5.
50 Jain A, Chaurasia R, Sengar NS, et al. Analysis of vitamin D level among asymptomatic and critically ill COVID-19 patients and its correlation with inflammatory markers. *Sci Rep*. 2020; **10** (1): 20191.
51 Almario CV, Chey WD,Spiegel BMR. Increased risk of COVID-19 among users of proton-pump inhibitors. *Am J Gastroenterol* 2020; Preprint at: https://journals.lww.com/ajg/Documents/AJG-20-1811_R1(PUBLISH%20AS%20WEBPART).pdf
52 Lee SW, Yeniova AO, Moon SY, et al. Severe clinical outcomes of COVID-19 associated with proton pump inhibitors: a nationwide cohort study with propensity score matching. *Gut* 2020; Preprint Published Online: July 30, 2020. doi: 10.1136/gutjnl-2020-322248.
53 O'Mara GJ. Could ACE inhibitors and particularly ARBs increase susceptibility to COVID-19 infection? *BMJ* 2020; **368**: m406.
54 Fang L, Karakiulakis G, Roth M. Are patients with hypertension and diabetes mellitus at increased risk for COVID-19 infection? *Lancet Respir Med* 2020; **8** (4): e21.
55 Gurwitz D. Angiotensin receptor blockers as tentative SARS-CoV-2 therapeutics. *Drug Dev Res* 2020; **81** (5): 537–40.
56 Ciulla MM. Switching to another antihypertensive effective drug when using ACEIs/ARBs to treat arterial hypertension during COVID-19. *Eur Heart J* 2020; **41** (19): 1856.
57 Perez-Guzman PN, Daunt A, Mukherjee S, et al. Clinical characteristics and predictors of outcomes of hospitalized patients with COVID-19 in a multi-ethnic London NHS Trust: a retrospective cohort study. *Clin Infect Dis* 2020; Preprint published online: August 7, 2020. doi: 10.1093/cid/ciaa1091.
58 McNeely J, Kumar PC, Rieckmann T, et al. Barriers and facilitators affecting the implementation of substance use screening in primary care clinics: a qualitative study of patients, providers, and staff. *Addict Sci Clin Pract* 2020; **13** (1): 8.

For peer review only - http://bmjopen.bmj.com/site/about/guidelines.xhtml

FIGURE LEGENDS

Figure 1 Selection of patients for the cross-sectional study.

Figure 2 Odds ratio estimates of COVID-19 for HPB patients with specific demographic, comorbidity, lifestyle and medication use characteristics. Odds ratio estimates for demographic characteristics are mutually controlled for each other, i.e., gender, ethnicity, and age group. Estimates for HPB disease subgroups are further controlled for each other. For comorbidity, lifestyle and medication use characteristics, estimates are controlled for gender, ethnicity, and dichotomous age group (under and over 60).

Figure 3 Odds ratio estimates of COVID-19 related death for HPB patients with specific demographic, comorbidity, lifestyle, medication use and post COVID-19 diagnosis complication characteristics. Odds ratio estimates for demographic characteristics are mutually controlled for each other, i.e., gender, ethnicity, and age group. Estimates for HPB disease subgroups are further controlled for each other. For comorbidity, lifestyle, medication use and post diagnosis complication characteristics, estimates are controlled for gender, ethnicity, and dichotomous age group (under and over 60). Categories with odds ratio P>0.95 are not shown.

For peer review only

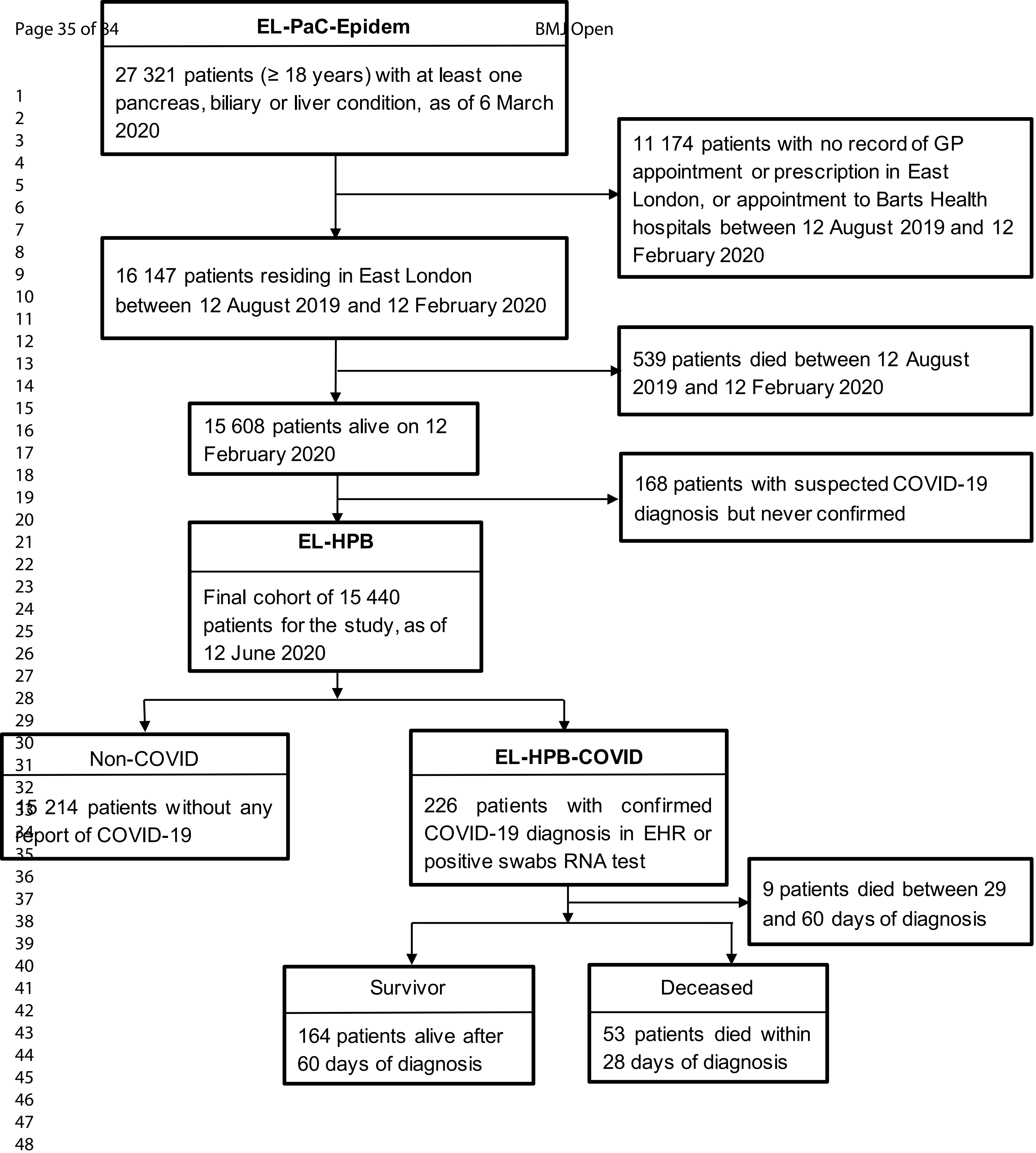

Figure 1 Selection of patients for the cross-sectional study.

	OR	95% CI	P val
**Demography**			
**Gender (ref=Female)**			
Male	1.56	1.2 to 2.04	0.002
**Ethnicity (ref=White)**			
South Asian	1.35	0.98 to 1.85	0.102
Black	2.04	1.39 to 2.95	<0.001
Other	0.87	0.52 to 1.4	0.654
**Age group (ref=18-40)**			
41-50	1.12	0.64 to 1.98	0.694
51-60	1.19	0.7 to 2.05	0.632
61-70	1.71	1.03 to 2.9	0.075
71-80	2.71	1.63 to 4.6	<0.001
80+	5.21	3.23 to 8.7	<0.001
**HPB disease (ref=No)**			
**Cancer**			
Yes	1.11	0.46 to 2.69	0.822
**Pancreatic disease**			
Acute	1.35	0.82 to 2.22	0.323
Chronic	1.89	1.25 to 2.85	0.007
**Biliary disease**			
Acute	1.1	0.58 to 2.09	0.822
Chronic	1.05	0.75 to 1.46	0.822
**Liver disease**			
Mild	1.52	1.07 to 2.15	0.039
Moderate/Severe	2.2	1.35 to 3.59	0.006
**Medical History (ref=No)**			
Diabetes	2.47	1.85 to 3.32	<0.001
Hypertension	2.35	1.59 to 3.48	<0.001
Cholesterol	1.47	1.09 to 1.98	0.019
Cardiovascular	2.77	2.07 to 3.71	<0.001
Renal	2.93	2.2 to 3.89	<0.001
Respiratory	2.06	1.58 to 2.69	<0.001
Number of comorbidities	1.62	1.46 to 1.79	<0.001
**Lifestyle (ref=Never)**			
**Smoker**			
Past	1.46	1.08 to 1.98	0.03
Current	0.65	0.41 to 1.04	0.116
**Drinker**			
Past	1.26	0.85 to 1.88	0.283
Current	0.81	0.56 to 1.16	0.283
**Substance user**			
Past	3.43	1.74 to 6.75	<0.001
Current	2.63	1.68 to 4.1	<0.001
**Obese**			
Past	1.61	1.13 to 2.3	0.016
Current	1.26	0.93 to 1.72	0.187
**Prescription medication use (ref=Non-user)**			
**ACE inhibitor**			
Past user	3	1.97 to 4.57	<0.001
Current user	0.8	0.55 to 1.15	0.25
**Angiotensin receptor blocker**			
Past user	1.18	0.48 to 2.91	0.722
Current user	1.27	0.86 to 1.86	0.293
**Aldosterone antagonist**			
Past user	3.74	1.86 to 7.5	<0.001
Current user	1.61	0.89 to 2.91	0.151
**Beta-blocker**			
Past user	1.95	1.07 to 3.56	0.045
Current user	1.62	1.19 to 2.19	0.004
**Calcium channel blocker**			
Past user	1.5	0.89 to 2.51	0.162
Current user	0.9	0.64 to 1.25	0.533
**Antiplatelet**			
Past user	1.41	0.73 to 2.71	0.345
Current user	1.84	1.35 to 2.51	<0.001
**Antiarrhythmic**			
Past user	2.42	1.11 to 5.27	0.039
Current user	1.85	1.15 to 2.97	0.021
**Anticoagulant**			
Past user	1.96	0.79 to 4.86	0.165
Current user	1.54	0.86 to 2.73	0.165
**Glucocorticoid**			
Past user	1.39	0.88 to 2.2	0.242
Current user	2.07	1.55 to 2.77	<0.001
**Beta2-agonist**			
Past user	2.08	1.01 to 4.27	0.071
Current user	2.07	1.48 to 2.9	<0.001
**Muscarinic antagonist**			
Past user	2.2	1.14 to 4.22	0.027
Current user	1.85	1.3 to 2.64	0.001
**NSAID**			
Past user	0.81	0.42 to 1.54	0.517
Current user	1.56	0.95 to 2.55	0.139
**Vitamin D**			
Past user	2.49	1.5 to 4.13	<0.001
Current user	2.5	1.84 to 3.4	<0.001
**Proton pump inhibitor**			
Past user	1.15	0.66 to 2.01	0.619
Current user	1.78	1.34 to 2.38	<0.001
**Statin**			
Past user	2.1	1.21 to 3.65	0.013
Current user	1.77	1.3 to 2.4	<0.001
**Immunosuppressant**			
Past user	1.42	0.58 to 3.51	0.448
Current user	1.7	0.83 to 3.5	0.191

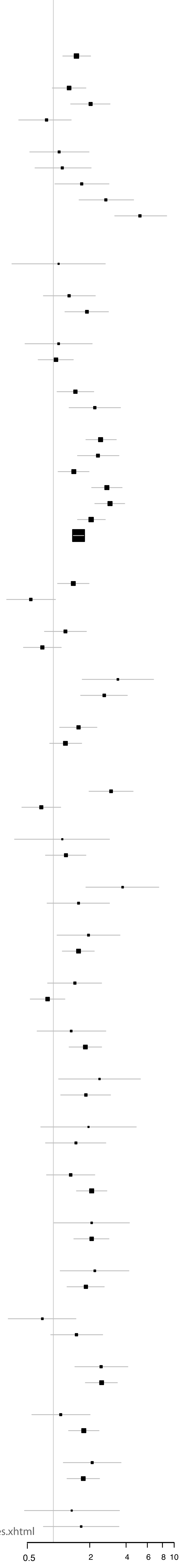

For peer review only

	OR	95% CI	P val
**Demography**			
**Gender (ref=Female)**			
Male	3.54	1.68 to 7.85	0.007
**Ethnicity (ref=White)**			
South Asian	2.08	0.91 to 4.88	0.143
Black	3.77	1.38 to 10.7	0.023
Other	0.29	0.02 to 1.82	0.371
**Age group (ref=18-40)**			
41-50	2.24	0.19 to 52.1	0.65
51-60	1.92	0.16 to 44.3	0.674
61-70	6.73	1.03 to 134	0.143
71-80	18.6	3.12 to 361	0.022
80+	25.4	4.32 to 491	0.012
**HPB disease (ref=No)**			
**Cancer**			
Yes	1.18	0.14 to 10.2	0.951
**Pancreatic disease**			
Acute	1.52	0.44 to 5.24	0.596
Chronic	3.26	1.13 to 9.44	0.082
**Biliary disease**			
Acute	2.44	0.38 to 15.9	0.544
Chronic	0.67	0.26 to 1.72	0.568
**Liver disease**			
Mild	0.51	0.19 to 1.35	0.347
Moderate/Severe	0.6	0.16 to 2.22	0.568
**Medical History (ref=No)**			
Diabetes	1.88	0.81 to 4.38	0.227
Hypertension	6.3	0.74 to 53.8	0.148
Cholesterol	1.25	0.56 to 2.82	0.67
Cardiovascular	2.82	1.21 to 6.59	0.026
Renal	1.72	0.8 to 3.7	0.248
Respiratory	0.85	0.42 to 1.73	0.743
**Lifestyle (ref=Never)**			
**Smoker**			
Past	1.87	0.83 to 4.19	0.219
Current	0.45	0.08 to 2.49	0.451
**Drinker**			
Past	1.2	0.41 to 3.49	0.847
Current	1.38	0.52 to 3.64	0.736
**Substance user**			
Past	0.62	0.08 to 4.94	0.81
Current	2.01	0.53 to 7.64	0.436
**Obese**			
Past	1.46	0.58 to 3.65	0.603
Current	1.03	0.44 to 2.4	0.991
**Prescription medication use (ref=Non-user)**			
**ACE inhibitor**			
Current user	2.25	0.88 to 5.73	0.161
**Aldosterone antagonist**			
Past user	1.29	0.28 to 5.82	0.838
Current user	1.29	0.28 to 5.97	0.838
**Beta-blocker**			
Past user	0.72	0.16 to 3.24	0.746
Current user	0.66	0.3 to 1.45	0.385
**Calcium channel blocker**			
Past user	0.16	0.03 to 0.89	0.065
Current user	0.92	0.4 to 2.12	0.948
**Antiplatelet**			
Past user	0.68	0.12 to 3.7	0.836
**Antiarrhythmic**			
Past user	0.53	0.06 to 4.82	0.641
Current user	2.12	0.72 to 6.26	0.261
**Anticoagulant**			
Past user	0.48	0.05 to 4.89	0.691
Current user	1.18	0.32 to 4.39	0.904
**Glucocorticoid**			
Past user	1.47	0.44 to 4.87	0.599
Current user	2.79	1.26 to 6.22	0.021
**Beta2-agonist**			
Past user	1.96	0.32 to 12	0.523
Current user	2.72	1.18 to 6.25	0.034
**Muscarinic antagonist**			
Past user	1.58	0.32 to 7.7	0.739
Current user	1.11	0.45 to 2.76	0.919
**NSAID**			
Past user	0.58	0.11 to 3.05	0.589
Current user	4.13	1.19 to 14.3	0.045
**Vitamin D**			
Past user	1.8	0.55 to 5.92	0.376
Current user	1.58	0.7 to 3.53	0.345
**Proton pump inhibitor**			
Past user	1.46	0.29 to 7.34	0.83
Current user	0.88	0.41 to 1.89	0.83
**Statin**			
Past user	0.82	0.18 to 3.77	0.902
Current user	1.51	0.62 to 3.66	0.47
**Immunosuppressant**			
Past user	1.3	0.17 to 10	0.904
Current user	2.44	0.44 to 13.7	0.4
**Complications post diagnosis (ref=No)**			
**Cardiovascular**			
Recurrent	2.53	1.04 to 6.12	0.071
Novel	0.44	0.04 to 4.47	0.547
**Respiratory**			
Recurrent	2.53	0.84 to 7.63	0.15
Novel	5.77	1.75 to 19	0.009
**Renal**			
Recurrent	1.72	0.66 to 4.46	0.34
Novel	1	0.3 to 3.34	0.997

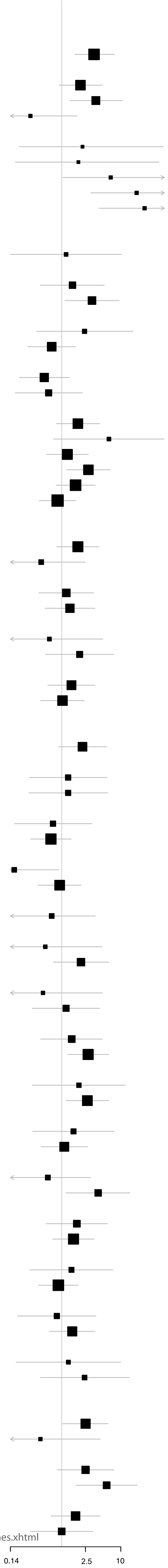

For peer review only

Supplemental Table 1 Codelist for hepato-pancreato-biliary diagnosis groups

Group	Subgroup	Terminology system	Code	Code description	Exclusion
HPB Cancer		ICD-10	C22	Malignant neoplasm of liver and intrahepatic bile ducts	
HPB Cancer		ICD-10	C23	Malignant neoplasm of gallbladder	
HPB Cancer		ICD-10	C24	Malignant neoplasm of other and unspecified parts of biliary tract	
HPB Cancer		ICD-10	C25	Malignant neoplasm of pancreas	
HPB Cancer		ICD-10	D015	Carcinoma in situ of Liver, gallbladder and bile ducts	
HPB Cancer		ICD-10	D017	Carcinoma in situ of Other specified digestive organs incl. Pancreas	
HPB Cancer		ICD-10	D376	Neoplasm of uncertain or unknown behaviour of Liver, gallbladder and bile ducts	
HPB Cancer		READ	B15	Malignant neoplasm of liver and intrahepatic bile ducts	
HPB Cancer		READ	B16	Malignant neoplasm gallbladder and extrahepatic bile ducts	
HPB Cancer		READ	B17	Malignant neoplasm of pancreas	
HPB Cancer		READ	B808.	Carcinoma in situ of liver and biliary system	
HPB Cancer		READ	B8080	Carcinoma in situ of liver	
HPB Cancer		READ	B8081	Carcinoma in situ of intrahepatic bile ducts	
HPB Cancer		READ	B8082	Carcinoma in situ of hepatic duct	
HPB Cancer		READ	B8083	Carcinoma in situ of gall bladder	
HPB Cancer		READ	B8085	Carcinoma in situ of common bile duct	
HPB Cancer		READ	B8086	Carcinoma in situ of ampulla of Vater	
HPB Cancer		READ	B8087	Carcinoma in situ of sphincter of Oddi	
HPB Cancer		READ	B80z0	Carcinoma in situ of pancreas	
HPB Cancer		READ	B903.	Neoplasm of uncertain behaviour of liver and biliary passage	
HPB Cancer		READ	B9030	Neoplasm of uncertain behaviour of liver	
HPB Cancer		READ	B9031	Neoplasm of uncertain behaviour of intra-hepatic bile ducts	

For peer review only

HPB Cancer	READ	B9032	Neoplasm of uncertain behaviour of hepatic duct	
HPB Cancer	READ	B9033	Neoplasm of uncertain behaviour of gall bladder	
HPB Cancer	READ	B9034	Neoplasm of uncertain behaviour of cystic duct	
HPB Cancer	READ	B9035	Neoplasm of uncertain behaviour of common bile duct	
HPB Cancer	READ	B9036	Neoplasm of uncertain behaviour of ampulla of Vater	
HPB Cancer	READ	B9037	Neoplasm of uncertain behaviour of sphincter of Oddi	
HPB Cancer	READ	B9051	Neoplasm of uncertain behaviour of pancreas	
HPB Cancer	READ	Byu10	[X]Other sarcomas of the liver	
HPB Cancer	READ	Byu11	[X]Other specified carcinomas of liver	
HPB Cancer	READ	Byu12	[X]Malignant neoplasm of intestinal tract, part unspecified	
HPB Cancer	SNOMED CT	92545000	Carcinoma in situ of biliary tract (disorder)	
HPB Cancer	SNOMED CT	92644006	Carcinoma in situ of liver (disorder)	
HPB Cancer	SNOMED CT	92672004	Carcinoma in situ of pancreas (disorder)	
HPB Cancer	SNOMED CT	93870000	Malignant neoplasm of liver (disorder)	94381002 (metastasis to liver)
HPB Cancer	SNOMED CT	94910002	Neoplasm of uncertain behavior of liver (disorder)	
HPB Cancer	SNOMED CT	94978003	Neoplasm of uncertain behavior of pancreas (disorder)	
HPB Cancer	SNOMED CT	255064003	Neoplasm of uncertain behavior of biliary system (disorder)	
HPB Cancer	SNOMED CT	363415003	Malignant tumor of biliary tract (disorder)	94185003 (metastasis to biliary tract)
HPB Cancer	SNOMED CT	363418001	Malignant tumor of pancreas (disorder)	94459006 (metastasis to pancreas)
HPB Cancer	CTV3	B15..	Malignant neoplasm of liver and intrahepatic bile ducts	
HPB Cancer	CTV3	B16..	Malignant tumour of biliary tract	
HPB Cancer	CTV3	B162.	Malignant tumour of ampulla of Vater	
HPB Cancer	CTV3	B17..	Malignant tumour of pancreas	B162. (ampullary tumor) X78kd (metastasis to pancreas)

For peer review only

HPB Cancer		CTV3	B80z0	Carcinoma in situ of pancreas	B8086 (ampullary carcinoma in situ)
HPB Cancer		CTV3	B9030	Neoplasm of uncertain behaviour of liver	
HPB Cancer		CTV3	B9031	Neoplasm of uncertain behaviour of intrahepatic bile ducts	
HPB Cancer		CTV3	B9032	Neoplasm of uncertain behaviour of hepatic duct	
HPB Cancer		CTV3	B9033	Neoplasm of uncertain behaviour of gallbladder	
HPB Cancer		CTV3	B9034	Neoplasm of uncertain behaviour of cystic duct	
HPB Cancer		CTV3	B9035	Neoplasm of uncertain behaviour of common bile duct	
HPB Cancer		CTV3	B9036	Neoplasm of uncertain behaviour of ampulla of Vater	
HPB Cancer		CTV3	B9037	Neoplasm of uncertain behaviour of sphincter of Oddi	
HPB Cancer		CTV3	B903z	Neop of uncertain behaviour of liver or biliary passages NOS	
HPB Cancer		CTV3	B9051	Neoplasm of uncertain behaviour of pancreas	
HPB Cancer		CTV3	X78ed	Neoplasm of uncertain behaviour of biliary system	
HPB Cancer		CTV3	X78mC	Carcinoma in situ of biliary tract	
HPB Cancer		CTV3	Xa97q	Malignant tumour of liver	
HPB Cancer		CTV3	XE2ve	Neoplasm of uncertain behaviour of liver and biliary passage	
Pancreatic disease	Acute	ICD-10	K85	Acute pancreatitis	
Pancreatic disease	Acute	ICD-10	K871	Disorders of pancreas in diseases classified elsewhere	
Pancreatic disease	Acute	READ	J670	Acute pancreatitis	
Pancreatic disease	Acute	SNOMED CT	39205007	Infectious pancreatitis (disorder)	838375006 (Chronic)
Pancreatic disease	Acute	SNOMED CT	197456007	Acute pancreatitis (disorder)	
Pancreatic disease	Acute	CTV3	J670.	Acute pancreatitis	
Pancreatic disease	Acute	CTV3	J6704	Subacute pancreatitis	
Pancreatic disease	Acute	CTV3	J670z	Pancreatitis (& [acute NOS])	
Pancreatic disease	Acute	CTV3	Jyu87	[X]Disorders of pancreas in diseases classified elsewhere	
Pancreatic disease	Acute	CTV3	X3092	Pancreatic abscess	
Pancreatic disease	Chronic	ICD-10	D136	Benign neoplasm of Pancreas excl. Endocrine pancreas	

For peer review only

Pancreatic disease	Chronic	ICD-10	D137	Benign neoplasm of Endocrine pancreas
Pancreatic disease	Chronic	ICD-10	K86	Other diseases of pancreas
Pancreatic disease	Chronic	ICD-10	Q450	Agenesis, aplasia and hypoplasia of pancreas
Pancreatic disease	Chronic	ICD-10	Q451	Annular pancreas
Pancreatic disease	Chronic	ICD-10	Q452	Congenital pancreatic cyst
Pancreatic disease	Chronic	ICD-10	Q453	Other congenital malformations of pancreas and pancreatic duct
Pancreatic disease	Chronic	READ	B716	Benign neoplasm of pancreas, excluding islets of Langerhans
Pancreatic disease	Chronic	READ	B717	Benign neoplasm of islets of Langerhans
Pancreatic disease	Chronic	READ	J671	Chronic pancreatitis
Pancreatic disease	Chronic	READ	J672	Cyst and pseudocyst of pancreas
Pancreatic disease	Chronic	READ	J67y	Other diseases of pancreas
Pancreatic disease	Chronic	READ	J67z.	Diseases of pancreas NOS
Pancreatic disease	Chronic	READ	PB7	Anomalies of pancreas
Pancreatic disease	Chronic	SNOMED CT	1835003	Necrosis of pancreas (disorder)
Pancreatic disease	Chronic	SNOMED CT	15402006	Calculus of pancreas (disorder)
Pancreatic disease	Chronic	SNOMED CT	25942009	Fibrosis of pancreas (disorder)
Pancreatic disease	Chronic	SNOMED CT	31258000	Cyst of pancreas (disorder)
Pancreatic disease	Chronic	SNOMED CT	37992001	Pancreatic insufficiency (disorder)
Pancreatic disease	Chronic	SNOMED CT	88281007	Atrophy of pancreas (disorder)
Pancreatic disease	Chronic	SNOMED CT	92264007	Benign neoplasm of pancreas (disorder)
Pancreatic disease	Chronic	SNOMED CT	235494005	Chronic pancreatitis (disorder)
Pancreatic disease	Chronic	SNOMED CT	235977001	Congenital malformation of pancreas (disorder)
Pancreatic disease	Chronic	SNOMED CT	838375006	Chronic infectious pancreatitis (disorder)
Pancreatic disease	Chronic	CTV3	J671.	Chronic pancreatitis
Pancreatic disease	Chronic	CTV3	J672.	Cyst and pseudocyst of pancreas
Pancreatic disease	Chronic	CTV3	J6720	Pancreatic cyst
Pancreatic disease	Chronic	CTV3	J6721	Pseudocyst of pancreas

For peer review only

Pancreatic disease	Chronic	CTV3	J67y0	Atrophy of pancreas
Pancreatic disease	Chronic	CTV3	J67y1	Calculus of pancreas
Pancreatic disease	Chronic	CTV3	J67y2	Fibrosis of pancreas
Pancreatic disease	Chronic	CTV3	J67z.	Diseases of pancreas NOS
Pancreatic disease	Chronic	CTV3	X3093	Pancreatic and peripancreatic necrosis
Pancreatic disease	Chronic	CTV3	X309O	Pancreatic insufficiency
Pancreatic disease	Chronic	CTV3	X309P	Congenital abnormality of pancreas
Pancreatic disease	Chronic	CTV3	X78oE	Benign tumour of pancreas
Pancreatic disease	Chronic	CTV3	XE0dV	(Disease pancreas NOS) or (cyst pancr) or (pseudocyst pancr)
Biliary disease	Acute	ICD-10	K800	Calculus of gallbladder with acute cholecystitis
Biliary disease	Acute	ICD-10	K803	Calculus of bile duct with cholangitis
Biliary disease	Acute	ICD-10	K804	Calculus of bile duct with cholecystitis
Biliary disease	Acute	ICD-10	K810	Acute cholecystitis
Biliary disease	Acute	ICD-10	K820	Obstruction of gallbladder
Biliary disease	Acute	ICD-10	K821	Hydrops of gallbladder
Biliary disease	Acute	ICD-10	K822	Perforation of gallbladder
Biliary disease	Acute	ICD-10	K823	Fistula of gallbladder
Biliary disease	Acute	ICD-10	K830	Cholangitis
Biliary disease	Acute	ICD-10	K831	Obstruction of bile duct
Biliary disease	Acute	ICD-10	K832	Perforation of bile duct
Biliary disease	Acute	ICD-10	K833	Fistula of bile duct
Biliary disease	Acute	READ	J640	Gallbladder calculus with acute cholecystitis
Biliary disease	Acute	READ	J643	Bile duct calculus with acute cholecystitis
Biliary disease	Acute	READ	J644	Bile duct calculus with other cholecystitis
Biliary disease	Acute	READ	J646	Calculus of bile duct with cholangitis
Biliary disease	Acute	READ	J650	Acute cholecystitis
Biliary disease	Acute	READ	J652	Obstruction of gallbladder

For peer review only

Biliary disease	Acute	READ	J653	Mucocele of gallbladder	
Biliary disease	Acute	READ	J654	Perforation of gallbladder	
Biliary disease	Acute	READ	J655	Fistula of gallbladder	
Biliary disease	Acute	READ	J661	Cholangitis	
Biliary disease	Acute	READ	J662	Obstruction of bile duct	
Biliary disease	Acute	READ	J663	Perforation of bile duct	
Biliary disease	Acute	READ	J664	Fistula of bile duct	
Biliary disease	Acute	READ	J666.	Biliary sepsis	
Biliary disease	Acute	SNOMED CT	6215006	Acute cholangitis (disorder)	
Biliary disease	Acute	SNOMED CT	16957005	Fistula of gallbladder (disorder)	
Biliary disease	Acute	SNOMED CT	25345001	Perforation of gallbladder	
Biliary disease	Acute	SNOMED CT	30093007	Calculus of bile duct (disorder)	91316003 (with chronic cholecystitis) 68368005 (with chronic cholecystitis) 4661003 (calculus of bile duct)
Biliary disease	Acute	SNOMED CT	30144000	Obstruction of bile duct (disorder)	
Biliary disease	Acute	SNOMED CT	37439003	Perforation of bile duct (disorder)	
Biliary disease	Acute	SNOMED CT	47312008	Hydrops of gallbladder (disorder)	
Biliary disease	Acute	SNOMED CT	53206008	Fistula of bile duct (disorder)	
Biliary disease	Acute	SNOMED CT	59771005	Calculus of gallbladder with acute cholecystitis (disorder)	
Biliary disease	Acute	SNOMED CT	68368005	Calculus of common bile duct with chronic cholecystitis (disorder)	
Biliary disease	Acute	SNOMED CT	75726005	Obstruction of gallbladder (disorder)	
Biliary disease	Acute	SNOMED CT	750511000000101	Biliary sepsis (disorder)	
Biliary disease	Acute	CTV3	J640.	Gallbladder calculus with acute cholecystitis	
Biliary disease	Acute	CTV3	J643.	Bile duct calculus with acute cholecystitis	
Biliary disease	Acute	CTV3	J650.	(Ac cholecystitis) or (empyema gallblad) or (absc gallblad)	
Biliary disease	Acute	CTV3	J653.	(Mucocele of gallbladder) or (hydrops of gallbladder)	

Biliary disease	Acute	CTV3	J655.	Fistula of gallbladder
Biliary disease	Acute	CTV3	J6550	Biliary tract fistula
Biliary disease	Acute	CTV3	J65y4	Cyst of gallbladder
Biliary disease	Acute	CTV3	J661.	Cholangitis
Biliary disease	Acute	CTV3	X3087	Obstruction of biliary tree
Biliary disease	Acute	CTV3	X308B	Empyema of gallbladder
Biliary disease	Acute	CTV3	X308C	Hydrops of gallbladder
Biliary disease	Acute	CTV3	X308E	Biliary stricture
Biliary disease	Acute	CTV3	X308P	Perforation of biliary tree
Biliary disease	Acute	CTV3	X308V	Obstructive jaundice
Biliary disease	Acute	CTV3	XaAzZ	Dilation of biliary tract
Biliary disease	Acute	CTV3	XaWzz	Biliary sepsis
Biliary disease	Acute	CTV3	XE0bF	Acute cholecystitis
Biliary disease	Acute	CTV3	XE0bG	Mucocele of gallbladder
Biliary disease	Chronic	ICD-10	D135	Benign neoplasm of Extrahepatic bile ducts
Biliary disease	Chronic	ICD-10	K801	Calculus of gallbladder with other cholecystitis
Biliary disease	Chronic	ICD-10	K802	Calculus of gallbladder without cholecystitis
Biliary disease	Chronic	ICD-10	K805	Calculus of bile duct without cholangitis or cholecystitis
Biliary disease	Chronic	ICD-10	K808	Other cholelithiasis
Biliary disease	Chronic	ICD-10	K811	Chronic cholecystitis
Biliary disease	Chronic	ICD-10	K818	Other cholecystitis
Biliary disease	Chronic	ICD-10	K819	Cholecystitis, unspecified
Biliary disease	Chronic	ICD-10	K824	Cholesterolosis of gallbladder
Biliary disease	Chronic	ICD-10	K828	Other specified diseases of gallbladder
Biliary disease	Chronic	ICD-10	K829	Disease of gallbladder, unspecified
Biliary disease	Chronic	ICD-10	K834	Spasm of sphincter of Oddi
Biliary disease	Chronic	ICD-10	K835	Biliary cyst
Biliary disease	Chronic	ICD-10	K838	Other specified diseases of biliary tract

For peer review only

Biliary disease	Chronic	ICD-10	K839	Disease of biliary tract, unspecified
Biliary disease	Chronic	ICD-10	Q440	Agenesis, aplasia and hypoplasia of gallbladder
Biliary disease	Chronic	ICD-10	Q441	Other congenital malformations of gallbladder
Biliary disease	Chronic	ICD-10	Q442	Atresia of bile ducts
Biliary disease	Chronic	ICD-10	Q443	Congenital stenosis and stricture of bile ducts
Biliary disease	Chronic	ICD-10	Q444	Choledochal cyst
Biliary disease	Chronic	ICD-10	Q445	Other congenital malformations of bile ducts
Biliary disease	Chronic	READ	B715.	Benign neoplasm of liver and biliary ducts
Biliary disease	Chronic	READ	B7152	Benign neoplasm of gallbladder
Biliary disease	Chronic	READ	B7155	Benign neoplasm of bile duct
Biliary disease	Chronic	READ	B7156	Benign neoplasm of sphincter of Oddi
Biliary disease	Chronic	READ	B7157	Benign neoplasm of ampulla of Vater
Biliary disease	Chronic	READ	J64..	Cholelithiasis
Biliary disease	Chronic	READ	J641	Gallbladder calculus with other cholecystitis
Biliary disease	Chronic	READ	J642	Gallbladder calculus without mention of cholecystitis
Biliary disease	Chronic	READ	J645	Bile duct calculus without mention of cholecystitis
Biliary disease	Chronic	READ	J64z	Cholelithiasis NOS
Biliary disease	Chronic	READ	J65..	Other gallbladder disorders
Biliary disease	Chronic	READ	J651	Other cholecystitis
Biliary disease	Chronic	READ	J656	Cholesterolosis of gallbladder
Biliary disease	Chronic	READ	J65y	Other specified gallbladder disorders
Biliary disease	Chronic	READ	J65z.	Other gallbladder disorders NOS
Biliary disease	Chronic	READ	J665	Spasm of sphincter of Oddi
Biliary disease	Chronic	READ	J66y	Other bile duct disorders
Biliary disease	Chronic	READ	J66z.	Bile duct disorder NOS
Biliary disease	Chronic	READ	PB601	Gallbladder anomaly, unspecified
Biliary disease	Chronic	READ	PB602	Bile duct anomaly, unspecified
Biliary disease	Chronic	READ	PB61	Biliary atresia

For peer review only

Biliary disease	Chronic	READ	PB640	Duplication of biliary duct
Biliary disease	Chronic	READ	PB641	Duplication of cystic duct
Biliary disease	Chronic	READ	PB642	Duplication of gallbladder
Biliary disease	Chronic	READ	PB6y0	Congenital choledochal cyst
Biliary disease	Chronic	READ	PB6y1	Congenital hepatomegaly
Biliary disease	Chronic	READ	PB6y2	Congenital floating gallbladder
Biliary disease	Chronic	READ	PB6y4	Intrahepatic gallbladder
Biliary disease	Chronic	READ	PB6y5	Hypoplasia of gallbladder
Biliary disease	Chronic	READ	PB6y7	Congenital dilation of bile duct
Biliary disease	Chronic	READ	PB6y8	Congenital diverticulum of bile duct
Biliary disease	Chronic	READ	PB6yx	Other congenital anomaly of gallbladder
Biliary disease	Chronic	SNOMED CT	1698001	Ulcer of bile duct (disorder)
Biliary disease	Chronic	SNOMED CT	4711003	Congenital anomaly of bile ducts (disorder)
Biliary disease	Chronic	SNOMED CT	13516000	Adhesion of gallbladder (disorder)
Biliary disease	Chronic	SNOMED CT	26874005	Hypertrophy of bile duct (disorder)
Biliary disease	Chronic	SNOMED CT	28132005	Spasm of sphincter of Oddi (disorder)
Biliary disease	Chronic	SNOMED CT	49714001	Congenital anomaly of gallbladder (disorder)
Biliary disease	Chronic	SNOMED CT	51854002	Atrophy of bile duct (disorder)
Biliary disease	Chronic	SNOMED CT	59612001	Ulcer of gallbladder (disorder)
Biliary disease	Chronic	SNOMED CT	61565001	Cholesterolosis of gallbladder (disorder)
Biliary disease	Chronic	SNOMED CT	64664008	Atrophy of gallbladder (disorder)
Biliary disease	Chronic	SNOMED CT	71912000	Chronic cholangitis (disorder)
Biliary disease	Chronic	SNOMED CT	76875008	Hypertrophy of gallbladder (disorder)
Biliary disease	Chronic	SNOMED CT	77972001	Adhesion of bile duct (disorder)
Biliary disease	Chronic	SNOMED CT	78900008	Nonfunctioning cystic duct (disorder)
Biliary disease	Chronic	SNOMED CT	80527006	Nonfunctioning gallbladder (disorder)
Biliary disease	Chronic	SNOMED CT	91316003	Calculus of bile duct with chronic cholecystitis (disorder)
Biliary disease	Chronic	SNOMED CT	91991003	Benign neoplasm of biliary tract (disorder)

For peer review only

Biliary disease	Chronic	SNOMED CT	95559000	Chronic cholecystitis with calculus (disorder)
Biliary disease	Chronic	SNOMED CT	204787003	Congenital absence of liver and/or gallbladder (disorder)
Biliary disease	Chronic	SNOMED CT	235924006	Cyst of biliary tract (disorder)
Biliary disease	Chronic	SNOMED CT	253804002	Biliary anomalies (disorder)
Biliary disease	Chronic	SNOMED CT	721721001	Dyskinesia of gallbladder (disorder)
Biliary disease	Chronic	SNOMED CT	722869007	Calculus of gallbladder without cholecystitis or cholangitis (disorder)
Biliary disease	Chronic	CTV3	B715.	Benign neoplasm: [liver & biliary ducts] or [biliary system]
Biliary disease	Chronic	CTV3	J641.	Gallbladder calculus with other cholecystitis
Biliary disease	Chronic	CTV3	J642.	Gallbladder calculus without mention of cholecystitis
Biliary disease	Chronic	CTV3	J644.	Bile duct calculus with other cholecystitis
Biliary disease	Chronic	CTV3	J645.	Bile duct calculus (& [without mention of cholecystitis])
Biliary disease	Chronic	CTV3	J64z.	Cholelithiasis NOS
Biliary disease	Chronic	CTV3	J651.	Other cholecystitis
Biliary disease	Chronic	CTV3	J6510	Chronic cholecystitis
Biliary disease	Chronic	CTV3	J651y	Other cholecystitis OS
Biliary disease	Chronic	CTV3	J651z	Cholecystitis NOS
Biliary disease	Chronic	CTV3	J656.	Cholesterolosis of gallbladder
Biliary disease	Chronic	CTV3	J65y8	Ulcer of gallbladder
Biliary disease	Chronic	CTV3	J65y9	Ulcer of cystic duct
Biliary disease	Chronic	CTV3	J65yA	Non-functioning gallbladder
Biliary disease	Chronic	CTV3	J65yz	Other specified gallbladder disorder NOS
Biliary disease	Chronic	CTV3	J65z.	Other gallbladder disorders NOS
Biliary disease	Chronic	CTV3	J665.	Spasm of sphincter of Oddi
Biliary disease	Chronic	CTV3	Jyu80	[X]Other cholelithiasis
Biliary disease	Chronic	CTV3	Jyu81	[X]Other cholecystitis
Biliary disease	Chronic	CTV3	Jyu82	[X]Other specified diseases of gallbladder
Biliary disease	Chronic	CTV3	X3082	Congenital disorder of gallbladder and biliary tract

For peer review only

Biliary disease	Chronic	CTV3	X308F	Biliary cyst
Biliary disease	Chronic	CTV3	X308L	Cyst of biliary tract
Biliary disease	Chronic	CTV3	X308Q	Adhesions of biliary tree
Biliary disease	Chronic	CTV3	X308R	Atrophy of biliary tree
Biliary disease	Chronic	CTV3	X308S	Hypertrophy of biliary tract
Biliary disease	Chronic	CTV3	X308U	Ulceration of biliary tree
Biliary disease	Chronic	CTV3	X78oB	Benign tumour of biliary tract
Biliary disease	Chronic	CTV3	Xa4g2	Poorly functioning gallbladder
Biliary disease	Chronic	CTV3	XE0bE	Bile duct calculus without mention of cholecystitis
Biliary disease	Chronic	CTV3	XE2xC	Benign neoplasm of liver and biliary ducts
Liver disease	Mild	ICD-10	B18	Chronic viral hepatitis
Liver disease	Mild	ICD-10	B18	Chronic viral hepatitis
Liver disease	Mild	ICD-10	D134	Benign neoplasm of Liver
Liver disease	Mild	ICD-10	K700	Alcoholic fatty liver
Liver disease	Mild	ICD-10	K701	Alcoholic hepatitis
Liver disease	Mild	ICD-10	K702	Alcoholic fibrosis and sclerosis of liver
Liver disease	Mild	ICD-10	K703	Alcoholic cirrhosis of liver
Liver disease	Mild	ICD-10	K709	Alcoholic liver disease, unspecified
Liver disease	Mild	ICD-10	K713	Toxic liver disease with chronic persistent hepatitis
Liver disease	Mild	ICD-10	K714	Toxic liver disease with chronic lobular hepatitis
Liver disease	Mild	ICD-10	K715	Toxic liver disease with chronic active hepatitis
Liver disease	Mild	ICD-10	K716	Toxic liver disease with hepatitis, not elsewhere classified
Liver disease	Mild	ICD-10	K717	Toxic liver disease with fibrosis and cirrhosis of liver
Liver disease	Mild	ICD-10	K718	Toxic liver disease with other disorders of liver
Liver disease	Mild	ICD-10	K719	Toxic liver disease, unspecified
Liver disease	Mild	ICD-10	K73	Chronic hepatitis, not elsewhere classified
Liver disease	Mild	ICD-10	K74	Fibrosis and cirrhosis of liver
Liver disease	Mild	ICD-10	K750	Abscess of liver

For peer review only

Liver disease	Mild	ICD-10	K753	Granulomatous hepatitis, not elsewhere classified
Liver disease	Mild	ICD-10	K758	Other specified inflammatory liver diseases
Liver disease	Mild	ICD-10	K759	Inflammatory liver disease, unspecified
Liver disease	Mild	ICD-10	K760	Fatty (change of) liver, not elsewhere classified
Liver disease	Mild	ICD-10	K762	Central haemorrhagic necrosis of liver
Liver disease	Mild	ICD-10	K763	Infarction of liver
Liver disease	Mild	ICD-10	K764	Peliosis hepatis
Liver disease	Mild	ICD-10	K768	Other specified diseases of liver
Liver disease	Mild	ICD-10	K769	Liver disease, unspecified
Liver disease	Mild	ICD-10	K770	Liver disorders in infectious and parasitic diseases classified elsewhere
Liver disease	Mild	ICD-10	K778	Liver disorders in other diseases classified elsewhere
Liver disease	Mild	ICD-10	Q446	Cystic disease of liver
Liver disease	Mild	ICD-10	Z944	Liver transplant status
Liver disease	Mild	READ	A707	Chronic viral hepatitis
Liver disease	Mild	READ	B715.	Benign neoplasm of liver and biliary ducts
Liver disease	Mild	READ	B7150	Benign neoplasm of liver
Liver disease	Mild	READ	B7151	Benign neoplasm of intrahepatic bile ducts
Liver disease	Mild	READ	B7154	Benign neoplasm of hepatic duct
Liver disease	Mild	READ	B7158	Focal nodular hyperplasia of liver
Liver disease	Mild	READ	J6001	Acute hepatitis - noninfective
Liver disease	Mild	READ	J6011	Subacute hepatitis - noninfective
Liver disease	Mild	READ	J610.	Alcoholic fatty liver
Liver disease	Mild	READ	J611.	Acute alcoholic hepatitis
Liver disease	Mild	READ	J612	Alcoholic cirrhosis of liver
Liver disease	Mild	READ	J614	Chronic hepatitis
Liver disease	Mild	READ	J615	Cirrhosis - non alcoholic
Liver disease	Mild	READ	J616	Biliary cirrhosis

For peer review only

Liver disease	Mild	READ	J617	Alcoholic hepatitis	
Liver disease	Mild	READ	J61y	Other non-alcoholic chronic liver disease	
Liver disease	Mild	READ	J61z.	Chronic liver disease NOS	
Liver disease	Mild	READ	J620	Liver abscess - excluding amoebic liver abscess	
Liver disease	Mild	READ	J62y.	Other sequelae of chronic liver disease	
Liver disease	Mild	READ	J62z.	Liver abscess and chronic liver disease causing sequelae NOS	
Liver disease	Mild	READ	J631	Hepatitis in viral diseases EC	
Liver disease	Mild	READ	J632	Hepatitis in other infectious diseases EC	
Liver disease	Mild	READ	J633	Hepatitis unspecified	
Liver disease	Mild	READ	J634.	Hepatic infarction	
Liver disease	Mild	READ	J6353	Toxic liver disease with chronic persistent hepatitis	
Liver disease	Mild	READ	J6354	Toxic liver disease with chronic lobular hepatitis	
Liver disease	Mild	READ	J6355	Toxic liver disease with chronic active hepatitis	
Liver disease	Mild	READ	J6356	Toxic liver disease with fibrosis and cirrhosis of liver	
Liver disease	Mild	READ	J635X	Toxic liver disease, unspecified	
Liver disease	Mild	READ	J636.	Central haemorrhagic necrosis of liver	
Liver disease	Mild	READ	J638.	Peliosis hepatis	
Liver disease	Mild	READ	J639.	Hepatic granulomas in berylliosis	
Liver disease	Mild	READ	J63A.	Hepatic granulomas in sarcoidosis	
Liver disease	Mild	READ	J63X.	Granulomatous hepatitis, not elsewhere classified	
Liver disease	Mild	READ	J63y0	Hepatoptosis	
Liver disease	Mild	READ	J63y2	Liver cyst	
Liver disease	Mild	SNOMED CT	18027006	Transplantation of liver (procedure)	
Liver disease	Mild	SNOMED CT	27916005	Abscess of liver (disorder)	
Liver disease	Mild	SNOMED CT	41309000	Alcoholic liver damage (disorder)	235881000 (Alcoholic hepatic failure)
Liver disease	Mild	SNOMED CT	50325005	Alcoholic fatty liver (disorder)	

For peer review only

Liver disease	Mild	SNOMED CT	50701000	Hepatoptosis (disorder)	
Liver disease	Mild	SNOMED CT	58008004	Peliosis hepatis (disorder)	
Liver disease	Mild	SNOMED CT	62484002	Hepatic fibrosis (disorder)	
Liver disease	Mild	SNOMED CT	72925005	Congenital cystic disease of liver (disorder)	
Liver disease	Mild	SNOMED CT	76783007	Chronic hepatitis (disorder)	
Liver disease	Mild	SNOMED CT	85057007	Cyst of liver (disorder)	
Liver disease	Mild	SNOMED CT	86514004	Granulomatous hepatitis (disorder)	
Liver disease	Mild	SNOMED CT	87248009	Hepatic necrosis (disorder)	
Liver disease	Mild	SNOMED CT	92186001	Benign neoplasm of liver (disorder)	
Liver disease	Mild	SNOMED CT	128241005	Inflammatory disease of liver (disorder)	69800000 (Neonatal hepatitis) 276553003 (Idiopathic hepatitis in infancy) 276551001 (Perinatal hepatitis)
Liver disease	Mild	SNOMED CT	197321007	Steatosis of liver (disorder)	
Liver disease	Mild	SNOMED CT	197354009	Toxic liver disease (disorder)	197355005 (with cholestasis) 197356006 (with hepatic necrosis) 197358007 (with acute hepatitis)
Liver disease	Mild	SNOMED CT	235875008	Alcoholic hepatitis (disorder)	
Liver disease	Mild	SNOMED CT	240789006	Hepatosplenic schistosomiasis (disorder)	
Liver disease	Mild	SNOMED CT	278527001	Focal nodular hyperplasia of liver (disorder)	
Liver disease	Mild	SNOMED CT	442685003	Nonalcoholic steatohepatitis (disorder)	
Liver disease	Mild	CTV3	B715.	Benign neoplasm: [liver & biliary ducts] or [biliary system]	
Liver disease	Mild	CTV3	B7150	Benign tumour of liver	
Liver disease	Mild	CTV3	J601z	Subacute necrosis of liver NOS	
Liver disease	Mild	CTV3	J614.	Chronic hepatitis	

For peer review only

Liver disease	Mild	CTV3	J61y.	Other non-alcoholic chronic liver disease	
Liver disease	Mild	CTV3	J61y3	Portal fibrosis without cirrhosis	
Liver disease	Mild	CTV3	J61z.	Chronic liver disease NOS	
Liver disease	Mild	CTV3	J62..	Liver abscess and sequelae of chronic liver disease	
Liver disease	Mild	CTV3	J634.	Infarction of liver	
Liver disease	Mild	CTV3	J6353	Toxic liver disease with chronic persistent hepatitis	
Liver disease	Mild	CTV3	J6354	Toxic liver disease with chronic lobular hepatitis	
Liver disease	Mild	CTV3	J6355	Toxic liver disease with chronic active hepatitis	
Liver disease	Mild	CTV3	J6356	Toxic liver disease with fibrosis and cirrhosis of liver	
Liver disease	Mild	CTV3	J639.	Hepatic granulomas in berylliosis	
Liver disease	Mild	CTV3	J63y0	Hepatoptosis	
Liver disease	Mild	CTV3	Jyu71	[X]Other and unspecified cirrhosis of liver	
Liver disease	Mild	CTV3	Jyu74	[X]Liver disorders in infectious and parasitic diseases CE	
Liver disease	Mild	CTV3	Jyu75	[X]Liver disorders in other diseases classified elsewhere	
Liver disease	Mild	CTV3	Jyu76	[X]Toxic liver disease, unspecified	
Liver disease	Mild	CTV3	Jyu77	[X]Granulomatous hepatitis, not elsewhere classified	
Liver disease	Mild	CTV3	PB62.	(Congenital cystic liver disease) or (congenit hepatic cyst)	
Liver disease	Mild	CTV3	X306T	Inflammatory liver disease	X306U (Nonspecific reactive hepatitis)
Liver disease	Mild	CTV3	X306x	Peliosis hepatis	
Liver disease	Mild	CTV3	X3071	Alcoholic liver disease	X3073 (Alcoholic hepatic failure)
Liver disease	Mild	CTV3	X307L	Cirrhosis of liver	
Liver disease	Mild	CTV3	X307v	Fatty change of liver	
Liver disease	Mild	CTV3	Xa0lo	Focal nodular hyperplasia of liver	
Liver disease	Mild	CTV3	Xa8De	Liver necrosis	
Liver disease	Mild	CTV3	XaREa	Liver disease due to cystic fibrosis	
Liver disease	Mild	CTV3	XE1L1	Congenital cystic liver disease	
Liver disease	Mild	CTV3	XE2xC	Benign neoplasm of liver and biliary ducts	

Liver disease	Moderate/Severe	ICD-10	I85	Esophageal varices
Liver disease	Moderate/Severe	ICD-10	I85	Esophageal varices
Liver disease	Moderate/Severe	ICD-10	I864	Gastric varices
Liver disease	Moderate/Severe	ICD-10	I864	Gastric varices
Liver disease	Moderate/Severe	ICD-10	I982	Oesophageal varices without bleeding in diseases classified elsewhere
Liver disease	Moderate/Severe	ICD-10	I982	Oesophageal varices without bleeding in diseases classified elsewhere
Liver disease	Moderate/Severe	ICD-10	K704	Alcoholic hepatic failure
Liver disease	Moderate/Severe	ICD-10	K710	Toxic liver disease with cholestasis
Liver disease	Moderate/Severe	ICD-10	K711	Toxic liver disease with hepatic necrosis
Liver disease	Moderate/Severe	ICD-10	K712	Toxic liver disease with acute hepatitis
Liver disease	Moderate/Severe	ICD-10	K720	Acute and subacute hepatic failure
Liver disease	Moderate/Severe	ICD-10	K721	Chronic hepatic failure
Liver disease	Moderate/Severe	ICD-10	K729	Hepatic failure, unspecified
Liver disease	Moderate/Severe	ICD-10	K751	Phlebitis of portal vein
Liver disease	Moderate/Severe	ICD-10	K752	Nonspecific reactive hepatitis
Liver disease	Moderate/Severe	ICD-10	K754	Autoimmune hepatitis
Liver disease	Moderate/Severe	ICD-10	K761	Chronic passive congestion of liver
Liver disease	Moderate/Severe	ICD-10	K765	Hepatic veno-occlusive disease
Liver disease	Moderate/Severe	ICD-10	K766	Portal hypertension
Liver disease	Moderate/Severe	ICD-10	K767	Hepatorenal syndrome
Liver disease	Moderate/Severe	READ	G850.	Oesophageal varices with bleeding
Liver disease	Moderate/Severe	READ	G851.	Oesophageal varices without bleeding
Liver disease	Moderate/Severe	READ	G852	Oesophageal varices in diseases EC
Liver disease	Moderate/Severe	READ	G857.	Gastric varices
Liver disease	Moderate/Severe	READ	G858.	Oesophageal varices NOS
Liver disease	Moderate/Severe	READ	J6000	Acute hepatic failure
Liver disease	Moderate/Severe	READ	J6010	Subacute hepatic failure

For peer review only

Liver disease	Moderate/Severe	READ	J6130	Alcoholic hepatic failure
Liver disease	Moderate/Severe	READ	J621.	Portal pyaemia
Liver disease	Moderate/Severe	READ	J622.	Hepatic coma
Liver disease	Moderate/Severe	READ	J623.	Portal hypertension
Liver disease	Moderate/Severe	READ	J624.	Hepatorenal syndrome
Liver disease	Moderate/Severe	READ	J625.	[X] Hepatic failure
Liver disease	Moderate/Severe	READ	J630.	Chronic passive liver congestion
Liver disease	Moderate/Severe	READ	J6350	Toxic liver disease with cholestasis
Liver disease	Moderate/Severe	READ	J6351	Toxic liver disease with hepatic necrosis
Liver disease	Moderate/Severe	READ	J6352	Toxic liver disease with acute hepatitis
Liver disease	Moderate/Severe	READ	J6357	Acute hepatic failure due to drugs
Liver disease	Moderate/Severe	READ	J637.	Hepatic veno-occlusive disease
Liver disease	Moderate/Severe	READ	J63B.	Autoimmune hepatitis
Liver disease	Moderate/Severe	READ	J63y1	Nonspecific reactive hepatitis
Liver disease	Moderate/Severe	SNOMED CT	28670008	Esophageal varices (disorder)
Liver disease	Moderate/Severe	SNOMED CT	28670008	Esophageal varices (disorder)
Liver disease	Moderate/Severe	SNOMED CT	34736002	Chronic passive congestion of liver (disorder)
Liver disease	Moderate/Severe	SNOMED CT	34742003	Portal hypertension (disorder)
Liver disease	Moderate/Severe	SNOMED CT	59927004	Hepatic failure (disorder)
Liver disease	Moderate/Severe	SNOMED CT	65617004	Veno-occlusive disease of the liver (disorder)
Liver disease	Moderate/Severe	SNOMED CT	85514005	Phlebitis of portal vein (disorder)
Liver disease	Moderate/Severe	SNOMED CT	91109007	Gastric varices (disorder)
Liver disease	Moderate/Severe	SNOMED CT	91109007	Gastric varices (disorder)
Liver disease	Moderate/Severe	SNOMED CT	197355005	Toxic liver disease with cholestasis (disorder)
Liver disease	Moderate/Severe	SNOMED CT	197356006	Toxic liver disease with hepatic necrosis (disorder)
Liver disease	Moderate/Severe	SNOMED CT	197358007	Toxic liver disease with acute hepatitis (disorder)
Liver disease	Moderate/Severe	SNOMED CT	235858002	Nonspecific reactive hepatitis (disorder)
Liver disease	Moderate/Severe	SNOMED CT	408335007	Autoimmune hepatitis (disorder)

For peer review only

Liver disease	Moderate/Severe	CTV3	G857.	Gastric varices
Liver disease	Moderate/Severe	CTV3	J623.	Portal hypertension
Liver disease	Moderate/Severe	CTV3	J62y.	(Hepat failure (& [NOS]) or (oth sequelae chronic liver dis)
Liver disease	Moderate/Severe	CTV3	J630.	Chronic passive congestion of liver
Liver disease	Moderate/Severe	CTV3	J6350	Toxic liver disease with cholestasis
Liver disease	Moderate/Severe	CTV3	J6351	Toxic liver disease with hepatic necrosis
Liver disease	Moderate/Severe	CTV3	J6352	Toxic liver disease with acute hepatitis
Liver disease	Moderate/Severe	CTV3	X2063	Oesophageal varices
Liver disease	Moderate/Severe	CTV3	X306U	Nonspecific reactive hepatitis
Liver disease	Moderate/Severe	CTV3	X306y	Hepatic veno-occlusive disease
Liver disease	Moderate/Severe	CTV3	X3073	Alcoholic hepatic failure
Liver disease	Moderate/Severe	CTV3	X3076	Hepatic failure
Liver disease	Moderate/Severe	CTV3	X307J	Autoimmune liver disease
Liver disease	Moderate/Severe	CTV3	Xa8Df	Yellow atrophy of the liver
Liver disease	Moderate/Severe	CTV3	XE0dB	(Acute/subacute necrosis of liver) or (acute liver failure)

Any incomplete ICD-10 code (less than four characters) or READ v2 code (less than five characters) implies inclusion of all codes starting with the prefix code. Any CTV3 or SNOMED CT code implies inclusion of all children codes, excluding those in the Exclusion column.

For peer review only

Supplemental Table 2 Codelist for COVID-19 diagnosis

Group	Terminology system	Code	Code description
Confirmed	ICD-10	U071	COVID-19, virus identified
Confirmed	SNOMED CT	1240751000000100	Disease caused by 2019 novel coronavirus (disorder)
Confirmed	SNOMED CT	1240381000000105	2019 novel coronavirus (organism)
Confirmed	SNOMED CT	1240581000000104	2019 novel coronavirus detected (finding)
Suspected	ICD-10	U072	COVID-19, virus not identified
Suspected	SNOMED CT	1240761000000102	Suspected coronavirus disease 19 caused by severe acute respiratory syndrome coronavirus 2 (situation)
Negative	SNOMED CT	1240591000000102	2019 novel coronavirus not detected (finding)

Any SNOMED CT code implies inclusion of all children codes, excluding those in the Exclusion column.

For peer review only

Supplemental Table 3 Differences in demographic, comorbidity, lifestyle, and medication use characteristics between COVID-19 infected and non-COVID-19 groups, stratified by mortality status.

	non-COVID-19 (15214)		COVID-19 (N=217)	
	Survivor (N=14845)	Deceased (N=369)	Survivor (N=164)	Deceased (N=53)
Demographics				
**Gender**				
Female	8406 (98.1%)	164 (1.9%)	82 (84.5%)	15 (15.5%)
Male	6439 (96.9%)	205 (3.1%)	82 (68.3%)	38 (31.7%)
**Ethnic origin**				
White	6712 (97.1%)	202 (2.9%)	74 (78.7%)	20 (21.3%)
South Asian	4319 (98.6%)	62 (1.4%)	48 (72.7%)	18 (27.3%)
Black	1591 (97.3%)	44 (2.7%)	22 (61.1%)	14 (38.9%)
Other	1814 (97.8%)	41 (2.2%)	18 (94.7%)	1 (5.3%)
Unknown	409 (95.3%)	20 (4.7%)	2 (100.0%)	0 (0.0%)
**Age group**				
18-40	2796 (99.8%)	7 (0.2%)	21 (95.5%)	1 (4.5%)
41-50	2690 (99.1%)	24 (0.9%)	23 (92.0%)	2 (8.0%)
51-60	3362 (98.7%)	45 (1.3%)	31 (93.9%)	2 (6.1%)
61-70	2885 (97.6%)	72 (2.4%)	33 (80.5%)	8 (19.5%)
71-80	1889 (95.4%)	91 (4.6%)	25 (61.0%)	16 (39.0%)
80+	1223 (90.4%)	130 (9.6%)	31 (56.4%)	24 (43.6%)
**HPB cancer**				
No	14484 (98.0%)	295 (2.0%)	161 (75.9%)	51 (24.1%)
Yes	361 (83.0%)	74 (17.0%)	3 (60.0%)	2 (40.0%)
**Pancreatic disease**				
No	12040 (98.2%)	224 (1.8%)	129 (79.6%)	33 (20.4%)
Acute	1194 (98.6%)	17 (1.4%)	14 (73.7%)	5 (26.3%)
Chronic	1250 (95.9%)	54 (4.1%)	18 (58.1%)	13 (41.9%)
**Biliary disease**				
No	7448 (98.1%)	141 (1.9%)	99 (79.2%)	26 (20.8%)
Acute	717 (97.2%)	21 (2.8%)	5 (50.0%)	5 (50.0%)
Chronic	6319 (97.9%)	133 (2.1%)	57 (74.0%)	20 (26.0%)
**Liver disease**				
No	6642 (98.0%)	139 (2.0%)	54 (66.7%)	27 (33.3%)
Mild	6867 (98.3%)	118 (1.7%)	88 (82.2%)	19 (17.8%)
Moderate/Severe	975 (96.2%)	38 (3.8%)	19 (79.2%)	5 (20.8%)
Comorbidities				
**Diabetes**				
No	9186 (98.1%)	174 (1.9%)	65 (85.5%)	11 (14.5%)
Yes	5659 (96.7%)	195 (3.3%)	99 (70.2%)	42 (29.8%)
**Hypertension**				
No	5415 (99.3%)	40 (0.7%)	32 (97.0%)	1 (3.0%)
Yes	9430 (96.6%)	329 (3.4%)	132 (71.7%)	52 (28.3%)
**High cholesterol**				
No	6850 (98.0%)	137 (2.0%)	58 (82.9%)	12 (17.1%)
Yes	7995 (97.2%)	232 (2.8%)	106 (72.1%)	41 (27.9%)

For peer review only

**Cardiovascular**				
No	10790 (98.7%)	141 (1.3%)	80 (88.9%)	10 (11.1%)
Yes	4055 (94.7%)	228 (5.3%)	84 (66.1%)	43 (33.9%)
**Renal**				
No	11905 (98.2%)	215 (1.8%)	97 (86.6%)	15 (13.4%)
Yes	2940 (95.0%)	154 (5.0%)	67 (63.8%)	38 (36.2%)
**Respiratory**				
No	10416 (97.9%)	224 (2.1%)	84 (76.4%)	26 (23.6%)
Yes	4429 (96.8%)	145 (3.2%)	80 (74.8%)	27 (25.2%)
**Number of comorbidities**				
None	2397 (99.5%)	13 (0.5%)	8 (100.0%)	0 (0.0%)
1	2885 (98.7%)	39 (1.3%)	13 (100.0%)	0 (0.0%)
2	2992 (98.5%)	47 (1.5%)	26 (96.3%)	1 (3.7%)
3 or more	6571 (96.1%)	270 (3.9%)	117 (69.2%)	52 (30.8%)
Lifestyle factors				
**Smoker**				
Not available	422 (96.8%)	14 (3.2%)	2 (100.0%)	0 (0.0%)
Never	6301 (98.1%)	124 (1.9%)	67 (82.7%)	14 (17.3%)
Past	4960 (97.1%)	150 (2.9%)	72 (66.1%)	37 (33.9%)
Current	3162 (97.5%)	81 (2.5%)	23 (92.0%)	2 (8.0%)
**Drinker**				
Not available	2451 (97.8%)	54 (2.2%)	21 (77.8%)	6 (22.2%)
Never	3772 (97.8%)	85 (2.2%)	44 (78.6%)	12 (21.4%)
Past	2077 (96.8%)	68 (3.2%)	32 (72.7%)	12 (27.3%)
Current	6545 (97.6%)	162 (2.4%)	67 (74.4%)	23 (25.6%)
**Substance user**				
Not available	7537 (98.1%)	149 (1.9%)	77 (80.2%)	19 (19.8%)
Never	3565 (98.9%)	41 (1.1%)	24 (85.7%)	4 (14.3%)
Past	380 (94.3%)	23 (5.7%)	10 (83.3%)	2 (16.7%)
Current	3363 (95.6%)	156 (4.4%)	53 (65.4%)	28 (34.6%)
**Obese**				
Not available	394 (97.0%)	12 (3.0%)	0 (0.0%)	1 (100.0%)
Never	6556 (97.6%)	159 (2.4%)	65 (78.3%)	18 (21.7%)
Past	2101 (95.5%)	98 (4.5%)	33 (68.8%)	15 (31.2%)
Current	5794 (98.3%)	100 (1.7%)	66 (77.6%)	19 (22.4%)
Prescription medication use				
**ACE inhibitor**				
Non-user	11751 (79.2%)	273 (74.0%)	122 (74.4%)	33 (62.3%)
Past user	487 (3.3%)	31 (8.4%)	20 (12.2%)	8 (15.1%)
Current user	2607 (17.6%)	65 (17.6%)	22 (13.4%)	12 (22.6%)
**Angiotensin receptor blocker**				
Non-user	13204 (88.9%)	326 (88.3%)	137 (83.5%)	43 (81.1%)
Past user	207 (1.4%)	20 (5.4%)	3 (1.8%)	2 (3.8%)
Current user	1434 (9.7%)	23 (6.2%)	24 (14.6%)	8 (15.1%)
**Aldosterone antagonist**				
Non-user	14316 (96.4%)	335 (90.8%)	150 (91.5%)	47 (88.7%)
Past user	130 (0.9%)	7 (1.9%)	6 (3.7%)	3 (5.7%)
Current user	399 (2.7%)	27 (7.3%)	8 (4.9%)	3 (5.7%)

For peer review only

**β-blocker**				
Non-user	11930 (80.4%)	231 (62.6%)	106 (64.6%)	32 (60.4%)
Past user	384 (2.6%)	26 (7.0%)	9 (5.5%)	3 (5.7%)
Current user	2531 (17.0%)	112 (30.4%)	49 (29.9%)	18 (34.0%)
**Calcium channel blocker**				
Non-user	11456 (77.2%)	258 (69.9%)	116 (70.7%)	35 (66.0%)
Past user	543 (3.7%)	38 (10.3%)	14 (8.5%)	2 (3.8%)
Current user	2846 (19.2%)	73 (19.8%)	34 (20.7%)	16 (30.2%)
**α-agonist**				
Non-user	14766 (99.5%)	365 (98.9%)	163 (99.4%)	53 (100.0%)
Past user	20 (0.1%)	3 (0.8%)	0 (0.0%)	0 (0.0%)
Current user	59 (0.4%)	1 (0.3%)	1 (0.6%)	0 (0.0%)
**Thiazide**				
Non-user	14763 (99.4%)	368 (99.7%)	163 (99.4%)	53 (100.0%)
Past user	32 (0.2%)	0 (0.0%)	0 (0.0%)	0 (0.0%)
Current user	50 (0.3%)	1 (0.3%)	1 (0.6%)	0 (0.0%)
**Antiplatelet**				
Non-user	12268 (82.6%)	244 (66.1%)	112 (68.3%)	28 (52.8%)
Past user	415 (2.8%)	31 (8.4%)	8 (4.9%)	2 (3.8%)
Current user	2162 (14.6%)	94 (25.5%)	44 (26.8%)	23 (43.4%)
**Antiarrhythmic**				
Non-user	14109 (95.0%)	331 (89.7%)	147 (89.6%)	43 (81.1%)
Past user	148 (1.0%)	8 (2.2%)	6 (3.7%)	1 (1.9%)
Current user	588 (4.0%)	30 (8.1%)	11 (6.7%)	9 (17.0%)
**Anticoagulant**				
Non-user	14272 (96.1%)	341 (92.4%)	152 (92.7%)	47 (88.7%)
Past user	133 (0.9%)	11 (3.0%)	4 (2.4%)	1 (1.9%)
Current user	440 (3.0%)	17 (4.6%)	8 (4.9%)	5 (9.4%)
**Glucocorticoid**				
Non-user	10644 (71.7%)	234 (63.4%)	95 (57.9%)	19 (35.8%)
Past user	1230 (8.3%)	48 (13.0%)	16 (9.8%)	6 (11.3%)
Current user	2971 (20.0%)	87 (23.6%)	53 (32.3%)	28 (52.8%)
**β2-agonist**				
Non-user	13132 (88.5%)	311 (84.3%)	131 (79.9%)	33 (62.3%)
Past user	276 (1.9%)	10 (2.7%)	6 (3.7%)	2 (3.8%)
Current user	1437 (9.7%)	48 (13.0%)	27 (16.5%)	18 (34.0%)
**Muscarinic antagonist**				
Non-user	13235 (89.2%)	296 (80.2%)	129 (78.7%)	39 (73.6%)
Past user	291 (2.0%)	9 (2.4%)	5 (3.0%)	4 (7.5%)
Current user	1319 (8.9%)	64 (17.3%)	30 (18.3%)	10 (18.9%)
**NSAID**				
Non-user	13368 (90.1%)	335 (90.8%)	146 (89.0%)	43 (81.1%)
Past user	743 (5.0%)	13 (3.5%)	8 (4.9%)	2 (3.8%)
Current user	734 (4.9%)	21 (5.7%)	10 (6.1%)	8 (15.1%)
**Vitamin D**				
Non-user	12295 (82.8%)	247 (66.9%)	109 (66.5%)	26 (49.1%)
Past user	543 (3.7%)	30 (8.1%)	12 (7.3%)	6 (11.3%)
Current user	2007 (13.5%)	92 (24.9%)	43 (26.2%)	21 (39.6%)

For peer review only

**Proton pump inhibitor**				
Non-user	8162 (55.0%)	170 (46.1%)	62 (37.8%)	18 (34.0%)
Past user	1137 (7.7%)	30 (8.1%)	11 (6.7%)	3 (5.7%)
Current user	5546 (37.4%)	169 (45.8%)	91 (55.5%)	32 (60.4%)
**Statin**				
Non-user	8945 (60.3%)	183 (49.6%)	72 (43.9%)	11 (20.8%)
Past user	547 (3.7%)	45 (12.2%)	12 (7.3%)	3 (5.7%)
Current user	5353 (36.1%)	141 (38.2%)	80 (48.8%)	39 (73.6%)
**Immunosuppressant**				
Non-user	14368 (96.8%)	354 (95.9%)	156 (95.1%)	48 (90.6%)
Past user	196 (1.3%)	8 (2.2%)	3 (1.8%)	2 (3.8%)
Current user	281 (1.9%)	7 (1.9%)	5 (3.0%)	3 (5.7%)

Values are n (%), unless otherwise specified. *Percentages are calculated row-wise across each strata of the exposure variable, as opposed to column-wise across the outcome variable, to demonstrate the differences in mortality due to COVID-19.

For peer review only

Supplemental Table 4 Odds ratio estimates of COVID-19 for HPB patients with specific demographic, comorbidity, lifestyle and medication use characteristics.

	Crude OR (95% CI)	P value	Adjusted OR (95% CI)	P value	Adjusted OR (+all comorbidity) (95% CI)	P value
**Demographics**						
Gender (ref=Female)						
Male	1.49 (1.14 to 1.93)	0.003	1.56 (1.2 to 2.04)	0.002	1.39 (1.06 to 1.82)	0.04
Ethnicity (ref=White)						
South Asian	1.11 (0.81 to 1.51)	0.526	1.35 (0.98 to 1.85)	0.102	1.07 (0.77 to 1.48)	0.742
Black	1.74 (1.2 to 2.53)	0.009	2.04 (1.39 to 2.95)	<0.001	1.82 (1.23 to 2.66)	0.006
Other	0.73 (0.44 to 1.2)	0.265	0.87 (0.52 to 1.4)	0.654	0.88 (0.52 to 1.43)	0.716
Age group (ref=18-40)						
41-50	1.17 (0.66 to 2.05)	0.59	1.12 (0.64 to 1.98)	0.694	0.83 (0.47 to 1.49)	0.644
51-60	1.25 (0.74 to 2.12)	0.486	1.19 (0.7 to 2.05)	0.632	0.64 (0.36 to 1.13)	0.219
61-70	1.73 (1.04 to 2.89)	0.053	1.71 (1.03 to 2.9)	0.075	0.66 (0.38 to 1.19)	0.273
71-80	2.65 (1.59 to 4.41)	<0.001	2.71 (1.63 to 4.6)	<0.001	0.81 (0.45 to 1.48)	0.633
80+	5.13 (3.15 to 8.37)	<0.001	5.21 (3.23 to 8.7)	<0.001	1.31 (0.73 to 2.39)	0.53
**HPB disease (ref=No)**						
Cancer						
Yes	0.93 (0.41 to 2.1)	0.855	1.11 (0.46 to 2.69)	0.822	1.04 (0.43 to 2.54)	0.931
Pancreatic disease						
Acute	1.13 (0.7 to 1.83)	0.815	1.35 (0.82 to 2.22)	0.323	1.24 (0.75 to 2.04)	0.582
Chronic	1.72 (1.16 to 2.53)	0.013	1.89 (1.25 to 2.85)	0.007	1.57 (1.04 to 2.38)	0.084
Liver disease						
Mild	1.34 (1 to 1.78)	0.062	1.52 (1.07 to 2.15)	0.039	1.32 (0.93 to 1.88)	0.237
Moderate/Severe	2.04 (1.3 to 3.21)	0.004	2.2 (1.35 to 3.59)	0.006	1.84 (1.12 to 3.02)	0.046
Biliary disease						
Acute	0.89 (0.48 to 1.66)	0.715	1.1 (0.58 to 2.09)	0.822	1.04 (0.55 to 1.98)	0.931
Chronic	0.76 (0.57 to 1)	0.108	1.05 (0.75 to 1.46)	0.822	0.96 (0.69 to 1.35)	0.925
**Comorbidities (ref=No)**						
Diabetes	3.03 (2.31 to 4.02)	<0.001	2.47 (1.85 to 3.32)	<0.001	1.78 (1.3 to 2.42)	<0.001
Hypertension	3.27 (2.29 to 4.82)	<0.001	2.35 (1.59 to 3.48)	<0.001	1.38 (0.9 to 2.1)	0.226
Cholesterol	1.89 (1.43 to 2.53)	<0.001	1.47 (1.09 to 1.98)	0.019	0.94 (0.69 to 1.29)	0.71
Cardiovascular	3.52 (2.7 to 4.6)	<0.001	2.77 (2.07 to 3.71)	<0.001	1.84 (1.35 to 2.5)	<0.001
Renal	3.71 (2.85 to 4.84)	<0.001	2.93 (2.2 to 3.89)	<0.001	2.13 (1.59 to 2.86)	<0.001
Respiratory	2.25 (1.72 to 2.92)	<0.001	2.06 (1.58 to 2.69)	<0.001	1.77 (1.35 to 2.33)	<0.001
Number of comorbidities	1.66 (1.53 to 1.81)	<0.001	1.62 (1.46 to 1.79)	<0.001		
**Lifestyle factors (ref=Never)**						
Smoker						
Past	1.71 (1.28 to 2.27)	<0.001	1.46 (1.08 to 1.98)	0.03	1.18 (0.86 to 1.61)	0.476
Current	0.61 (0.39 to 0.95)	0.04	0.65 (0.41 to 1.04)	0.116	0.58 (0.36 to 0.93)	0.053

For peer review only

Drinker						
Past	1.46 (0.99 to 2.15)	0.115	1.26 (0.85 to 1.88)	0.283	1.11 (0.74 to 1.66)	0.756
Current	0.92 (0.66 to 1.28)	0.63	0.81 (0.56 to 1.16)	0.283	0.87 (0.61 to 1.24)	0.639
Substance user						
Past	4.01 (2.07 to 7.78)	<0.001	3.43 (1.74 to 6.75)	<0.001	2.18 (1.1 to 4.34)	0.053
Current	3 (1.97 to 4.59)	<0.001	2.63 (1.68 to 4.1)	<0.001	1.96 (1.25 to 3.07)	0.008
Obese						
Past	1.83 (1.29 to 2.6)	0.001	1.61 (1.13 to 2.3)	0.016	1.21 (0.84 to 1.74)	0.438
Current	1.19 (0.88 to 1.61)	0.248	1.26 (0.93 to 1.72)	0.187	1.01 (0.74 to 1.38)	0.973
**Prescription medication use (ref=Non-user)**						
ACE inhibitor						
Past user	4.04 (2.68 to 6.09)	<0.001	3 (1.97 to 4.57)	<0.001	1.88 (1.22 to 2.89)	0.007
Current user	1.03 (0.72 to 1.48)	0.855	0.8 (0.55 to 1.15)	0.25	0.56 (0.39 to 0.82)	0.005
Angiotensin receptor blocker						
Past user	1.59 (0.65 to 3.89)	0.314	1.18 (0.48 to 2.91)	0.722	0.67 (0.27 to 1.66)	0.524
Current user	1.63 (1.12 to 2.37)	0.016	1.27 (0.86 to 1.86)	0.293	0.91 (0.62 to 1.34)	0.681
Aldosterone agonist						
Past user	4.69 (2.36 to 9.35)	<0.001	3.74 (1.86 to 7.5)	<0.001	2.24 (1.1 to 4.56)	0.056
Current user	2.01 (1.12 to 3.63)	0.02	1.61 (0.89 to 2.91)	0.151	1 (0.54 to 1.82)	0.986
β-blocker						
Past user	2.45 (1.35 to 4.46)	0.003	1.95 (1.07 to 3.56)	0.045	1.24 (0.67 to 2.31)	0.666
Current user	2.19 (1.64 to 2.93)	<0.001	1.62 (1.19 to 2.19)	0.004	0.93 (0.67 to 1.29)	0.711
Calcium channel blocker						
Past user	2.17 (1.31 to 3.6)	0.004	1.5 (0.89 to 2.51)	0.162	0.99 (0.59 to 1.68)	0.982
Current user	1.3 (0.94 to 1.78)	0.111	0.9 (0.64 to 1.25)	0.533	0.68 (0.49 to 0.96)	0.056
α-agonist						
Past user	0 (0 to 2.41e+253)	0.97	0 (0 to 2.56e+248)	0.969	0 (0 to 1.73e+242)	0.968
Current user	1.12 (0.16 to 8.12)	0.97	0.77 (0.1 to 5.61)	0.895	0.6 (0.08 to 4.43)	0.771
Thiazide						
Past user	0 (0 to 1.16e+214)	0.965	0 (0 to 8.76e+208)	0.971	0 (0 to 9.71e+205)	0.962
Current user	1.32 (0.18 to 9.58)	0.965	1.04 (0.14 to 7.59)	0.971	0.83 (0.11 to 6.17)	0.919
Antiplatelet						
Past user	1.91 (1 to 3.65)	0.05	1.41 (0.73 to 2.71)	0.345	0.79 (0.4 to 1.54)	0.661
Current user	2.6 (1.95 to 3.48)	<0.001	1.84 (1.35 to 2.51)	<0.001	0.95 (0.67 to 1.33)	0.754
Antiarrhythmic						
Past user	3.26 (1.51 to 7.03)	0.003	2.42 (1.11 to 5.27)	0.039	1.74 (0.79 to 3.83)	0.282
Current user	2.35 (1.47 to 3.75)	<0.001	1.85 (1.15 to 2.97)	0.021	1.19 (0.73 to 1.93)	0.602
Anticoagulant						
Past user	2.44 (0.99 to 6.01)	0.053	1.96 (0.79 to 4.86)	0.165	1.39 (0.55 to 3.48)	0.661
Current user	2 (1.13 to 3.53)	0.025	1.54 (0.86 to 2.73)	0.165	1 (0.56 to 1.8)	0.989
Glucocorticoid						
Past user	1.53 (0.97 to 2.43)	0.067	1.39 (0.88 to 2.2)	0.242	1.09 (0.68 to 1.74)	0.774
Current user	2.39 (1.8 to 3.17)	<0.001	2.07 (1.55 to 2.77)	<0.001	1.38 (1 to 1.92)	0.101

For peer review only

β2-agonist						
Past user	2.19 (1.07 to 4.48)	0.033	2.08 (1.01 to 4.27)	0.071	1.36 (0.64 to 2.87)	0.562
Current user	2.42 (1.74 to 3.37)	<0.001	2.07 (1.48 to 2.9)	<0.001	1.32 (0.89 to 1.96)	0.281
Muscarinic antagonist						
Past user	2.58 (1.35 to 4.92)	0.004	2.2 (1.14 to 4.22)	0.027	1.67 (0.86 to 3.24)	0.241
Current user	2.29 (1.62 to 3.24)	<0.001	1.85 (1.3 to 2.64)	0.001	1.25 (0.86 to 1.81)	0.365
NSAID						
Past user	0.92 (0.48 to 1.74)	0.787	0.81 (0.42 to 1.54)	0.517	0.79 (0.42 to 1.51)	0.598
Current user	1.65 (1.01 to 2.69)	0.066	1.56 (0.95 to 2.55)	0.139	1.57 (0.96 to 2.59)	0.14
Vitamin D						
Past user	2.83 (1.72 to 4.66)	<0.001	2.49 (1.5 to 4.13)	<0.001	1.84 (1.1 to 3.06)	0.033
Current user	2.97 (2.21 to 3.97)	<0.001	2.5 (1.84 to 3.4)	<0.001	1.79 (1.31 to 2.45)	<0.001
Proton pump inhibitor						
Past user	1.26 (0.72 to 2.19)	0.412	1.15 (0.66 to 2.01)	0.619	0.95 (0.54 to 1.66)	0.858
Current user	2.16 (1.64 to 2.85)	<0.001	1.78 (1.34 to 2.38)	<0.001	1.18 (0.87 to 1.59)	0.447
Statin						
Past user	2.87 (1.67 to 4.92)	<0.001	2.1 (1.21 to 3.65)	0.013	1.24 (0.69 to 2.21)	0.643
Current user	2.4 (1.82 to 3.16)	<0.001	1.77 (1.3 to 2.4)	<0.001	0.94 (0.66 to 1.33)	0.758
Immunosuppressant						
Past user	1.69 (0.69 to 4.16)	0.25	1.42 (0.58 to 3.51)	0.448	1.28 (0.52 to 3.18)	0.686
Current user	1.92 (0.94 to 3.93)	0.111	1.7 (0.83 to 3.5)	0.191	1.45 (0.7 to 2.99)	0.481

	**Adjusted OR (+Diabetes) (95% CI)**	**P value**	**Adjusted OR (+Hypertension) (95% CI)**	**P value**	**Adjusted OR (+Cholesterol) (95% CI)**	**P value**
**Demographics**						
Gender (ref=Female)						
Male	1.48 (1.14 to 1.94)	0.009	1.52 (1.16 to 1.99)	0.005	1.55 (1.19 to 2.02)	0.004
Ethnicity (ref=White)						
South Asian	1.04 (0.75 to 1.44)	0.868	1.3 (0.94 to 1.78)	0.18	1.29 (0.93 to 1.77)	0.209
Black	1.76 (1.19 to 2.56)	0.009	1.95 (1.32 to 2.82)	0.002	2.06 (1.4 to 2.99)	<0.001
Other	0.78 (0.46 to 1.26)	0.503	0.88 (0.52 to 1.42)	0.75	0.87 (0.51 to 1.4)	0.705
Age group (ref=18-40)						
41-50	0.95 (0.54 to 1.69)	0.868	0.97 (0.55 to 1.72)	0.914	1.02 (0.58 to 1.83)	0.933
51-60	0.9 (0.52 to 1.56)	0.83	0.92 (0.54 to 1.61)	0.835	1.04 (0.61 to 1.82)	0.933
61-70	1.16 (0.69 to 1.99)	0.789	1.2 (0.71 to 2.09)	0.664	1.45 (0.86 to 2.5)	0.235
71-80	1.75 (1.03 to 3.03)	0.08	1.79 (1.05 to 3.14)	0.071	2.25 (1.33 to 3.92)	0.008
80+	3.39 (2.06 to 5.76)	<0.001	3.36 (2.01 to 5.8)	<0.001	4.32 (2.61 to 7.39)	<0.001
**HPB disease (ref=No)**						
Cancer						
Yes	1 (0.41 to 2.43)	0.994	1.04 (0.43 to 2.54)	0.926	1.11 (0.46 to 2.71)	0.836

For peer review only

Pancreatic disease						
Acute	1.28 (0.78 to 2.1)	0.453	1.29 (0.78 to 2.12)	0.429	1.32 (0.8 to 2.17)	0.369
Chronic	1.69 (1.11 to 2.56)	0.033	1.77 (1.17 to 2.66)	0.017	1.84 (1.22 to 2.78)	0.011
Liver disease						
Mild	1.4 (0.99 to 1.98)	0.112	1.44 (1.01 to 2.04)	0.08	1.47 (1.04 to 2.09)	0.058
Moderate/Severe	2.04 (1.25 to 3.33)	0.013	2.09 (1.28 to 3.42)	0.01	2.16 (1.33 to 3.53)	0.008
Biliary disease						
Acute	1.08 (0.57 to 2.05)	0.941	1.07 (0.56 to 2.03)	0.926	1.07 (0.57 to 2.04)	0.836
Chronic	1.03 (0.74 to 1.44)	0.941	1.02 (0.73 to 1.42)	0.926	1.04 (0.74 to 1.45)	0.836
**Comorbidities (ref=No)**						
Diabetes	2.47 (1.85 to 3.32)	<0.001	2.19 (1.62 to 2.96)	<0.001	2.37 (1.76 to 3.2)	<0.001
Hypertension	1.87 (1.25 to 2.8)	0.005	2.35 (1.59 to 3.48)	<0.001	2.21 (1.48 to 3.29)	<0.001
Cholesterol	1.21 (0.9 to 1.64)	0.275	1.29 (0.96 to 1.75)	0.142	1.47 (1.09 to 1.98)	0.019
Cardiovascular	2.38 (1.77 to 3.2)	<0.001	2.45 (1.82 to 3.29)	<0.001	2.66 (1.98 to 3.58)	<0.001
Renal	2.55 (1.91 to 3.4)	<0.001	2.64 (1.98 to 3.52)	<0.001	2.83 (2.12 to 3.77)	<0.001
Respiratory	1.95 (1.49 to 2.54)	<0.001	1.98 (1.51 to 2.58)	<0.001	2.02 (1.54 to 2.64)	<0.001
**Lifestyle factors (ref=Never)**						
Smoker						
Past	1.39 (1.02 to 1.89)	0.064	1.42 (1.05 to 1.93)	0.044	1.42 (1.05 to 1.93)	0.052
Current	0.66 (0.41 to 1.04)	0.118	0.64 (0.4 to 1.02)	0.094	0.65 (0.41 to 1.02)	0.099
Drinker						
Past	1.19 (0.8 to 1.78)	0.446	1.21 (0.81 to 1.81)	0.394	1.23 (0.82 to 1.84)	0.38
Current	0.82 (0.57 to 1.17)	0.388	0.8 (0.56 to 1.14)	0.345	0.8 (0.56 to 1.14)	0.335
Substance user						
Past	3.08 (1.56 to 6.08)	0.002	3.14 (1.6 to 6.19)	0.002	3.29 (1.67 to 6.48)	0.001
Current	2.5 (1.6 to 3.89)	<0.001	2.47 (1.58 to 3.85)	<0.001	2.56 (1.64 to 3.99)	<0.001
Obese						
Past	1.38 (0.96 to 1.97)	0.148	1.47 (1.03 to 2.1)	0.061	1.56 (1.09 to 2.22)	0.033
Current	1.1 (0.81 to 1.5)	0.6	1.16 (0.85 to 1.57)	0.391	1.23 (0.9 to 1.67)	0.233
**Prescription medication use (ref=Non-user)**						
ACE inhibitor						
Past user	2.52 (1.65 to 3.85)	<0.001	2.54 (1.66 to 3.87)	<0.001	2.86 (1.88 to 4.36)	<0.001
Current user	0.64 (0.44 to 0.93)	0.029	0.67 (0.46 to 0.97)	0.047	0.75 (0.52 to 1.09)	0.19
Angiotensin receptor blocker						
Past user	0.98 (0.4 to 2.43)	0.981	1.04 (0.42 to 2.57)	0.928	1.13 (0.46 to 2.78)	0.799
Current user	1.1 (0.75 to 1.62)	0.784	1.1 (0.75 to 1.61)	0.707	1.2 (0.82 to 1.77)	0.427
Aldosterone agonist						
Past user	3.47 (1.73 to 6.99)	0.001	3.5 (1.74 to 7.02)	0.001	3.62 (1.8 to 7.27)	<0.001
Current user	1.49 (0.82 to 2.71)	0.232	1.49 (0.82 to 2.69)	0.217	1.56 (0.86 to 2.83)	0.199
β-blocker						
Past user	1.79 (0.98 to 3.28)	0.083	1.78 (0.97 to 3.25)	0.087	1.87 (1.02 to 3.42)	0.06
Current user	1.4 (1.04 to 1.9)	0.048	1.44 (1.06 to 1.95)	0.03	1.54 (1.14 to 2.09)	0.013

For peer review only

Calcium channel blocker						
Past user	1.33 (0.79 to 2.24)	0.309	1.24 (0.74 to 2.09)	0.459	1.42 (0.84 to 2.38)	0.236
Current user	0.79 (0.57 to 1.11)	0.245	0.73 (0.52 to 1.03)	0.119	0.86 (0.62 to 1.21)	0.437
α-agonist						
Past user	0 (0 to 1.19e+246)	0.978	0 (0 to 2.77e+247)	0.969	0 (0 to 2.45e+248)	0.969
Current user	0.68 (0.09 to 4.93)	0.874	0.72 (0.1 to 5.26)	0.831	0.73 (0.1 to 5.32)	0.838
Thiazide						
Past user	0 (0 to 3.39e+207)	0.986	0 (0 to 8.99e+209)	0.963	0 (0 to 1.01e+209)	0.989
Current user	0.91 (0.12 to 6.66)	0.986	0.92 (0.13 to 6.76)	0.963	1.01 (0.14 to 7.43)	0.989
Antiplatelet						
Past user	1.2 (0.62 to 2.32)	0.655	1.27 (0.66 to 2.44)	0.48	1.34 (0.7 to 2.59)	0.423
Current user	1.54 (1.13 to 2.11)	0.013	1.64 (1.2 to 2.23)	0.004	1.73 (1.27 to 2.38)	0.002
Antiarrhythmic						
Past user	2.4 (1.1 to 5.24)	0.04	2.24 (1.03 to 4.87)	0.06	2.32 (1.07 to 5.07)	0.048
Current user	1.71 (1.06 to 2.75)	0.04	1.72 (1.07 to 2.77)	0.041	1.78 (1.11 to 2.87)	0.031
Anticoagulant						
Past user	1.91 (0.77 to 4.75)	0.233	1.83 (0.74 to 4.54)	0.237	1.89 (0.76 to 4.69)	0.204
Current user	1.44 (0.81 to 2.57)	0.269	1.44 (0.81 to 2.57)	0.237	1.48 (0.83 to 2.63)	0.204
Glucocorticoid						
Past user	1.29 (0.81 to 2.05)	0.313	1.33 (0.84 to 2.11)	0.304	1.36 (0.86 to 2.16)	0.276
Current user	1.89 (1.41 to 2.52)	<0.001	1.96 (1.46 to 2.61)	<0.001	2 (1.5 to 2.68)	<0.001
β2-agonist						
Past user	1.93 (0.94 to 3.98)	0.107	1.94 (0.94 to 4)	0.103	2.04 (0.99 to 4.2)	0.076
Current user	1.92 (1.37 to 2.69)	<0.001	1.97 (1.41 to 2.76)	<0.001	2 (1.43 to 2.81)	<0.001
Muscarinic antagonist						
Past user	2.05 (1.06 to 3.94)	0.046	2.11 (1.1 to 4.06)	0.036	2.13 (1.11 to 4.09)	0.034
Current user	1.74 (1.22 to 2.47)	0.004	1.75 (1.23 to 2.48)	0.004	1.8 (1.27 to 2.57)	0.003
NSAID						
Past user	0.81 (0.42 to 1.53)	0.569	0.78 (0.41 to 1.49)	0.506	0.8 (0.42 to 1.52)	0.492
Current user	1.53 (0.93 to 2.5)	0.154	1.51 (0.92 to 2.46)	0.173	1.53 (0.94 to 2.51)	0.148
Vitamin D						
Past user	2.27 (1.37 to 3.76)	0.003	2.35 (1.42 to 3.9)	0.002	2.4 (1.45 to 3.99)	0.001
Current user	2.29 (1.68 to 3.11)	<0.001	2.34 (1.72 to 3.18)	<0.001	2.42 (1.78 to 3.29)	<0.001
Proton pump inhibitor						
Past user	1.1 (0.63 to 1.91)	0.754	1.11 (0.64 to 1.93)	0.715	1.11 (0.64 to 1.94)	0.704
Current user	1.55 (1.16 to 2.07)	0.007	1.62 (1.21 to 2.17)	0.002	1.69 (1.26 to 2.27)	0.001
Statin						
Past user	1.62 (0.93 to 2.85)	0.152	1.84 (1.06 to 3.21)	0.045	1.95 (1.1 to 3.45)	0.036
Current user	1.3 (0.94 to 1.8)	0.16	1.49 (1.09 to 2.05)	0.02	1.64 (1.17 to 2.29)	0.01
Immunosuppressant						
Past user	1.4 (0.57 to 3.46)	0.519	1.36 (0.55 to 3.35)	0.507	1.38 (0.56 to 3.4)	0.489
Current user	1.59 (0.77 to 3.27)	0.297	1.65 (0.8 to 3.39)	0.242	1.69 (0.82 to 3.47)	0.231

For peer review only

	Adjusted OR (+Cardiovascular) (95% CI)	P value	Adjusted OR (+Renal) (95% CI)	P value	Adjusted OR (+Respiratory) (95% CI)	P value
**Demographics**						
Gender (ref=Female)						
Male	1.39 (1.06 to 1.82)	0.042	1.53 (1.17 to 2)	0.004	1.57 (1.21 to 2.06)	0.002
Ethnicity (ref=White)						
South Asian	1.3 (0.94 to 1.78)	0.186	1.28 (0.93 to 1.76)	0.22	1.37 (1 to 1.88)	0.087
Black	2.01 (1.36 to 2.91)	<0.001	1.93 (1.31 to 2.8)	0.002	2.17 (1.47 to 3.15)	<0.001
Other	0.89 (0.53 to 1.44)	0.792	0.86 (0.51 to 1.39)	0.675	0.93 (0.55 to 1.5)	0.779
Age group (ref=18-40)						
41-50	1.03 (0.58 to 1.82)	0.925	1.04 (0.59 to 1.84)	0.944	1.11 (0.63 to 1.97)	0.779
51-60	0.96 (0.56 to 1.66)	0.925	1.02 (0.6 to 1.76)	0.944	1.12 (0.66 to 1.94)	0.779
61-70	1.18 (0.7 to 2.05)	0.712	1.29 (0.77 to 2.21)	0.466	1.55 (0.94 to 2.64)	0.141
71-80	1.66 (0.97 to 2.9)	0.139	1.75 (1.03 to 3.04)	0.086	2.37 (1.43 to 4.05)	0.002
80+	2.84 (1.68 to 4.93)	<0.001	2.88 (1.71 to 4.99)	<0.001	4.58 (2.83 to 7.66)	<0.001
**HPB disease (ref=No)**						
Cancer						
Yes	1.13 (0.46 to 2.74)	0.888	1.11 (0.45 to 2.7)	0.902	1.1 (0.45 to 2.68)	0.872
Pancreatic disease						
Acute	1.33 (0.81 to 2.18)	0.356	1.33 (0.81 to 2.18)	0.363	1.33 (0.81 to 2.19)	0.348
Chronic	1.77 (1.17 to 2.67)	0.017	1.78 (1.18 to 2.69)	0.015	1.81 (1.2 to 2.74)	0.011
Liver disease						
Mild	1.46 (1.03 to 2.08)	0.07	1.48 (1.04 to 2.09)	0.054	1.46 (1.03 to 2.07)	0.067
Moderate/Severe	2.08 (1.27 to 3.41)	0.011	2.07 (1.26 to 3.39)	0.012	2.12 (1.3 to 3.46)	0.008
Biliary disease						
Acute	1.07 (0.57 to 2.04)	0.888	1.07 (0.56 to 2.03)	0.902	1.11 (0.59 to 2.11)	0.86
Chronic	1.01 (0.73 to 1.42)	0.935	1.01 (0.72 to 1.4)	0.975	1.03 (0.74 to 1.43)	0.872
**Comorbidities (ref=No)**						
Diabetes	2.1 (1.56 to 2.83)	<0.001	2.12 (1.57 to 2.86)	<0.001	2.35 (1.75 to 3.15)	<0.001
Hypertension	1.83 (1.22 to 2.75)	0.008	1.93 (1.3 to 2.89)	0.004	2.21 (1.49 to 3.27)	<0.001
Cholesterol	1.24 (0.91 to 1.68)	0.22	1.28 (0.95 to 1.74)	0.155	1.39 (1.03 to 1.88)	0.044
Cardiovascular	2.77 (2.07 to 3.71)	<0.001	2.26 (1.67 to 3.05)	<0.001	2.57 (1.92 to 3.45)	<0.001
Renal	2.4 (1.79 to 3.22)	<0.001	2.93 (2.2 to 3.89)	<0.001	2.81 (2.11 to 3.74)	<0.001
Respiratory	1.86 (1.42 to 2.44)	<0.001	1.96 (1.5 to 2.56)	<0.001	2.06 (1.58 to 2.69)	<0.001
**Lifestyle factors (ref=Never)**						
Smoker						
Past	1.34 (0.99 to 1.82)	0.096	1.37 (1.01 to 1.86)	0.08	1.31 (0.96 to 1.78)	0.137
Current	0.62 (0.39 to 0.98)	0.092	0.68 (0.43 to 1.08)	0.158	0.56 (0.36 to 0.9)	0.029
Drinker						
Past	1.23 (0.82 to 1.84)	0.432	1.22 (0.82 to 1.82)	0.405	1.2 (0.8 to 1.8)	0.407
Current	0.86 (0.6 to 1.24)	0.471	0.84 (0.58 to 1.2)	0.405	0.8 (0.56 to 1.14)	0.305

For peer review only

Substance user						
Past	2.74 (1.38 to 5.42)	0.007	2.91 (1.47 to 5.75)	0.004	3.02 (1.53 to 5.96)	0.003
Current	2.21 (1.41 to 3.46)	0.001	2.41 (1.54 to 3.76)	<0.001	2.38 (1.52 to 3.72)	<0.001
Obese						
Past	1.49 (1.05 to 2.13)	0.05	1.46 (1.02 to 2.08)	0.071	1.53 (1.07 to 2.19)	0.034
Current	1.2 (0.88 to 1.63)	0.264	1.21 (0.89 to 1.64)	0.261	1.21 (0.89 to 1.65)	0.264
**Prescription medication use (ref=Non-user)**						
ACE inhibitor						
Past user	2.38 (1.56 to 3.64)	<0.001	2.43 (1.59 to 3.72)	<0.001	2.88 (1.89 to 4.4)	<0.001
Current user	0.67 (0.46 to 0.97)	0.055	0.72 (0.5 to 1.03)	0.105	0.78 (0.54 to 1.13)	0.215
Angiotensin receptor blocker						
Past user	0.93 (0.38 to 2.31)	0.884	0.87 (0.35 to 2.15)	0.758	1.1 (0.45 to 2.73)	0.834
Current user	1.1 (0.75 to 1.62)	0.689	1.09 (0.74 to 1.6)	0.742	1.21 (0.83 to 1.78)	0.409
Aldosterone agonist						
Past user	2.76 (1.37 to 5.56)	0.009	2.9 (1.44 to 5.86)	0.006	3.56 (1.77 to 7.15)	<0.001
Current user	1.17 (0.64 to 2.13)	0.612	1.29 (0.71 to 2.34)	0.455	1.5 (0.82 to 2.72)	0.228
β-blocker						
Past user	1.4 (0.76 to 2.58)	0.353	1.6 (0.87 to 2.94)	0.184	1.99 (1.09 to 3.63)	0.037
Current user	1.09 (0.79 to 1.5)	0.614	1.32 (0.97 to 1.79)	0.132	1.6 (1.18 to 2.16)	0.005
Calcium channel blocker						
Past user	1.24 (0.74 to 2.09)	0.466	1.25 (0.74 to 2.1)	0.452	1.43 (0.85 to 2.41)	0.225
Current user	0.83 (0.6 to 1.16)	0.346	0.8 (0.57 to 1.11)	0.229	0.9 (0.64 to 1.25)	0.579
α-agonist						
Past user	0 (0 to 1.39e+247)	0.969	0 (0 to 4.77e+244)	0.968	0 (0 to 1.01e+246)	0.969
Current user	0.69 (0.09 to 5.08)	0.799	0.66 (0.09 to 4.86)	0.762	0.81 (0.11 to 5.9)	0.926
Thiazide						
Past user	0 (0 to 1.82e+207)	0.962	0 (0 to 4.41e+206)	0.962	0 (0 to 1.47e+209)	0.986
Current user	1.1 (0.15 to 8.06)	0.962	0.9 (0.12 to 6.63)	0.962	1.02 (0.14 to 7.47)	0.986
Antiplatelet						
Past user	0.9 (0.46 to 1.75)	0.751	1.18 (0.61 to 2.27)	0.632	1.34 (0.69 to 2.58)	0.431
Current user	1.11 (0.79 to 1.56)	0.624	1.53 (1.12 to 2.1)	0.015	1.7 (1.24 to 2.32)	0.002
Antiarrhythmic						
Past user	1.87 (0.85 to 4.08)	0.177	2.02 (0.92 to 4.42)	0.13	2.28 (1.04 to 4.98)	0.055
Current user	1.34 (0.83 to 2.18)	0.256	1.5 (0.93 to 2.42)	0.139	1.75 (1.09 to 2.82)	0.036
Anticoagulant						
Past user	1.49 (0.6 to 3.7)	0.494	1.69 (0.68 to 4.2)	0.327	1.9 (0.76 to 4.72)	0.23
Current user	1.15 (0.64 to 2.05)	0.647	1.25 (0.7 to 2.23)	0.498	1.45 (0.81 to 2.58)	0.23
Glucocorticoid						
Past user	1.29 (0.81 to 2.05)	0.317	1.32 (0.83 to 2.1)	0.299	1.21 (0.76 to 1.93)	0.475
Current user	1.88 (1.41 to 2.52)	<0.001	1.93 (1.44 to 2.58)	<0.001	1.57 (1.13 to 2.19)	0.011
β2-agonist						
Past user	1.84 (0.89 to 3.8)	0.141	2 (0.97 to 4.12)	0.089	1.42 (0.68 to 3.01)	0.393
Current user	1.84 (1.31 to 2.59)	0.001	2.02 (1.44 to 2.84)	<0.001	1.38 (0.93 to 2.05)	0.153

For peer review only

Muscarinic antagonist						
Past user	1.95 (1.01 to 3.76)	0.066	2.15 (1.11 to 4.14)	0.032	1.82 (0.94 to 3.52)	0.101
Current user	1.61 (1.13 to 2.3)	0.017	1.74 (1.22 to 2.48)	0.005	1.39 (0.96 to 2.03)	0.101
NSAID						
Past user	0.81 (0.42 to 1.54)	0.567	0.83 (0.43 to 1.58)	0.565	0.77 (0.4 to 1.47)	0.477
Current user	1.55 (0.95 to 2.55)	0.136	1.64 (1 to 2.69)	0.085	1.5 (0.91 to 2.45)	0.156
Vitamin D						
Past user	2.22 (1.34 to 3.69)	0.004	2.15 (1.3 to 3.58)	0.005	2.32 (1.4 to 3.85)	0.002
Current user	2.18 (1.6 to 2.97)	<0.001	2.13 (1.56 to 2.91)	<0.001	2.3 (1.69 to 3.13)	<0.001
Proton pump inhibitor						
Past user	1.08 (0.62 to 1.88)	0.797	1.05 (0.6 to 1.84)	0.855	1.1 (0.63 to 1.92)	0.738
Current user	1.46 (1.09 to 1.97)	0.023	1.58 (1.18 to 2.12)	0.005	1.61 (1.2 to 2.16)	0.002
Statin						
Past user	1.68 (0.96 to 2.95)	0.098	1.75 (1 to 3.06)	0.072	1.99 (1.14 to 3.46)	0.022
Current user	1.35 (0.98 to 1.86)	0.098	1.46 (1.07 to 2)	0.03	1.65 (1.21 to 2.25)	0.003
Immunosuppressant						
Past user	1.37 (0.55 to 3.39)	0.549	1.33 (0.54 to 3.28)	0.542	1.39 (0.56 to 3.43)	0.531
Current user	1.69 (0.82 to 3.48)	0.222	1.56 (0.76 to 3.22)	0.286	1.59 (0.77 to 3.28)	0.257

Odds ratios (ORs), except the crude ones, are mutually adjusted for gender, ethnicity, and age group, and also for additional conditions when mentioned inside the parenthesis. Dichotomous age groups (over and under 60) are used for controlling for all categories except demographics. All P values presented, except for the crude odds ratios, are Benjamini-Hochberg corrected.

For peer review only

Supplemental Table 5 Association between HPB disease and COVID-19 risk factors according to HPB disease subtypes

	Pancreatic disease			
	No (N=12434)	Acute (N=1230)	Chronic (N=1335)	
	OR (95% CI)	OR (95% CI)	OR (95% CI)	P-het
**Demographics**				
Gender (ref=Female)				0.33
Male	1.58 (1.15 to 2.15)	0.74 (0.29 to 1.88)	1.7 (0.79 to 3.63)	
Ethnicity (ref=White)				0.83
South Asian	1.36 (0.94 to 1.96)	1.82 (0.59 to 5.61)	1.29 (0.51 to 3.29)	
Black	2.07 (1.34 to 3.2)	4.14 (1.1 to 15.61)	2.02 (0.76 to 5.37)	
Other	0.92 (0.53 to 1.62)	1.37 (0.26 to 7.07)	0.74 (0.17 to 3.3)	
Age group (ref=18-40)				0.87
41-50	0.98 (0.5 to 1.9)	0.68 (0.11 to 4.27)	1.86 (0.45 to 7.59)	
51-60	1.26 (0.69 to 2.3)	0.79 (0.13 to 4.91)	0.62 (0.12 to 3.16)	
61-70	1.51 (0.83 to 2.76)	1.33 (0.26 to 6.85)	1.71 (0.42 to 7.02)	
71-80	2.76 (1.52 to 5.02)	3.14 (0.68 to 14.57)	2.02 (0.47 to 8.65)	
80+	5.9 (3.32 to 10.48)	6.07 (1.38 to 26.64)	4.63 (1.18 to 18.13)	
**HPB disease (ref=No)**				
Liver disease				0.15
Mild	1.8 (1.14 to 2.84)	2.27 (0.8 to 6.41)	1.65 (0.73 to 3.73)	
Moderate/Severe	2.82 (1.56 to 5.12)	4.9 (0.97 to 24.82)	1.54 (0.34 to 6.93)	
Biliary disease				0.04
Acute	1.05 (0.49 to 2.29)	0 (0 to Inf)	2.15 (0.61 to 7.64)	
Chronic	1.17 (0.76 to 1.78)	0.61 (0.22 to 1.66)	0.84 (0.33 to 2.15)	
**Comorbidities (ref=No)**				
Diabetes	2.34 (1.67 to 3.27)	3.2 (1.07 to 9.57)	3.39 (1.4 to 8.24)	0.18
Hypertension	2.22 (1.43 to 3.44)	2.67 (0.58 to 12.35)	1.91 (0.62 to 5.88)	0.65
Cholesterol	1.51 (1.07 to 2.13)	1.1 (0.38 to 3.18)	1.2 (0.53 to 2.68)	0.91
Cardiovascular	2.55 (1.83 to 3.55)	3.9 (1.36 to 11.21)	3.07 (1.35 to 6.98)	0.91
Renal	3 (2.16 to 4.16)	2.56 (0.93 to 7.06)	3.56 (1.6 to 7.94)	0.36
Respiratory	1.98 (1.46 to 2.7)	2.39 (0.94 to 6.07)	1.81 (0.87 to 3.76)	0.67
**Lifestyle factors (ref=Never)**				
Smoker				0.04
Past	1.52 (1.07 to 2.16)	0.74 (0.26 to 2.15)	1.32 (0.57 to 3.07)	
Current	0.71 (0.42 to 1.2)	0 (0 to Inf)	0.45 (0.14 to 1.42)	
Drinker				0.49
Past	1.44 (0.91 to 2.3)	0.24 (0.03 to 2.07)	0.91 (0.32 to 2.61)	
Current	0.87 (0.57 to 1.34)	0.56 (0.17 to 1.82)	0.43 (0.17 to 1.11)	
Substance user				0.4
Past	2.85 (1.24 to 6.58)	16.83 (1.95 to 145.12)	1.95 (0.43 to 8.95)	
Current	2.35 (1.39 to 3.95)	4.99 (0.96 to 26.04)	1.1 (0.37 to 3.25)	
Obese				0.59
Past	1.27 (0.83 to 1.96)	4.03 (1.23 to 13.24)	2.5 (1.05 to 5.92)	
Current	1.22 (0.87 to 1.73)	1.85 (0.56 to 6.1)	1.39 (0.56 to 3.46)	
**Prescription medication use (ref=Non-user)**				
ACE inhibitor				0.78
Past user	3 (1.86 to 4.82)	4.12 (1.02 to 16.7)	2.48 (0.68 to 9.02)	
Current user	0.7 (0.45 to 1.08)	1.11 (0.34 to 3.63)	1.22 (0.5 to 3)	

For peer review only

Angiotensin receptor blocker				0.36
Past user	1.52 (0.61 to 3.78)	0 (0 to Inf)	0 (0 to Inf)	
Current user	1.13 (0.71 to 1.77)	0.93 (0.2 to 4.29)	3.21 (1.26 to 8.15)	
Aldosterone agonist				0.33
Past user	3.45 (1.55 to 7.66)	0 (0 to Inf)	5.33 (1.06 to 26.73)	
Current user	1.16 (0.56 to 2.42)	2.47 (0.28 to 21.87)	1.19 (0.15 to 9.21)	
β-blocker				0.7
Past user	1.46 (0.67 to 3.19)	1.82 (0.21 to 15.44)	4.7 (1.47 to 15.02)	
Current user	1.63 (1.15 to 2.3)	1.76 (0.6 to 5.13)	1.21 (0.49 to 3.02)	
Calcium channel blocker				0.25
Past user	1.55 (0.86 to 2.8)	0 (0 to Inf)	2.54 (0.69 to 9.38)	
Current user	0.94 (0.64 to 1.38)	0.99 (0.33 to 2.95)	0.89 (0.36 to 2.24)	
α-agonist				0.93
Past user	0 (0 to 7.13e+251)	NA	NA	
Current user	1.2 (0.16 to 8.83)	0 (0 to Inf)	0 (0 to Inf)	
Thiazide				1
Past user	0 (0 to 3.25e+232)	0 (0 to Inf)	0 (0 to Inf)	
Current user	1.22 (0.17 to 8.97)	0 (0 to Inf)	0 (0 to Inf)	
Antiplatelet				0.82
Past user	1.63 (0.78 to 3.4)	1.79 (0.21 to 15.05)	0.77 (0.1 to 6.06)	
Current user	1.89 (1.32 to 2.71)	2.69 (0.95 to 7.67)	1.77 (0.75 to 4.15)	
Antiarrhythmic				0.23
Past user	1.39 (0.43 to 4.46)	3.17 (0.34 to 29.87)	3.29 (0.69 to 15.7)	
Current user	1.86 (1.09 to 3.16)	3.22 (0.65 to 15.87)	0.65 (0.08 to 4.96)	
Anticoagulant				0.33
Past user	1.72 (0.54 to 5.53)	5.22 (0.58 to 46.91)	0 (0 to Inf)	
Current user	1.55 (0.81 to 3)	1.91 (0.23 to 16.18)	0.71 (0.09 to 5.54)	
Glucocorticoid				0.88
Past user	1.41 (0.83 to 2.4)	0.69 (0.09 to 5.51)	1.29 (0.36 to 4.56)	
Current user	2.06 (1.47 to 2.88)	1.47 (0.52 to 4.11)	1.96 (0.88 to 4.38)	
β2-agonist				0.08
Past user	1.69 (0.68 to 4.2)	9.51 (2.34 to 38.57)	0 (0 to Inf)	
Current user	2.02 (1.37 to 2.99)	1.93 (0.52 to 7.23)	1.67 (0.65 to 4.28)	
Muscarinic antagonist				0.86
Past user	2.32 (1.12 to 4.83)	0 (0 to Inf)	2.79 (0.61 to 12.77)	
Current user	1.77 (1.17 to 2.68)	1.62 (0.44 to 5.96)	2.54 (1.07 to 5.99)	
NSAID				0.53
Past user	0.62 (0.27 to 1.41)	2.48 (0.52 to 11.86)	1.17 (0.26 to 5.15)	
Current user	1.44 (0.81 to 2.58)	3.08 (0.81 to 11.74)	0.87 (0.11 to 6.73)	
Vitamin D				0.41
Past user	1.93 (1.02 to 3.65)	3.91 (0.77 to 19.82)	2.63 (0.84 to 8.18)	
Current user	2.44 (1.71 to 3.48)	2.16 (0.71 to 6.62)	1.62 (0.68 to 3.88)	
Proton pump inhibitor				0.32
Past user	1.25 (0.69 to 2.28)	0 (0 to Inf)	0.55 (0.07 to 4.42)	
Current user	1.51 (1.08 to 2.1)	2.8 (0.95 to 8.29)	2.03 (0.88 to 4.68)	
Statin				0.89
Past user	2.25 (1.18 to 4.27)	2.21 (0.41 to 11.97)	1.77 (0.37 to 8.53)	
Current user	1.8 (1.26 to 2.57)	1.34 (0.45 to 3.96)	2.15 (0.92 to 5)	
Immunosuppressant				0.76
Past user	1.14 (0.36 to 3.65)	2.49 (0.27 to 22.9)	1.91 (0.24 to 15.09)	
Current user	1.85 (0.85 to 4)	2.82 (0.34 to 23.76)	0 (0 to Inf)	

For peer review only

	Liver disease			
	No (N=6863)	Mild (N=7098)	Moderate/Severe (N=1038)	
	OR (95% CI)	OR (95% CI)	OR (95% CI)	P-het
**Demographics**				
Gender (ref=Female)				0.41
Male	1.48 (0.94 to 2.31)	1.62 (1.1 to 2.37)	0.94 (0.42 to 2.11)	
Ethnicity (ref=White)				0.78
South Asian	1.29 (0.76 to 2.22)	1.51 (0.96 to 2.37)	1.07 (0.39 to 2.94)	
Black	1.9 (1.02 to 3.56)	2.58 (1.52 to 4.38)	1.87 (0.58 to 6.06)	
Other	0.65 (0.25 to 1.66)	1.09 (0.55 to 2.14)	1.12 (0.31 to 4.08)	
Age group (ref=18-40)				0.24
41-50	1.05 (0.35 to 3.13)	1.02 (0.5 to 2.07)	1.1 (0.18 to 6.81)	
51-60	0.79 (0.25 to 2.52)	1.21 (0.63 to 2.33)	0.71 (0.12 to 4.36)	
61-70	2.21 (0.86 to 5.68)	1.04 (0.51 to 2.1)	2.38 (0.5 to 11.33)	
71-80	3.57 (1.44 to 8.82)	2.08 (1.04 to 4.18)	3.34 (0.63 to 17.88)	
80+	9.14 (4 to 20.85)	3.45 (1.67 to 7.12)	3.99 (0.64 to 24.98)	
**HPB disease (ref=No)**				
Pancreatic disease				0.02
Acute	1.32 (0.64 to 2.75)	1.6 (0.69 to 3.73)	1.89 (0.42 to 8.58)	
Chronic	1.96 (0.97 to 3.98)	1.96 (1 to 3.83)	1.12 (0.25 to 5.06)	
Biliary disease				0.01
Acute	1.15 (0.43 to 3.03)	0.93 (0.29 to 3)	1.07 (0.14 to 8.46)	
Chronic	1.03 (0.52 to 2.05)	0.93 (0.57 to 1.52)	1.79 (0.74 to 4.32)	
**Comorbidities (ref=No)**				
Diabetes	2.73 (1.66 to 4.5)	2.36 (1.56 to 3.56)	2.41 (0.98 to 5.93)	0.14
Hypertension	2.08 (1.05 to 4.14)	1.87 (1.13 to 3.1)	8.74 (1.14 to 66.9)	0.1
Cholesterol	1.16 (0.71 to 1.91)	1.43 (0.94 to 2.16)	2.06 (0.79 to 5.4)	0.76
Cardiovascular	2.19 (1.34 to 3.57)	2.85 (1.9 to 4.27)	3.93 (1.6 to 9.66)	0.79
Renal	2.61 (1.63 to 4.18)	3.98 (2.64 to 5.99)	1.56 (0.67 to 3.63)	0.16
Respiratory	2.29 (1.47 to 3.58)	1.92 (1.31 to 2.8)	1.47 (0.65 to 3.35)	0.39
**Lifestyle factors (ref=Never)**				
Smoker				0.15
Past	1.36 (0.84 to 2.22)	1.35 (0.88 to 2.09)	1.63 (0.61 to 4.3)	
Current	0.29 (0.1 to 0.85)	0.7 (0.39 to 1.28)	0.71 (0.2 to 2.55)	
Drinker				0.66
Past	0.94 (0.47 to 1.9)	1.33 (0.76 to 2.3)	1.8 (0.46 to 7)	
Current	0.97 (0.55 to 1.72)	0.6 (0.36 to 1.01)	1 (0.29 to 3.45)	
Substance user				0.04
Past	3.43 (1.14 to 10.3)	3.53 (1.46 to 8.51)	0 (0 to Inf)	
Current	2.84 (1.37 to 5.87)	1.45 (0.78 to 2.69)	7.26 (0.9 to 58.68)	
Obese				0.55
Past	2.01 (1.14 to 3.54)	1.14 (0.65 to 1.98)	2.59 (0.97 to 6.94)	
Current	1.43 (0.84 to 2.42)	1.17 (0.77 to 1.77)	1.78 (0.65 to 4.86)	
**Prescription medication use (ref=Non-user)**				
ACE inhibitor				0.31
Past user	3.67 (1.89 to 7.1)	2.05 (1.04 to 4.05)	5.91 (2.11 to 16.55)	
Current user	0.93 (0.51 to 1.67)	0.66 (0.39 to 1.12)	1.07 (0.34 to 3.36)	
Angiotensin receptor blocker				0.08
Past user	1.24 (0.29 to 5.2)	0 (0 to Inf)	5.41 (1.36 to 21.48)	
Current user	1.09 (0.56 to 2.1)	1.61 (0.97 to 2.67)	0.48 (0.06 to 3.67)	

For peer review only

Aldosterone agonist				0.17
Past user	4.13 (1.18 to 14.45)	3.9 (1.38 to 11.03)	2.26 (0.49 to 10.39)	
Current user	0.5 (0.07 to 3.64)	0.9 (0.28 to 2.87)	2.36 (0.89 to 6.25)	
β-blocker				0.33
Past user	0.79 (0.19 to 3.29)	1.79 (0.71 to 4.52)	6.03 (1.96 to 18.56)	
Current user	1.32 (0.8 to 2.18)	1.94 (1.26 to 2.97)	1.28 (0.5 to 3.23)	
Calcium channel blocker				0.08
Past user	1.91 (0.92 to 3.98)	1.45 (0.65 to 3.24)	0 (0 to Inf)	
Current user	0.57 (0.31 to 1.06)	1.29 (0.83 to 2)	0.9 (0.32 to 2.53)	
α-agonist				0.93
Past user	0 (0 to Inf)	0 (0 to Inf)	0 (0 to Inf)	
Current user	1.8 (0.23 to 13.81)	0 (0 to Inf)	0 (0 to Inf)	
Thiazide				0.76
Past user	0 (0 to Inf)	0 (0 to Inf)	NA	
Current user	0 (0 to Inf)	2.14 (0.28 to 16.16)	0 (0 to Inf)	
Antiplatelet				0.77
Past user	1.43 (0.5 to 4.08)	1.56 (0.62 to 3.94)	1.28 (0.16 to 10.39)	
Current user	1.82 (1.1 to 3.01)	1.93 (1.23 to 3.03)	2.39 (0.96 to 5.92)	
Antiarrhythmic				0.13
Past user	3.69 (1.28 to 10.63)	1.28 (0.3 to 5.4)	0 (0 to Inf)	
Current user	1.35 (0.57 to 3.19)	2.35 (1.23 to 4.51)	1.13 (0.25 to 5.05)	
Anticoagulant				0.5
Past user	1.9 (0.45 to 8.04)	1.67 (0.4 to 7.06)	0 (0 to Inf)	
Current user	1.46 (0.58 to 3.71)	1.63 (0.7 to 3.82)	0.8 (0.1 to 6.32)	
Glucocorticoid				0.56
Past user	1.3 (0.58 to 2.92)	1.48 (0.8 to 2.73)	0.56 (0.07 to 4.41)	
Current user	2.3 (1.43 to 3.7)	1.8 (1.18 to 2.73)	1.76 (0.74 to 4.18)	
β2-agonist				0.28
Past user	2.86 (0.87 to 9.43)	2.04 (0.81 to 5.13)	0 (0 to Inf)	
Current user	2.22 (1.3 to 3.8)	1.73 (1.04 to 2.88)	1.89 (0.67 to 5.29)	
Muscarinic antagonist				0.44
Past user	1.22 (0.29 to 5.11)	2.91 (1.31 to 6.46)	2.27 (0.28 to 18.28)	
Current user	2.3 (1.35 to 3.91)	1.4 (0.8 to 2.45)	2.25 (0.8 to 6.33)	
NSAID				0.17
Past user	1.42 (0.6 to 3.34)	0.42 (0.13 to 1.32)	0.94 (0.12 to 7.49)	
Current user	2.49 (1.22 to 5.11)	1.21 (0.58 to 2.53)	0 (0 to Inf)	
Vitamin D				0.06
Past user	4.19 (2.1 to 8.36)	1.36 (0.54 to 3.42)	0.58 (0.07 to 4.54)	
Current user	2.28 (1.36 to 3.82)	2.51 (1.61 to 3.9)	1.5 (0.61 to 3.69)	
Proton pump inhibitor				0.73
Past user	0.78 (0.3 to 2.02)	1.4 (0.65 to 3.03)	0.62 (0.07 to 5.22)	
Current user	1.12 (0.7 to 1.8)	2.1 (1.38 to 3.19)	2.17 (0.87 to 5.42)	
Statin				0.35
Past user	3.23 (1.45 to 7.2)	1.3 (0.5 to 3.33)	2.04 (0.41 to 10.12)	
Current user	1.7 (1 to 2.9)	1.76 (1.15 to 2.69)	2.08 (0.85 to 5.1)	
Immunosuppressant				0.65
Past user	2.45 (0.74 to 8.08)	1.09 (0.26 to 4.53)	0 (0 to Inf)	
Current user	1.25 (0.3 to 5.23)	1.66 (0.6 to 4.6)	3.04 (0.66 to 14.08)	

For peer review only

	Biliary disease			
	No (N=7716)	Acute (N=749)	Chronic (N=6534)	P-het
	OR (95% CI)	OR (95% CI)	OR (95% CI)	
**Demographics**				
Gender (ref=Female)				0.72
Male	1.35 (0.94 to 1.94)	1.35 (0.38 to 4.83)	1.58 (1.01 to 2.47)	
Ethnicity (ref=White)				0.6
South Asian	1.33 (0.86 to 2.03)	1.67 (0.43 to 6.57)	1.42 (0.83 to 2.42)	
Black	1.78 (1.06 to 2.98)	2.26 (0.41 to 12.54)	3.2 (1.75 to 5.86)	
Other	0.98 (0.52 to 1.84)	0 (0 to Inf)	0.99 (0.43 to 2.26)	
Age group (ref=18-40)				0.13
41-50	1.1 (0.57 to 2.09)	2.4 (0.21 to 27.67)	0.63 (0.15 to 2.63)	
51-60	1.01 (0.54 to 1.89)	1.44 (0.12 to 17.27)	1.36 (0.44 to 4.22)	
61-70	1.07 (0.56 to 2.05)	0.95 (0.06 to 15.97)	3.37 (1.23 to 9.29)	
71-80	1.71 (0.88 to 3.33)	1.18 (0.07 to 20.15)	6.46 (2.39 to 17.44)	
80+	4.16 (2.19 to 7.89)	6.53 (0.66 to 64.15)	10.15 (3.86 to 26.68)	
**HPB disease (ref=No)**				
Pancreatic disease				0.93
Acute	1.92 (0.97 to 3.82)	0 (0 to Inf)	1.04 (0.44 to 2.43)	
Chronic	2.2 (1.22 to 3.98)	3.68 (0.87 to 15.57)	1.25 (0.53 to 2.96)	
Liver disease				0.59
Mild	2.05 (1.09 to 3.88)	1.22 (0.29 to 5.17)	1.55 (0.92 to 2.61)	
Moderate/Severe	2.63 (1.22 to 5.66)	1.45 (0.15 to 13.66)	3.95 (1.8 to 8.67)	
**Comorbidities (ref=No)**				
Diabetes	2.58 (1.75 to 3.82)	2.49 (0.64 to 9.69)	2.25 (1.36 to 3.71)	0.22
Hypertension	2.16 (1.33 to 3.5)	0.87 (0.18 to 4.14)	2.5 (1.15 to 5.45)	0.16
Cholesterol	1.3 (0.89 to 1.9)	1.49 (0.36 to 6.16)	1.48 (0.87 to 2.52)	0.59
Cardiovascular	3.31 (2.26 to 4.86)	1.04 (0.26 to 4.19)	2.09 (1.28 to 3.41)	0.45
Renal	3.21 (2.19 to 4.72)	2.73 (0.73 to 10.11)	2.73 (1.7 to 4.38)	0.23
Respiratory	1.96 (1.37 to 2.8)	4.25 (1.2 to 15.04)	1.77 (1.13 to 2.77)	0.43
**Lifestyle factors (ref=Never)**				
Smoker				0.14
Past	1.2 (0.8 to 1.81)	2.38 (0.54 to 10.62)	1.58 (0.97 to 2.58)	
Current	0.55 (0.31 to 0.97)	1.42 (0.21 to 9.7)	0.38 (0.13 to 1.09)	
Drinker				0.9
Past	1.38 (0.79 to 2.42)	0.41 (0.04 to 3.97)	1.15 (0.61 to 2.16)	
Current	0.75 (0.46 to 1.23)	0.71 (0.16 to 3.12)	0.76 (0.42 to 1.37)	
Substance user				0.03
Past	2.6 (1.03 to 6.53)	0 (0 to Inf)	4.95 (1.68 to 14.58)	
Current	1.41 (0.78 to 2.57)	0.97 (0.19 to 4.91)	4.5 (2.08 to 9.74)	
Obese				0.84
Past	1.58 (0.98 to 2.56)	0.73 (0.14 to 3.88)	1.83 (1.01 to 3.31)	
Current	1.23 (0.82 to 1.84)	0.68 (0.16 to 2.93)	1.65 (0.96 to 2.81)	
**Prescription medication use (ref=Non-user)**				
ACE inhibitor				0.88
Past user	3.04 (1.72 to 5.38)	4.61 (0.79 to 26.78)	2.84 (1.44 to 5.61)	
Current user	0.76 (0.46 to 1.25)	1.09 (0.21 to 5.75)	0.8 (0.44 to 1.43)	
Angiotensin receptor blocker				0.74
Past user	0.77 (0.19 to 3.16)	0 (0 to Inf)	1.73 (0.52 to 5.77)	
Current user	1.37 (0.82 to 2.3)	3.39 (0.77 to 14.93)	1.01 (0.52 to 1.95)	

For peer review only

Aldosterone agonist				0.31
Past user	4.05 (1.7 to 9.64)	0 (0 to Inf)	2.62 (0.76 to 9.05)	
Current user	1.32 (0.57 to 3.09)	0 (0 to Inf)	1.27 (0.45 to 3.61)	
**β**-blocker				0.23
Past user	2.42 (1.15 to 5.09)	8.19 (0.78 to 86.4)	1.1 (0.33 to 3.6)	
Current user	1.52 (1 to 2.31)	0.39 (0.05 to 3.31)	1.77 (1.1 to 2.85)	
Calcium channel blocker				0.12
Past user	1.19 (0.51 to 2.79)	2.22 (0.24 to 20.37)	1.64 (0.79 to 3.42)	
Current user	1.28 (0.84 to 1.95)	2.19 (0.55 to 8.83)	0.49 (0.26 to 0.91)	
α-agonist				0.75
Past user	0 (0 to Inf)	NA	0 (0 to Inf)	
Current user	0 (0 to Inf)	0 (0 to Inf)	2.38 (0.3 to 18.73)	
Thiazide				0.81
Past user	0 (0 to Inf)	NA	0 (0 to Inf)	
Current user	1.94 (0.26 to 14.41)	0 (0 to Inf)	0 (0 to Inf)	
Antiplatelet				0.4
Past user	1.59 (0.68 to 3.7)	0 (0 to Inf)	1.36 (0.47 to 3.91)	
Current user	1.77 (1.15 to 2.73)	1.38 (0.32 to 5.95)	2.11 (1.29 to 3.45)	
Antiarrhythmic				0.34
Past user	2.07 (0.64 to 6.74)	0 (0 to Inf)	2.15 (0.64 to 7.22)	
Current user	1.95 (1.03 to 3.7)	2.71 (0.31 to 23.91)	1.41 (0.63 to 3.16)	
Anticoagulant				0.49
Past user	1.43 (0.34 to 5.94)	0 (0 to Inf)	2.01 (0.47 to 8.59)	
Current user	1.31 (0.56 to 3.03)	5.28 (0.58 to 48.33)	1.31 (0.51 to 3.36)	
Glucocorticoid				0.35
Past user	1.69 (0.96 to 2.99)	1.11 (0.13 to 9.69)	0.83 (0.33 to 2.13)	
Current user	1.77 (1.19 to 2.64)	1.27 (0.31 to 5.22)	2.38 (1.49 to 3.8)	
**β**2-agonist				0.46
Past user	2.46 (1.06 to 5.75)	0 (0 to Inf)	1.52 (0.36 to 6.36)	
Current user	1.89 (1.18 to 3.02)	0.85 (0.1 to 7.22)	2.09 (1.22 to 3.57)	
Muscarinic antagonist				0.52
Past user	3.51 (1.66 to 7.41)	0 (0 to Inf)	1.04 (0.25 to 4.33)	
Current user	1.72 (1.05 to 2.83)	2.14 (0.42 to 10.99)	2.06 (1.19 to 3.56)	
NSAID				0.65
Past user	0.58 (0.21 to 1.59)	0 (0 to Inf)	1.23 (0.52 to 2.91)	
Current user	1.7 (0.88 to 3.3)	1.97 (0.23 to 17.04)	1.22 (0.52 to 2.86)	
Vitamin D				0.15
Past user	1.87 (0.89 to 3.94)	6.13 (1.03 to 36.35)	2.34 (1.03 to 5.32)	
Current user	2.35 (1.54 to 3.58)	0.56 (0.07 to 4.71)	2.45 (1.5 to 4.01)	
Proton pump inhibitor				0.42
Past user	1.03 (0.46 to 2.29)	0.94 (0.11 to 7.98)	1.09 (0.44 to 2.67)	
Current user	1.87 (1.28 to 2.74)	0.39 (0.09 to 1.72)	1.53 (0.93 to 2.51)	
Statin				0.22
Past user	1.71 (0.76 to 3.84)	0 (0 to Inf)	3.31 (1.47 to 7.47)	
Current user	1.82 (1.22 to 2.72)	1.06 (0.28 to 4.03)	1.72 (1 to 2.96)	
Immunosuppressant				0.36
Past user	0.49 (0.07 to 3.56)	0 (0 to Inf)	3.28 (1.15 to 9.38)	
Current user	2 (0.8 to 4.99)	6.18 (0.65 to 59.04)	0.98 (0.23 to 4.07)	

Odds ratios (ORs) are mutually adjusted for gender, ethnicity, age group, and other HPB diagnoses.
Dichotomous age groups (over and under 60) are used for controlling for all categories except demographics.
All P values presented are Benjamini-Hochberg corrected.

For peer review only

Supplemental Table 6 Odds ratio estimates of COVID-19 mortality for HPB patients with specific demographic, comorbidity, lifestyle, medication use, and post diagnosis complication characteristics.

	Crude OR (95% CI)	P value	Adjusted OR (95% CI)	P value	Adjusted OR (+all comorbidity) (95% CI)	P value
**Demographics**						
Gender (ref=Female)						
Male	2.53 (1.29 to 4.96)	0.007	3.54 (1.68 to 7.85)	0.007	3.43 (1.61 to 7.67)	0.008
Ethnicity (ref=White)						
South Asian	1.39 (0.67 to 2.89)	0.477	2.08 (0.91 to 4.88)	0.143	1.52 (0.64 to 3.62)	0.492
Black	2.35 (1.02 to 5.41)	0.109	3.77 (1.38 to 10.7)	0.023	3.51 (1.27 to 10)	0.053
Other	0.21 (0.03 to 1.63)	0.225	0.29 (0.02 to 1.82)	0.371	0.18 (0.01 to 1.19)	0.259
Age group (ref=18-40)						
41-50	1.83 (0.15 to 21.6)	0.76	2.24 (0.19 to 52.1)	0.65	1.86 (0.13 to 48.8)	0.858
51-60	1.35 (0.12 to 15.9)	0.809	1.92 (0.16 to 44.3)	0.674	1.48 (0.11 to 37.4)	0.938
61-70	5.09 (0.59 to 43.7)	0.207	6.73 (1.03 to 134)	0.143	4.12 (0.51 to 90.6)	0.379
71-80	13.4 (1.64 to 110)	0.031	18.6 (3.12 to 361)	0.022	8.8 (1.12 to 192)	0.204
80+	16.3 (2.04 to 130)	0.025	25.4 (4.32 to 491)	0.012	13.2 (1.76 to 283)	0.13
**HPB disease (ref=No)**						
Cancer						
Yes	2.1 (0.34 to 12.9)	0.422	1.18 (0.14 to 10.2)	0.951	1.35 (0.13 to 13.7)	0.959
Pancreatic disease						
Acute	1.4 (0.47 to 4.15)	0.549	1.52 (0.44 to 5.24)	0.596	1.16 (0.32 to 4.23)	0.959
Chronic	2.82 (1.26 to 6.34)	0.024	3.26 (1.13 to 9.44)	0.082	2.65 (0.84 to 8.32)	0.283
Liver disease						
Mild	0.43 (0.22 to 0.85)	0.03	0.51 (0.19 to 1.35)	0.347	0.43 (0.15 to 1.19)	0.283
Moderate/Severe	0.53 (0.18 to 1.56)	0.33	0.6 (0.16 to 2.22)	0.568	0.47 (0.12 to 1.81)	0.454
Biliary disease						
Acute	3.81 (1.02 to 14.1)	0.092	2.44 (0.38 to 15.9)	0.544	3.98 (0.46 to 34.6)	0.42
Chronic	1.34 (0.68 to 2.61)	0.395	0.67 (0.26 to 1.72)	0.568	0.62 (0.24 to 1.62)	0.513
**Comorbidities (ref=No)**						
Diabetes	2.51 (1.24 to 5.44)	0.014	1.88 (0.81 to 4.38)	0.227	1.71 (0.71 to 4.11)	0.38
Hypertension	12.6 (2.6 to 227)	0.014	6.3 (0.74 to 53.8)	0.148	5.57 (0.57 to 54.3)	0.259
Cholesterol	1.87 (0.93 to 3.97)	0.088	1.25 (0.56 to 2.82)	0.67	0.95 (0.4 to 2.22)	0.979
Cardiovascular	4.1 (2 to 9.13)	<0.001	2.82 (1.21 to 6.59)	0.026	2.66 (1.05 to 6.79)	0.104
Renal	3.67 (1.9 to 7.38)	<0.001	1.72 (0.8 to 3.7)	0.248	1.07 (0.47 to 2.47)	0.979
Respiratory	1.09 (0.59 to 2.03)	0.784	0.85 (0.42 to 1.73)	0.743	0.83 (0.38 to 1.78)	0.815
**Lifestyle factors (ref=Never)**						
Smoker						
Past	2.46 (1.22 to 4.95)	0.023	1.87 (0.83 to 4.19)	0.219	1.86 (0.79 to 4.36)	0.274
Current	0.42 (0.09 to 1.97)	0.359	0.45 (0.08 to 2.49)	0.451	0.44 (0.08 to 2.58)	0.533
Drinker						
Past	1.38 (0.55 to 3.45)	0.761	1.2 (0.41 to 3.49)	0.847	1.07 (0.36 to 3.22)	0.964
Current	1.26 (0.57 to 2.79)	0.761	1.38 (0.52 to 3.64)	0.736	1.44 (0.52 to 3.96)	0.774

For peer review only

Substance user						
Past	1.2 (0.19 to 7.64)	0.847	0.62 (0.08 to 4.94)	0.81	0.31 (0.03 to 2.87)	0.488
Current	3.17 (1 to 10)	0.1	2.01 (0.53 to 7.64)	0.436	1.34 (0.32 to 5.55)	0.842
Obese						
Past	1.64 (0.74 to 3.66)	0.453	1.46 (0.58 to 3.65)	0.603	0.97 (0.36 to 2.59)	0.995
Current	1.04 (0.5 to 2.16)	0.986	1.03 (0.44 to 2.4)	0.991	0.91 (0.37 to 2.2)	0.995
**Prescription medication use (ref=Non-user)**						
ACE inhibitor						
Past user	1.48 (0.6 to 3.66)	0.397	1.02 (0.38 to 2.75)	0.988	0.84 (0.29 to 2.42)	0.889
Current user	2.02 (0.9 to 4.5)	0.13	2.25 (0.88 to 5.73)	0.161	1.97 (0.73 to 5.32)	0.337
Angiotensin receptor blocker						
Past user	2.12 (0.34 to 13.1)	0.626	1.07 (0.14 to 8.29)	0.988	0.78 (0.1 to 6.34)	0.987
Current user	1.06 (0.44 to 2.54)	0.892	0.86 (0.32 to 2.32)	0.988	0.81 (0.28 to 2.35)	0.946
Aldosterone agonist						
Past user	1.6 (0.38 to 6.63)	0.78	1.29 (0.28 to 5.82)	0.838	0.88 (0.19 to 4.1)	0.989
Current user	1.2 (0.3 to 4.69)	0.797	1.29 (0.28 to 5.97)	0.838	0.97 (0.2 to 4.67)	0.989
β-blocker						
Past user	1.1 (0.28 to 4.32)	0.887	0.72 (0.16 to 3.24)	0.746	0.29 (0.06 to 1.46)	0.204
Current user	1.22 (0.62 to 2.38)	0.848	0.66 (0.3 to 1.45)	0.385	0.26 (0.1 to 0.67)	0.017
Calcium channel blocker						
Past user	0.47 (0.1 to 2.18)	0.338	0.16 (0.03 to 0.89)	0.065	0.07 (0.01 to 0.45)	0.016
Current user	1.56 (0.77 to 3.15)	0.324	0.92 (0.4 to 2.12)	0.948	0.52 (0.21 to 1.29)	0.243
α-agonist						
Past user	0 (0 to Inf)	0.988	0 (0 to Inf)	0.992	0 (0 to Inf)	0.994
Thiazide						
Past user	0 (0 to Inf)	0.988	0 (0 to Inf)	0.993	0 (0 to Inf)	0.995
Current user	1 (0.2 to 4.97)	1	0.68 (0.12 to 3.7)	0.836	0.53 (0.09 to 3.17)	0.659
Antiplatelet						
Past user	2.09 (1.09 to 4.02)	0.04	0.99 (0.45 to 2.17)	0.988	0.68 (0.29 to 1.58)	0.56
Antiarrhythmic						
Past user	0.57 (0.07 to 4.86)	0.607	0.53 (0.06 to 4.82)	0.641	0.31 (0.03 to 3.1)	0.529
Current user	2.8 (1.09 to 7.19)	0.049	2.12 (0.72 to 6.26)	0.261	1.66 (0.52 to 5.3)	0.531
Anticoagulant						
Past user	0.81 (0.09 to 7.41)	0.851	0.48 (0.05 to 4.89)	0.691	0.34 (0.03 to 3.57)	0.555
Current user	2.02 (0.63 to 6.48)	0.354	1.18 (0.32 to 4.39)	0.904	0.94 (0.24 to 3.66)	0.989
Glucocorticoid						
Past user	1.88 (0.65 to 5.41)	0.245	1.47 (0.44 to 4.87)	0.599	1.62 (0.46 to 5.68)	0.564
Current user	2.64 (1.35 to 5.18)	0.007	2.79 (1.26 to 6.22)	0.021	3.66 (1.49 to 9.02)	0.014
β2-agonist						
Past user	1.32 (0.26 to 6.86)	0.739	1.96 (0.32 to 12)	0.523	5.41 (0.65 to 45.1)	0.222
Current user	2.65 (1.3 to 5.37)	0.011	2.72 (1.18 to 6.25)	0.034	5.24 (1.78 to 15.4)	0.01
Muscarinic antagonist						
Past user	2.65 (0.68 to 10.3)	0.242	1.58 (0.32 to 7.7)	0.739	1.85 (0.32 to 10.5)	0.733
Current user	1.1 (0.5 to 2.45)	0.811	1.11 (0.45 to 2.76)	0.919	1.16 (0.44 to 3.09)	0.95

For peer review only

NSAID						
Past user	0.85 (0.17 to 4.15)	0.84	0.58 (0.11 to 3.05)	0.589	0.7 (0.12 to 3.99)	0.795
Current user	2.72 (1.01 to 7.31)	0.072	4.13 (1.19 to 14.3)	0.045	4.24 (1.15 to 15.6)	0.089
Vitamin D						
Past user	2.1 (0.72 to 6.11)	0.175	1.8 (0.55 to 5.92)	0.376	1.51 (0.43 to 5.34)	0.653
Current user	2.05 (1.04 to 4.02)	0.056	1.58 (0.7 to 3.53)	0.345	1.5 (0.61 to 3.7)	0.625
Proton pump inhibitor						
Past user	0.94 (0.24 to 3.73)	0.929	1.46 (0.29 to 7.34)	0.83	0.95 (0.17 to 5.35)	0.989
Current user	1.21 (0.62 to 2.35)	0.855	0.88 (0.41 to 1.89)	0.83	0.51 (0.22 to 1.23)	0.27
Statin						
Past user	1.64 (0.4 to 6.74)	0.495	0.82 (0.18 to 3.77)	0.902	0.4 (0.07 to 2.25)	0.494
Current user	3.19 (1.52 to 6.69)	0.003	1.51 (0.62 to 3.66)	0.47	0.82 (0.27 to 2.47)	0.882
Immunosuppressant						
Past user	2.17 (0.35 to 13.3)	0.405	1.3 (0.17 to 10)	0.904	1.63 (0.19 to 14.1)	0.819
Current user	1.95 (0.45 to 8.46)	0.405	2.44 (0.44 to 13.7)	0.4	3.41 (0.56 to 20.6)	0.341
**Complications post diagnosis (ref=No)**						
**Cardiovascular**						
Recurrent	3.75 (1.71 to 8.25)	0.001	2.53 (1.04 to 6.12)	0.071	2.37 (0.9 to 6.26)	0.231
Novel	0.52 (0.06 to 4.47)	0.555	0.44 (0.04 to 4.47)	0.547	0.42 (0.04 to 4.7)	0.668
**Respiratory**						
Recurrent	3.31 (1.19 to 9.16)	0.021	2.53 (0.84 to 7.63)	0.15	2.69 (0.84 to 8.63)	0.188
Novel	5.88 (2.02 to 17.1)	0.002	5.77 (1.75 to 19)	0.009	6.55 (1.88 to 22.9)	0.011
**Renal**						
Recurrent	4.75 (2.06 to 10.9)	<0.001	1.72 (0.66 to 4.46)	0.34	0.8 (0.27 to 2.35)	0.797
Novel	1.95 (0.65 to 5.88)	0.233	1 (0.3 to 3.34)	0.997	0.58 (0.16 to 2.16)	0.585

	**Adjusted OR (+Diabetes) (95% CI)**	**P value**	**Adjusted OR (+Hypertension) (95% CI)**	**P value**	**Adjusted OR (+Cholesterol) (95% CI)**	**P value**
**Demographics**						
Gender (ref=Female)						
Male	3.31 (1.6 to 7.19)	0.005	3.35 (1.62 to 7.24)	0.004	3.18 (1.55 to 6.84)	0.006
Ethnicity (ref=White)						
South Asian	1.55 (0.67 to 3.6)	0.349	1.79 (0.79 to 4.06)	0.216	1.8 (0.8 to 4.08)	0.252
Black	3.23 (1.22 to 8.82)	0.039	3.67 (1.4 to 9.97)	0.018	3.55 (1.36 to 9.57)	0.021
Other	0.24 (0.01 to 1.48)	0.267	0.26 (0.01 to 1.61)	0.261	0.27 (0.01 to 1.63)	0.314
Age group (ref=18-40)						
41-50	1.88 (0.16 to 44.3)	0.753	2.43 (0.2 to 58.2)	0.597	2.1 (0.17 to 50.1)	0.76
51-60	1.47 (0.12 to 34.8)	0.836	2.01 (0.17 to 47.3)	0.646	1.81 (0.15 to 42.7)	0.778
61-70	4.91 (0.71 to 100)	0.287	6.1 (0.9 to 124)	0.196	6.34 (0.93 to 129)	0.184
71-80	12.4 (1.91 to 249)	0.063	15.3 (2.45 to 301)	0.035	17.3 (2.69 to 347)	0.028
80+	19.9 (3.26 to 391)	0.029	20 (3.25 to 395)	0.029	23.8 (3.83 to 473)	0.019

For peer review only

**HPB disease (ref=No)**						
Cancer						
Yes	1.41 (0.16 to 12.8)	0.811	1.04 (0.12 to 9.04)	0.988	1.16 (0.13 to 10.3)	0.957
Pancreatic disease						
Acute	1.5 (0.43 to 5.18)	0.605	1.37 (0.4 to 4.74)	0.715	1.5 (0.43 to 5.18)	0.653
Chronic	2.88 (0.97 to 8.55)	0.171	3.19 (1.09 to 9.34)	0.102	3.19 (1.1 to 9.27)	0.099
Liver disease						
Mild	0.49 (0.19 to 1.31)	0.39	0.48 (0.18 to 1.27)	0.26	0.5 (0.19 to 1.32)	0.346
Moderate/Severe	0.58 (0.16 to 2.12)	0.509	0.53 (0.14 to 1.97)	0.471	0.59 (0.16 to 2.19)	0.591
Biliary disease						
Acute	2.45 (0.37 to 16.5)	0.496	3.05 (0.38 to 24.2)	0.437	2.4 (0.36 to 15.9)	0.591
Chronic	0.65 (0.25 to 1.66)	0.496	0.67 (0.26 to 1.7)	0.499	0.67 (0.26 to 1.71)	0.591
**Comorbidities (ref=No)**						
Diabetes	1.88 (0.81 to 4.38)	0.227	1.87 (0.8 to 4.38)	0.222	1.86 (0.8 to 4.33)	0.271
Hypertension	6.35 (0.73 to 55.4)	0.17	6.3 (0.74 to 53.8)	0.148	6.09 (0.7 to 52.7)	0.181
Cholesterol	1.21 (0.53 to 2.74)	0.734	1.1 (0.48 to 2.52)	0.914	1.25 (0.56 to 2.82)	0.67
Cardiovascular	2.76 (1.17 to 6.48)	0.036	2.66 (1.13 to 6.27)	0.046	2.8 (1.19 to 6.62)	0.034
Renal	1.61 (0.74 to 3.5)	0.291	1.53 (0.71 to 3.31)	0.312	1.7 (0.79 to 3.66)	0.287
Respiratory	0.93 (0.45 to 1.94)	0.963	0.9 (0.44 to 1.84)	0.86	0.84 (0.41 to 1.72)	0.719
**Lifestyle factors (ref=Never)**						
Smoker						
Past	1.92 (0.84 to 4.37)	0.218	1.86 (0.82 to 4.2)	0.222	1.85 (0.82 to 4.17)	0.252
Current	0.42 (0.08 to 2.37)	0.401	0.46 (0.08 to 2.58)	0.458	0.45 (0.08 to 2.5)	0.498
Drinker						
Past	1.19 (0.41 to 3.48)	0.824	1.21 (0.41 to 3.53)	0.806	1.18 (0.4 to 3.45)	0.84
Current	1.42 (0.54 to 3.75)	0.657	1.29 (0.49 to 3.43)	0.806	1.37 (0.52 to 3.62)	0.812
Substance user						
Past	0.65 (0.08 to 5.32)	0.837	0.53 (0.07 to 4.35)	0.68	0.57 (0.07 to 4.69)	0.745
Current	2.17 (0.56 to 8.41)	0.361	1.65 (0.43 to 6.32)	0.634	1.92 (0.5 to 7.37)	0.538
Obese						
Past	1.33 (0.52 to 3.39)	0.749	1.34 (0.53 to 3.39)	0.733	1.43 (0.57 to 3.61)	0.704
Current	0.92 (0.39 to 2.18)	0.991	1.05 (0.45 to 2.46)	0.994	1.03 (0.44 to 2.4)	0.991
**Prescription medication use (ref=Non-user)**						
ACE inhibitor						
Past user	0.97 (0.36 to 2.61)	0.988	0.93 (0.34 to 2.53)	0.986	0.99 (0.36 to 2.74)	0.988
Current user	2.07 (0.8 to 5.34)	0.25	1.96 (0.76 to 5.01)	0.234	2.19 (0.84 to 5.69)	0.218
Angiotensin receptor blocker						
Past user	0.95 (0.12 to 7.27)	0.988	1 (0.13 to 7.75)	0.998	1.16 (0.15 to 8.83)	0.988
Current user	0.82 (0.3 to 2.24)	0.877	0.8 (0.3 to 2.17)	0.823	0.82 (0.3 to 2.23)	0.866
Aldosterone agonist						
Past user	1.26 (0.28 to 5.72)	0.86	1.15 (0.26 to 5.2)	0.947	1.24 (0.28 to 5.64)	0.892
Current user	1.25 (0.27 to 5.78)	0.86	1.18 (0.25 to 5.49)	0.947	1.22 (0.26 to 5.75)	0.892
β-blocker						
Past user	0.68 (0.15 to 3.04)	0.679	0.6 (0.13 to 2.71)	0.561	0.73 (0.16 to 3.32)	0.758

Current user	0.6 (0.27 to 1.35)	0.298	0.56 (0.25 to 1.26)	0.229	0.61 (0.27 to 1.38)	0.397
Calcium channel blocker						
Past user	0.14 (0.02 to 0.83)	0.06	0.13 (0.02 to 0.76)	0.047	0.14 (0.02 to 0.82)	0.058
Current user	0.89 (0.38 to 2.07)	0.876	0.79 (0.34 to 1.84)	0.656	0.88 (0.38 to 2.05)	0.852
α-agonist						
Past user	0 (0 to Inf)	0.992	0 (0 to Inf)	0.995	0 (0 to Inf)	0.992
Thiazide						
Past user	0 (0 to Inf)	0.993	0 (0 to Inf)	0.995	0 (0 to Inf)	0.993
Current user	0.69 (0.12 to 3.89)	0.844	0.66 (0.12 to 3.67)	0.79	0.64 (0.12 to 3.57)	0.764
Antiplatelet						
Past user	0.96 (0.44 to 2.11)	0.988	0.96 (0.44 to 2.11)	0.988	0.96 (0.44 to 2.12)	0.988
Antiarrhythmic						
Past user	0.44 (0.05 to 4.11)	0.519	0.47 (0.05 to 4.3)	0.56	0.53 (0.06 to 4.89)	0.688
Current user	2.14 (0.72 to 6.43)	0.288	2.02 (0.67 to 6.08)	0.302	2.1 (0.71 to 6.18)	0.296
Anticoagulant						
Past user	0.48 (0.05 to 4.78)	0.669	0.44 (0.04 to 4.48)	0.614	0.48 (0.05 to 4.94)	0.753
Current user	1.16 (0.31 to 4.39)	0.918	1.13 (0.3 to 4.35)	0.95	1.16 (0.31 to 4.31)	0.917
Glucocorticoid						
Past user	1.45 (0.43 to 4.9)	0.609	1.34 (0.4 to 4.49)	0.709	1.45 (0.43 to 4.88)	0.683
Current user	2.86 (1.28 to 6.4)	0.021	2.62 (1.17 to 5.86)	0.038	2.77 (1.23 to 6.24)	0.028
β2-agonist						
Past user	2.3 (0.37 to 14.3)	0.464	2.04 (0.32 to 13.1)	0.504	1.91 (0.31 to 11.8)	0.61
Current user	3.06 (1.3 to 7.22)	0.021	2.66 (1.15 to 6.15)	0.045	2.69 (1.16 to 6.2)	0.041
Muscarinic antagonist						
Past user	1.51 (0.3 to 7.58)	0.775	1.84 (0.35 to 9.65)	0.59	1.53 (0.31 to 7.5)	0.768
Current user	1.16 (0.46 to 2.89)	0.838	1.13 (0.45 to 2.84)	0.877	1.11 (0.45 to 2.75)	0.918
NSAID						
Past user	0.64 (0.12 to 3.39)	0.663	0.55 (0.1 to 2.94)	0.542	0.56 (0.11 to 2.92)	0.541
Current user	4.47 (1.28 to 15.6)	0.038	4.06 (1.15 to 14.3)	0.059	4.28 (1.24 to 14.7)	0.042
Vitamin D						
Past user	1.81 (0.54 to 6.09)	0.421	1.59 (0.48 to 5.25)	0.496	1.81 (0.55 to 5.98)	0.415
Current user	1.73 (0.76 to 3.93)	0.278	1.41 (0.62 to 3.17)	0.496	1.57 (0.7 to 3.51)	0.392
Proton pump inhibitor						
Past user	1.58 (0.3 to 8.28)	0.698	1.34 (0.26 to 6.98)	0.807	1.4 (0.28 to 7.17)	0.758
Current user	0.83 (0.38 to 1.8)	0.698	0.78 (0.36 to 1.7)	0.663	0.83 (0.38 to 1.82)	0.758
Statin						
Past user	0.64 (0.13 to 3.08)	0.719	0.7 (0.15 to 3.29)	0.729	0.79 (0.16 to 3.85)	0.952
Current user	1.23 (0.49 to 3.13)	0.731	1.29 (0.52 to 3.2)	0.719	1.44 (0.52 to 3.96)	0.684
Immunosuppressant						
Past user	1.39 (0.16 to 11.8)	0.847	1.23 (0.16 to 9.64)	0.936	1.24 (0.16 to 9.65)	0.93
Current user	2.37 (0.42 to 13.5)	0.473	2.33 (0.4 to 13.4)	0.429	2.34 (0.42 to 13.3)	0.479
**Complications post diagnosis (ref=No)**						
**Cardiovascular**						
Recurrent	2.41 (0.99 to 5.89)	0.107	2.44 (1 to 5.96)	0.101	2.5 (1.02 to 6.14)	0.091
Novel	0.38 (0.04 to 3.97)	0.465	0.49 (0.04 to 5.47)	0.627	0.44 (0.04 to 4.47)	0.604

For peer review only

**Respiratory**						
Recurrent	2.82 (0.92 to 8.71)	0.118	2.74 (0.9 to 8.38)	0.125	2.51 (0.83 to 7.6)	0.173
Novel	5.82 (1.75 to 19.3)	0.01	6.02 (1.8 to 20.2)	0.009	5.71 (1.73 to 18.9)	0.011
**Renal**						
Recurrent	1.67 (0.64 to 4.37)	0.419	1.33 (0.5 to 3.54)	0.716	1.68 (0.65 to 4.39)	0.408
Novel	1.08 (0.32 to 3.65)	0.988	0.75 (0.22 to 2.6)	0.722	0.98 (0.29 to 3.29)	0.988

	Adjusted OR (+Cardiovascular) (95% CI)	P value	Adjusted OR (+Renal) (95% CI)	P value	Adjusted OR (+Respiratory) (95% CI)	P value
**Demographics**						
Gender (ref=Female)						
Male	3.26 (1.57 to 7.13)	0.006	2.99 (1.44 to 6.47)	0.011	3.16 (1.54 to 6.8)	0.006
Ethnicity (ref=White)						
South Asian	1.8 (0.79 to 4.13)	0.207	1.73 (0.77 to 3.93)	0.248	1.85 (0.82 to 4.17)	0.218
Black	3.82 (1.45 to 10.4)	0.015	3.14 (1.18 to 8.61)	0.046	3.52 (1.34 to 9.51)	0.022
Other	0.23 (0.01 to 1.41)	0.207	0.26 (0.01 to 1.63)	0.263	0.26 (0.01 to 1.62)	0.305
Age group (ref=18-40)						
41-50	2.01 (0.16 to 47.4)	0.712	2.03 (0.17 to 47.6)	0.702	2.13 (0.18 to 49.8)	0.663
51-60	1.54 (0.13 to 35.9)	0.807	1.74 (0.15 to 40.5)	0.728	1.94 (0.16 to 45)	0.663
61-70	5.21 (0.77 to 105)	0.221	5.86 (0.87 to 118)	0.208	6.75 (1.03 to 135)	0.156
71-80	12.8 (2.07 to 252)	0.053	15.2 (2.36 to 305)	0.047	19.2 (3.19 to 373)	0.022
80+	16.7 (2.69 to 328)	0.035	20.5 (3.18 to 411)	0.031	26.8 (4.52 to 522)	0.011
**HPB disease (ref=No)**						
Cancer						
Yes	1.26 (0.13 to 12.2)	0.899	1.69 (0.18 to 15.5)	0.688	1.19 (0.14 to 10.1)	0.937
Pancreatic disease						
Acute	1.28 (0.36 to 4.59)	0.818	1.47 (0.42 to 5.14)	0.635	1.53 (0.44 to 5.29)	0.599
Chronic	2.97 (0.98 to 8.98)	0.134	3.11 (1.06 to 9.13)	0.118	3.22 (1.11 to 9.37)	0.096
Liver disease						
Mild	0.47 (0.17 to 1.29)	0.27	0.49 (0.18 to 1.3)	0.312	0.5 (0.19 to 1.33)	0.357
Moderate/Severe	0.53 (0.14 to 2.01)	0.482	0.66 (0.18 to 2.45)	0.635	0.6 (0.16 to 2.19)	0.592
Biliary disease						
Acute	3.25 (0.48 to 22.2)	0.36	2.53 (0.38 to 16.6)	0.501	2.56 (0.38 to 17)	0.552
Chronic	0.65 (0.25 to 1.71)	0.482	0.67 (0.26 to 1.7)	0.539	0.67 (0.26 to 1.7)	0.592
**Comorbidities (ref=No)**						
Diabetes	1.78 (0.76 to 4.19)	0.236	1.76 (0.75 to 4.13)	0.291	1.86 (0.79 to 4.38)	0.283
Hypertension	5.73 (0.62 to 52.9)	0.185	5.62 (0.64 to 49.5)	0.216	6.16 (0.72 to 52.7)	0.174
Cholesterol	1.05 (0.45 to 2.42)	0.989	1.21 (0.53 to 2.75)	0.731	1.26 (0.56 to 2.84)	0.719
Cardiovascular	2.82 (1.21 to 6.59)	0.026	2.6 (1.06 to 6.34)	0.065	3.01 (1.27 to 7.14)	0.022
Renal	1.28 (0.57 to 2.88)	0.623	1.72 (0.8 to 3.7)	0.248	1.73 (0.8 to 3.73)	0.271
Respiratory	0.72 (0.34 to 1.51)	0.428	0.83 (0.41 to 1.71)	0.695	0.85 (0.42 to 1.73)	0.743

**Lifestyle factors (ref=Never)**						
Smoker						
Past	1.75 (0.76 to 4.02)	0.269	1.79 (0.79 to 4.07)	0.298	1.92 (0.84 to 4.36)	0.22
Current	0.45 (0.08 to 2.48)	0.436	0.44 (0.08 to 2.48)	0.429	0.48 (0.08 to 2.73)	0.56
Drinker						
Past	1.09 (0.37 to 3.26)	0.958	1.27 (0.43 to 3.76)	0.783	1.19 (0.41 to 3.47)	0.879
Current	1.5 (0.55 to 4.09)	0.588	1.6 (0.59 to 4.33)	0.492	1.37 (0.52 to 3.61)	0.826
Substance user						
Past	0.33 (0.04 to 2.9)	0.437	0.48 (0.06 to 4.03)	0.61	0.65 (0.08 to 5.26)	0.843
Current	1.44 (0.36 to 5.87)	0.744	1.77 (0.45 to 6.91)	0.564	2.12 (0.56 to 8.07)	0.428
Obese						
Past	1.14 (0.44 to 2.96)	0.996	1.4 (0.55 to 3.52)	0.658	1.45 (0.58 to 3.63)	0.679
Current	1 (0.42 to 2.36)	0.996	1.05 (0.45 to 2.45)	0.991	1.03 (0.44 to 2.39)	0.991
**Prescription medication use (ref=Non-user)**						
ACE inhibitor						
Past user	0.93 (0.34 to 2.57)	0.989	0.92 (0.34 to 2.52)	0.967	1.02 (0.38 to 2.75)	0.988
Current user	2.25 (0.89 to 5.73)	0.146	2.07 (0.81 to 5.31)	0.26	2.32 (0.9 to 5.93)	0.16
Angiotensin receptor blocker						
Past user	0.86 (0.11 to 6.75)	0.982	1.03 (0.14 to 7.79)	0.988	1.14 (0.15 to 8.91)	0.988
Current user	0.9 (0.32 to 2.55)	0.982	0.84 (0.31 to 2.27)	0.915	0.85 (0.31 to 2.29)	0.928
Aldosterone agonist						
Past user	0.93 (0.2 to 4.24)	0.989	1.07 (0.23 to 4.96)	0.988	1.34 (0.29 to 6.13)	0.805
Current user	1.03 (0.22 to 4.77)	0.989	1.32 (0.28 to 6.08)	0.906	1.32 (0.28 to 6.19)	0.805
β-blocker						
Past user	0.44 (0.09 to 2.13)	0.341	0.55 (0.12 to 2.59)	0.502	0.69 (0.15 to 3.12)	0.699
Current user	0.38 (0.16 to 0.92)	0.053	0.54 (0.24 to 1.24)	0.208	0.64 (0.29 to 1.43)	0.396
Calcium channel blocker						
Past user	0.09 (0.01 to 0.56)	0.016	0.13 (0.02 to 0.75)	0.045	0.16 (0.03 to 0.91)	0.078
Current user	0.64 (0.27 to 1.55)	0.364	0.76 (0.32 to 1.8)	0.587	0.91 (0.39 to 2.12)	0.977
α-agonist						
Past user	0 (0 to Inf)	0.992	0 (0 to Inf)	0.992	0 (0 to Inf)	0.992
Thiazide						
Past user	0 (0 to Inf)	0.993	0 (0 to Inf)	0.993	0 (0 to Inf)	0.993
Current user	0.46 (0.08 to 2.63)	0.44	0.73 (0.13 to 3.97)	0.816	0.7 (0.13 to 3.93)	0.862
Antiplatelet						
Past user	0.7 (0.3 to 1.61)	0.44	0.87 (0.39 to 1.94)	0.816	1 (0.46 to 2.2)	0.993
Antiarrhythmic						
Past user	0.41 (0.04 to 3.95)	0.493	0.44 (0.05 to 4.14)	0.525	0.51 (0.05 to 4.69)	0.618
Current user	1.66 (0.55 to 5.01)	0.466	1.9 (0.64 to 5.68)	0.328	2.17 (0.73 to 6.46)	0.272
Anticoagulant						
Past user	0.37 (0.04 to 3.85)	0.506	0.47 (0.05 to 4.68)	0.651	0.48 (0.05 to 4.84)	0.76
Current user	0.96 (0.26 to 3.57)	0.989	1.1 (0.29 to 4.18)	0.982	1.19 (0.32 to 4.46)	0.881
Glucocorticoid						
Past user	1.54 (0.46 to 5.18)	0.537	1.56 (0.46 to 5.23)	0.524	1.54 (0.46 to 5.21)	0.538
Current user	2.97 (1.31 to 6.76)	0.018	2.82 (1.26 to 6.31)	0.024	3.21 (1.38 to 7.45)	0.013

For peer review only

β2-agonist						
Past user	2 (0.3 to 13.1)	0.523	1.86 (0.3 to 11.7)	0.563	3.7 (0.53 to 25.8)	0.25
Current user	2.54 (1.09 to 5.93)	0.052	2.69 (1.17 to 6.21)	0.041	4.8 (1.7 to 13.6)	0.008
Muscarinic antagonist						
Past user	1.67 (0.33 to 8.45)	0.671	1.61 (0.32 to 7.99)	0.703	1.56 (0.32 to 7.58)	0.766
Current user	1 (0.4 to 2.51)	0.998	1.03 (0.41 to 2.59)	0.988	1.19 (0.46 to 3.08)	0.796
NSAID						
Past user	0.64 (0.12 to 3.47)	0.676	0.65 (0.12 to 3.45)	0.683	0.6 (0.11 to 3.14)	0.643
Current user	3.81 (1.08 to 13.5)	0.063	4.66 (1.29 to 16.8)	0.047	4.23 (1.22 to 14.7)	0.046
Vitamin D						
Past user	1.52 (0.45 to 5.1)	0.552	1.67 (0.51 to 5.49)	0.449	1.96 (0.58 to 6.6)	0.344
Current user	1.38 (0.6 to 3.15)	0.552	1.42 (0.62 to 3.26)	0.449	1.75 (0.75 to 4.05)	0.344
Proton pump inhibitor						
Past user	1.05 (0.21 to 5.37)	0.989	1.23 (0.24 to 6.21)	0.894	1.39 (0.27 to 7.2)	0.838
Current user	0.61 (0.26 to 1.4)	0.306	0.78 (0.36 to 1.73)	0.685	0.88 (0.41 to 1.91)	0.838
Statin						
Past user	0.62 (0.13 to 3.04)	0.699	0.68 (0.14 to 3.28)	0.701	0.81 (0.18 to 3.74)	0.879
Current user	1.17 (0.47 to 2.95)	0.816	1.31 (0.52 to 3.27)	0.701	1.52 (0.62 to 3.69)	0.511
Immunosuppressant						
Past user	1.59 (0.21 to 11.9)	0.725	1.31 (0.16 to 10.5)	0.889	1.25 (0.16 to 9.91)	0.923
Current user	3.31 (0.59 to 18.5)	0.259	2.18 (0.38 to 12.6)	0.481	2.5 (0.45 to 13.9)	0.425
**Complications post diagnosis (ref=No)**						
**Cardiovascular**						
Recurrent	2.53 (1.04 to 6.12)	0.071	2.33 (0.92 to 5.88)	0.149	2.7 (1.1 to 6.68)	0.062
Novel	0.44 (0.04 to 4.47)	0.547	0.44 (0.04 to 4.52)	0.607	0.46 (0.05 to 4.65)	0.569
**Respiratory**						
Recurrent	2.24 (0.72 to 6.97)	0.205	2.49 (0.82 to 7.55)	0.179	2.53 (0.84 to 7.63)	0.15
Novel	6.17 (1.81 to 21)	0.009	5.85 (1.76 to 19.4)	0.01	5.77 (1.75 to 19)	0.009
**Renal**						
Recurrent	1.06 (0.37 to 3.02)	0.989	1.72 (0.66 to 4.46)	0.34	1.75 (0.67 to 4.57)	0.358
Novel	0.7 (0.2 to 2.51)	0.734	1 (0.3 to 3.34)	0.997	1.02 (0.3 to 3.44)	0.988

Odds ratios (ORs), except the crude ones, are mutually adjusted for gender, ethnicity, and age group, and also for additional conditions when mentioned inside the parenthesis. Dichotomous age groups (over and under 60) are used for controlling for all categories except demographics. All P values presented, except for the crude odds ratios, are Benjamini-Hochberg corrected.

For peer review only

STROBE Statement

Checklist of items that should be included in reports of observational studies

Section/Topic	Item No	Recommendation	Reported on Page No
**Title and abstract**	1	(*a*) Indicate the study's design with a commonly used term in the title or the abstract	1,2
		(*b*) Provide in the abstract an informative and balanced summary of what was done and what was found	2
**Introduction**			
Background/rationale	2	Explain the scientific background and rationale for the investigation being reported	4,5
Objectives	3	State specific objectives, including any prespecified hypotheses	4,5
**Methods**			
Study design	4	Present key elements of study design early in the paper	6
Setting	5	Describe the setting, locations, and relevant dates, including periods of recruitment, exposure, follow-up, and data collection	6,7
Participants	6	(*a*) *Cohort study*—Give the eligibility criteria, and the sources and methods of selection of participants. Describe methods of follow-up<br>*Case-control study*—Give the eligibility criteria, and the sources and methods of case ascertainment and control selection. Give the rationale for the choice of cases and controls<br>*Cross-sectional study*—Give the eligibility criteria, and the sources and methods of selection of participants	6,7
		(*b*) *Cohort study*—For matched studies, give matching criteria and number of exposed and unexposed<br>*Case-control study*—For matched studies, give matching criteria and the number of controls per case	
Variables	7	Clearly define all outcomes, exposures, predictors, potential confounders, and effect modifiers. Give diagnostic criteria, if applicable	7-9, Table 1
Data sources/measurement	8*	For each variable of interest, give sources of data and details of methods of assessment (measurement). Describe comparability of assessment methods if there is more than one group	6-9, Supplemental Tables 1,2
Bias	9	Describe any efforts to address potential sources of bias	7
Study size	10	Explain how the study size was arrived at	6,7
Quantitative variables	11	Explain how quantitative variables were handled in the analyses. If applicable, describe which groupings were chosen and why	9,10
Statistical methods	12	(*a*) Describe all statistical methods, including those used to control for confounding	9,10
		(*b*) Describe any methods used to examine subgroups and interactions	
		(*c*) Explain how missing data were addressed	
		(*d*) *Cohort study*—If applicable, explain how loss to follow-up was addressed<br>*Case-control study*—If applicable, explain how matching of cases and controls was addressed<br>*Cross-sectional study*—If applicable, describe analytical methods taking account of sampling strategy	

For peer review only - http://bmjopen.bmj.com/site/about/guidelines.xhtml

(*e*) Describe any sensitivity analyses

Section/Topic	Item No	Recommendation	Reported on Page No
**Results**			
Participants	13*	(a) Report numbers of individuals at each stage of study—eg numbers potentially eligible, examined for eligibility, confirmed eligible, included in the study, completing follow-up, and analysed	11, Figure 1
		(b) Give reasons for non-participation at each stage	
		(c) Consider use of a flow diagram	
Descriptive data	14*	(a) Give characteristics of study participants (eg demographic, clinical, social) and information on exposures and potential confounders	11-19, Tables 2,3, Supplemental table 3
		(b) Indicate number of participants with missing data for each variable of interest	
		(c) *Cohort study*—Summarise follow-up time (eg, average and total amount)	
Outcome data	15*	*Cohort study*—Report numbers of outcome events or summary measures over time	11
		Case-control study—Report numbers in each exposure category, or summary measures of exposure	
		Cross-sectional study—Report numbers of outcome events or summary measures	
Main results	16	(*a*) Give unadjusted estimates and, if applicable, confounder-adjusted estimates and their precision (eg, 95% confidence interval). Make clear which confounders were adjusted for and why they were included	19-21, Figures 2,3, Supplemental tables 4,5
		(*b*) Report category boundaries when continuous variables were categorized	
		(*c*) If relevant, consider translating estimates of relative risk into absolute risk for a meaningful time period	
Other analyses	17	Report other analyses done—eg analyses of subgroups and interactions, and sensitivity analyses	20, Supplemental table 6
**Discussion**			
Key results	18	Summarise key results with reference to study objectives	22-24
Limitations	19	Discuss limitations of the study, taking into account sources of potential bias or imprecision. Discuss both direction and magnitude of any potential bias	25
Interpretation	20	Give a cautious overall interpretation of results considering objectives, limitations, multiplicity of analyses, results from similar studies, and other relevant evidence	22-24
Generalisability	21	Discuss the generalisability (external validity) of the study results	24-26
**Other Information**			

For peer review only - http://bmjopen.bmj.com/site/about/guidelines.xhtml

Funding	22	Give the source of funding and the role of the funders for the present study and, if applicable, for the original study on which the present article is based	27

**Give information separately for cases and controls in case-control studies and, if applicable, for exposed and unexposed groups in cohort and cross-sectional studies.*

Note: An Explanation and Elaboration article discusses each checklist item and gives methodological background and published examples of transparent reporting. The STROBE checklist is best used in conjunction with this article (freely available on the Web sites of PLoS Medicine at http://www.plosmedicine.org/, Annals of Internal Medicine at http://www.annals.org/, and Epidemiology at http://www.epidem.com/). Information on the STROBE Initiative is available at www.strobe-statement.org.

For peer review only
